# Genome-wide association and genomic prediction of breeding values for fatty acid composition in subcutaneous adipose and *longissimus lumborum* muscle of beef cattle

**DOI:** 10.1186/s12863-015-0290-0

**Published:** 2015-11-21

**Authors:** Liuhong Chen, Chinyere Ekine-Dzivenu, Michael Vinsky, John Basarab, Jennifer Aalhus, Mike E. R. Dugan, Carolyn Fitzsimmons, Paul Stothard, Changxi Li

**Affiliations:** Department of Agricultural, Food and Nutritional Science, University of Alberta, Edmonton, AB T6G 2P5 Canada; Lacombe Research Centre, Agriculture and Agri-Food Canada, 6000 C&E Trail, Lacombe, AB T4L 1 W1 Canada; Lacombe Research Centre, Alberta Agriculture and Forestry, 6000 C & E Trail, Lacombe, AB T4L 1 W1 Canada

**Keywords:** Fatty acid composition, Beef cattle, Genome-wide association study, Genomic prediction, Single nucleotide polymorphism

## Abstract

**Background:**

Identification of genetic variants that are associated with fatty acid composition in beef will enhance our understanding of host genetic influence on the trait and also allow for more effective improvement of beef fatty acid profiles through genomic selection and marker-assisted diet management. In this study, 81 and 83 fatty acid traits were measured in subcutaneous adipose (SQ) and *longissimus lumborum* muscle (LL), respectively, from 1366 purebred and crossbred beef steers and heifers that were genotyped on the Illumina BovineSNP50 Beadchip. The objective was to conduct genome-wide association studies (GWAS) for the fatty acid traits and to evaluate the accuracy of genomic prediction for fatty acid composition using genomic best linear unbiased prediction (GBLUP) and Bayesian methods.

**Results:**

In total, 302 and 360 significant SNPs spanning all autosomal chromosomes were identified to be associated with fatty acid composition in SQ and LL tissues, respectively. Proportions of total genetic variance explained by individual significant SNPs ranged from 0.03 to 11.06 % in SQ, and from 0.005 to 24.28 % in the LL muscle. Markers with relatively large effects were located near fatty acid synthase (*FASN*), stearoyl-CoA desaturase (*SCD*), and thyroid hormone responsive (*THRSP*) genes. For the majority of the fatty acid traits studied, the accuracy of genomic prediction was relatively low (<0.40). Relatively high accuracies (> = 0.50) were achieved for 10:0, 12:0, 14:0, 15:0, 16:0, 9c-14:1, 12c-16:1, 13c-18:1, and health index (HI) in LL, and for 12:0, 14:0, 15:0, 10 t,12c-18:2, and 11 t,13c + 11c,13 t-18:2 in SQ. The Bayesian method performed similarly as GBLUP for most of the traits but substantially better for traits that were affected by SNPs of large effects as identified by GWAS.

**Conclusions:**

Fatty acid composition in beef is influenced by a few host genes with major effects and many genes of smaller effects. With the current training population size and marker density, genomic prediction has the potential to predict the breeding values of fatty acid composition in beef cattle at a moderate to relatively high accuracy for fatty acids that have moderate to high heritability.

**Electronic supplementary material:**

The online version of this article (doi:10.1186/s12863-015-0290-0) contains supplementary material, which is available to authorized users.

## Background

Dietary fats influence risks for developing cardiovascular disease, obesity and various forms of cancer, and have led to recommendations to limit consumption of some foods including beef [1]. Recommendations to limit beef consumption are mainly related to its relatively high content of saturated fatty acids (SFAs) as SFA consumption is believed to have negative effects on human health [2, 3]. Beef, however, is also a natural source of polyunsaturated fatty acid (PUFA) biohydrogenation intermediates (BHI) including vaccenic acid (11 t-18:1) and conjugated linoleic acids (CLAs), which have a number of purported health benefits [4–6]. In addition, beef is rich in monounsaturated fatty acids (MUFAs), in particular oleic acid 9c-18:1, which is the main fatty acid found in healthy Mediterranean diets, and may also contribute positively to beef flavour and tenderness [7]. Considerable efforts have, therefore, gone into improving beef fatty acid profiles in beef to meet the consumers’ growing demand for more nutritious, healthier and more palatable meat. Diet is known to have a major influence on beef fatty acid composition [8], but the use of genomic technologies to improve beef fatty acid profiles have not been thoroughly investigated [9].

The fatty acid composition of beef is a complex trait with heritability estimates ranging from near 0 to 0.73, depending on populations and the types of fatty acid [10–18]. To further elucidate the genetic control of host animals on fatty acid composition, chromosomal regions or quantitative trait loci (QTL) and candidate genes that are associated with fatty acid composition in beef cattle have been identified on multiple chromosomes based on low density DNA markers [19–21], and based on candidate gene DNA marker association analyses [22–36]. Genome-wide association studies (GWAS) using a relatively high density of single nucleotide polymorphism (SNP) markers (e.g. Illumina BovineSNP50 Beadchip) in recent years have assisted in the search for DNA markers associated with the fatty acid composition of beef, but studies are limited to a small number of fatty acids in certain beef breeds [7, 37, 38]. Saatchi *et al*. [16] analyzed 49 fatty acid traits in steaks of Angus beef cattle and reported results of GWAS and genomic prediction of direct genomic breeding values of the fatty acid traits. Onogi et al. [39] also reported genomic prediction for 8 fatty acid traits in Japanese Black cattle. Many fatty acids with potential health value (i.e. PUFA-BHI) were, however, not reported in those studies. In this study, we comprehensively analyzed fatty acid profiles and report GWAS and genomic prediction of breeding values for 81 and 83 individual and grouped fatty acids in subcutaneous adipose (SQ) and *longissimus lumborum* muscle (LL), respectively, in Canadian beef cattle populations.

## Results and discussion

### Descriptive statistics and genomic heritability estimates

Summary statistics and genomic heritability estimates for the 81 fatty acid traits of SQ and 83 fatty acid traits of LL are presented in Table 1. In general, the estimates of heritability for the same fatty acids are comparable in both the adipose and muscle tissues, with a correlation coefficient of 0.61. Relatively higher (>0.40) heritability estimates were found for 10:0, 12:0, 18:0, ai15:0, 9c-14:1, 9c-16:1, 13c-18:1, 18:3n-3, 18:2n-6, n-3, n-6, sumtrans 18:1, total PUFA, P/S, and P/(S + B) in the SQ tissue, and for 12:0, 14:0, 16:0, 9c-14:1, 9c-16:1, 12c-16:1, 9c-18:1, 13c-18:1, SFA, SFA + BFA, MUFA, n-6/n-3, and health index (HI) in the LL muscle, which suggests greater direct host genetic effects on these traits in the corresponding tissues. Very low (<0.05) or zero heritability were observed for 22:0, 7c-17:1, 12 t-18:1, 15 t-18:1, 6 t,8 t:18:2, 7 t,9 t-18:2, 9 t,11 t-18:2, 10 t,12 t-18:2, 12 t,14 t-18:2, and n-6/n-3 in the SQ tissue, and for 7c-17:1, 15 t-18:1, 6 t,8 t-18:2, 7 t,9 t-18:2, 7 t,9c-18:2, 8 t,10 t-18:2, 12 t,14 t-18:2 in the LL muscle, which indicates weak host direct genetic control on these traits. In general, the heritability estimates for the fatty acids in this study are in line with those reported in other studies [10, 12, 15]. Therefore, the genomic estimated additive genetic variance and heritability for the fatty acid traits were further used in the calculation of total genetic variance explained by significant markers identified in GWAS and in the derivation of realised accuracy of genomic prediction in this study.

**Table 1:** Summary statistics of mean, standard deviation (SD), additive genetic variance (*σ*_*a*_^2^), and heritability estimates (h^2^ ± SE)

	Subcutaneous adipose			*Longissimus lumborum*		
Trait^a^	Mean (SD)	*σ*_*a*_^2^ × 10^4^	h^2^ ± SE	Mean (SD)	*σ*_*a*_^2^ × 10^4^	h^2^ ± SE
10:0	0.051 (0.014)	0.66	0.43 ± 0.06	0.056 (0.012)	0.60	0.37 ± 0.09
12:0	0.071 (0.017)	1.60	0.53 ± 0.08	0.072 (0.015)	1.30	0.57 ± 0.07
13:0	0.028 (0.010)	0.28	0.26 ± 0.07	0.027 (0.009)	0.14	0.16 ± 0.06
14:0	3.204 (0.599)	1605.35	0.35 ± 0.11	2.804 (0.486)	1292.53	0.53 ± 0.10
15:0	0.642 (0.167)	90.38	0.22 ± 0.10	0.502 (0.111)	38.96	0.23 ± 0.10
16:0	25.092 (2.594)	12965.10	0.21 ± 0.06	24.607 (2.056)	11278.40	0.42 ± 0.08
17:0	1.709 (0.445)	575.54	0.32 ± 0.11	1.548 (0.329)	326.58	0.31 ± 0.12
18:0	10.545 (1.956)	14701.00	0.41 ± 0.07	12.406 (1.417)	7779.92	0.38 ± 0.09
19:0	0.108 (0.032)	0.90	0.06 ± 0.03	0.090 (0.029)	0.82	0.08 ± 0.04
20:0	0.082 (0.019)	0.75	0.20 ± 0.06	0.089 (0.016)	0.55	0.18 ± 0.06
22:0	0.033 (0.009)	0.000036	0.00 ± 0.03	0.069 (0.021)	0.73	0.09 ± 0.05
24:0	0.035 (0.015)	0.60	0.22 ± 0.07	0.151 (0.070)	7.65	0.21 ± 0.07
SFA	41.598 (3.469)	36697.00	0.29 ± 0.07	42.421 (2.695)	25765.90	0.43 ± 0.08
iso14:0	0.031 (0.012)	0.14	0.12 ± 0.05	0.027 (0.008)	0.050	0.10 ± 0.05
iso15:0	0.109 (0.026)	1.11	0.22 ± 0.06	0.082 (0.015)	0.52	0.25 ± 0.07
ai15:0	0.180 (0.048)	11.26	0.52 ± 0.08	0.140 (0.028)	3.07	0.30 ± 0.10
iso16:0	0.177 (0.041)	5.97	0.34 ± 0.08	0.140 (0.027)	1.98	0.22 ± 0.08
iso17:0	0.382 (0.062)	12.74	0.31 ± 0.08	0.345 (0.062)	5.48	0.11 ± 0.05
ai17:0	0.672 (0.095)	22.50	0.22 ± 0.07	0.489 (0.076)	15.93	0.19 ± 0.08
iso18:0	0.163 (0.037)	3.25	0.23 ± 0.07	0.133 (0.028)	1.95	0.21 ± 0.08
BFA	1.714 (0.261)	225.65	0.29 ± 0.08	1.356 (0.203)	94.08	0.16 ± 0.07
SFA + BFA	43.312 (3.561)	38438.00	0.28 ± 0.07	43.777 (2.681)	25797.30	0.43 ± 0.08
9c-14:1	1.046 (0.390)	542.74	0.43 ± 0.08	0.640 (0.184)	185.50	0.59 ± 0.07
9c-15:1	0.034 (0.012)	0.38	0.23 ± 0.08	0.026 (0.009)	0.073	0.08 ± 0.04
7c-16:1	0.140 (0.025)	1.90	0.34 ± 0.07	0.136 (0.019)	1.20	0.30 ± 0.09
9c-16:1	4.247 (1.096)	4450.52	0.42 ± 0.07	3.408 (0.564)	1730.54	0.64 ± 0.07
11 t-16:1	0.047 (0.012)	0.21	0.12 ± 0.05	0.042 (0.012)	0.12	0.06 ± 0.04
12c-16:1	0.239 (0.090)	23.77	0.36 ± 0.07	0.168 (0.044)	9.70	0.53 ± 0.07
7c-17:1	0.023 (0.009)	0	0	0.025 (0.013)	0.040	0.02 ± 0.03
9c-17:1	1.377 (0.340)	267.08	0.19 ± 0.09	1.191 (0.298)	130.35	0.15 ± 0.07
9c-18:1	37.917 (4.343)	30916.80	0.13 ± 0.05	36.679 (2.997)	35344.80	0.47 ± 0.07
11c-18:1	1.960 (1.736)	1591.98	0.05 ± 0.04	1.836 (0.244)	152.64	0.30 ± 0.10
12c-18:1	0.260 (0.079)	6.88	0.09 ± 0.04	0.227 (0.071)	6.70	0.13 ± 0.06
13c-18:1	0.487 (0.159)	83.84	0.44 ± 0.07	0.396 (0.089)	40.17	0.57 ± 0.07
14c-18:1	0.053 (0.011)	0.17	0.12 ± 0.05	0.048 (0.009)	0.13	0.19 ± 0.06
15c-18:1	0.248 (0.060)	13.68	0.38 ± 0.07	0.204 (0.044)	5.62	0.28 ± 0.07
6 t + 8 t-18:1	0.275 (0.121)	38.18	0.34 ± 0.06	0.193 (0.085)	14.30	0.21 ± 0.07
9 t-18:1	0.291 (0.094)	18.74	0.27 ± 0.06	0.232 (0.067)	9.04	0.22 ± 0.07
10 t-18:1	2.908 (1.685)	10759.90	0.38 ± 0.11	2.028 (1.119)	4230.39	0.36 ± 0.10
11 t-18:1	0.546 (0.234)	119.36	0.17 ± 0.07	0.441 (0.164)	70.08	0.26 ± 0.08
12 t-18:1	0.184 (0.172)	0.00012	0	0.137 (0.029)	1.06	0.13 ± 0.05
15 t-18:1	0.169 (0.179)	1.45	0.00 ± 0.03	0.130 (0.079)	2.25	0.04 ± 0.05
16 t-18:1	0.113 (0.037)	2.51	0.11 ± 0.05	0.092 (0.026)	1.78	0.19 ± 0.08
sumtrans18:1	4.486 (1.687)	10976.60	0.42 ± 0.09	3.252 (1.131)	4357.97	0.37 ± 0.09
9c-20:1	0.107 (0.019)	0.82	0.22 ± 0.08	0.090 (0.013)	0.20	0.08 ± 0.04
11c-20:1	0.270 (0.075)	15.80	0.37 ± 0.07	0.198 (0.035)	5.20	0.38 ± 0.08
MUFA	52.941 (3.583)	37044.00	0.26 ± 0.06	48.565 (2.691)	26915.70	0.47 ± 0.06
9c,13 t + 8 t,12c-18:2	0.242 (0.045)	6.07	0.30 ± 0.09	0.165 (0.029)	1.94	0.24 ± 0.08
9c,15c-18:2	0.184 (0.044)	5.29	0.27 ± 0.07	0.178 (0.036)	3.28	0.25 ± 0.08
8 t,13c-18:2	0.165 (0.043)	4.38	0.19 ± 0.08	0.121 (0.025)	0.63	0.10 ± 0.05
11 t,15c-18:2	0.162 (0.100)	32.52	0.32 ± 0.08	0.122 (0.070)	17.25	0.37 ± 0.08
9c,11 t + 9 t,11c-18:2	0.471 (0.358)	33.83	0.25 ± 0.06	0.257 (0.062)	9.26	0.16 ± 0.06
6 t,8 t-18:2	0.0024 (0.003)	0	0	0.0019 (0.004)	0.0002	0.00 ± 0.03
7 t,9c-18:2	0.108 (0.083)	11.38	0.25 ± 0.09	0.060 (0.060)	1.22	0.03 ± 0.03
12 t,14 t-18:2	0.0088 (0.011)	0.041	0.02 ± 0.01	0.0061 (0.008)	0.023	0.03 ± 0.02
11 t,13 t-18:2	0.0083 (0.006)	0.017	0.10 ± 0.04	0.0060 (0.002)	0.0056	0.15 ± 0.05
10 t,12 t-18:2	0.010 (0.005)	0	0	0.0061 (0.002)	0.0049	0.12 ± 0.05
9 t,11 t-18:2	0.014 (0.010)	0	0	0.0092 (0.003)	0.0083	0.08 ± 0.04
8 t,10 t-18:2	0.0026 (0.002)	0.0020	0.08 ± 0.04	0.0017 (0.002)	0.00084	0.04 ± 0.04
7 t,9 t-18:2	0.0069 (0.005)	0.0020	0.02 ± 0.02	0.0041 (0.003)	0.0010	0.01 ± 0.02
12 t,14c + 12c,14 t −18:2	0.012 (0.010)	0.042	0.07 ± 0.03	0.0068 (0.003)	0.018	0.21 ± 0.07
11 t,13c + 11c,13 t −18:2	0.024 (0.024)	0.15	0.15 ± 0.05	0.012 (0.005)	0.031	0.14 ± 0.06
10 t,12c-18:2	0.025 (0.012)	0.32	0.24 ± 0.07	0.018 (0.010)	0.065	0.07 ± 0.04
8 t,10c-18:2	0.012 (0.010)	0.022	0.11 ± 0.05	0.0079 (0.003)	0.018	0.21 ± 0.07
Total CLA	0.704 (0.493)	77.85	0.30 ± 0.06	0.395 (0.080)	11.21	0.15 ± 0.05
18:2n-6	1.876 (0.587)	1032.59	0.53 ± 0.08	4.387 (1.612)	5072.90	0.39 ± 0.08
18:3n-3	0.211 (0.055)	11.32	0.43 ± 0.07	0.297 (0.082)	8.60	0.20 ± 0.06
18:3n-6	0.0023 (0.006)	0.027	0.06 ± 0.05	0.043 (0.016)	0.51	0.16 ± 0.07
20:2n-6	0.038 (0.013)	0.22	0.19 ± 0.05	0.068 (0.022)	0.41	0.11 ± 0.05
20:3n-6	0.059 (0.017)	0.32	0.08 ± 0.04	0.292 (0.098)	23.30	0.32 ± 0.08
20:3n-9	0.017 (0.016)	0.12	0.06 ± 0.04	0.066 (0.025)	1.28	0.20 ± 0.06
20:4n-6	0.040 (0.012)	0.22	0.15 ± 0.05	1.000 (0.412)	277.29	0.25 ± 0.07
20:5n-3	*ND*	*NA*	*NA*	0.029 (0.009)	0.049	0.05 ± 0.04
22:4n-6	0.031 (0.011)	0.19	0.13 ± 0.05	0.136 (0.045)	4.67	0.20 ± 0.08
22:5n-3	0.017 (0.010)	0.05	0.11 ± 0.05	0.332 (0.126)	28.06	0.20 ± 0.07
22:6n-3	*ND*	*NA*	*NA*	0.046 (0.023)	0.78	0.16 ± 0.05
PUFA	2.290 (0.627)	1252.63	0.51 ± 0.08	6.695 (2.231)	8788.56	0.31 ± 0.08
n-3	0.228 (0.054)	10.59	0.40 ± 0.07	0.704 (0.208)	58.08	0.17 ± 0.06
n-6	2.046 (0.602)	1058.05	0.51 ± 0.08	5.926 (2.107)	8016.81	0.34 ± 0.08
n-6/n-3	9.263 (5.078)	2605.22	0.01 ± 0.02	8.628 (2.526)	9313.42	0.42 ± 0.09
P/S	0.056 (0.016)	0.77	0.50 ± 0.07	0.160 (0.058)	6.60	0.30 ± 0.09
P/(S + B)	0.053 (0.015)	0.69	0.50 ± 0.08	0.155 (0.056)	6.03	0.30 ± 0.09
HI	1.488 (0.265)	236.45	0.32 ± 0.08	1.566 (0.232)	201.90	0.47 ± 0.08

^a^The concentrations of fatty acids were expressed as a percentage of fatty acid methyl esters (FAME) quantified. c = cis, t = trans. SFA = 10:0 + 12:0 + 13:0 + 14:0 + 15:0 + 16:0 + 17:0 + 18:0 + 19:0 + 20:0 + 22:0 + 24:0; BFA = iso14:0 + iso15:0 + ai15:0 + iso16:0 + iso17:0 + ai17:0 + iso18:0; SFA + BFA: sum of SFA and BFA; sumtrans18:1 = 6 t/8 t-18:1 + 9 t-18:1 + 10 t-18:1 + 11 t-18:1 + 12 t-18:1 + 15 t-18:1 + 16 t-18:1; MUFA = 9c-14:1 + 9c-15:1 + 7c-16:1 + 9c-16:1 + 11 t-16:1 + 12c-16:1 + 7c-17:1 + 9c-17:1 + 9c-18:1 + 11c-18:1 + 12c-18:1 + 13c-18:1 + 14c-18:1 + 15c-18:1 + 9c-20:1 + 11c-20 + 6 t/8 t-18:1 + 9 t-18:1 + 10 t-18:1 + 11 t-18:1 + 12 t-18:1 + 15 t-18:1 + 16 t-18:1; Total CLA = 9c,11 t + 9 t,11c-18:2 + 6 t,8 t-18:2 + 7 t,9c-18:2 + 12 t,(14 t-18:2 + 11 t,13 t)-18:2 + 10 t,12 t-18:2 + 9 t,11 t-18:2 + 8 t,10 t-18:2 + 7 t,9 t-18:2 + (12 t,14c + 12c,14 t) -18:2 + (11 t,13c + 11c,13 t)-18:2 + 10 t,12c-18:2 + 8 t,10c-18:2; PUFA = 18:2n-6 + 18:3n-6 + 18:3n-3 + 20:2n-6 + 20:3n-9 + 20:3n-6 + 20:4n-6 + 22:4n-6 + 22:5n-3+ 22:6n-3; n-3 = 18:3n-3 + 20:5n-3 + 22:5n-3 + 22:6n-3; n-6 = 18:2n-6 + 18:3n-6 + 20:2n-6 + 20:3n-6 + 20:4n-6 + 22:4n-6; n-6/n-3: ratio between n-6 and n-3; P/S = PUFA/SFA; P/(S + B) = PUFA/(SFA + BFA); HI = (MUFA + PUFA) / (4 × 14:0 + 16:0)

*ND* not detected, *NA* not applicable

### Genome-wide association study

**Fig. 1:**
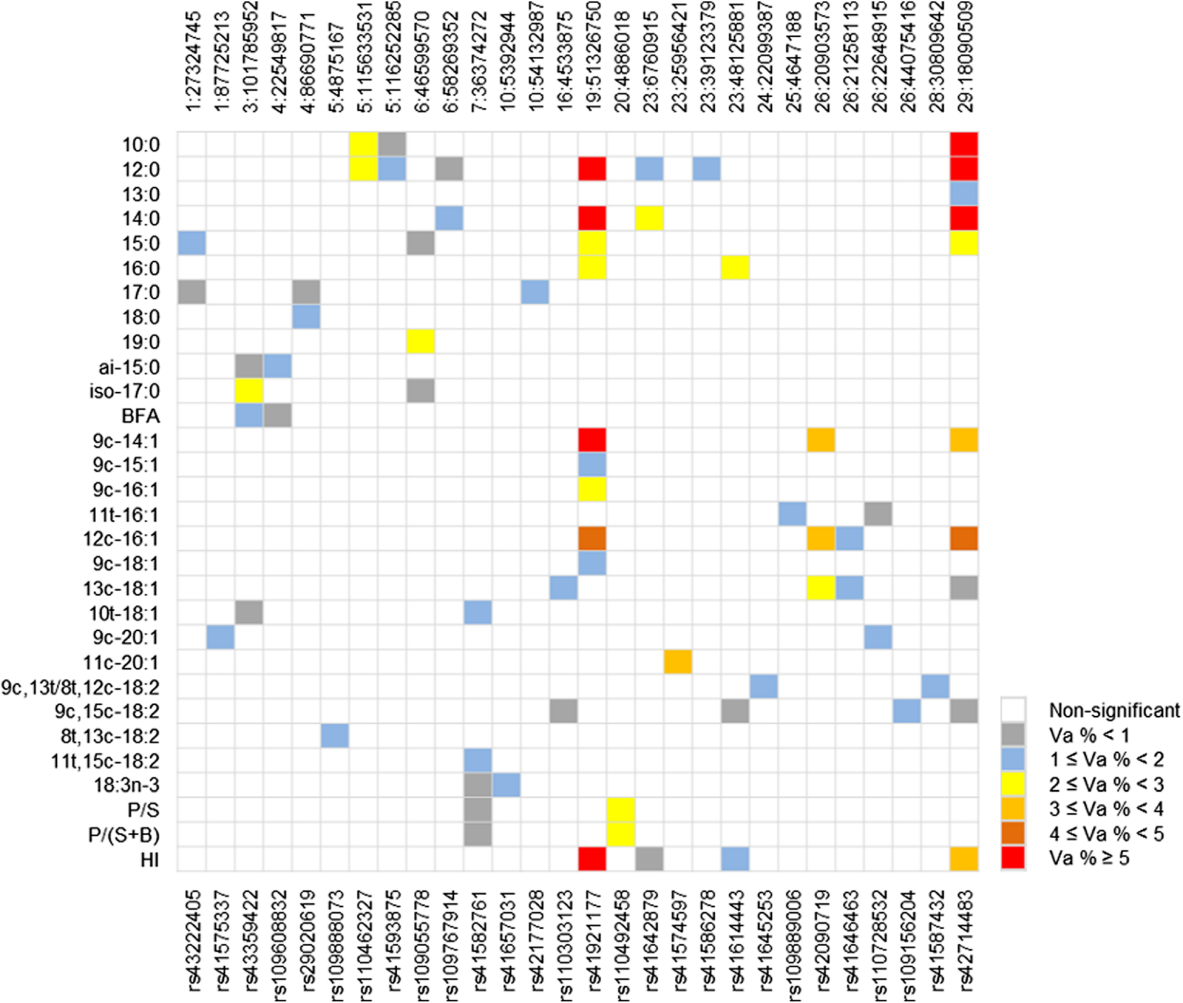
Summary of fatty acid trait associations across genomic regions (SNPs) and percentage of genetic variance explained by significant SNPs in the subcutaneous adipose tissue (SQ). Each row represents a trait and each column represents a SNP. Only traits with at least one significant SNP explaining greater than 1 % of genetic variance were listed, and only SNPs that explain greater than 1 % of genetic variance for at least one trait were shown. The top of the figure shows the chromosome and position and the bottom shows the name of the SNP. Va %: percentage of genetic variance

**Fig. 2:**
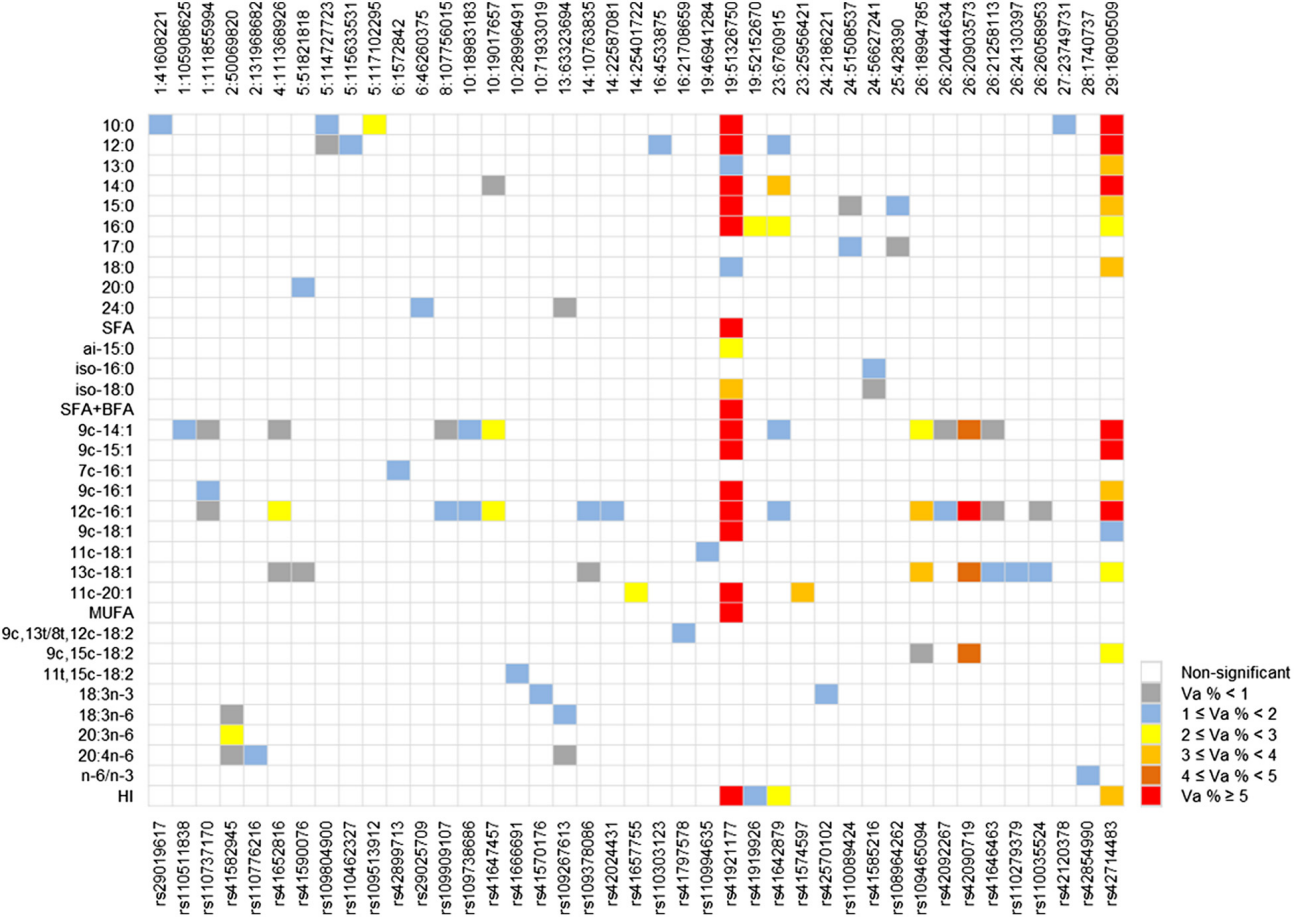
Summary of fatty acid trait associations across genomic regions (SNPs) and percentage of genetic variance explained by significant SNPs in *longissimus lumborum* muscle (LL). Each row represents a trait and each column represents a SNP. Only traits with at least one significant SNP explaining greater than 1 % of genetic variance were listed, and only SNPs that explain greater than 1 % of genetic variance for at least one trait were shown. The top of the figure shows the chromosome and position and the bottom shows the name of the SNP. Va %: percentage of genetic variance

In total, 302 and 360 significant SNPs spanning all autosomal chromosomes were identified to be associated with one or more fatty acid traits in the SQ and LL tissues, respectively, at the genome-wise empirical significance threshold at α = 0.05. Significant SNPs and their distributions over the genome varied for different fatty acid traits. Manhattan plots of posterior probability of inclusion (PPI) were provided in Additional file 1 for all fatty acid traits in the two tissues. Proportions of genotypic variance explained by individual significant SNPs ranged from 0.03 to 11.06 % in SQ, and from 0.005 to 24.28 % in LL. Among these, 28 and 41 SNPs individually explained greater than 1 % of total genetic variance for at least one fatty acid trait in the SQ and LL tissues, respectively. Figures 1 and 2 showed these SNPs and their associated traits in SQ and LL, respectively. Of these SNPs, SNP *rs41921177* at the location of BTA19:51326750 had the largest effects on multiple fatty acid traits in both tissues, followed by SNP *rs42714483* at BTA29:18090509 and SNP *rs42090719* at BTA26:20903573. Details of all significant SNPs including SNP name, chromosome position, allele substitution effect, percentage of total genetic variance explained, and PPI were also provided in additional files (Additional file 2 for SQ and Additional file 3 for LL). Candidate genes within 1 mega base pair (Mb) region centering the significant SNPs were provided separately (Additional file 4).

SNP *rs41921177* was significantly associated with 19 individual and grouped fatty acids in the LL muscle including SFAs 10:0, 12:0, 13:0, 14:0, 15:0, 16:0, 18:0, branched fatty acids (BFAs) ai 15:0 and iso 18:0, MUFAs 9c-14:1, 9c-15:1, 9c-16:1, 12c-16:1, 9c-18:1, 11c-20:1, grouped fatty acids total SFA, SFA + BFA, total MUFA and HI, with genetic variance explained from 1.37 % (18:0) to 24.28 % (14:0). The same SNP also showed significant associations with 11 of the above fatty acids in SQ including saturated fatty acids 12:0, 14:0, 15:0, 16:0, SFA, monounsaturated fatty acids 9c-14:1, 9c-15:1, 9c-16:1, 9c-18:1, 12c-16:1 and HI, explaining 0.33 % (SFA) to 11:06 % (14:0) of the genetic variance (also see Additional file 2). This chromosomal region was previously identified to be associated with 14:0, 16:0, 16:1 and 18:1 in adipose and muscle tissues of a Jersey and Limousin crossbred beef cattle [21], with 9c-18:1, and 14:0, 14:1, 16:0, 16:1 in intramuscular fat of Japanese Black cattle [7, 37], with 14:0, 16:0, 9c-14:1 and 9c-18:1 in adipose tissue of an Australian multi-breed beef population [38], with 14:0, 14:1, 16:0, 16:1, 9c-18:1, MUFA, SFA, and Atherogenic index (AI, the inverse of HI) in muscle of American Angus beef cattle [16]. The association of this chromosomal region with the fatty acid traits was therefore confirmed in both the SQ and LL tissues of a Canadian beef population of diverse breed compositions, indicating a strong host genetic effect on the fatty acid composition in beef tissues. Multiple genes are within 1 Mb region centering the SNP (see Additional file 4), with *FASN* being a strong candidate gene due to its function in fatty acid synthesis [40, 41]. Different SNPs of the *FASN* gene have also been reported to be associated with concentrations of saturated and monounsaturated fatty acids in various beef and dairy cattle populations [7, 16, 18, 22, 37, 38, 40–45].

SNP *rs42714483* showed significant associations with concentrations of 15 fatty acids in the LL tissue and 10 fatty acids in SQ including 10:0, 12:0, 13:0, 14:0, 15:0, 9c-14:1, 12c-16:1, 13c-18:1, 9c,15c-18:2, and HI in both the tissues, and 16:0, 18:0, 9c-15:1, 9c-16:1, and 9c-18:1 in the LL tissue. Saatchi *et al.* [16] also identified the same chromosomal region associated with fatty acids 14:0, 9c-14:1, 16:0, 16:1, 18:0, 9c-18:1, and AI, and Kelly *et al.* [38] found SNPs in the same chromosomal region that were associated with fatty acids 14:0, 9c-14:1 in subcutaneous adipose tissue of an Australian multi-breed beef population [38]. These results strongly support that the chromosome region on BTA 29 harbors host genes that influence fatty acid composition of beef tissues. In this study, the SNP at BTA29:18090509 is a missense mutation (*T/C*) of the thyroid hormone responsive gene (*THRSP*), causing amino acid change from isoleucine to valine (I16V). Recently *THRSP* has been considered as a candidate gene for fatty acid composition in beef [16, 46]. Substitution of allele *T* with *C* of this missense mutation was associated with decrease of 10:0, 12:0, 13:0, 14:0, 15:0, 16:0, 9c-14:1, 9c-15:1, 9c-16:1, 12c-16:1, 13c-18:1, 9c,15c-18:2, and increase of 18:0, 9c-18:1, and HI (see Additional files 2 and 3). The direction of the allele substitution effect on different fatty acid traits also coincided with that of SNP *rs41921177*, which is close to *FASN* gene, suggesting possible co-ordinations between *THRSP* and *FASN* genes in fatty acid synthesis.

SNP *rs42090719* at BTA26: 20903573 was found to be significantly associated with 9c-14:1, 12c-16:1, 13c-18:1 in both the LL and SQ tissues, 9c,15c-18:2 and CLA isomers 11 t,13c + 11c,13 t-18:2 (also see Additional file 3) in the LL tissue. In addition, SNP *rs41646463* at BTA26:21258113 also showed significant associations with 13c-18:1 in both the LL and SQ tissues. In the nearby chromosomal region of BTA26:18994785, SNP *rs109465094* was significantly associated with 9c-14:1, 12c-16:1, 13c-18:1 and 9c,15c-18:2 in LL. The chromosomal regions on BTA 26 were previously found associated with a variety of fatty acids in muscle of American Angus and in adipose tissue in an Australian multi-breed beef population, and stearoyl-CoA desaturase gene (*SCD*) was suggested as a candidate gene [16, 38]. The two SNPs, *rs42090719* and *rs41646463,* are within 250 kilo base pairs (Kb) of *SCD*. The other SNP *rs109465094* is more than 2 Mb distant from *SCD*, indicating a possible alternative candidate gene or its association could merely be due to LD with *SCD*. Linkage disequilibrium between SNPs around the SCD gene were analysed and visualised using the Haploview software [47] and results are shown in Additional file 5. Indeed, the three significant SNPs are in moderate to high LD with SNPs in a LD block containing the SCD gene. The *SCD* gene is involved in the synthesis of particular MUFA and CLA isomers, in creating a double bond at the Δ^9^ position of fatty-acyl CoA [48, 49]. *SCD* has been reported to be associated with both meat and milk fatty acid composition in cattle [7, 16, 18, 32, 34, 37, 38, 42, 50–56]. The present study showed that SNPs close to the *SCD* gene were associated with many MUFAs and several CLA isomers but none of the SFAs, which supports the proposed role of *SCD* in fatty acid composition in beef. However, in this study none of the SNPs around *SCD* were associated with oleic acid, 9c-18:1, the most abundant MUFA in beef. This could be partly due to lack of SNPs in the current panel that are in a high LD with *SCD* to capture all its effects. Interestingly, several other studies also showed no associations between *SCD* and oleic acid in various beef and dairy cattle populations, using different SNP panels or *SCD* gene SNP [7, 16, 38, 50, 51, 57], although two other studies have reported significant associations between *SCD* SNP variants and oleic acid concentrations in Japanese Black cattle [18, 32]. The role of *SCD* on the concentration of oleic acid in beef is worthy of further investigation.

Other SNPs on BTA 1, 3, 4, 5, 6, 7, 10, 16, 20, 23, 24, 25, 28 and on 1, 2, 4, 5, 6, 8, 10, 13, 14, 16, 23, 24, 25, 27, 28 were found significantly associated with one or more fatty acid concentrations in the SQ and LL, respectively, but with relatively smaller effects (Figs. 1 and 2). The SNP *rs41642879* at BTA23:6760915 was associated with 12:0, 14:0, and HI of both the LL and SQ, and with 16:0, 9c-14:1 in LL tissue. There are several genes within the 1 Mb window centering the SNP. One possible candidate gene is the glutamate-cysteine ligase catalytic subunit gene (*GCLC*), which is involved in the synthesis of glutathione (GSH) [58]. Glutathione has an antioxidant function by oxidizing itself into Glutathione disulfide (GSSG), which in turn is reduced to GSH at the expense of nicotinamide adenine dinucleotide phosphate (NADPH) oxidase [58]. The latter is essential for fatty acid synthesis [40]. The SNP *rs41574597* at BTA23:25956421 was found to be associated with 11c-20:1 in both tissues. Several genes belonging to the butyrophilin family are located nearby. Butyrophilin is the major protein associated with milk fat droplets and has been reported to be related to milk quality in cattle [59]. However, it was suggested that butyrophilin is specific to mammary tissue [60] hence its role in meat fatty acid production remains unclear.

Fewer SNPs were identified for PUFAs (90 in SQ and 87 in LL) in comparison to the number of significant SNPs for SFAs (121 in SQ and 117 in LL) and MUFAs (174 in SQ and 120 in LL). One SNP *rs41582945* at BTA2:50069820 explained 2.45 % of genetic variance for dihomo-gamma-linolenic acid (Dihomo-GLA, 20:3n-6) in LL. However, no known genes exist in the 1 Mb region of this SNP. Several SNPs were also found to be associated with the intermediate product of Dihomo-GLA, arachidomic acid in LL (20:4n-6). The most significant SNP *rs110776216* on BTA2:131968682 explained 1.91 % of total genetic variance of 20:4n-6 and was located within the endothelin converting enzyme 1 gene (*ECE1*) which encodes the enzyme that converts big endothelin-1 to endothelin-1. Endothelin-1 was previously found to stimulate arachidonic acid release in human pericardial smooth muscle cells [61, 62]. In this study, no significant SNPs were found to be associated with iso14:0, 7c-17:1, 15 t-18:1, 9c-20:1, 6 t,8 t-18:2, 7 t,9c-18:2, 9 t,11 t-18:2, 8 t,10 t-18:2, 7 t,9 t-18:2, 20:5n3 in LL and 22:0, 7c-17:1, 11c-18:1, 12 t-18:1, 15 t-18:1, 6 t,8 t-18:2, 10 t,12 t-18:2, 9 t,11 t-18:2, 8 t,10 t-18:2, 7 t,9 t-18:2, 18:3n6, 20:3n9, and n-6/n-3 in SQ. These fatty acid traits had very low or near zero heritability estimates (Table 1), therefore their concentrations were less likely influenced by host direct genetic effects.

### Genomic prediction

Realized accuracies of genomic prediction measured as the Pearson’s correlation coefficients between genomic estimated breeding values (GEBV) and adjusted phenotypic values of fatty acid traits divided by square root of heritability are presented in Table 2. Accuracies of breeding values estimated from the pedigree-based BLUP method (PBLUP) are also presented in Table 2 as comparisons. The realized accuracy of genomic prediction ranged from −0.05 for 15 t-18:1 to 0.73 for 14:0 in the LL muscle, and varied from −0.05 for 20:3n-9 to 0.65 for 16 t-18:1 in the SQ tissue. Averaged across all traits, accuracies from PBLUP, GBLUP, and the Bayesian method were 0.23, 0.32, and 0.35, respectively, in SQ, and 0.17, 0.39, and 0.46, respectively, in LL. These results suggested the effectiveness of genomic prediction using either GBLUP or the Bayesian method. However, the incompleteness of the pedigree (only one generation) may largely contribute to the low accuracy for the PBLUP method. It should be noted that the realized accuracy could be overestimated when heritability is underestimated as pointed out by Lourenco *et al.* [63]. Accuracies that were substantially overestimated tended to have relatively large SE (>0.10) as shown in Table 2. Additionally, Pearson’s correlation coefficient between estimated breeding values and adjusted phenotypes, and regression coefficient by regressing adjusted phenotypes on estimated breeding values were also calculated and provided in Additional file 6. The correlation coefficients averaged 0.11, 0.15, and 0.15 for PBLUP, GBLUP, and the Bayesian method, respectively, in SQ, and averaged 0.08, 0.14, and 0.16, respectively in LL. The average regression coefficients in SQ were 1.02, 0.77, and 0.92, and were 1.04, 0.90, and 0.83 in LL for PBLUP, GBLUP, and the Bayesian method, respectively. The regression coefficient is expected to be 1 if the estimated breeding values were unbiased predictions of the true breeding values. Nevertheless, for most of the fatty acid traits, the accuracy of genomic prediction were relatively low (<0.40), which was expected given the low heritability estimates and the small sample size used in this study [64]. Relatively higher accuracy (*r*_(*GEBV*,*y*)_/*h* ≥ 0.50 with SE < 0.10) were achieved for 10:0 (0.53), 12:0 (0.53), 14:0 (0.73), 15:0 (0.69), 16:0 (0.50), 9c-14:1 (0.55), 12c-16:1 (0.55), 13c-18:1 (0.51), and HI (0.59) in LL, and for 12:0 (0.58), 14:0 (0.61), 15:0 (0.62), 10 t,12c-18:2 (0.52), and 11 t,13c + 11c,13 t-18:2 (0.56) in SQ. The relatively higher accuracy for certain saturated and monounsaturated fatty acids, and HI, and relatively lower accuracy for CLAs and other PUFAs in muscle were compatible with the magnitude of their estimated heritability (Table 1). The correlations between heritability estimates and realised accuracy of genomic prediction in LL were 0.61 and 0.39 for Bayesian and GBLUP methods, respectively. However, in SQ such correlations were only 0.10 for GBLUP and 0.23 for the Bayesian method, which is likely due to many overestimations of realised accuracy for traits with low and inaccurate heritability estimates. Genomic prediction from the Bayesian method performed similarly as GBLUP for most of the traits, but substantially better for several traits in LL muscle such as 10:0 (0.37 for GBLUP vs 0.53 for BayesCπ), 12:0 (0.31 vs 0.53), 14:0 (0.45 vs 0.73), 15:0 (0.57 vs 0.69), 16:0 (0.36 vs 0.50), 9c-14:1 (0.34 vs 0.55), 9c-16:1 (0.37 vs 0.49), 12c-16:1 (0.32 vs 0.55), 9c-18:1 (0.27 vs 0.37), 13c-18:1 (0.36 vs 0.51), and HI (0.41 vs 0.59), and for traits in SQ including 12:0 (0.42 vs 0.58), 14:0 (0.39 vs 0.61), and 9c-14:1 (0.31 vs 0.43). These traits have been shown to have SNPs with larger effects from GWAS results (Figs. 1 and 2). The Bayesian method adopted in this study allows a fraction of SNPs to take relatively large effects, which may better characterize the genetic architecture of traits that have QTL of larger effects than the GBLUP method [65], which assumes all SNPs have the same genetic variance.

**Table 2:** Realised accuracy (±SE) of breeding value prediction for fatty acid traits in the subcutaneous adipose and *longissimus lumborum *musle

	Subcutaneous adipose			*Longissimus lumborum*		
Trait^a^	PBLUP	GBLUP	BayesCπ	PBLUP	GBLUP	BayesCπ
10:0	0.32 ± 0.05	0.37 ± 0.05	0.45 ± 0.05	0.27 ± 0.05	0.37 ± 0.07	0.53 ± 0.06
12:0	0.26 ± 0.04	0.42 ± 0.03	0.58 ± 0.02	0.23 ± 0.03	0.31 ± 0.06	0.53 ± 0.04
13:0	0.06 ± 0.06	0.35 ± 0.04	0.34 ± 0.04	−0.05 ± 0.06	0.31 ± 0.07	0.32 ± 0.07
14:0	0.34 ± 0.04	0.39 ± 0.05	0.61 ± 0.04	0.27 ± 0.04	0.45 ± 0.06	0.73 ± 0.04
15:0	0.38 ± 0.06	0.55 ± 0.06	0.62 ± 0.06	0.26 ± 0.06	0.57 ± 0.08	0.69 ± 0.08
16:0	0.34 ± 0.05	0.28 ± 0.05	0.31 ± 0.05	0.23 ± 0.03	0.36 ± 0.05	0.50 ± 0.05
17:0	0.30 ± 0.05	0.33 ± 0.05	0.34 ± 0.05	0.29 ± 0.05	0.40 ± 0.04	0.46 ± 0.04
18:0	0.15 ± 0.05	0.29 ± 0.06	0.29 ± 0.07	0.24 ± 0.05	0.35 ± 0.05	0.38 ± 0.04
19:0	0.33 ± 0.10	0.40 ± 0.09	0.40 ± 0.10	0.18 ± 0.11	0.18 ± 0.13	0.18 ± 0.13
20:0	0.05 ± 0.06	0.22 ± 0.08	0.21 ± 0.08	0.25 ± 0.05	0.24 ± 0.05	0.24 ± 0.05
22:0	0.24 ± 0.07	0.36 ± 0.05	0.38 ± 0.05	0.17 ± 0.06	0.44 ± 0.07	0.43 ± 0.08
24:0	-	-	-	0.23 ± 0.06	0.27 ± 0.06	0.28 ± 0.06
SFA	0.29 ± 0.06	0.28 ± 0.07	0.29 ± 0.07	0.14 ± 0.04	0.37 ± 0.06	0.43 ± 0.06
iso14:0	0.16 ± 0.10	0.25 ± 0.10	0.24 ± 0.09	0.30 ± 0.05	0.18 ± 0.08	0.18 ± 0.08
iso15:0	0.20 ± 0.05	0.24 ± 0.07	0.25 ± 0.07	0.17 ± 0.03	0.20 ± 0.07	0.18 ± 0.07
ai15:0	0.21 ± 0.03	0.30 ± 0.04	0.31 ± 0.04	0.18 ± 0.04	0.37 ± 0.05	0.39 ± 0.05
iso16:0	0.20 ± 0.07	0.27 ± 0.06	0.28 ± 0.05	0.15 ± 0.04	0.28 ± 0.06	0.29 ± 0.05
iso17:0	0.18 ± 0.06	0.27 ± 0.05	0.27 ± 0.05	0.25 ± 0.07	0.29 ± 0.06	0.29 ± 0.06
ai17:0	0.14 ± 0.08	0.33 ± 0.08	0.31 ± 0.08	0.15 ± 0.04	0.34 ± 0.04	0.34 ± 0.05
iso18:0	0.12 ± 0.05	0.22 ± 0.05	0.22 ± 0.05	0.21 ± 0.05	0.33 ± 0.06	0.37 ± 0.06
BFA	0.20 ± 0.06	0.31 ± 0.05	0.31 ± 0.05	0.17 ± 0.05	0.32 ± 0.04	0.32 ± 0.04
SFA + BFA	0.30 ± 0.06	0.29 ± 0.07	0.31 ± 0.07	0.14 ± 0.04	0.36 ± 0.06	0.42 ± 0.06
9c-14:1	0.19 ± 0.06	0.31 ± 0.04	0.43 ± 0.04	0.27 ± 0.06	0.34 ± 0.04	0.55 ± 0.03
9c-15:1	0.16 ± 0.06	0.23 ± 0.07	0.23 ± 0.08	0.24 ± 0.11	0.36 ± 0.10	0.37 ± 0.10
7c-16:1	0.19 ± 0.05	0.18 ± 0.07	0.20 ± 0.07	0.16 ± 0.07	0.22 ± 0.05	0.24 ± 0.05
9c-16:1	0.13 ± 0.04	0.25 ± 0.06	0.26 ± 0.07	0.29 ± 0.03	0.37 ± 0.03	0.49 ± 0.02
11 t-16:1	0.14 ± 0.08	0.33 ± 0.05	0.33 ± 0.05	0.08 ± 0.08	0.12 ± 0.09	0.13 ± 0.10
12c-16:1	0.22 ± 0.05	0.34 ± 0.03	0.43 ± 0.03	0.25 ± 0.05	0.32 ± 0.04	0.55 ± 0.02
7c-17:1	-	-	-	−0.02 ± 0.15	0.33 ± 0.24	0.32 ± 0.21
9c-17:1	0.34 ± 0.07	0.48 ± 0.06	0.48 ± 0.05	0.37 ± 0.08	0.40 ± 0.08	0.40 ± 0.08
9c-18:1	0.35 ± 0.10	0.30 ± 0.10	0.29 ± 0.10	0.14 ± 0.03	0.27 ± 0.06	0.37 ± 0.05
11c-18:1	0.57 ± 0.18	0.57 ± 0.18	0.55 ± 0.18	0.29 ± 0.02	0.44 ± 0.07	0.45 ± 0.07
12c-18:1	0.00 ± 0.09	0.11 ± 0.10	0.11 ± 0.09	0.11 ± 0.10	0.18 ± 0.10	0.19 ± 0.10
13c-18:1	0.19 ± 0.05	0.36 ± 0.04	0.41 ± 0.04	0.21 ± 0.03	0.36 ± 0.05	0.51 ± 0.04
14c-18:1	0.10 ± 0.09	0.28 ± 0.11	0.28 ± 0.11	0.15 ± 0.09	0.17 ± 0.07	0.17 ± 0.07
15c-18:1	0.20 ± 0.04	0.30 ± 0.04	0.31 ± 0.04	0.19 ± 0.05	0.26 ± 0.05	0.25 ± 0.06
6 t + 8 t-18:1	0.30 ± 0.06	0.35 ± 0.04	0.34 ± 0.04	0.20 ± 0.07	0.29 ± 0.04	0.30 ± 0.04
9 t-18:1	0.30 ± 0.09	0.33 ± 0.06	0.33 ± 0.06	0.26 ± 0.08	0.31 ± 0.04	0.32 ± 0.04
10 t-18:1	0.28 ± 0.03	0.42 ± 0.03	0.43 ± 0.03	0.26 ± 0.03	0.41 ± 0.03	0.42 ± 0.03
11 t-18:1	0.35 ± 0.08	0.43 ± 0.09	0.42 ± 0.09	0.29 ± 0.04	0.38 ± 0.04	0.35 ± 0.05
12 t-18:1	-	-	-	0.05 ± 0.07	0.27 ± 0.10	0.26 ± 0.10
15 t-18:1	-	-	-	−0.26 ± 0.15	−0.05 ± 0.16	−0.01 ± 0.16
16 t-18:1	0.41 ± 0.07	0.65 ± 0.13	0.64 ± 0.13	0.33 ± 0.05	0.47 ± 0.07	0.48 ± 0.07
sumtrans18:1	0.22 ± 0.03	0.35 ± 0.04	0.35 ± 0.03	0.25 ± 0.02	0.36 ± 0.03	0.37 ± 0.03
9c-20:1	0.33 ± 0.06	0.45 ± 0.07	0.45 ± 0.07	−0.03 ± 0.10	0.02 ± 0.09	0.00 ± 0.09
11c-20:1	0.20 ± 0.05	0.27 ± 0.04	0.30 ± 0.04	0.35 ± 0.06	0.37 ± 0.05	0.47 ± 0.04
MUFA	0.29 ± 0.07	0.29 ± 0.08	0.30 ± 0.08	0.11 ± 0.03	0.25 ± 0.07	0.31 ± 0.06
9c,13 t + 8 t,12c-18:2	0.22 ± 0.05	0.34 ± 0.07	0.33 ± 0.07	0.14 ± 0.06	0.36 ± 0.08	0.36 ± 0.08
9c,15c-18:2	0.18 ± 0.07	0.33 ± 0.05	0.35 ± 0.05	0.22 ± 0.04	0.38 ± 0.06	0.45 ± 0.06
8 t,13c-18:2	0.23 ± 0.05	0.38 ± 0.06	0.37 ± 0.06	0.02 ± 0.10	0.30 ± 0.04	0.31 ± 0.04
11 t,15c-18:2	0.25 ± 0.05	0.31 ± 0.06	0.32 ± 0.06	0.25 ± 0.03	0.34 ± 0.04	0.36 ± 0.04
9c,11 t + 9 t,11c-18:2	0.05 ± 0.08	0.25 ± 0.07	0.24 ± 0.07	0.26 ± 0.05	0.26 ± 0.07	0.25 ± 0.06
7 t,9c-18:2	0.58 ± 0.14	0.63 ± 0.12	0.64 ± 0.13	0.43 ± 0.22	0.31 ± 0.25	0.28 ± 0.26
12 t,14 t-18:2	0.06 ± 0.28	0.44 ± 0.33	0.41 ± 0.34	0.25 ± 0.19	0.50 ± 0.23	0.48 ± 0.22
11 t,13 t-18:2	0.48 ± 0.16	0.47 ± 0.11	0.46 ± 0.12	0.11 ± 0.10	0.06 ± 0.09	0.06 ± 0.09
10 t,12 t-18:2	-	-	-	0.09 ± 0.08	0.08 ± 0.11	0.08 ± 0.11
9 t,11 t-18:2	-	-	-	0.11 ± 0.10	0.01 ± 0.08	0.02 ± 0.08
8 t,10 t-18:2	0.35 ± 0.14	0.28 ± 0.14	0.28 ± 0.13	0.24 ± 0.13	−0.01 ± 0.10	0.05 ± 0.11
7 t,9 t-18:2	0.41 ± 0.44	0.10 ± 0.47	0.04 ± 0.48	-	-	-
12 t,14c + 12c,14 t −18:2	−0.01 ± 0.26	0.32 ± 0.25	0.34 ± 0.24	0.06 ± 0.09	0.04 ± 0.07	0.04 ± 0.07
11 t,13c + 11c,13 t −18:2	0.54 ± 0.06	0.56 ± 0.08	0.54 ± 0.08	0.16 ± 0.10	0.29 ± 0.06	0.30 ± 0.06
10 t,12c-18:2	0.44 ± 0.09	0.52 ± 0.07	0.52 ± 0.08	0.04 ± 0.12	0.21 ± 0.14	0.22 ± 0.14
8 t,10c-18:2	0.33 ± 0.17	0.32 ± 0.14	0.31 ± 0.14	0.27 ± 0.08	0.15 ± 0.07	0.13 ± 0.07
Total CLA	0.25 ± 0.09	0.37 ± 0.09	0.36 ± 0.10	0.12 ± 0.06	0.06 ± 0.06	0.06 ± 0.06
18:2n-6	0.21 ± 0.02	0.35 ± 0.04	0.36 ± 0.04	0.15 ± 0.05	0.29 ± 0.04	0.32 ± 0.04
18:3n-3	0.27 ± 0.04	0.38 ± 0.04	0.38 ± 0.04	0.28 ± 0.06	0.33 ± 0.04	0.34 ± 0.04
18:3n-6	0.10 ± 0.11	0.09 ± 0.09	0.11 ± 0.09	0.20 ± 0.05	0.36 ± 0.05	0.41 ± 0.04
20:2n-6	0.20 ± 0.07	0.06 ± 0.05	0.06 ± 0.06	0.07 ± 0.11	0.10 ± 0.07	0.11 ± 0.07
20:3n-6	0.10 ± 0.11	0.24 ± 0.11	0.22 ± 0.11	0.14 ± 0.03	0.32 ± 0.04	0.34 ± 0.05
20:3n-9	0.20 ± 0.16	−0.03 ± 0.11	−0.05 ± 0.13	0.12 ± 0.04	0.33 ± 0.07	0.33 ± 0.07
20:4n-6	−0.10 ± 0.04	0.10 ± 0.09	0.09 ± 0.08	0.09 ± 0.03	0.29 ± 0.06	0.31 ± 0.05
20:5n-3	-	-	-	0.32 ± 0.06	0.10 ± 0.11	0.09 ± 0.11
22:4n-6	0.10 ± 0.04	0.19 ± 0.08	0.19 ± 0.08	0.15 ± 0.06	0.38 ± 0.08	0.40 ± 0.08
22:5n-3	0.04 ± 0.10	0.14 ± 0.08	0.14 ± 0.08	0.15 ± 0.04	0.39 ± 0.07	0.39 ± 0.07
22:6n-3	-	-	-	0.00 ± 0.05	0.10 ± 0.08	0.10 ± 0.07
PUFA	0.21 ± 0.02	0.35 ± 0.04	0.36 ± 0.04	0.14 ± 0.04	0.30 ± 0.04	0.33 ± 0.04
n-3	0.27 ± 0.04	0.37 ± 0.05	0.38 ± 0.04	0.22 ± 0.04	0.40 ± 0.05	0.40 ± 0.05
n-6	0.20 ± 0.02	0.35 ± 0.04	0.36 ± 0.04	0.14 ± 0.04	0.29 ± 0.04	0.32 ± 0.04
n-6/n-3	-	-	-	0.11 ± 0.04	0.16 ± 0.04	0.14 ± 0.04
P/S	0.19 ± 0.03	0.33 ± 0.04	0.34 ± 0.04	0.14 ± 0.03	0.38 ± 0.04	0.39 ± 0.03
P/(S + B)	0.19 ± 0.03	0.33 ± 0.04	0.34 ± 0.04	0.14 ± 0.09	0.38 ± 0.04	0.39 ± 0.04
HI	0.31 ± 0.05	0.30 ± 0.05	0.38 ± 0.05	0.22 ± 0.05	0.41 ± 0.06	0.59 ± 0.06

^a^The concentrations of fatty acids were expressed as a percentage of fatty acid methyl esters (FAME) quantified. c = cis, t = trans. SFA = 10:0 + 12:0 + 13:0 + 14:0 + 15:0 + 16:0 + 17:0 + 18:0 + 19:0 + 20:0 + 22:0 + 24:0; BFA = iso14:0 + iso15:0 + ai15:0 + iso16:0 + iso17:0 + ai17:0 + iso18:0; SFA + BFA: sum of SFA and BFA; sumtrans18:1 = 6 t/8 t-18:1 + 9 t-18:1 + 10 t-18:1 + 11 t-18:1 + 12 t-18:1 + 15 t-18:1 + 16 t-18:1; MUFA = 9c-14:1 + 9c-15:1 + 7c-16:1 + 9c-16:1 + 11 t-16:1 + 12c-16:1 + 7c-17:1 + 9c-17:1 + 9c-18:1 + 11c-18:1 + 12c-18:1 + 13c-18:1 + 14c-18:1 + 15c-18:1 + 9c-20:1 + 11c-20 + 6 t/8 t-18:1 + 9 t-18:1 + 10 t-18:1 + 11 t-18:1 + 12 t-18:1 + 15 t-18:1 + 16 t-18:1; Total CLA = 9c,11 t + 9 t,11c-18:2 + 6 t,8 t-18:2 + 7 t,9c-18:2 + 12 t,(14 t-18:2 + 11 t,13 t)-18:2 + 10 t,12 t-18:2 + 9 t,11 t-18:2 + 8 t,10 t-18:2 + 7 t,9 t-18:2 + (12 t,14c + 12c,14 t) -18:2 + (11 t,13c + 11c,13 t)-18:2 + 10 t,12c-18:2 + 8 t,10c-18:2; PUFA = 18:2n-6 + 18:3n-6 + 18:3n-3 + 20:2n-6 + 20:3n-9 + 20:3n-6 + 20:4n-6 + 22:4n-6 + 22:5n-3+ 22:6n-3; n-3 = 18:3n-3 + 20:5n-3 + 22:5n-3 + 22:6n-3; n-6 = 18:2n-6 + 18:3n-6 + 20:2n-6 + 20:3n-6 + 20:4n-6 + 22:4n-6; n-6/n-3: ratio between n-6 and n-3; P/S = PUFA/SFA; P/(S + B) = PUFA/(SFA + BFA); HI = (MUFA + PUFA) / (4 × 14:0 + 16:0). ‘-’ = not calculated due to a zero heritability

Fatty acid composition is a complex trait and it is difficult and expensive to measure, making it a good candidate trait for genomic selection. To date, genomic prediction for fatty acid composition in beef cattle has only been reported by Saatchi *et al.* [16] for 24 individual and grouped/ratio of fatty acids in steaks of American Angus beef cattle, and by Onogi *et al.* [39] for 8 fatty acid traits in *musculus trapezius *of Japanese Black cattle. Relatively higher prediction accuracies were found for 14:0 (0.57), 16:0 (0.53), total long chain saturated fatty acids (0.57), total medium chain saturated fatty acids (0.57), 9c-18:1 (0.35), 12c-18:1 (0.35), total MUFA (0.38), (14:0 + 16:0)/all (0.55), and AI (0.56) in Saatchi’s study, compared to other fatty acid traits. In this study, relatively higher accuracies were also obtained for SFAs 12:0, 14:0, and 15:0 in both the LL and SQ tissues, and for 10:0, 16:0, 9c-14:1, 12c-16:1, 13c-18:1, and HI in LL (Table 2), suggesting strong host genetic controls on synthesis of these SFAs and MUFAs. Saatchi *et al.* [16] reported genomic prediction accuracies for 12 PUFAs and all were very low (<0.30). In this study, we analyzed 32 and 34 PUFAs and PUFA-BHI (including CLAs and 11 t-18:1) in the adipose and muscle tissues, respectively, and found moderate accuracies (between 0.30 and 0.45) for 11 t-18:1, 9c,13 t + 8 t,12c-18:2, 9c,15c-18:2, 8 t,13c-18:2, 11 t,15c-18:2, 18:2n-6, 18:3n-3, n-3, n-6, and total PUFA in both the adipose and muscle tissues, moderate accuracies for 20:3n-6, 20:3n-9, 22:4n-6, 22:6n-3 in the muscle, and relatively high accuracies (>0.50) for 12 t,14c + 12c,14 t-18:2, and 11 t,13c + 11c,13 t-18:2 in the adipose tissue, suggesting considerable host genetic influence on these fatty acids. Different beef cattle populations, environments where the animals were raised, sample sizes and statistical models may also contribute to the differences of genomic prediction accuracy observed between different studies. Although most dietary PUFAs are biohydrogenated by rumen bacteria [66], a portion of PUFAs and PUFA-BHI may escape and deposit into body fat of beef. In addition, some PUFAs can be endogenously synthesized, for example CLAs can be synthesized from one of the PUFA-BHI, vaccenic acid (11 t-18:1) by the host [67]. Therefore, contents of both PUFAs and PUFA-BHI are potentially influenced by host genetics and thus predictable by genomic prediction. Onogi *et al*. [39] also reported a relatively high accuracy (0.56) for PUFA C18:2 in Japanese Black cattle. Although it would be worthwhile to further verify the genomic prediction accuracy in other beef cattle populations, the moderate to relatively high genomic prediction accuracies achieved in this study for the HI, several individual SFAs, MUFAs, PUFAs and PUFA-BHI suggest that genomic selection is a promising tool for genetic improvement of fatty acid profiles in beef cattle to produce healthier meat. Therefore, as consumers’ demand for healthier meat continues to grow, beef producers may get more premiums by producing meat with enhanced fatty acid profiles, which can be achieved by incorporating fatty acid composition traits into a multi-trait selection index for selection and/or by genetic based diet management.

## Conclusions

Fatty acid composition in beef tissues is a polygenic trait that is controlled by a few major host genes and many genes of small effects. Several genes, including *FASN*, *SCD*, and *THRSP*, are major candidate genes for variations of fatty acid contents in beef cattle. Accuracy of genomic prediction was low for most of the fatty acid traits investigated. Moderate accuracy was obtained for SFAs 10:0, 12:0, 13:0, 14:0, 15:0, 16:0, MUFAs 9c-14:1, 12c-16:1, 13c-18:1, and HI in LL, and for SFAs 12:0, 14:0, 15:0, and CLA isomers 10 t,12c-18:2, and 11 t,13c + 11c,13 t-18:2 in SQ. The Bayesian method performed similarly as GBLUP for most of the traits, but substantially better for fatty acid traits that are influenced by QTL of larger effects. The moderate genomic prediction accuracy achieved in this study for HI in LL and several individual fatty acids in LL and SQ tissue suggest that it is possible to genetically improve fatty acid profiles in beef cattle to produce healthier meat through genomic selection. Further investigations on the identification of causal mutations for variations of fatty acid contents in beef tissues and on improvement of genomic prediction accuracy are required.

## Methods

### Animal populations, tissue collection and fatty acid analyses

A total of 1366 steers and heifers born between 2008 and 2011 were used in this study. The animals were from four different herds including three commercial herds and one experimental herd located in Alberta of Canada. All dietary treatments and experimental procedures were approved by the AAFC Lacombe Research Centre Animal Care Committee and animals were cared for as outlined under the guidelines established by the Canadian Council on Animal Care [68]. Breed compositions of the 1366 steers and heifers were represented by purebred Angus (ANAN, *n* = 6), Hereford-Angus crossbreds (HEAN, *n* = 120), Charolais-Red Angus crossbreds (CHAR, *n* = 93), crossbreds produced by mating Hereford-Angus to Gelbvieh-Angus crossbreds (HEANGV, *n* = 209), and calves produced from crosses between a composite terminal bull strain which was derived from Hereford, Black Angus, Red Angus, and Limousin, and crossbred cows with a mixed background of Angus, Red Angus, Hereford, Simmental, Charolais, Limousin and Gelbvieh (TXX, *n* = 938). A more detailed description of breeding and management of the herds have been described previously [69–71]. A written consent from the owner of the commercial herds was obtained for the use of cattle data in this study. After weaning, animals were raised under one of four production systems: (1) calf-fed, growth implant; (2) calf-fed; no growth implant; (3) yearling-fed, growth implant; (4) yearling-fed, no growth implant [70, 72]. All animals were fed high concentration diets for finishing and were targeted to be slaughtered at a constant back fat thickness of 9 to 10 mm measured between the 12^th^ and 13^th^ ribs.

After slaughter, the *longissimus luborum* muscle (LL) of each animal was taken from the left striploin at 48 h post-mortem, vacuum packed and then chilled at 2 °C. The striploin samples were then transported by a refrigerated truck to a meat lab of the AAFC Lacombe Research Centre where a sub-sample of approximately 10 grams of LL muscle and 5 grams of subcutaneous adipose (SQ) tissue from the side of the striploin of each animal was taken, vacuum packed and frozen at −80 °C for subsequent fatty acid analyses. The two tissues have distinct metabolism roles involving fat usage: muscle is mainly for energy expenditure to produce force and motion while adipose including intramuscular fat within muscle is the main tissue for fat storage [73]. The two tissues were selected mainly because they are major parts of carcass that are consumed as beef products by humans. Fatty acid analyses of LL and SQ tissues were based on the protocols described previously with some modifications [10]. Briefly, lipid was extracted from the LL muscle tissue using Folch’s method [74] as outlined by Cruz-Hernandez et al. [75] and from the SQ tissue based on the procedures described in [75] and [76]. Fatty acid methyl esters (FAME) were then derivatized using sodium methoxide from the lipid extracts for quantification of fatty acid composition. Gas chromatography (GC) and silver-ion high performance liquid chromatography (Ag + HPLC) analyses were conducted to separate and quantify individual fatty acids as outlined in [77] using a two-step GC procedure and in [75] using Ag + HPLC. Individual fatty acids were expressed as a percentage of the total FAME. Concentrations of groups of fatty acids, including total saturated fatty acids (SFA), branched fatty acids (BFA), sum of SFA and BFA (SFA + BFA), mono-unsaturated fatty acids (MUFA), poly-unsaturated fatty acids (PUFA), sum of trans 18:1 fatty acids (sumtrans18:1), total conjugated linoleic acid (Total CLA), n-3, and n-6, were measured by summing up the percentages of individual fatty acids within the fatty acid group. Ratios between PUFA and SFA (P/S), PUFA and sum of SFA and BFA (P/(S + B)), and between n-6 and n-3 (n-6/n-3) were also calculated. A health index (HI), proposed in [35], was computed as HI = (MUFA + PUFA) / (4 × 14:0 + 16:0). A total of 83 individual and grouped/ratio fatty acid traits in the LL muscle and 81 in the SQ tissue were quantified. Two fatty acids, 20:5n3 and 22:6n3 were not detected in SQ in this study due to their extremely low concentrations in the tissue.

### Single nucleotide polymorphism genotyping

All animals were genotyped on the Illumina BovineSNP50 Beadchip comprised of 54,609 SNP markers. Markers with minor allele frequency less than 0.05, missing rate greater than 0.20, extremely deviated from Hardy-Weinberg equilibrium test (*P* < 10^−6^), or in high correlation with another SNP (*r* ≥ 0.95) were removed. After filtering, 35,446 SNPs were kept for analyses. Sporadically missing genotypes represented 0.14 % of the total genotypes and were imputed via Beagle 3.3.2 [78].

### Genome-wide association study

Phenotypic values were adjusted for fixed effects and random contemporary group effects using a linear mixed model which included fixed effects of breed type, gender, production system, linear covariates of animal’s age at slaughter, days between slaughter and fatty acid extraction, and metabolic energy of diet, and random effects of contemporary groups defined as combinations of feedlot location and year, additive genetic effects and residual errors. Fatty acid traits in LL muscle were also adjusted for intramuscular fat content by including the marbling score as an additional fixed linear covariate. A genomic relationship matrix for additive genetic effects was constructed from SNP marker genotypes using the first method of VanRaden [79]. Variance components and heritability were estimated using the above model and average-information REML algorithm implemented via ASReml v3.0 software package [80].

The adjusted phenotypes were subsequently analysed using the BayesCπ method [81] for genome-wide association studies. The model can be described as follows:

$$ {y}_i=\mu +{\displaystyle \sum_{j=1}^M{x}_{ij}{a}_j+{e}_i,} $$

where *y*_*i*_ is the adjusted phenotypic value of the *i*^*th*^ animal, *μ* is the general mean, *x*_*ij*_ is the *j*^*th*^ SNP genotype of animal *i* and was coded as 0, 1 or 2 depending on copies of an arbitrarily specified allele, *M* is the total number of SNP markers, *a*_*j*_ is the allele substitution effect of SNP *j*, and *e*_*i*_ is the random residual effect.

A mixture distribution was assumed for *a*_*j*_ so that (*a*_*j*_|*π*, *σ*_*a*_^2^) ~ (1 − *π*)*N*(0, *σ*_*a*_^2^) + *πδ*_0_(*a*_*j*_), where *N*(0, *σ*_*a*_^2^) is a normal distribution with mean 0 and variance *σ*_*a*_^2^, and *δ*_0_(*a*_*j*_) denotes a distribution concentrated at zero, and (1 − *π*) and *π* are the weights for the two distributions. A latent indicator variable *γ*_*j*_ was introduced for each SNP so that when *γ*_*j*_ = 1, *a*_*j*_ *~ N*(0, *σ*_*a*_^2^), and when *γ*_*j*_ = 0, *a*_*j*_ = 0. Prior distribution for *γ*_*j*_ follows a Bernoulli distribution with probability (1 − *π*), and the joint prior density for *γ* is $f\left(\gamma \Big|\pi \right) = {\prod}_j{\pi}^{\left(1-{\gamma}_j\right)}{\left(1-\pi \right)}^{\gamma_j}.$ Residual error *e*_*i*_ was assumed from a normal distribution *N*(0, *σ*_*e*_^2^). The prior distribution for *σ*_*a*_^2^ (or *σ*_*e*_^2^) is a scaled inverse Chi-square distribution with degree of freedom *v*_*a*_ (or *v*_*e*_) and a scale parameter *S*_*a*_^2^ (or *S*_*e*_^2^). The hyper-parameter *v*_a_ (or *v*_e_) was arbitrarily set to 4 (or 10), and *S*_*a*_^2^ (or *S*_*e*_^2^) was set to ${\widehat{\sigma}}_u^2\left({v}_a-2\right)/\left[{v}_a\left(1-\pi \right)\sum 2{p}_j\left(1-{p}_j\right)\right]$ (or ${\widehat{\sigma}}_0^2\left({v}_e-2\right)/{v}_e$), where* p*_*j*_ is allele frequency for marker *j*, ${\widehat{\sigma}}_u^2$ and ${\widehat{\sigma}}_0^2$ were total additive genetic and residual variances obtained from the analyses described previously. A Gibbs sampling algorithm was used for generating samples for unknown parameters from their joint posterior distribution. The computer program was self-written in C language using the computing algorithm as described by Chen *et al*. [82]. The Gibbs chain length was 45,000 with the first 5000 discarded as burn-in. Posterior inclusion probability for each SNP was estimated as sample mean of the latent indicator variable for that SNP, and was used as a signal of association. To declare the significance of a SNP effect, empirical genome-wise significance threshold at α = 0.05 was determined by 1000 permutation analyses according to the procedure of Churchill and Doerge [83]. Briefly, the adjusted phenotype values of each fatty acid were randomly shuffled and assigned back to the animals for the BayesCπ analyses while the genotype data remained intact. The process was repeated 1000 times and the largest SNP posterior inclusion probability from each permutation analysis was kept and ordered in ascending order, and the 950^th^ value was defined as the genome-wise significance threshold. Candidate genes in the window of 1 Mb centering the significant SNPs were obtained by querying the Ensemble gene database using SNP locations from the bovine UMD3.1 genome assembly via the SNP annotation tool in the NGS-SNP suite [84].

### Genomic prediction

Genomic best linear unbiased prediction (GBLUP) and BayesCπ methods were used for genomic prediction. A ten-fold cross validation was used to evaluate the accuracy of genomic prediction. The data was first split into 10 approximately equal-sized groups according to sires of the animals so that no sire families overlapped between any two groups. For each breed type, the number of animals in each cross-validation group was kept approximately the same so that each breed in the validation group was also represented in the training population. For each cross validation, nine groups were used for training and the remaining one was used as the validation population. For GBLUP, animals in the validation population were assumed with no phenotypic values, and the animals in the training and validation populations were then combined to estimate the breeding values for animals in the validation population using a linear animal model, which can be written as:

$$ {\mathbf{y}}^{\ast }=\mathbf{1}\mu +\mathbf{Z}\mathbf{a}+\mathbf{e}, $$

Where y* is the vector of adjusted fatty acid phenotypic values from animals in the training population, *μ* is the overall mean, **a** is the vector of breeding values for all animals, **e** is the vector of random residuals and **Z** is the incidence matrix relating **a** to y*. The additive genomic relationship matrix for all animals was derived from the SNP markers using the first method of VanRaden [79], and ASReml 3.0 [80] was used to estimate the breeding values. For the BayesCπ method, SNP effects were estimated based on the training population using the statistical model as described in the GWAS analyses. The GEBV for animal *i* in the validation population was predicted by summing up SNP effects over all loci as follows:${\mathrm{GEBV}}_i={\displaystyle {\sum}_{j=1}^M{x}_{ij}{a}_j}$, where *a*_*j*_ is the estimated effect for SNP *j*. For comparisons, a pedigree based BLUP method (PBLUP) was also used to estimate the breeding values, assuming no phenotypic values for validation animals. However, only one generation of the pedigree was available for construction of the additive genetic relationship matrix. Realized accuracy of genomic prediction was measured as the correlation between estimated breeding values and the adjusted phenotype in the validation groups divided by square root of heritability and was averaged across the ten cross-validations.

### Availability of supporting data

The data sets supporting the results of this article are included within the article and its additional files. The original data sets used in this study are available upon request as part of the data is not public.

## Additional files

Additional file 1:
**Manhattan plot of posterior probability of inclusion of single nucleotide polymorphisms (SNP) for 81 fatty acid composition traits in subcutaneous adipose (SQ) and 83 traits in** ***longissimus lumborum*** **muscle (LL).** Dashed line indicates the significance threshold at genome-wise empirical threshold at α = 0.05 determined from a 1000 permutation analysis. (PDF 13567 kb) Additional file 2:
**List of significant single nucleotide polymorphisms (SNP) for 81 fatty acid composition traits in subcutaneous adipose (SQ).** Results include trait name, SNP name, chromosome, position on the UMD3.1 genome assembly, alleles, allele substitution effect, percentage of total genetic variance explained, and posterior probability of inclusion of SNP. (CSV 24 kb) Additional file 3:
**List of significant single nucleotide polymorphisms (SNP) for 81 fatty acid composition traits in** ***longissimus lumborum*** **muscle (LL).** Results include trait name, SNP name, chromosome, position on the UMD3.1 genome assembly, alleles, allele substitution effect, percentage of total genetic variance explained, and posterior probability of inclusion of SNP. (CSV 27 kb) Additional file 4:
**Genes within 1 Mb region centering significant single nucleotide polymorphisms (SNP).** Candidate genes were obtained from the Ensemble database by querying the SNP locations against the bovine UMD3.1 genome assembly using the SNP annotation tool in the NGS-SNP suite. (CSV 1037 kb) Additional file 5:
**Linkage disequilibrium (LD) beween single nucleotide polymorphisms (SNP) around the SCD gene.** Linkage disequilibrium was measured as D prime. The red triangle indicates the location of the SCD gene. An LD block was shown surrounding the SCD gene. Names of the SNPs significantly associated with fatty acid composition were highlighted in green. (PDF 971 kb) Additional file 6:
**Pearson’s correlation coefficients between estimated breeding values and adjusted phenotypes and regression coefficients of adjusted phenotypes on estimated breeding values.** (DOCX 35 kb)

## Abbreviations

AAFC: Agriculture and Agri-food Canada
Ag + HPLC: silver-ion high performance liquid chromatography
AI: atherogenic index
BFA: branched fatty acid
BHI: biohydrogenation intermediates
BTA: *Bos taurus* autosome
CLA: conjugated linoleic acid
FAME: fatty acid methyl esters
GBLUP: genomic best linear unbiased prediction
GC: gas chromatography
GEBV: genomic estimated breeding value
GWAS: genome-wide association study
HI: health index
LD: linkage disequilibrium
LL: *Longissimus lumborum *muscle
MME: mixed model equations
MUFA: monounsaturated fatty acid
PBLUP: pedigree-based best linear unbiased prediction
PPI: posterior probability of inclusion
PUFA: polyunsaturated fatty acid
QTL: quantitative trait loci
SFA: saturated fatty acid
SNP: single nucleotide polymorphism
SQ: subcutaneous adipose
