# Supplementary material for: Genome-wide association and genomic prediction of breeding values for fatty acid composition in subcutaneous adipose and longissimus lumborum muscle of beef cattle
Source: BMC Genet. 2015 Nov 21;16:135. doi: 10.1186/s12863-015-0290-0 (PMC4654876; doi:10.1186/s12863-015-0290-0)
Supplement: Additional file 1: — Manhattan plot of posterior probability of inclusion of single nucleotide polymorphisms (SNP) for 81 fatty acid composition traits in subcutaneous adipose (SQ) and 83 traits in longissimus lumborum muscle (LL). Dashed line indicates the significance threshold at genome-wise empirical threshold at α = 0.05 determined from a 1000 permutation analysis. (PDF 13567 kb) [file 12863_2015_290_MOESM1_ESM.pdf]

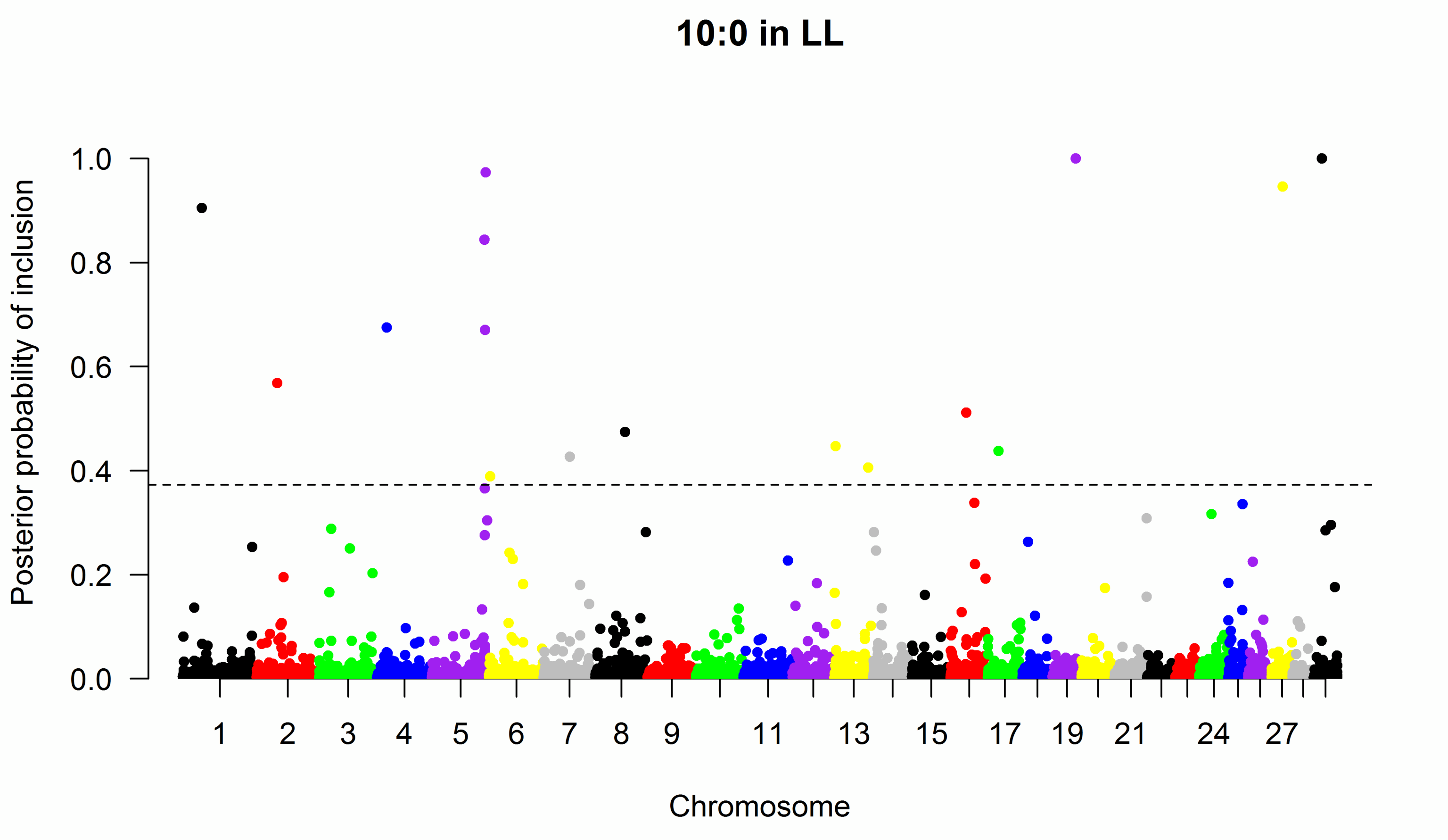

10:0 in LL
Posterior probability of inclusion
1.0
0.8
0.6
0.4
0.2
0.0
1
2
3
4
5
6
7
8
9
11
13
15
17
19
21
24
27
Chromosome

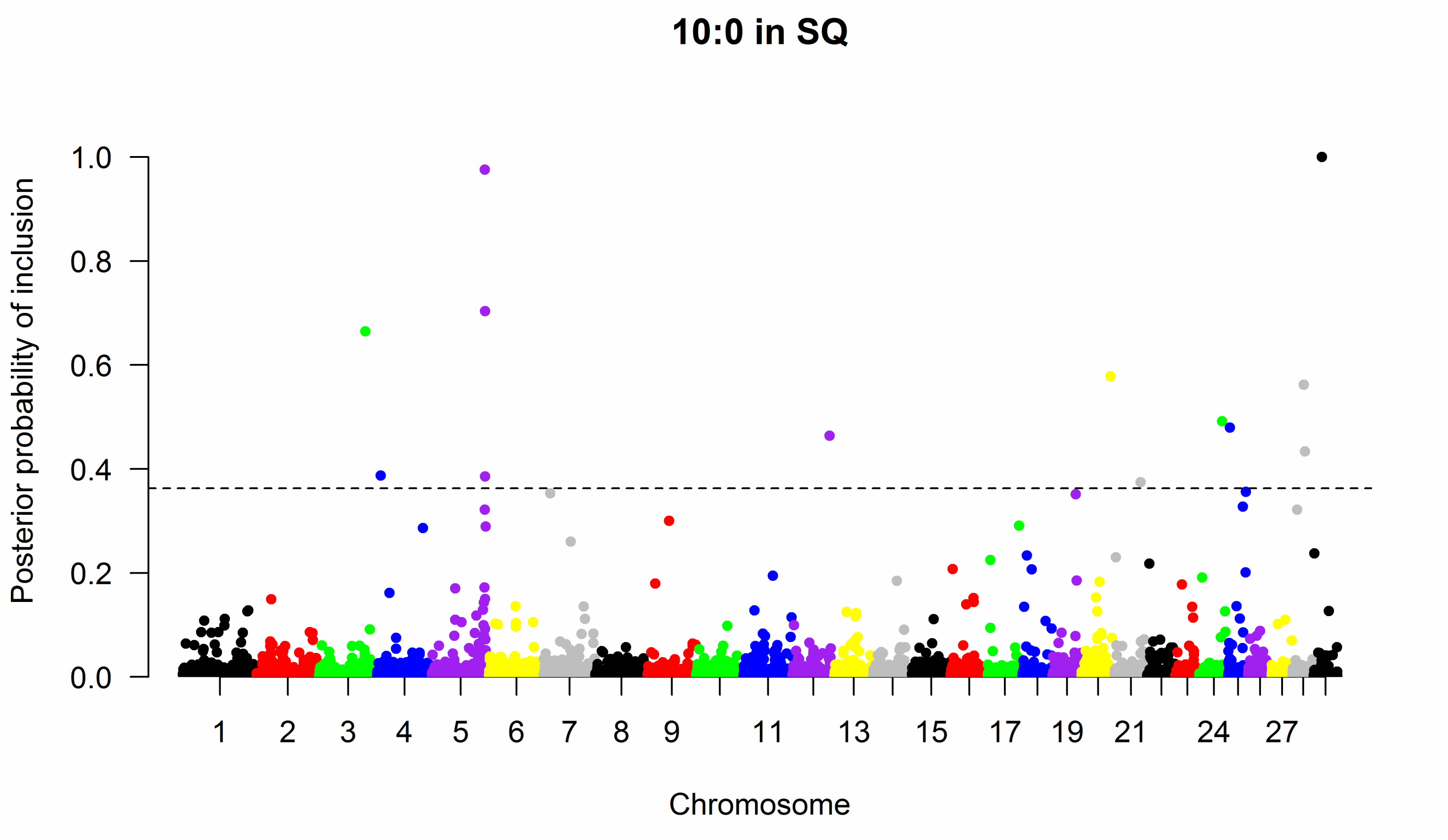

10:0 in SQ
Posterior probability of inclusion
1.0
0.8
0.6
0.4
0.2
0.0
1
2
3
4
5
6
7
8
9
11
13
15
17
19
21
24
27
Chromosome

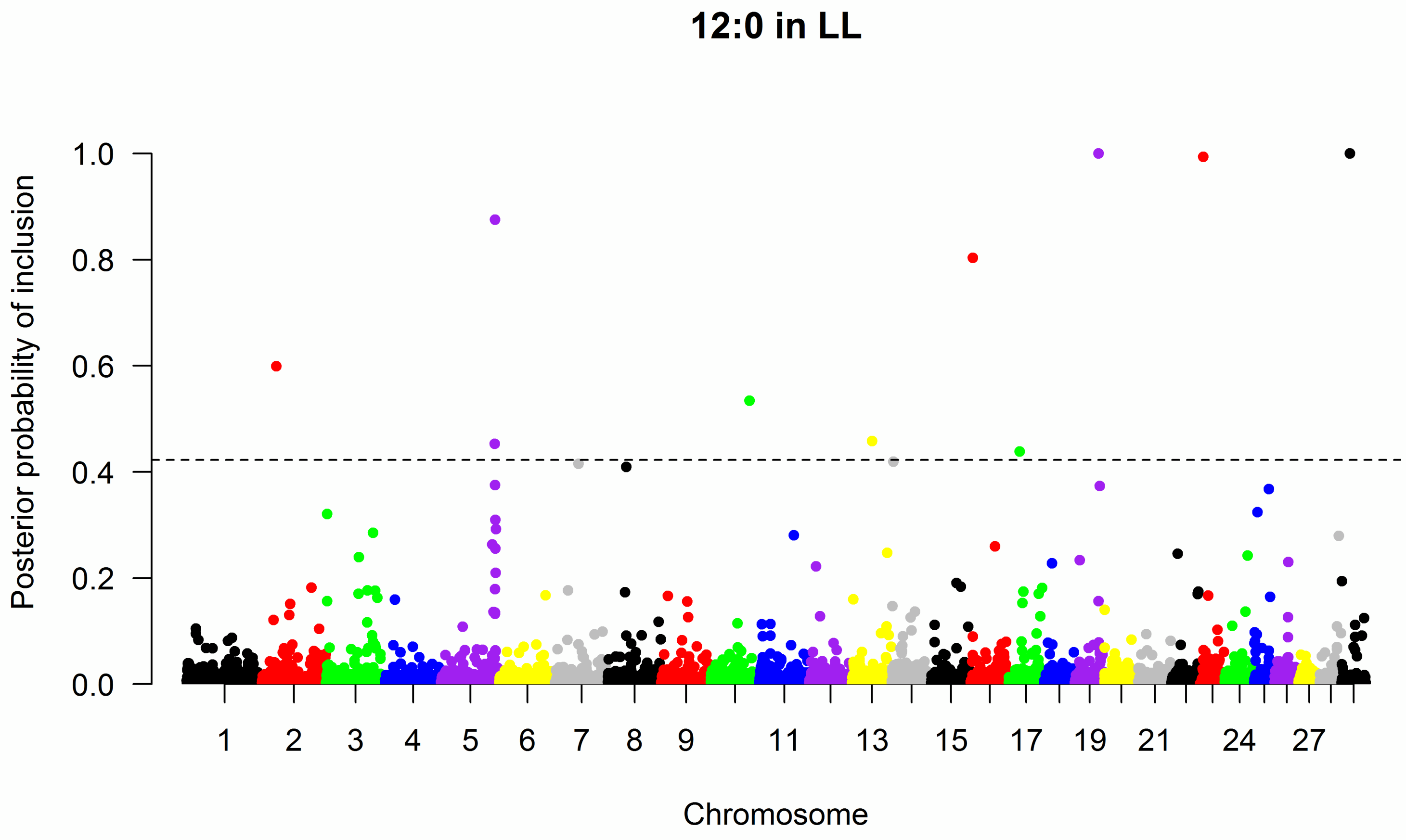
12:0 in LL
Posterior probability of inclusion
0.0
0.2
0.4
0.6
0.8
1.0
1
2
3
4
5
6
7
8
9
11
13
15
17
19
21
24
27
Chromosome

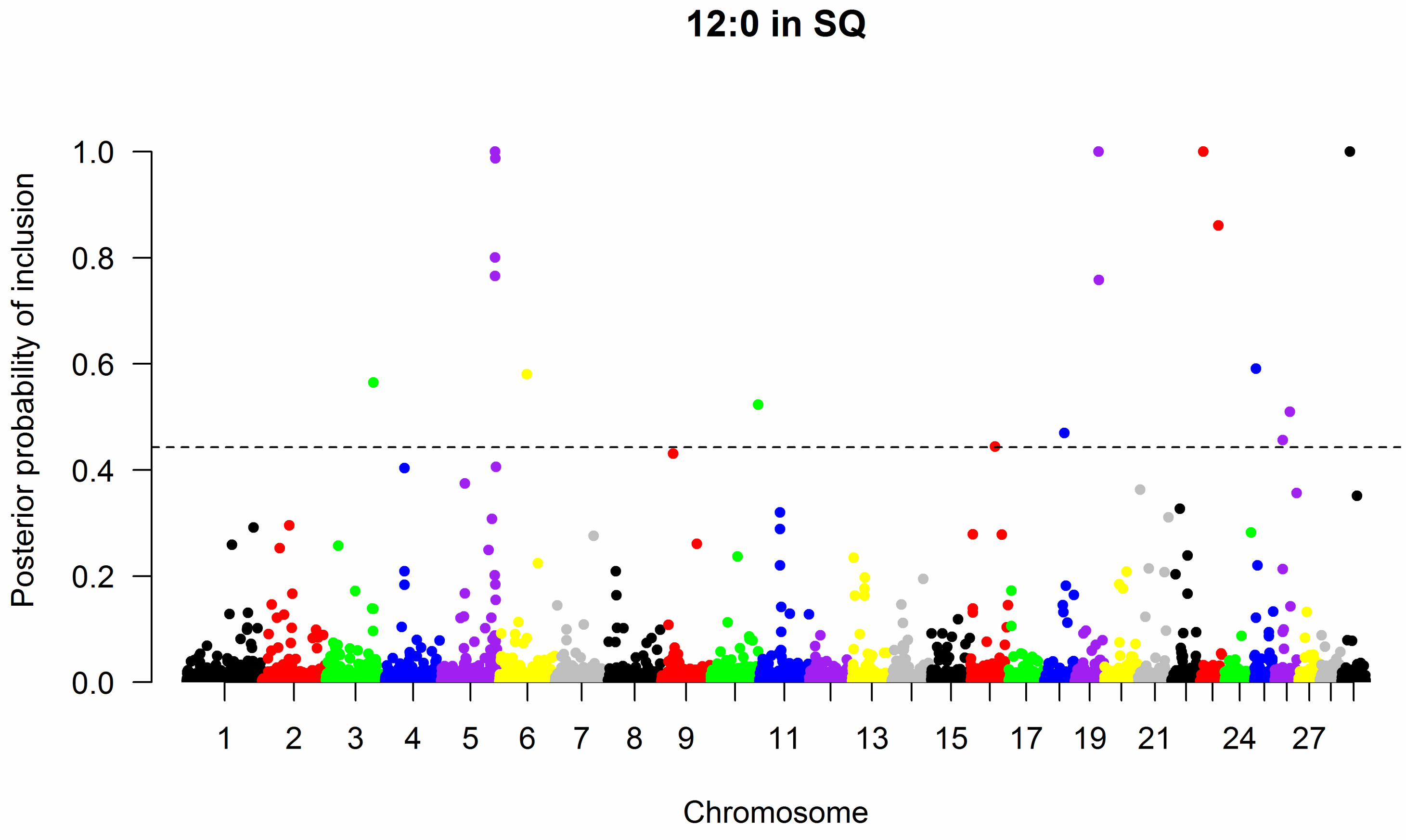
12:0 in SQ
Posterior probability of inclusion
0.0
0.2
0.4
0.6
0.8
1.0
1
2
3
4
5
6
7
8
9
11
13
15
17
19
21
24
27
Chromosome

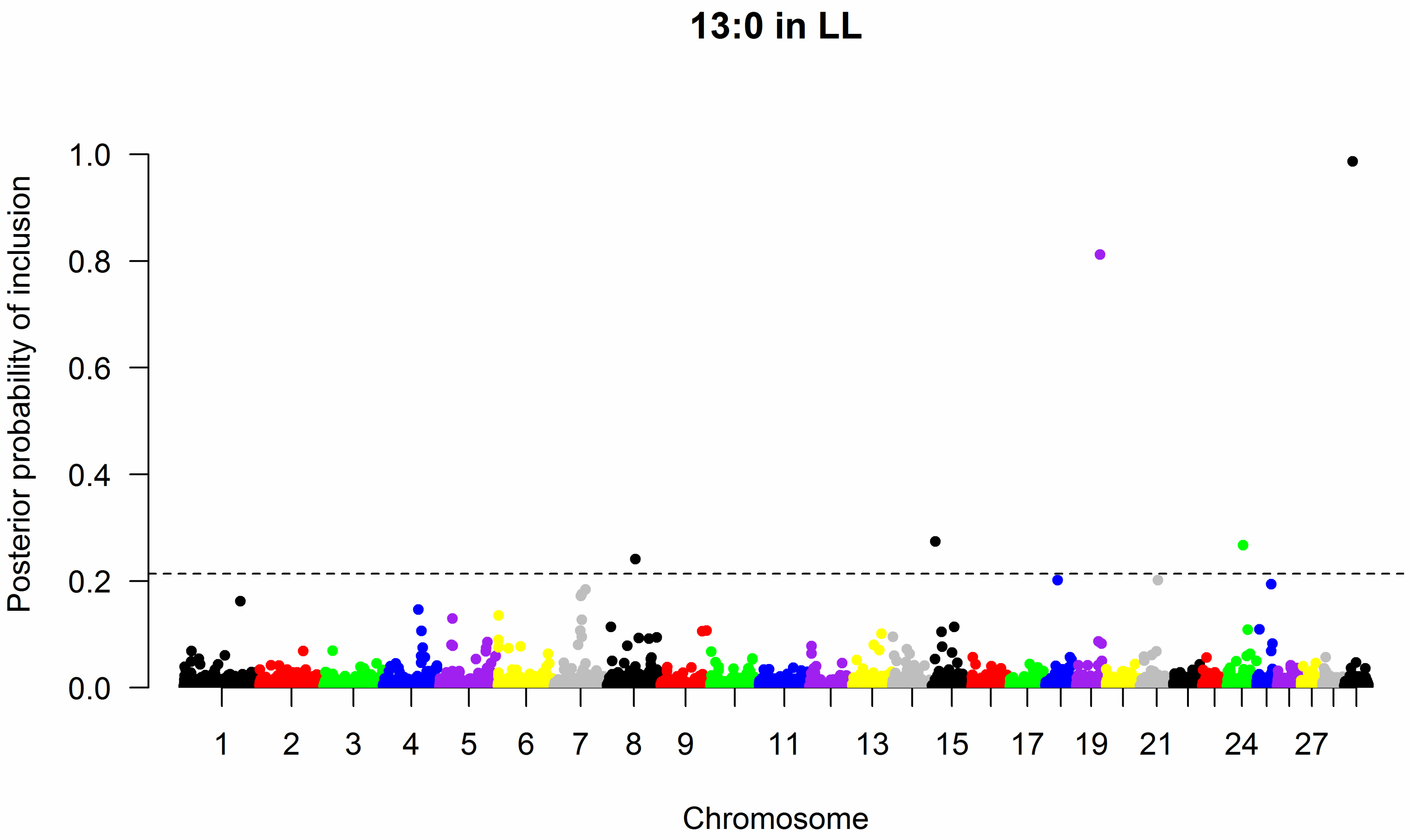
13:0 in LL
Posterior probability of inclusion
1.0
0.8
0.6
0.4
0.2
0.0
1
2
3
4
5
6
7
8
9
11
13
15
17
19
21
24
27
Chromosome

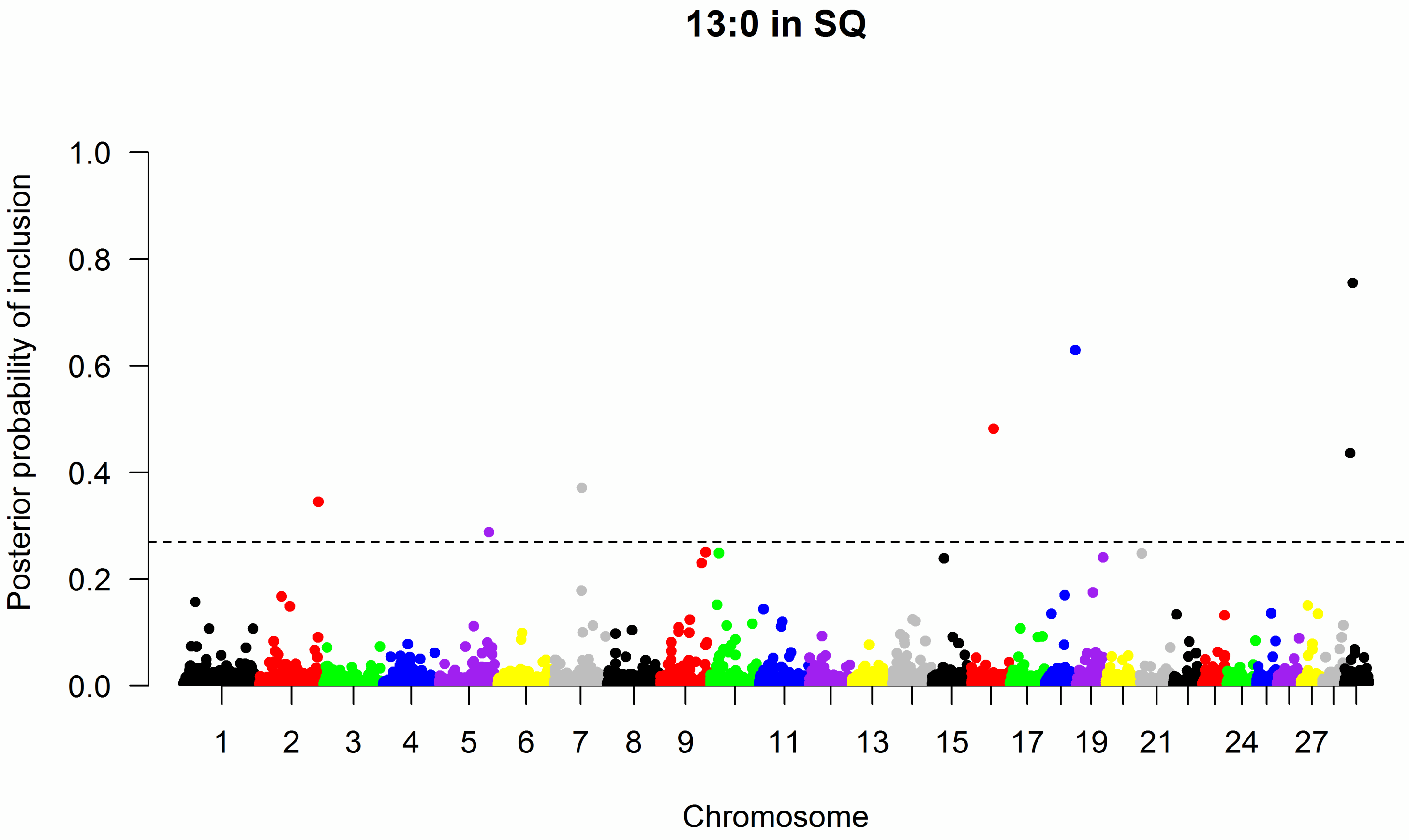
13:0 in SQ
Posterior probability of inclusion
1.0
0.8
0.6
0.4
0.2
0.0
1
2
3
4
5
6
7
8
9
11
13
15
17
19
21
24
27
Chromosome

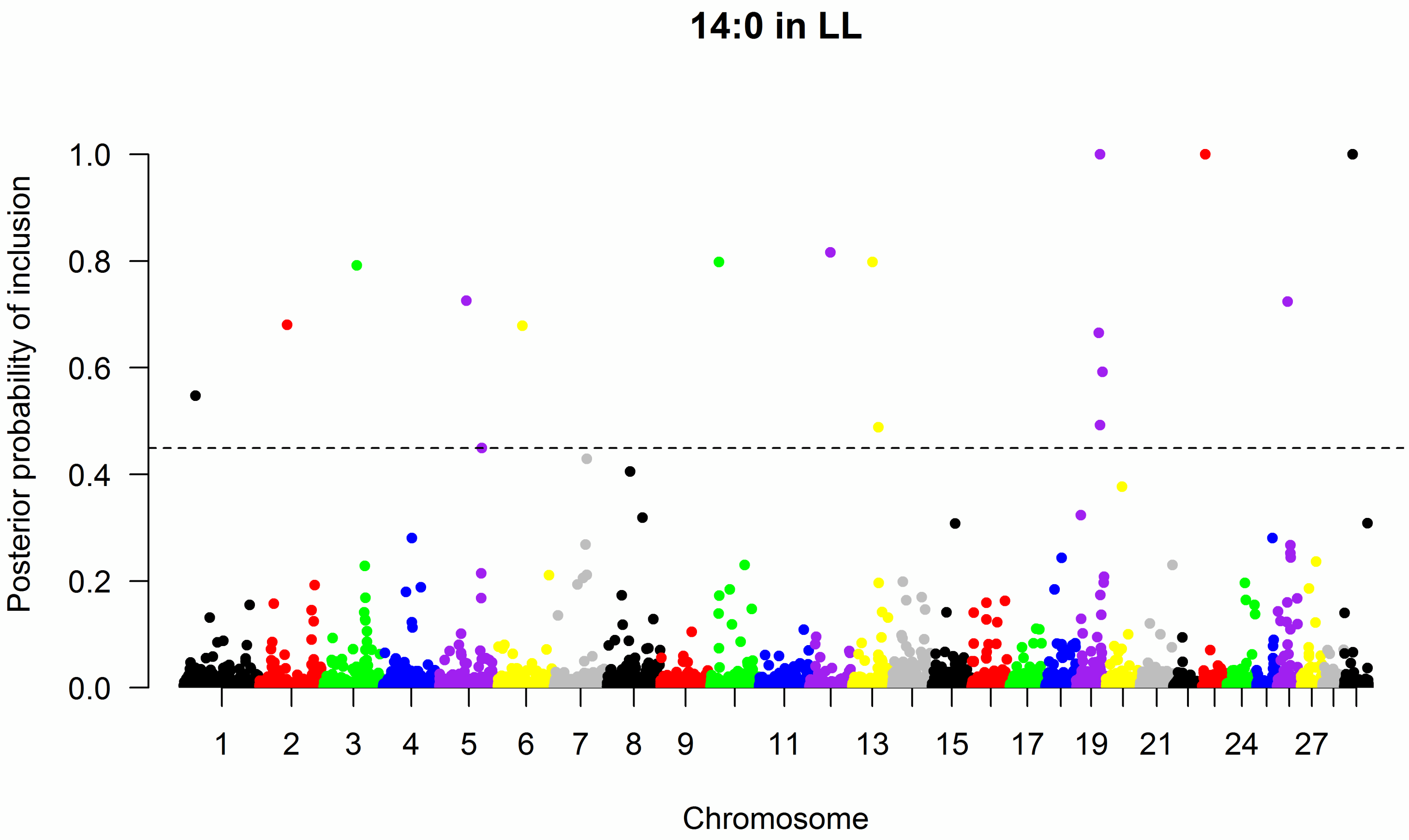
14:0 in LL
Posterior probability of inclusion
Chromosome
0.0
0.2
0.4
0.6
0.8
1.0
1
2
3
4
5
6
7
8
9
11
13
15
17
19
21
24
27

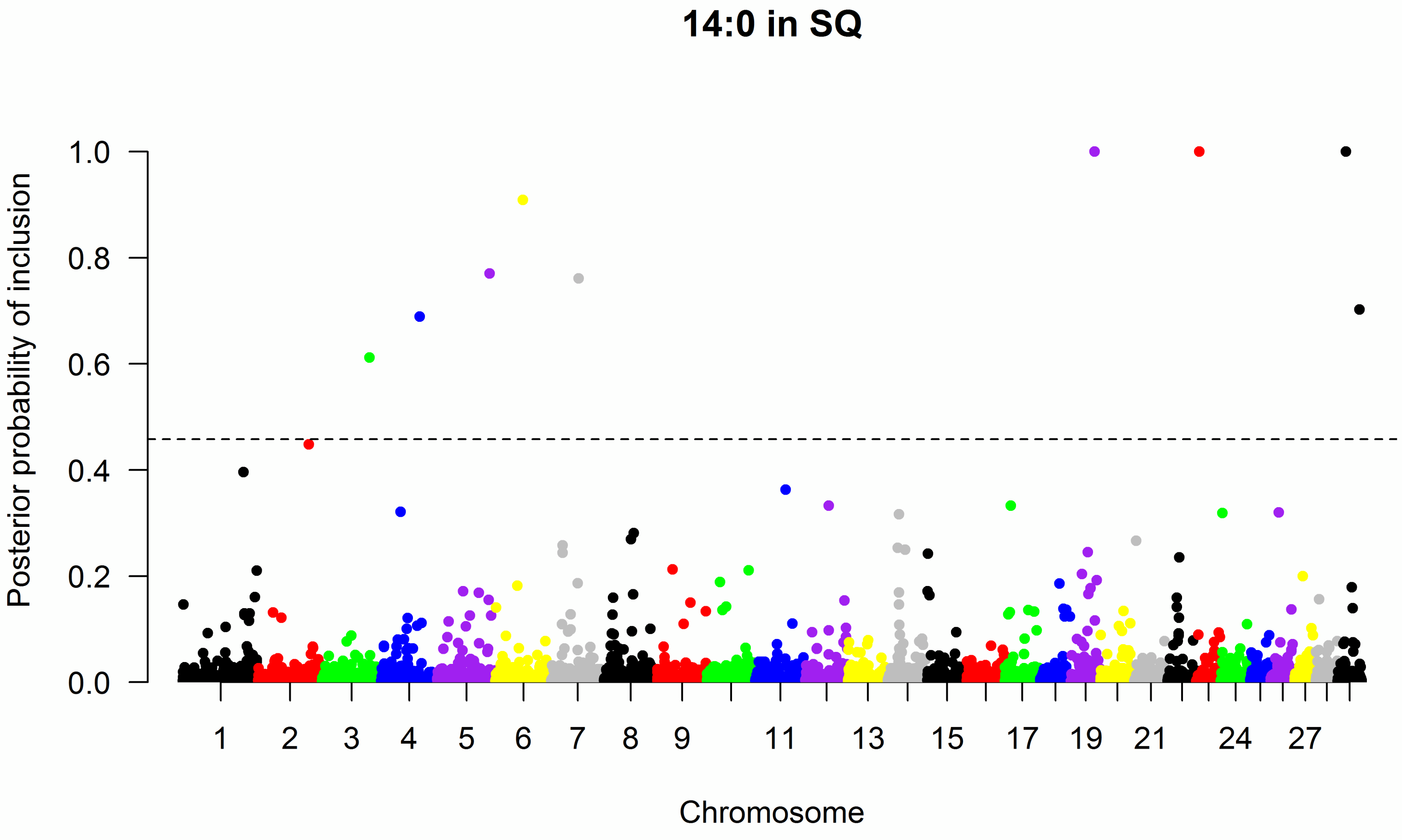
14:0 in SQ
Posterior probability of inclusion
Chromosome
0.0
0.2
0.4
0.6
0.8
1.0
1
2
3
4
5
6
7
8
9
11
13
15
17
19
21
24
27

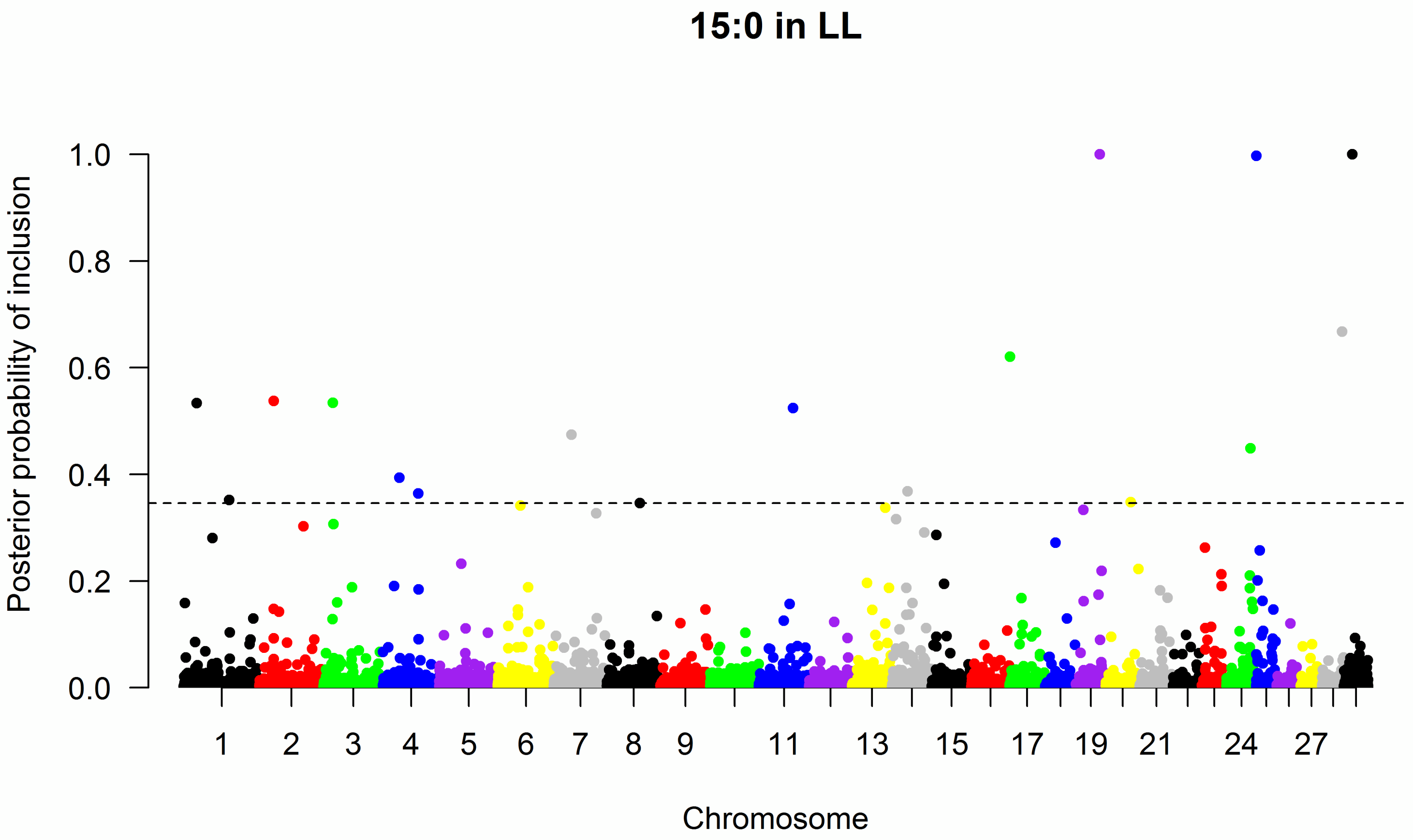
15:0 in LL
Posterior probability of inclusion
0.0
0.2
0.4
0.6
0.8
1.0
1 2 3 4 5 6 7 8 9 11 13 15 17 19 21 24 27
Chromosome

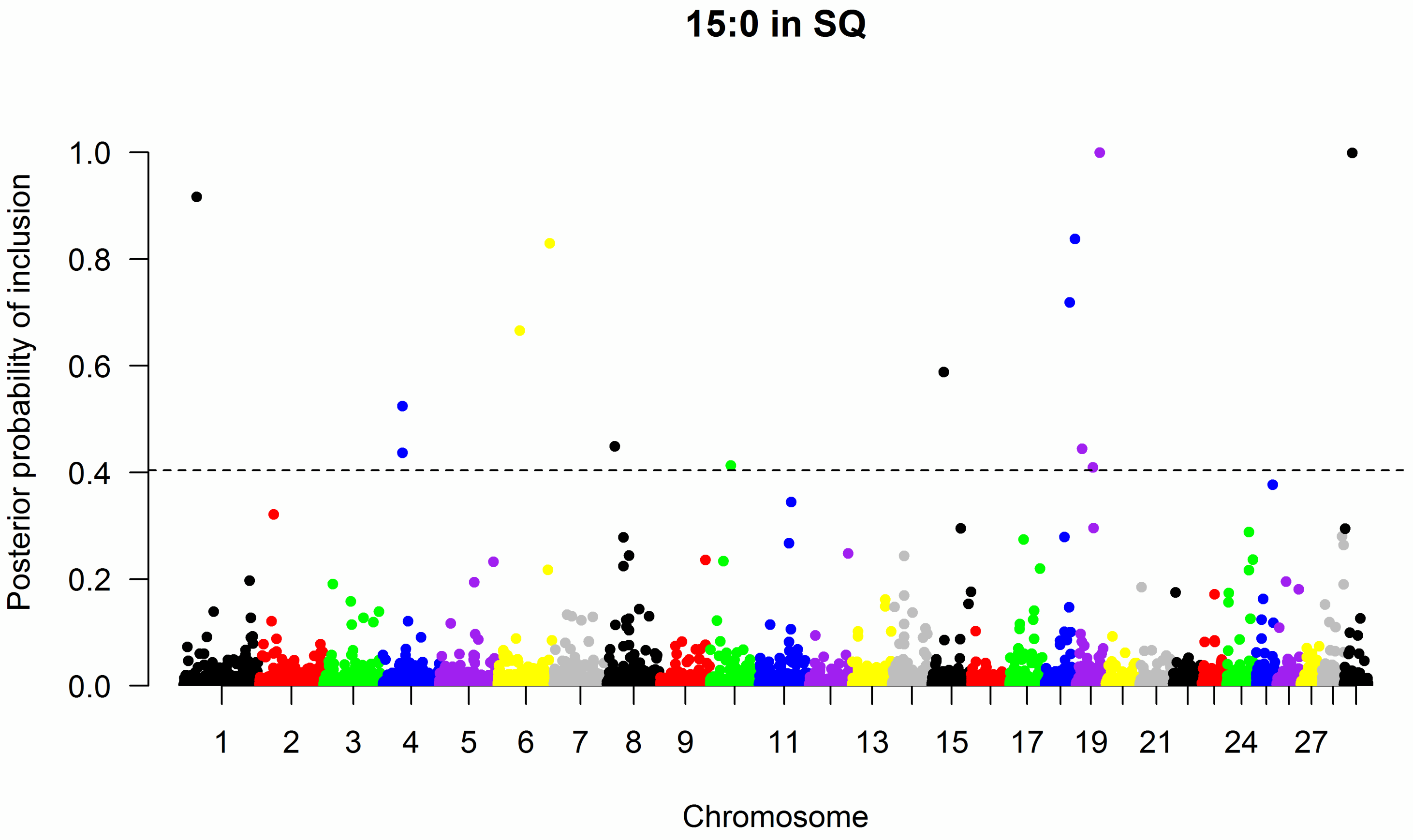
15:0 in SQ
Posterior probability of inclusion
0.0
0.2
0.4
0.6
0.8
1.0
1 2 3 4 5 6 7 8 9 11 13 15 17 19 21 24 27
Chromosome

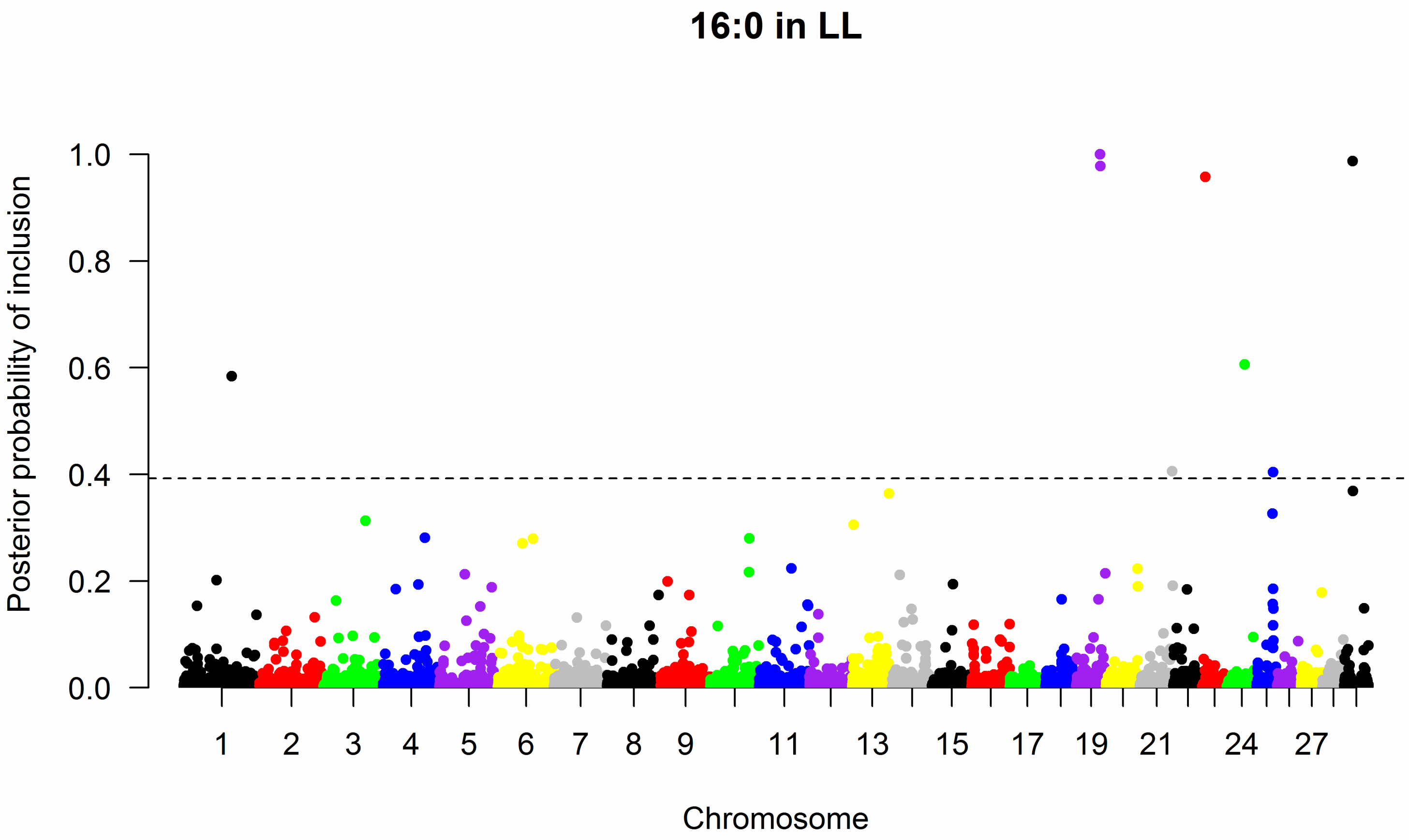
16:0 in LL
Posterior probability of inclusion
0.0
0.2
0.4
0.6
0.8
1.0
1
2
3
4
5
6
7
8
9
11
13
15
17
19
21
24
27
Chromosome

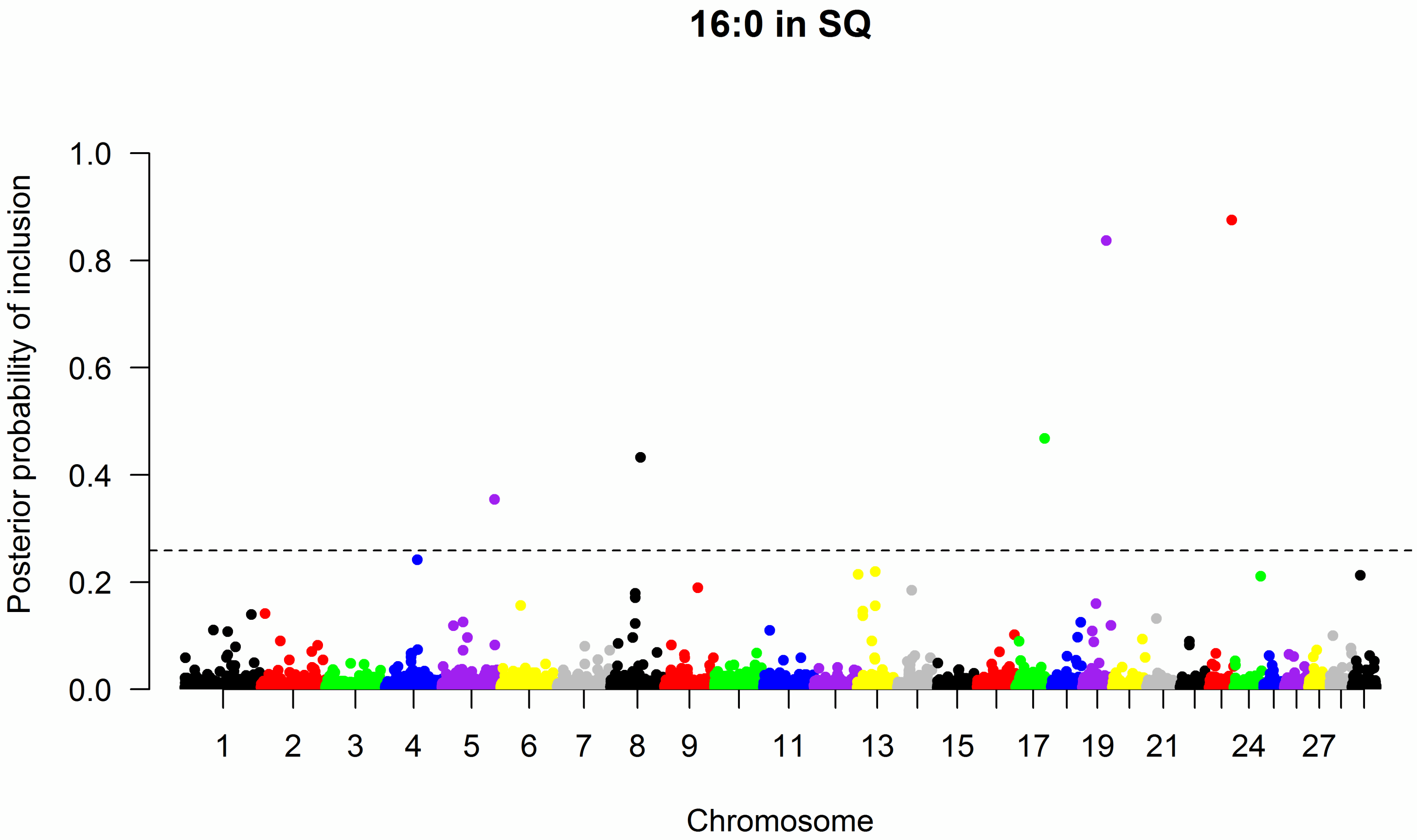
16:0 in SQ
Posterior probability of inclusion
0.0
0.2
0.4
0.6
0.8
1.0
1
2
3
4
5
6
7
8
9
11
13
15
17
19
21
24
27
Chromosome

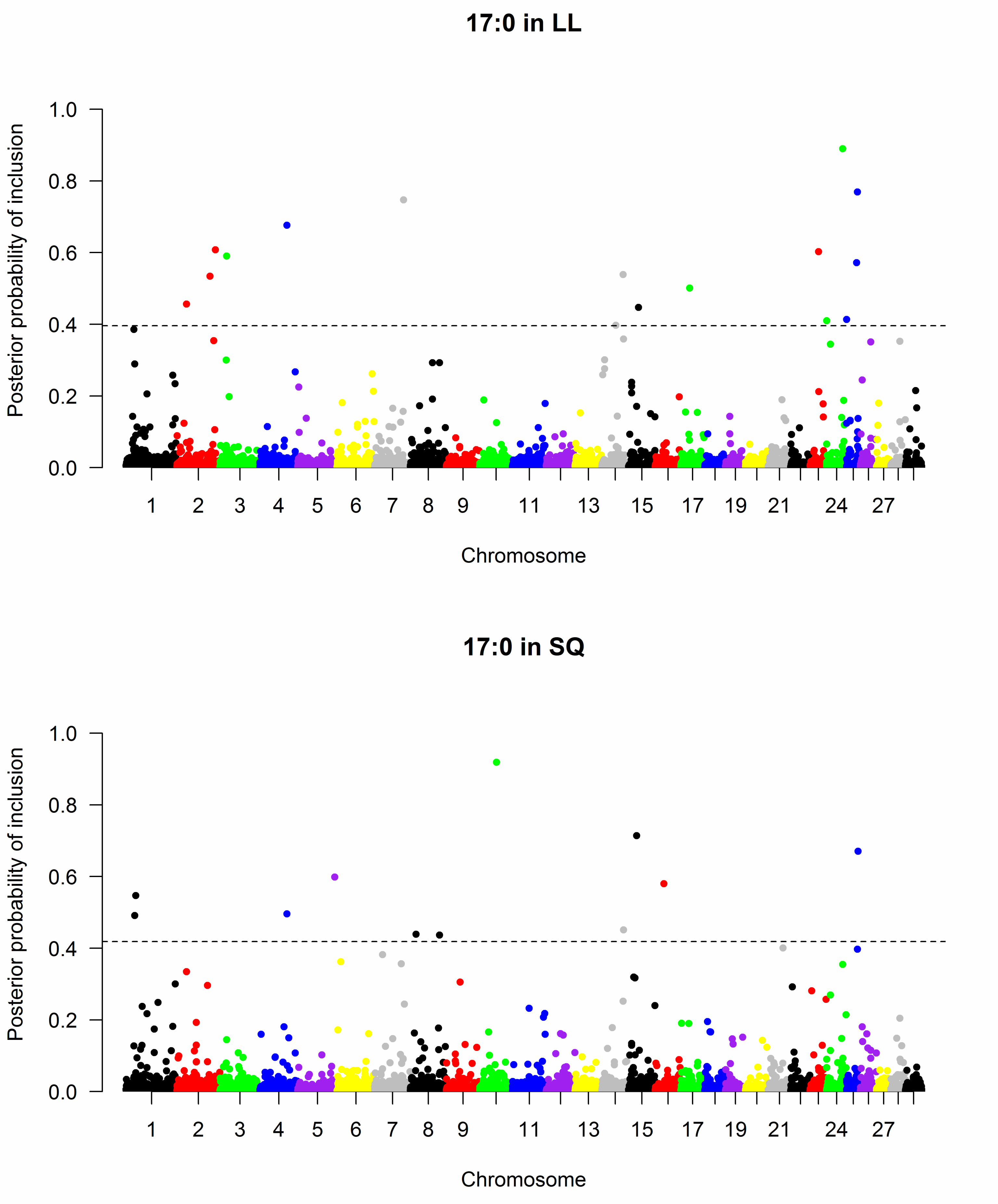

17:0 in LL
Posterior probability of inclusion
1.0
0.8
0.6
0.4
0.2
0.0
1 2 3 4 5 6 7 8 9 11 13 15 17 19 21 24 27
Chromosome
17:0 in SQ
Posterior probability of inclusion
1.0
0.8
0.6
0.4
0.2
0.0
1 2 3 4 5 6 7 8 9 11 13 15 17 19 21 24 27
Chromosome

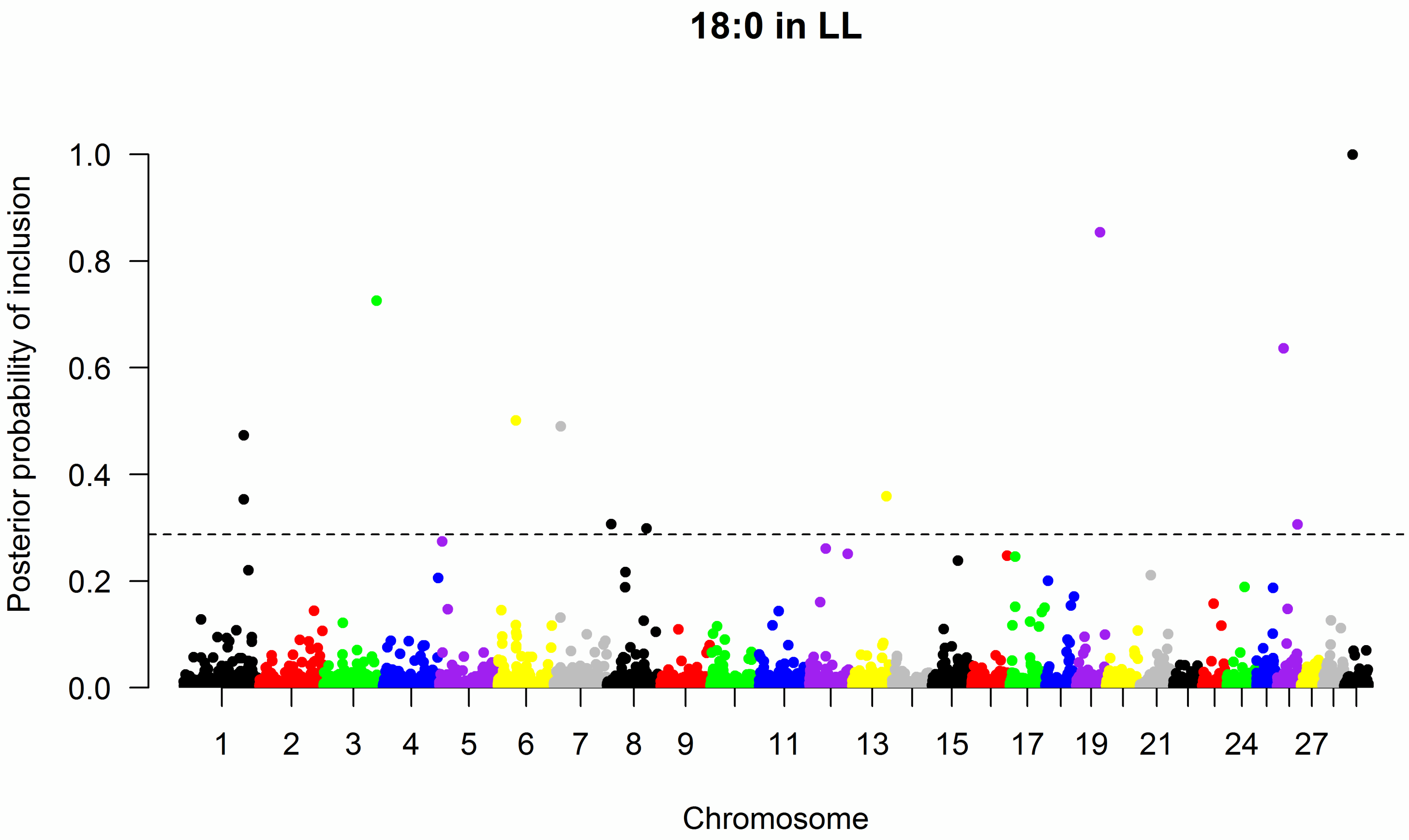
18:0 in LL
Posterior probability of inclusion
0.0
0.2
0.4
0.6
0.8
1.0
1
2
3
4
5
6
7
8
9
11
13
15
17
19
21
24
27
Chromosome

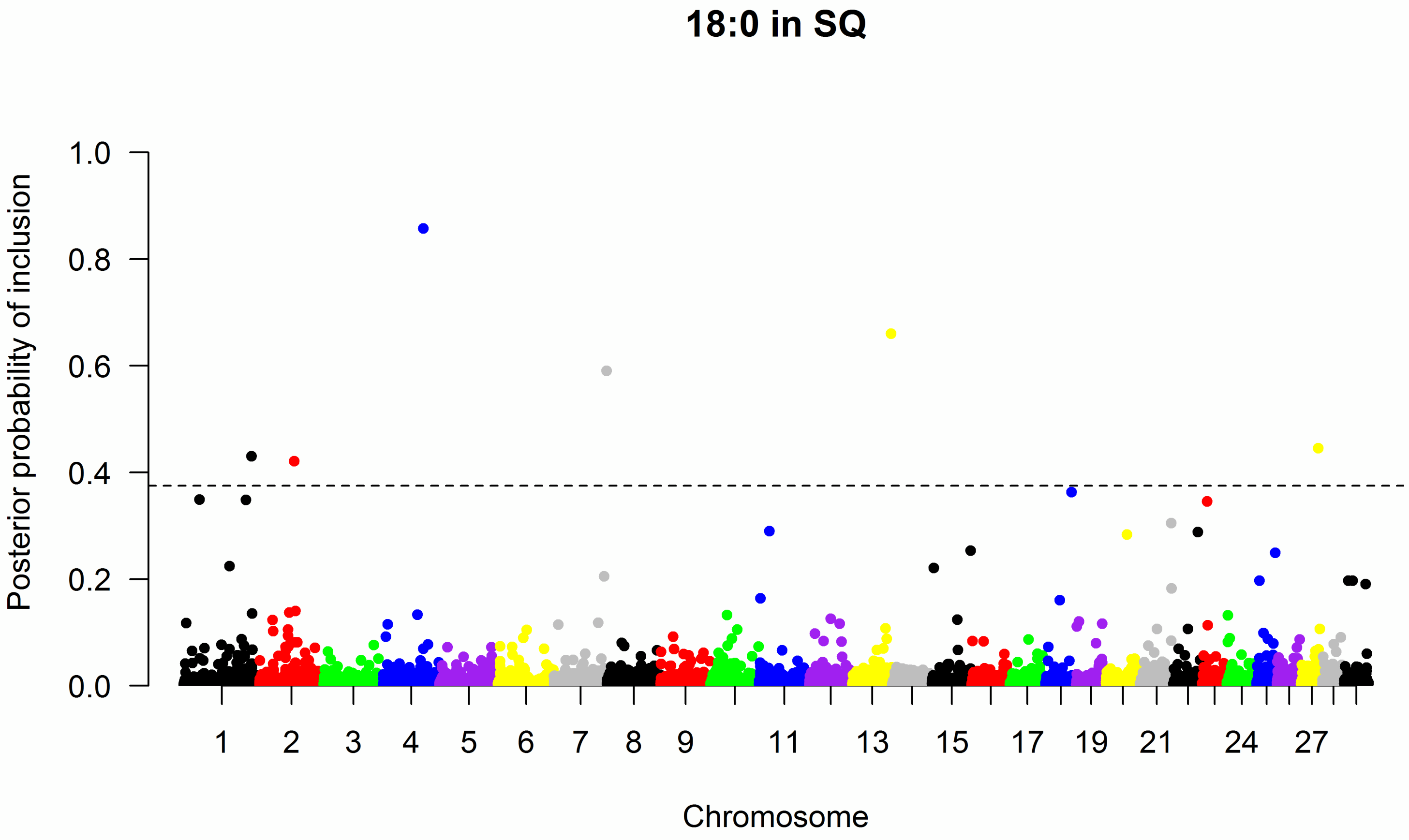
18:0 in SQ
Posterior probability of inclusion
0.0
0.2
0.4
0.6
0.8
1.0
1
2
3
4
5
6
7
8
9
11
13
15
17
19
21
24
27
Chromosome

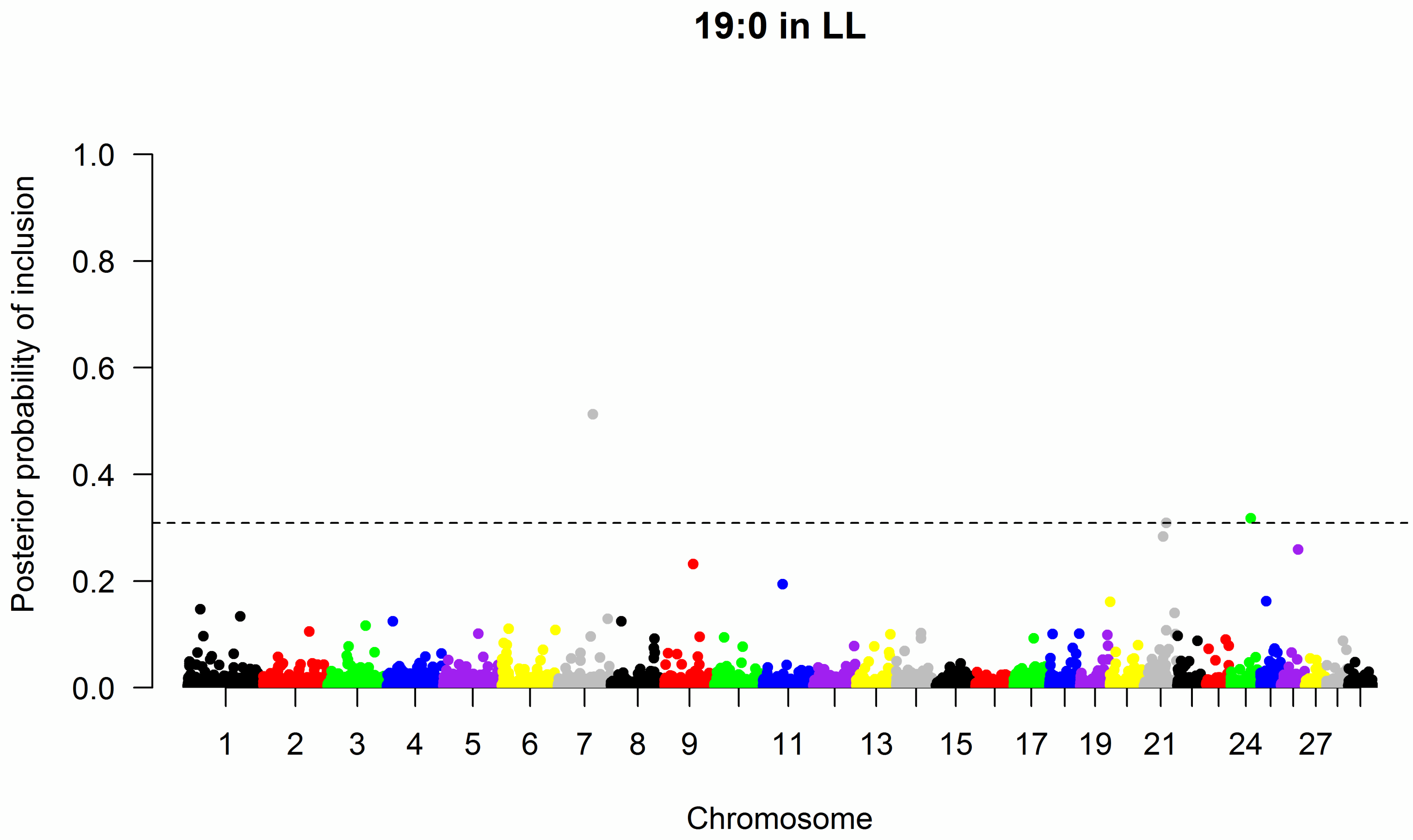
19:0 in LL
Posterior probability of inclusion
0.0
0.2
0.4
0.6
0.8
1.0
1 2 3 4 5 6 7 8 9 11 13 15 17 19 21 24 27
Chromosome

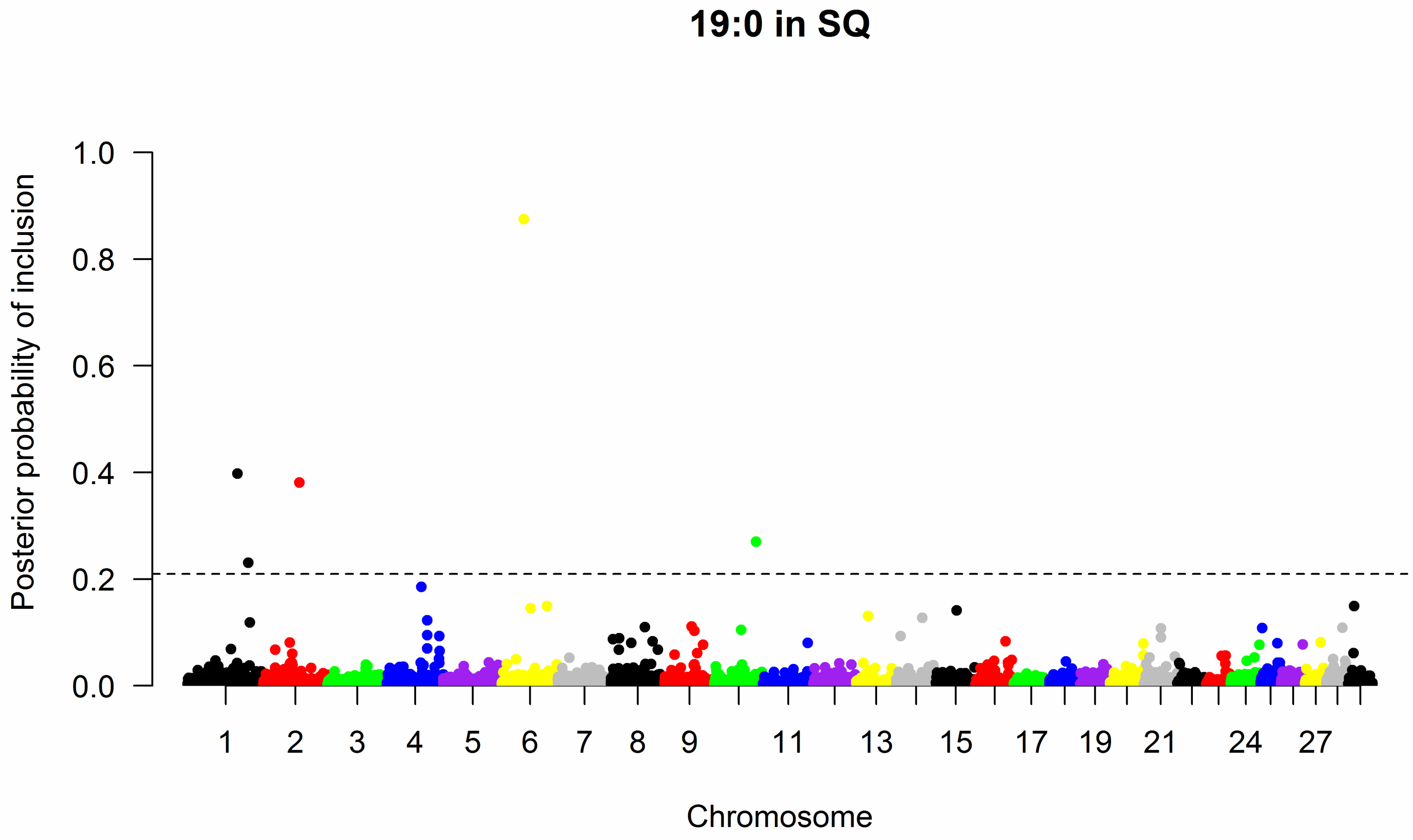
19:0 in SQ
Posterior probability of inclusion
0.0
0.2
0.4
0.6
0.8
1.0
1 2 3 4 5 6 7 8 9 11 13 15 17 19 21 24 27
Chromosome

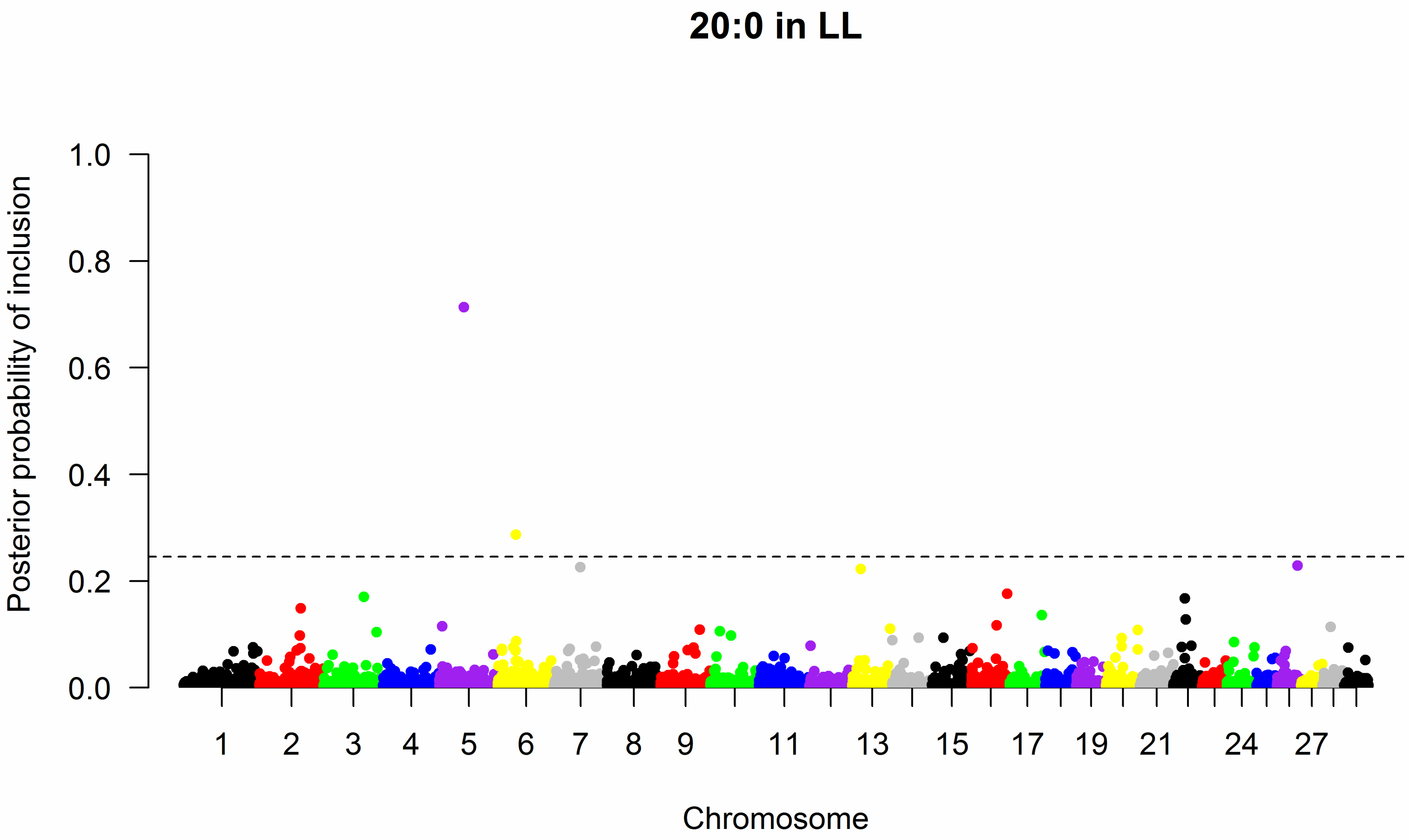

20:0 in LL
Posterior probability of inclusion
1.0
0.8
0.6
0.4
0.2
0.0
1 2 3 4 5 6 7 8 9 11 13 15 17 19 21 24 27
Chromosome

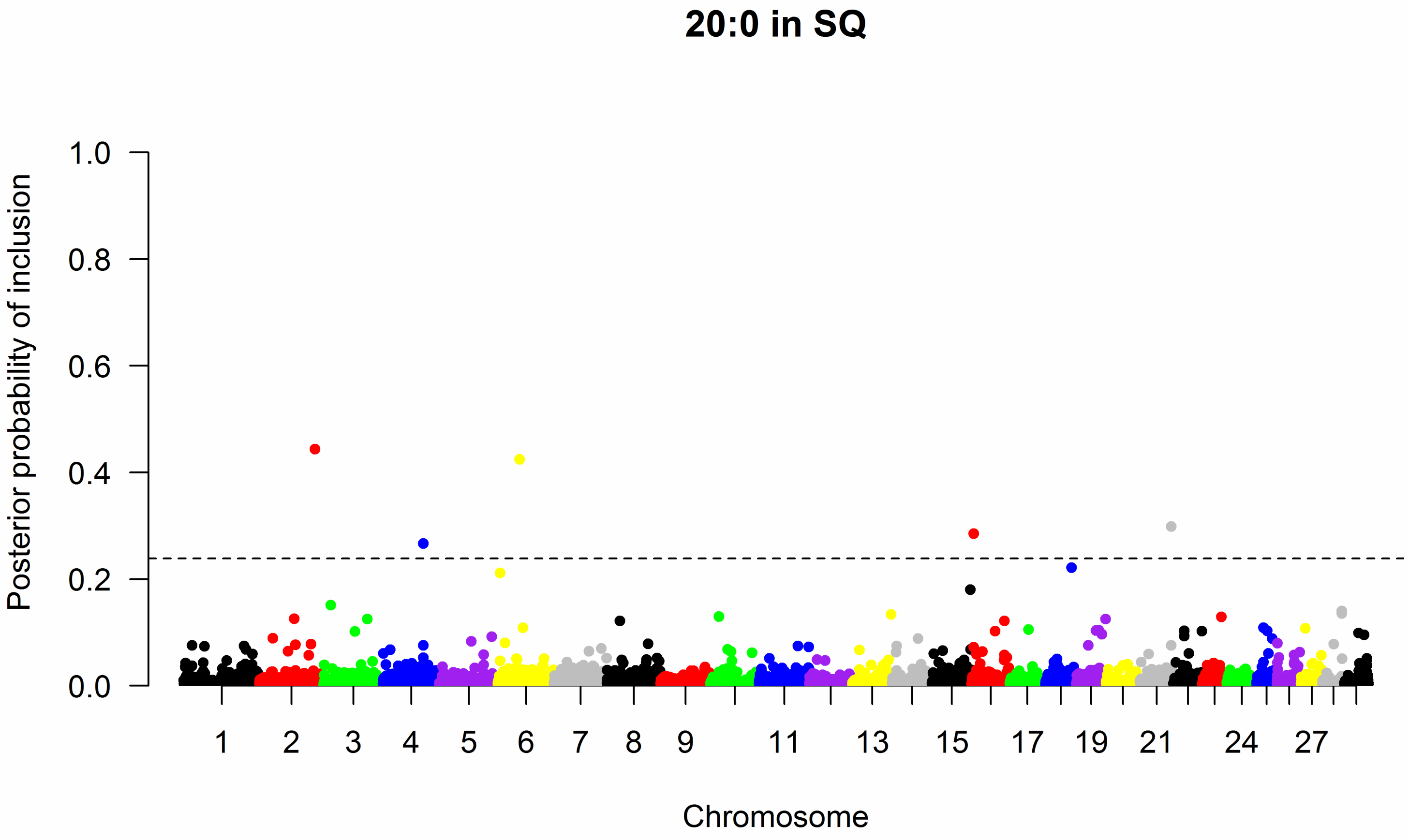

20:0 in SQ
Posterior probability of inclusion
1.0
0.8
0.6
0.4
0.2
0.0
1 2 3 4 5 6 7 8 9 11 13 15 17 19 21 24 27
Chromosome

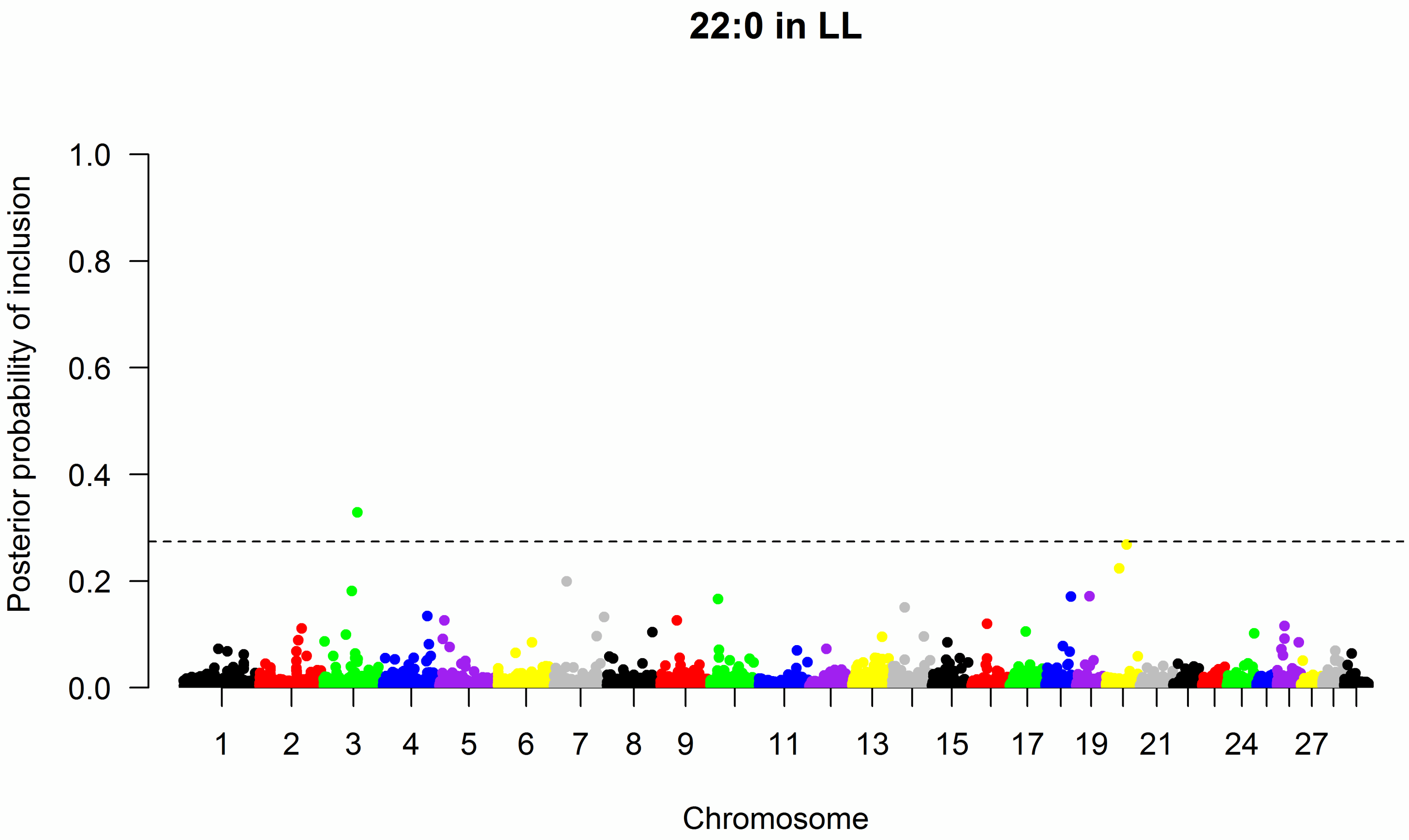
22:0 in LL
Posterior probability of inclusion
1.0
0.8
0.6
0.4
0.2
0.0
1
2
3
4
5
6
7
8
9
11
13
15
17
19
21
24
27
Chromosome

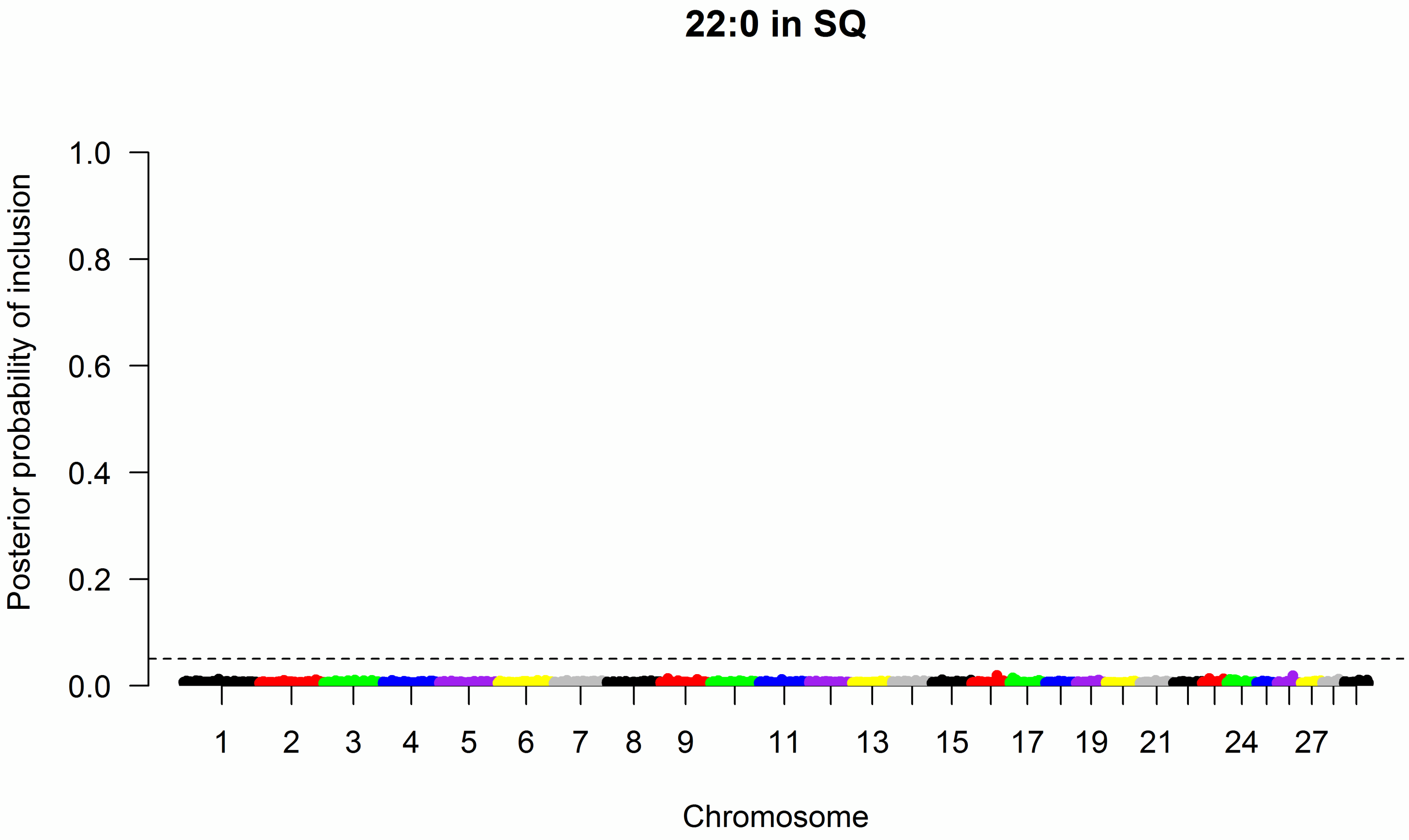
22:0 in SQ
Posterior probability of inclusion
1.0
0.8
0.6
0.4
0.2
0.0
1
2
3
4
5
6
7
8
9
11
13
15
17
19
21
24
27
Chromosome

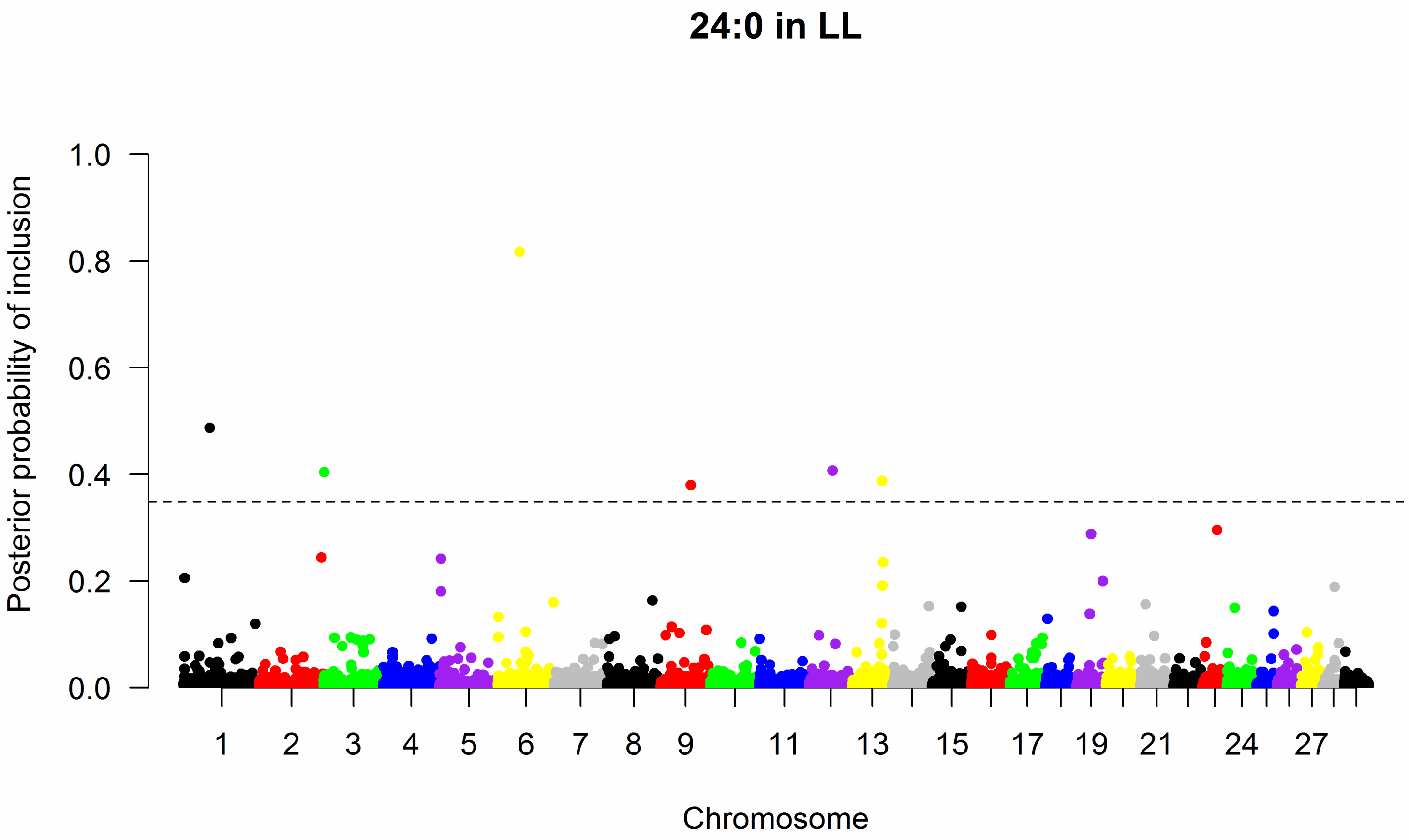
24:0 in LL
Posterior probability of inclusion
1.0
0.8
0.6
0.4
0.2
0.0
1
2
3
4
5
6
7
8
9
11
13
15
17
19
21
24
27
Chromosome

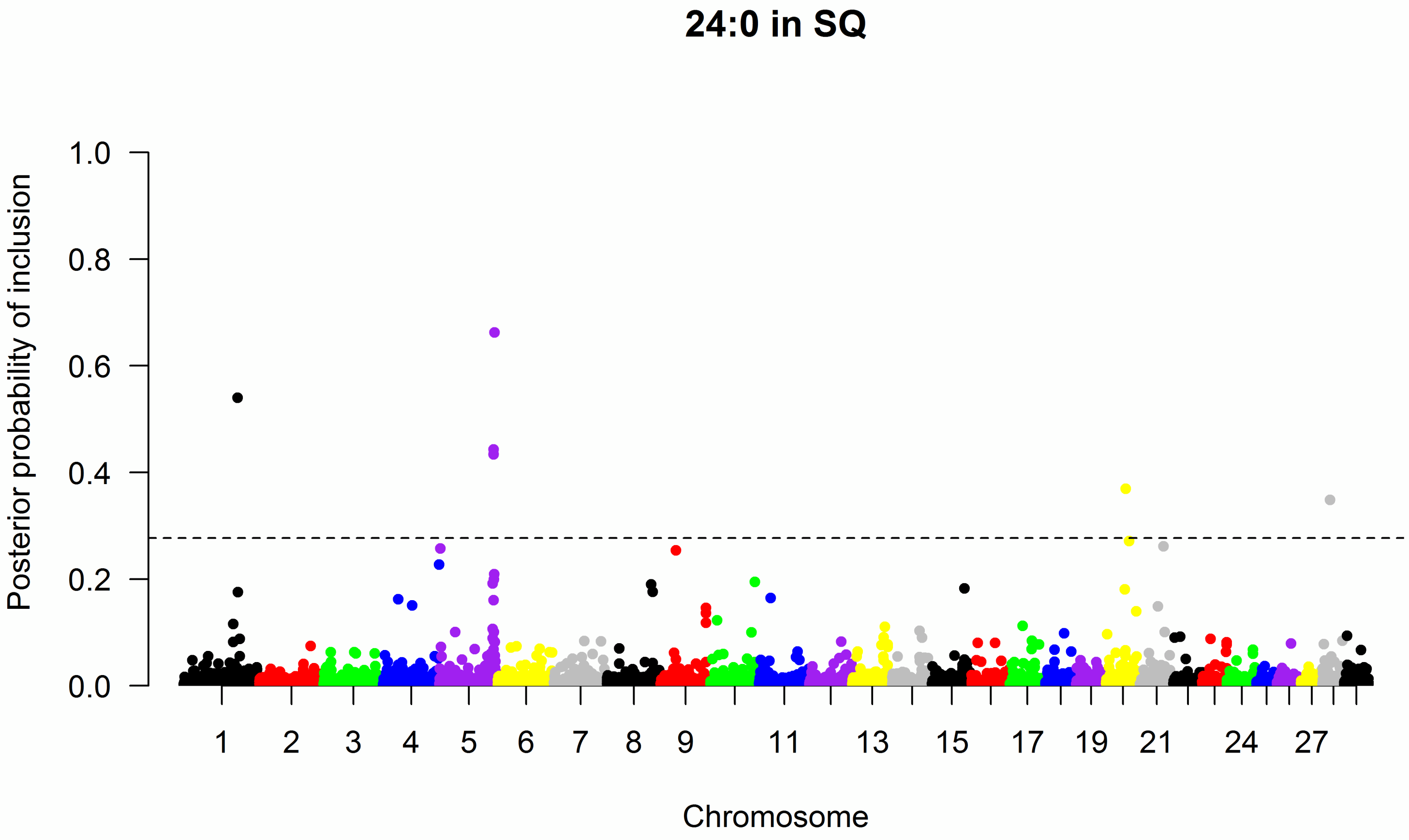
24:0 in SQ
Posterior probability of inclusion
1.0
0.8
0.6
0.4
0.2
0.0
1
2
3
4
5
6
7
8
9
11
13
15
17
19
21
24
27
Chromosome

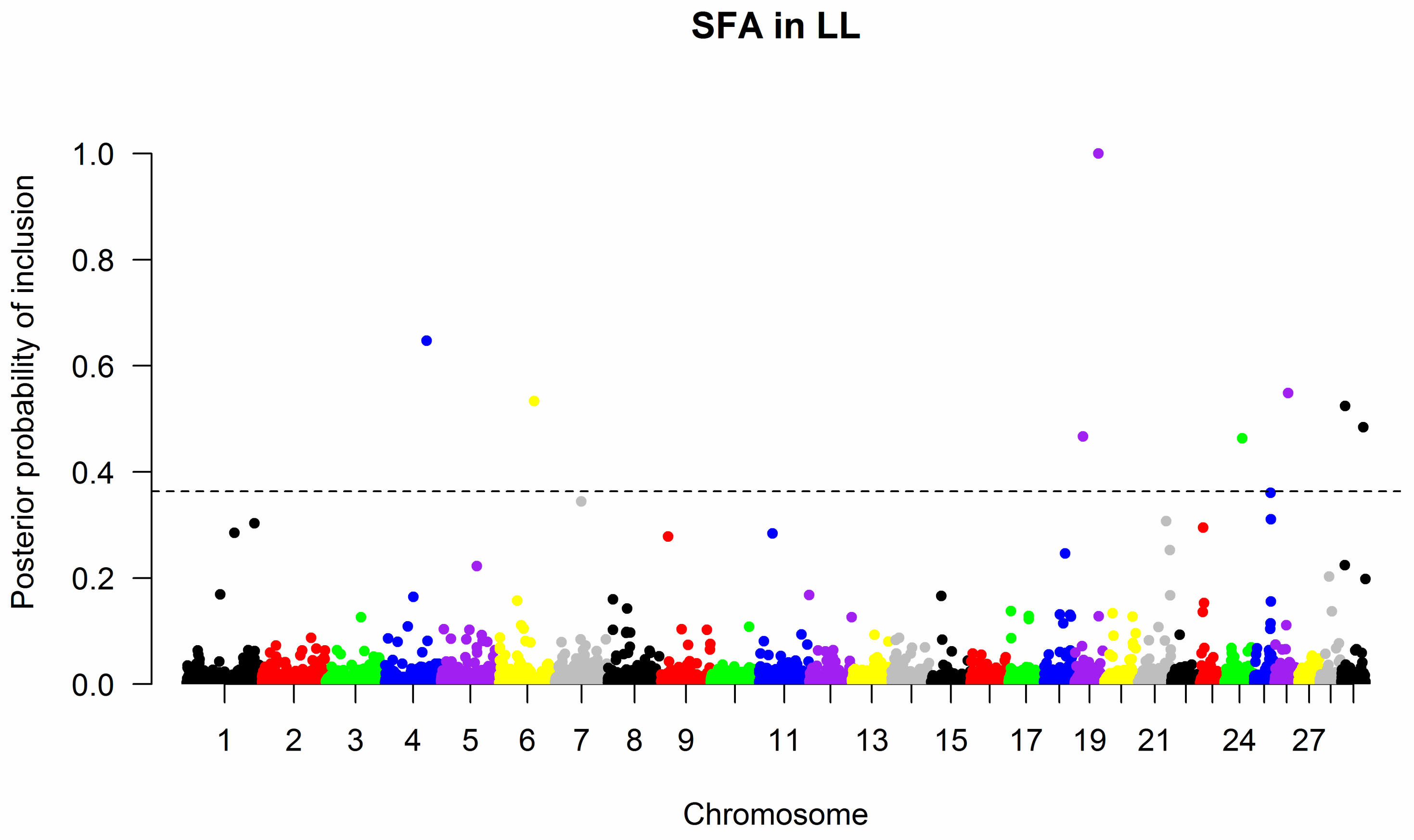
SFA in LL
Posterior probability of inclusion
1.0
0.8
0.6
0.4
0.2
0.0
1
2
3
4
5
6
7
8
9
11
13
15
17
19
21
24
27
Chromosome

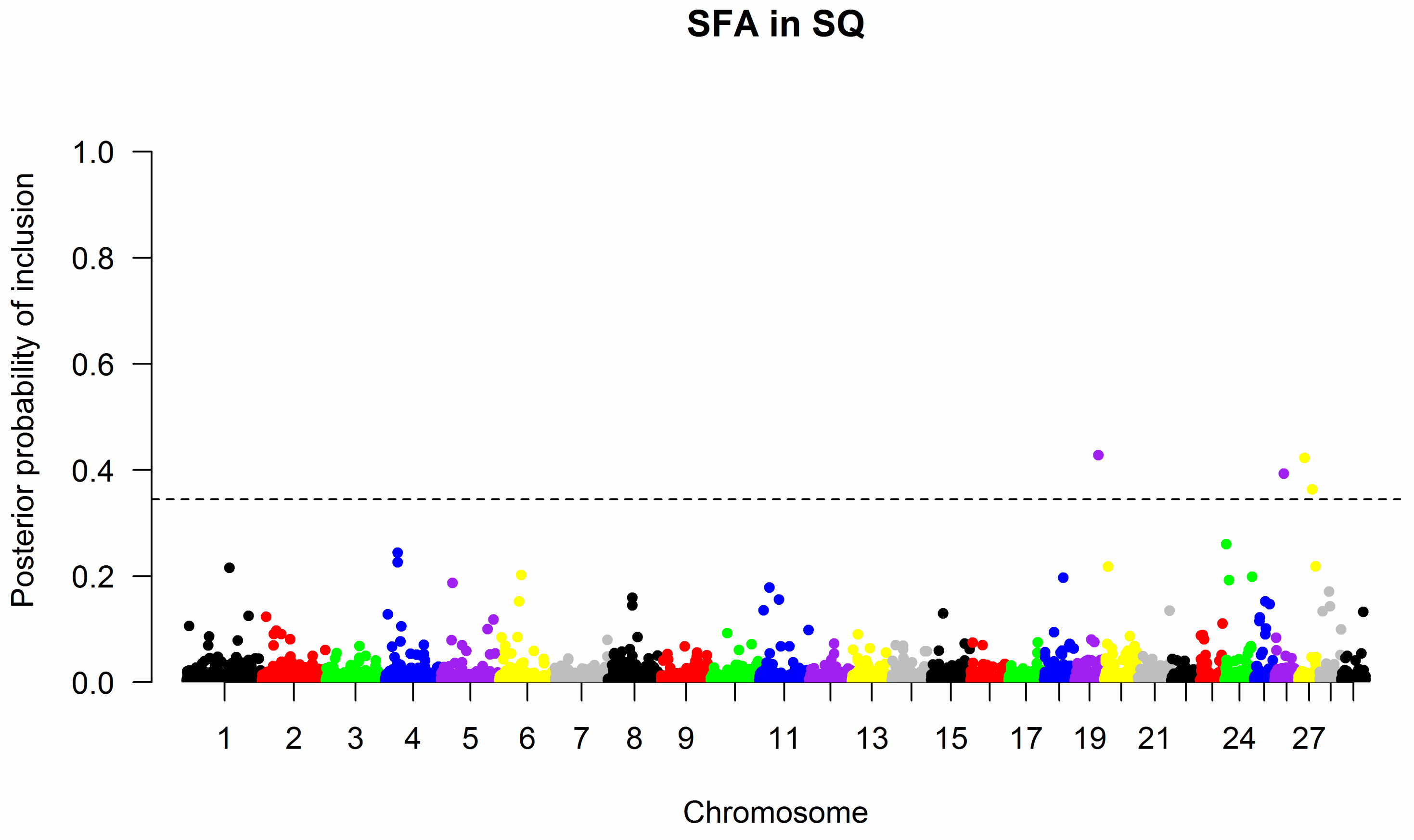
SFA in SQ
Posterior probability of inclusion
1.0
0.8
0.6
0.4
0.2
0.0
1
2
3
4
5
6
7
8
9
11
13
15
17
19
21
24
27
Chromosome

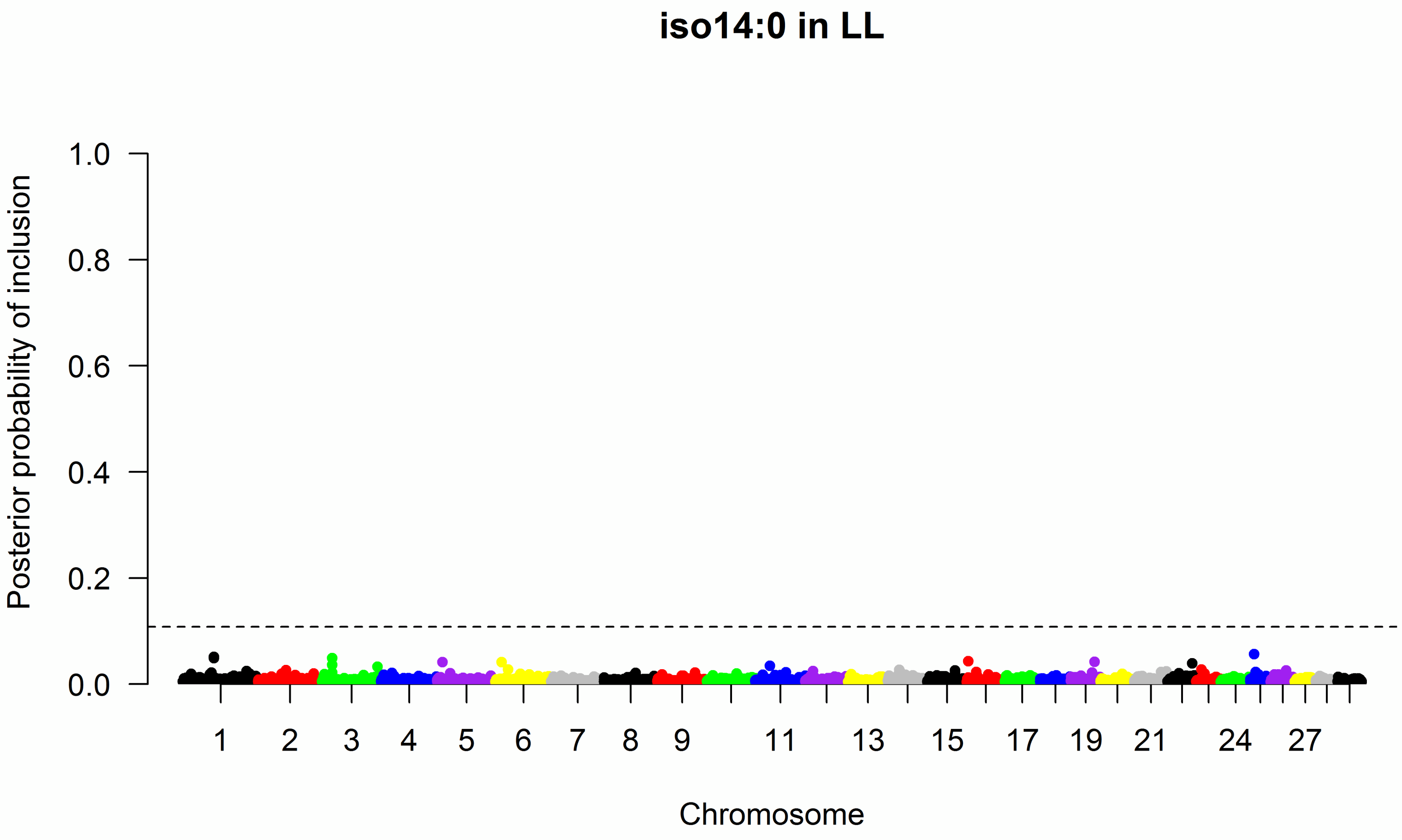
iso14:0 in LL
Posterior probability of inclusion
1.0
0.8
0.6
0.4
0.2
0.0
1 2 3 4 5 6 7 8 9 11 13 15 17 19 21 24 27
Chromosome

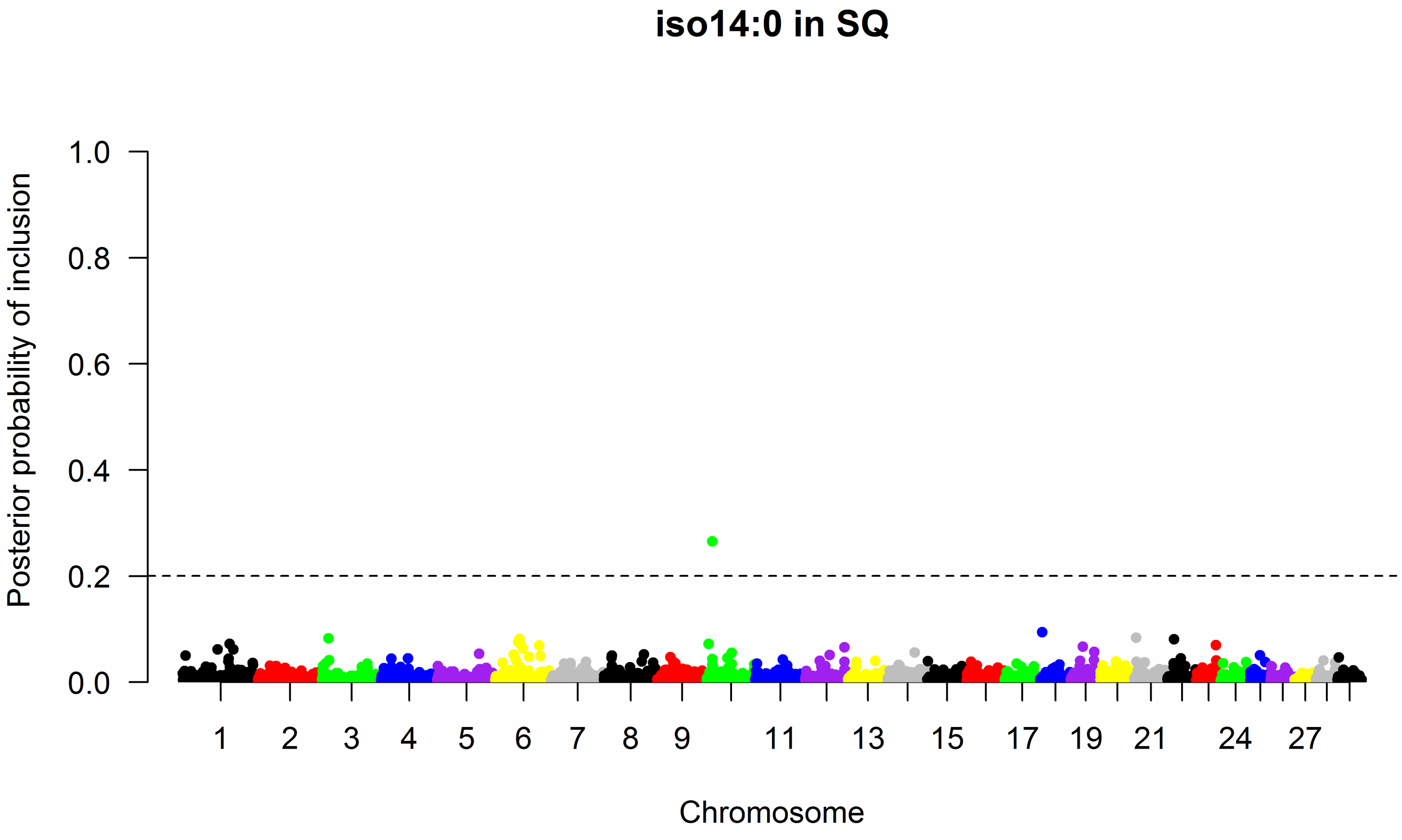
iso14:0 in SQ
Posterior probability of inclusion
1.0
0.8
0.6
0.4
0.2
0.0
1 2 3 4 5 6 7 8 9 11 13 15 17 19 21 24 27
Chromosome

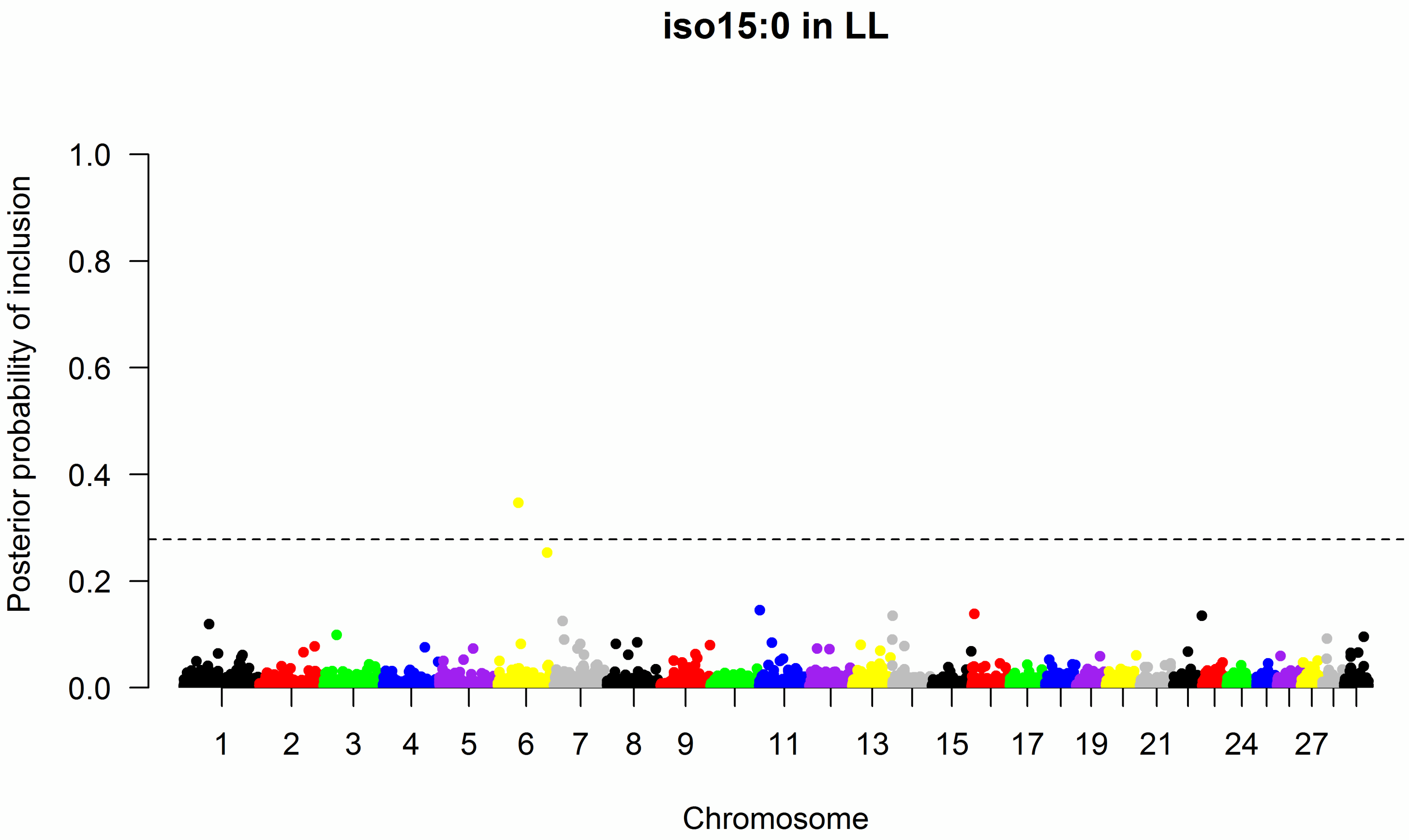

iso15:0 in LL
Posterior probability of inclusion
1.0
0.8
0.6
0.4
0.2
0.0
1
2
3
4
5
6
7
8
9
11
13
15
17
19
21
24
27
Chromosome

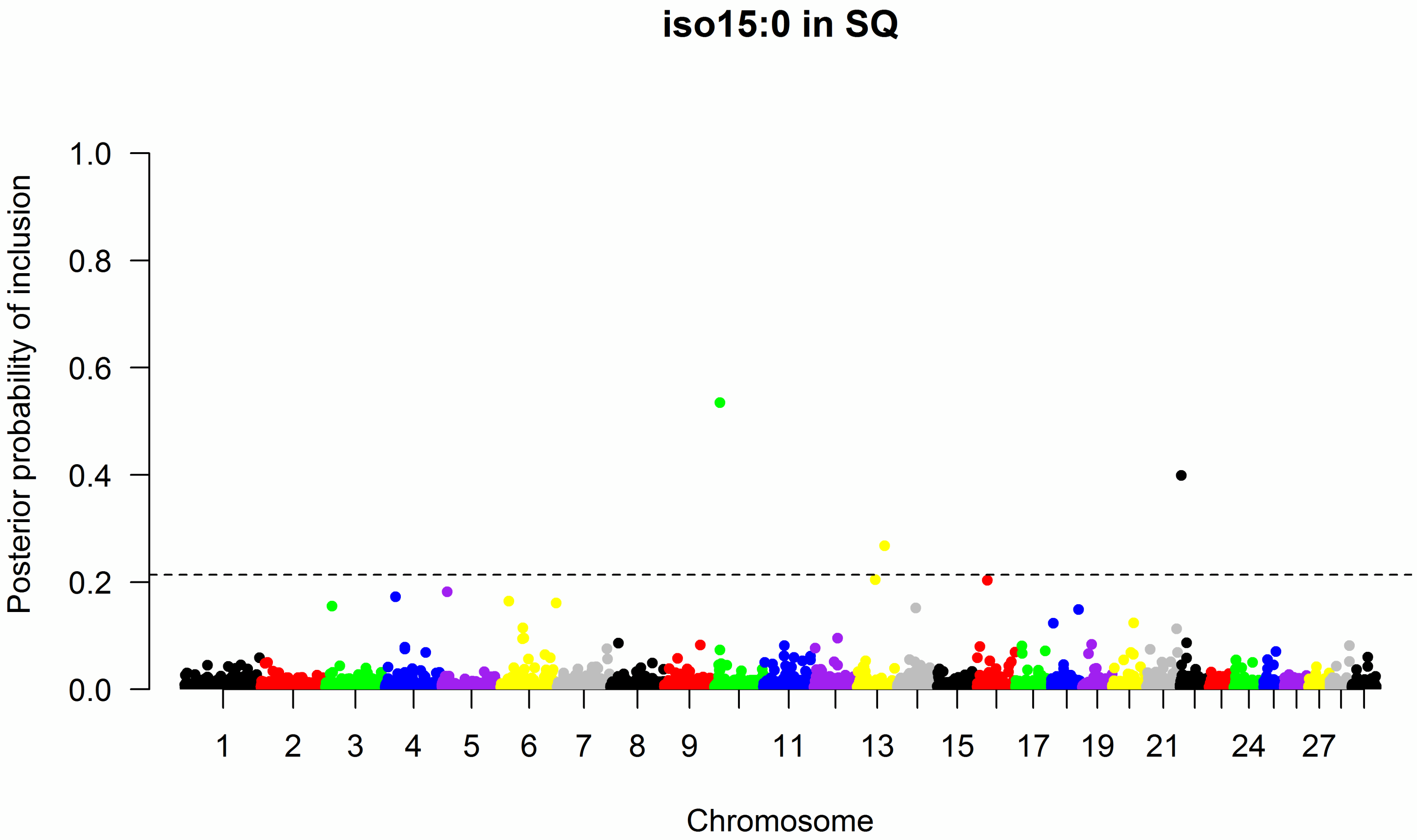

iso15:0 in SQ
Posterior probability of inclusion
1.0
0.8
0.6
0.4
0.2
0.0
1
2
3
4
5
6
7
8
9
11
13
15
17
19
21
24
27
Chromosome

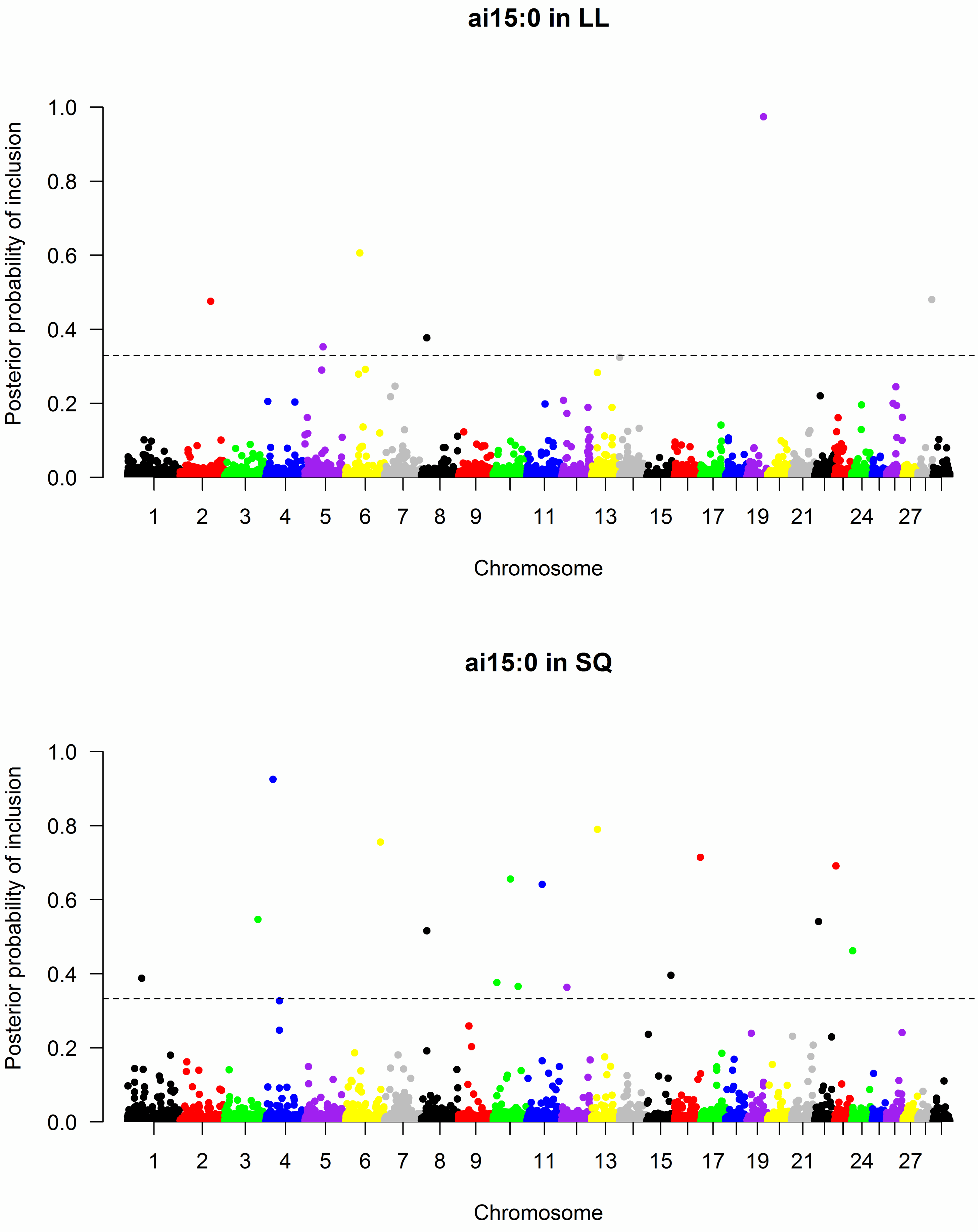
ai15:0 in LL
Posterior probability of inclusion
0.0
0.2
0.4
0.6
0.8
1.0
1
2
3
4
5
6
7
8
9
11
13
15
17
19
21
24
27
Chromosome
ai15:0 in SQ
Posterior probability of inclusion
0.0
0.2
0.4
0.6
0.8
1.0
1
2
3
4
5
6
7
8
9
11
13
15
17
19
21
24
27
Chromosome

iso16:0 in LL

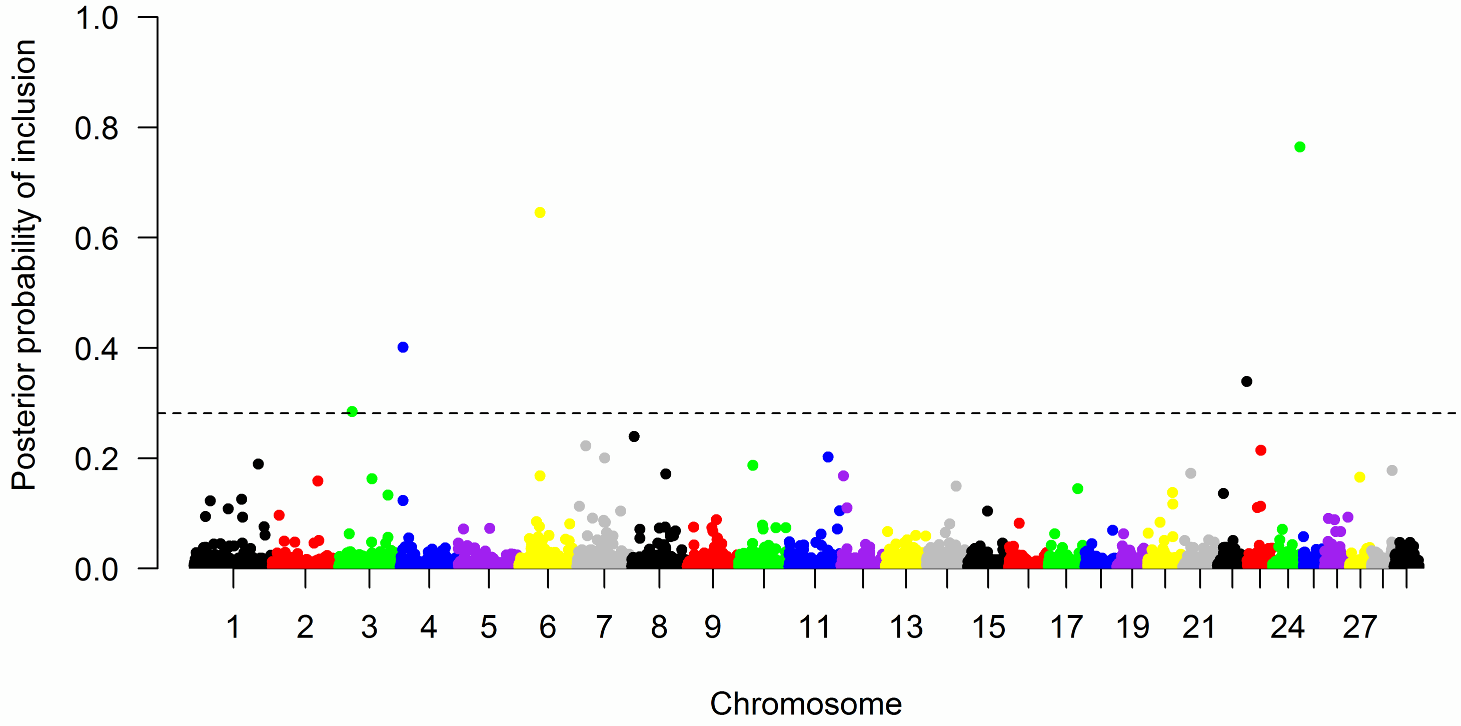

iso16:0 in SQ

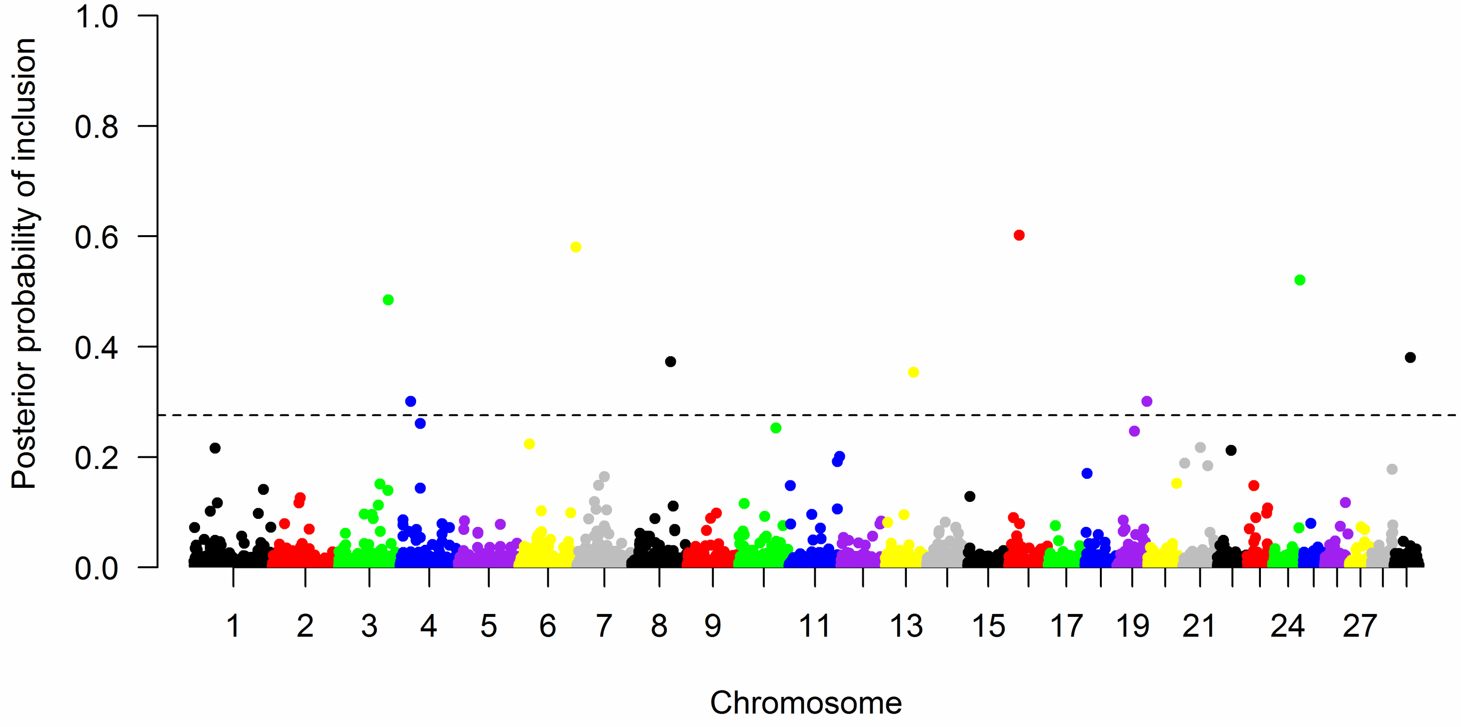

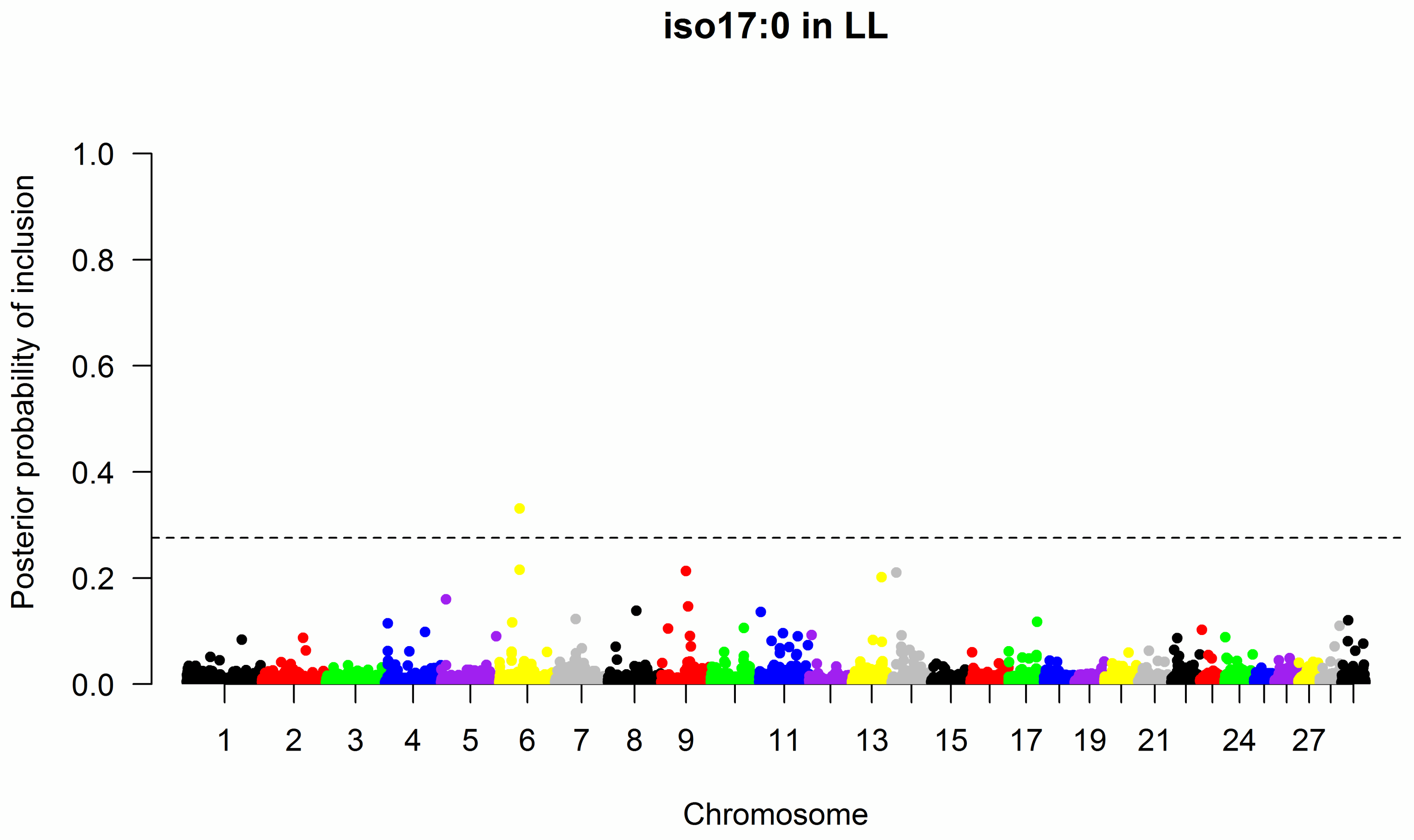
iso17:0 in LL
Posterior probability of inclusion
1.0
0.8
0.6
0.4
0.2
0.0
1
2
3
4
5
6
7
8
9
11
13
15
17
19
21
24
27
Chromosome

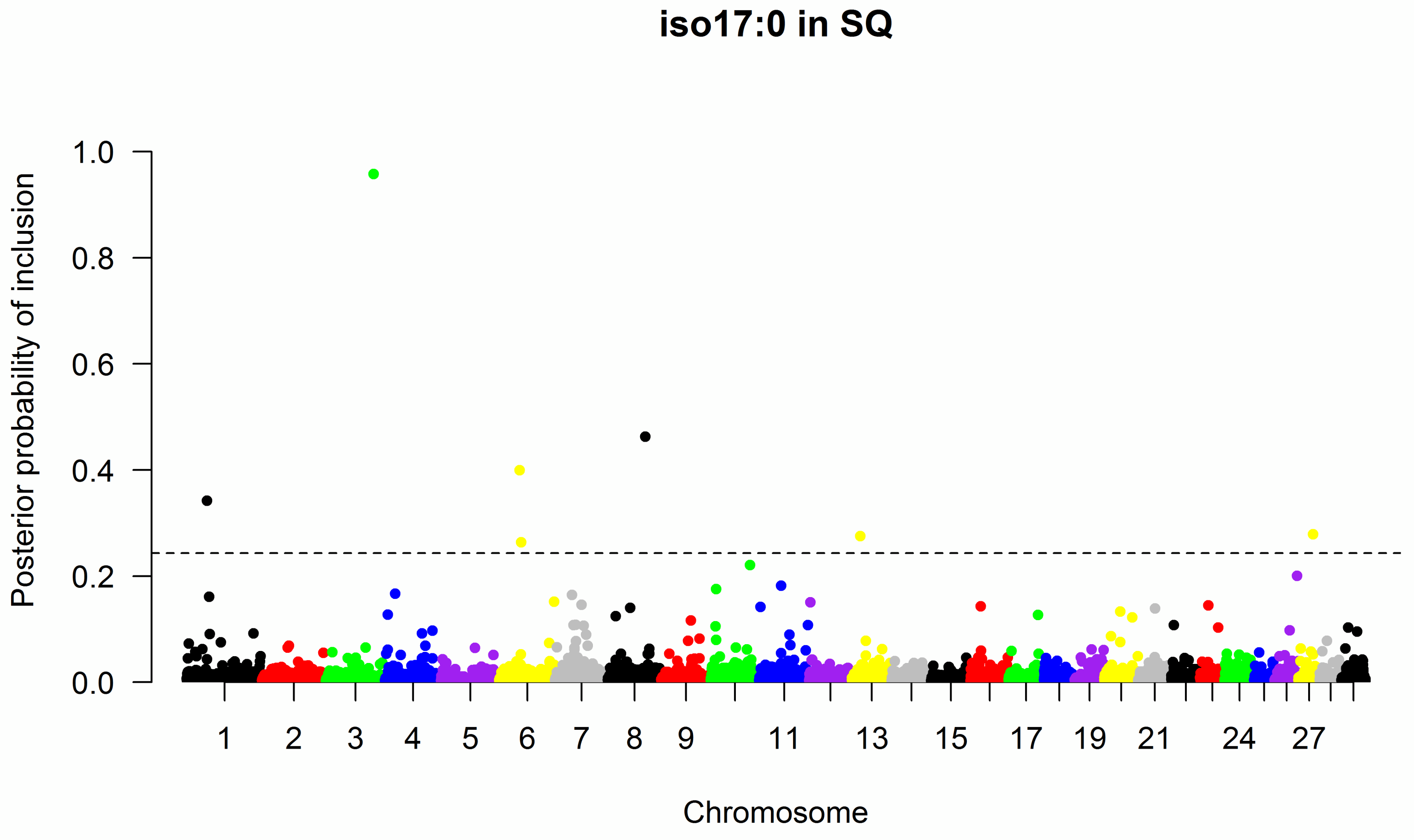
iso17:0 in SQ
Posterior probability of inclusion
1.0
0.8
0.6
0.4
0.2
0.0
1
2
3
4
5
6
7
8
9
11
13
15
17
19
21
24
27
Chromosome

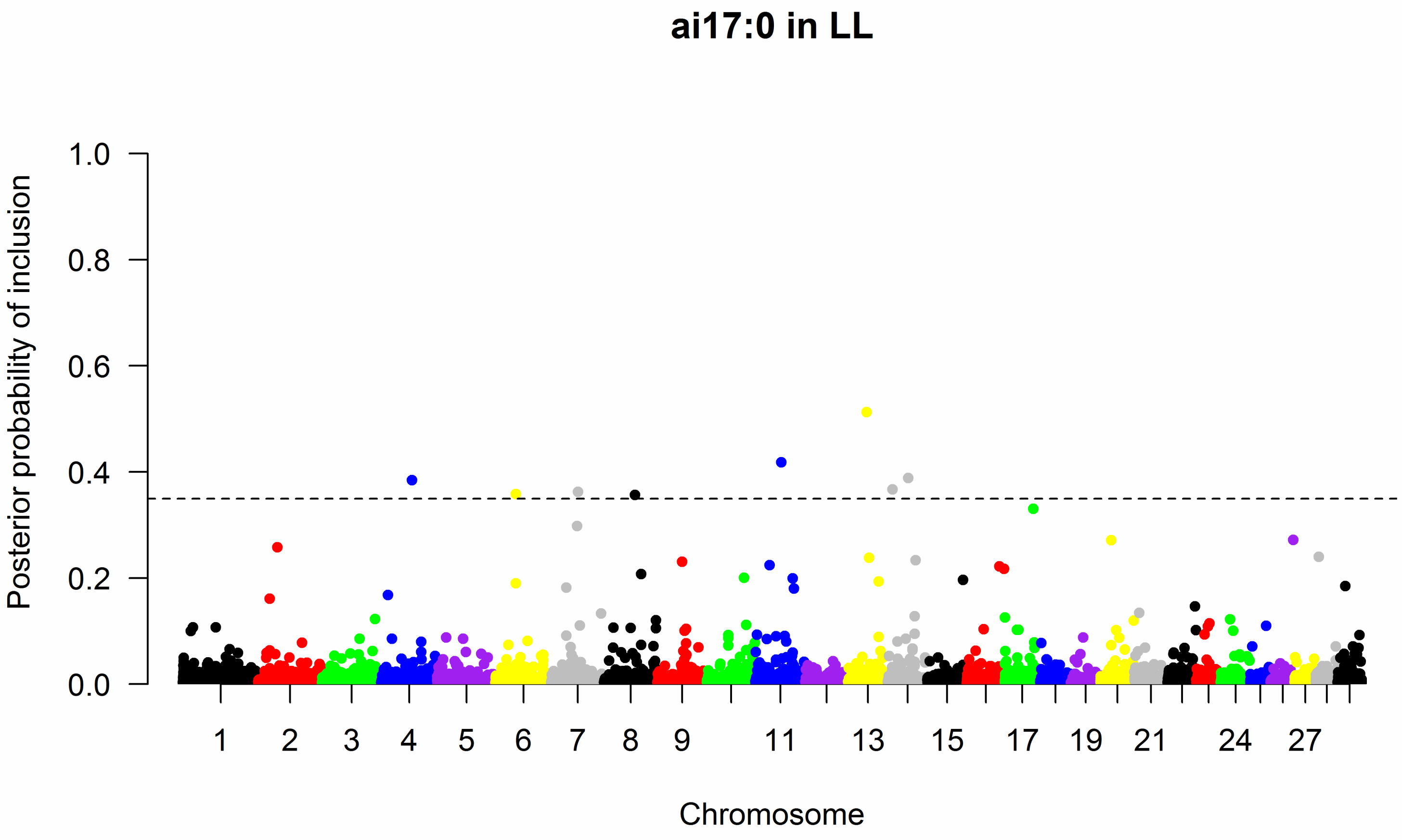
ai17:0 in LL
Posterior probability of inclusion
0.0
0.2
0.4
0.6
0.8
1.0
1 2 3 4 5 6 7 8 9 11 13 15 17 19 21 24 27
Chromosome

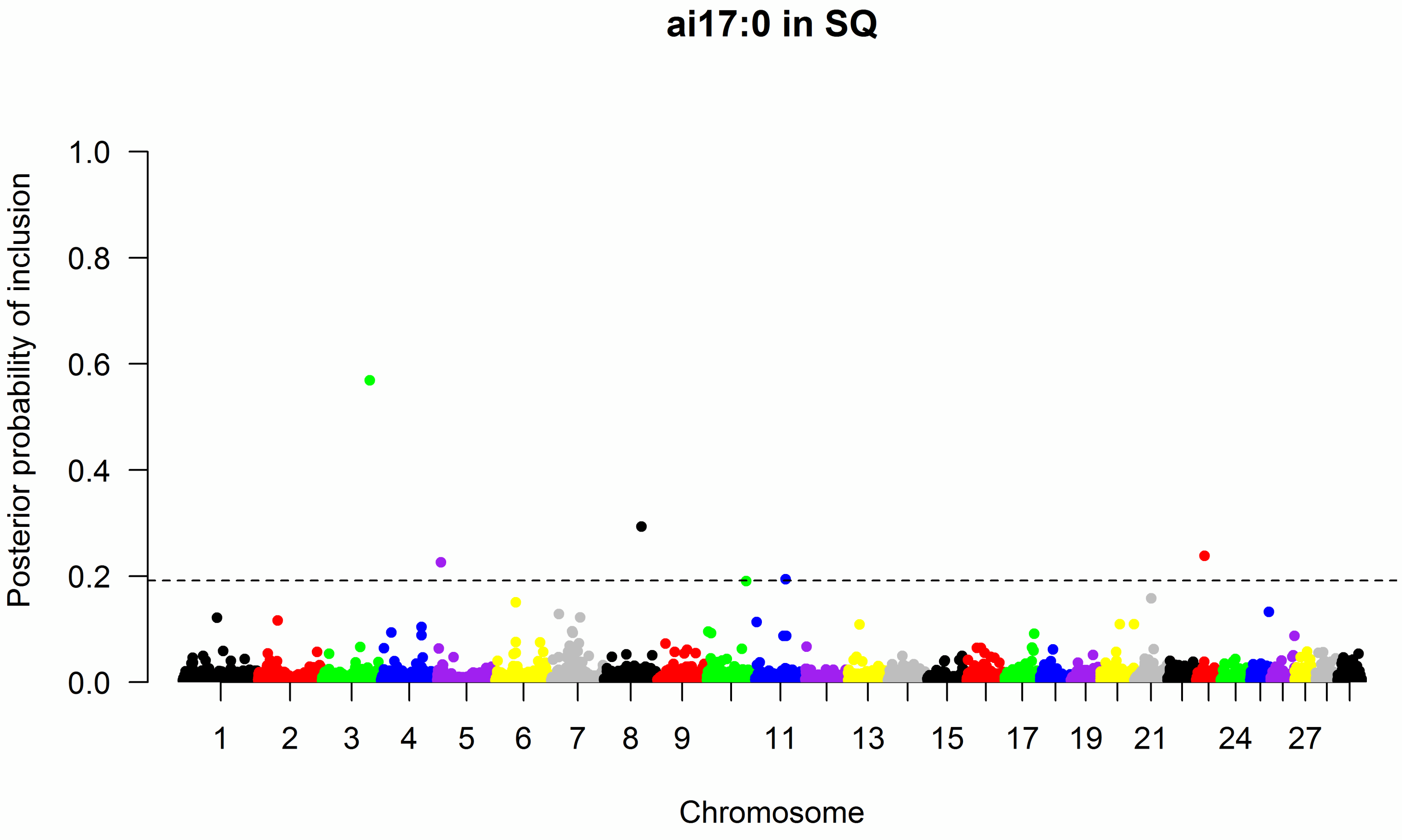
ai17:0 in SQ
Posterior probability of inclusion
0.0
0.2
0.4
0.6
0.8
1.0
1 2 3 4 5 6 7 8 9 11 13 15 17 19 21 24 27
Chromosome

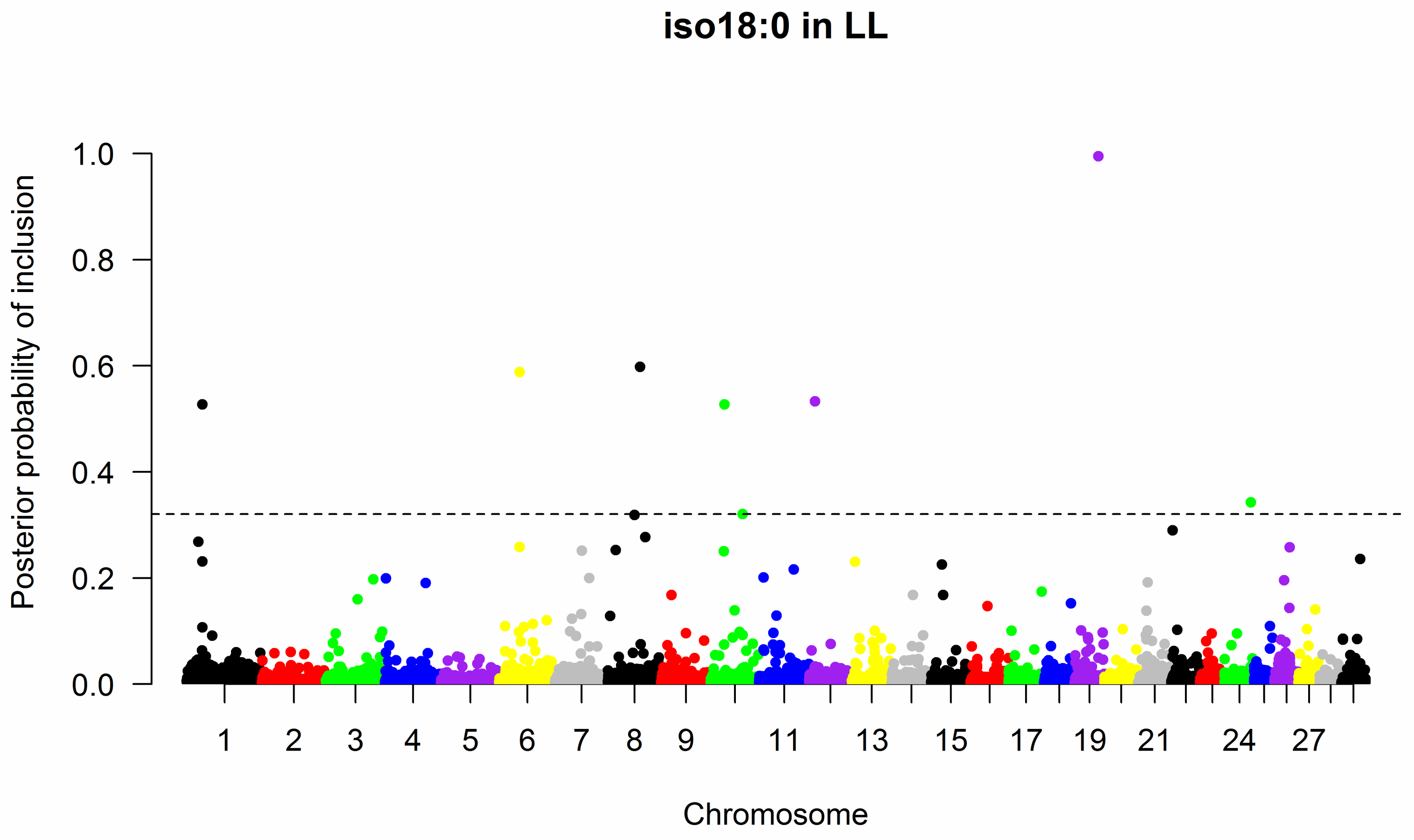
iso18:0 in LL
Posterior probability of inclusion
1.0
0.8
0.6
0.4
0.2
0.0
1
2
3
4
5
6
7
8
9
11
13
15
17
19
21
24
27
Chromosome

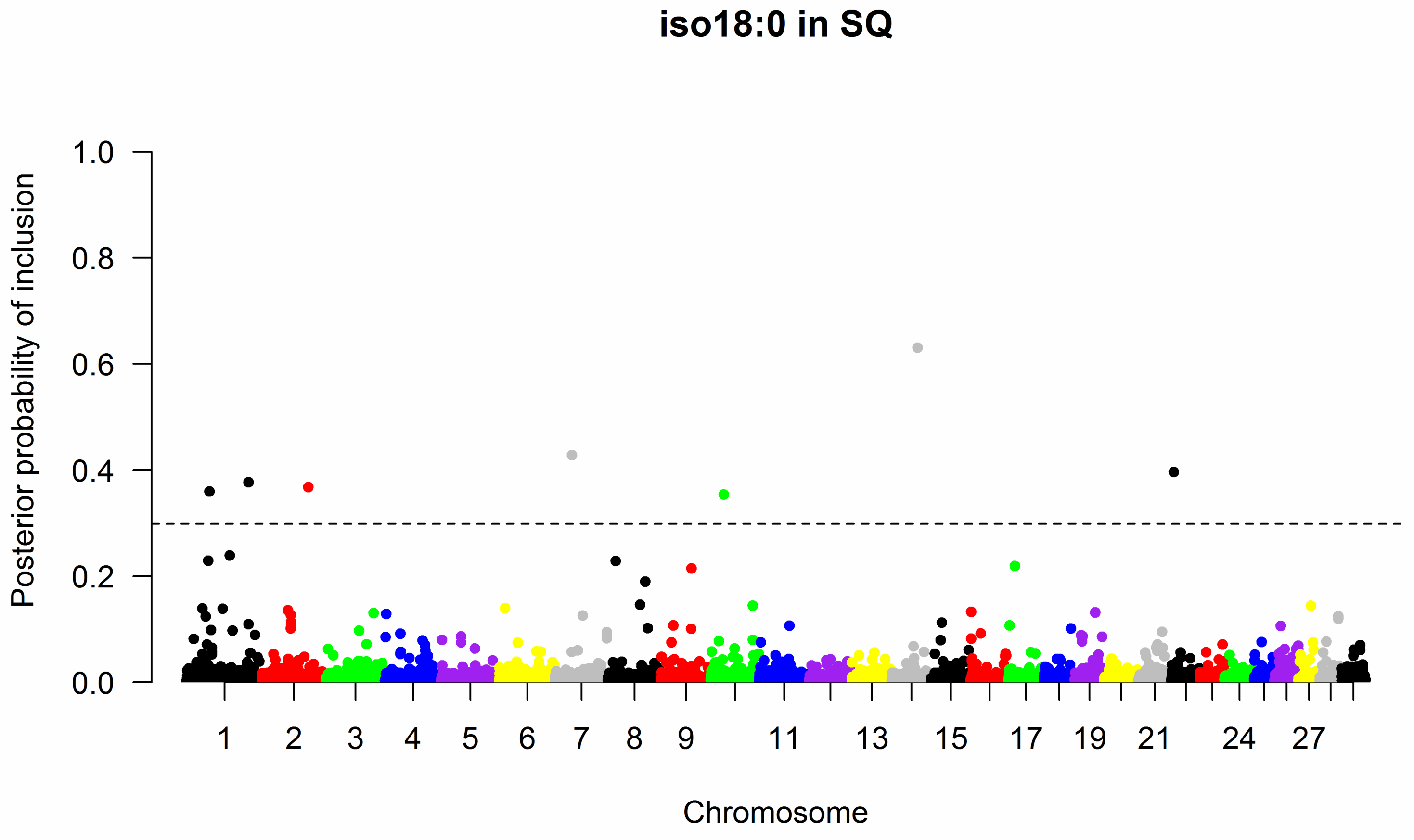
iso18:0 in SQ
Posterior probability of inclusion
1.0
0.8
0.6
0.4
0.2
0.0
1
2
3
4
5
6
7
8
9
11
13
15
17
19
21
24
27
Chromosome

BFA in LL

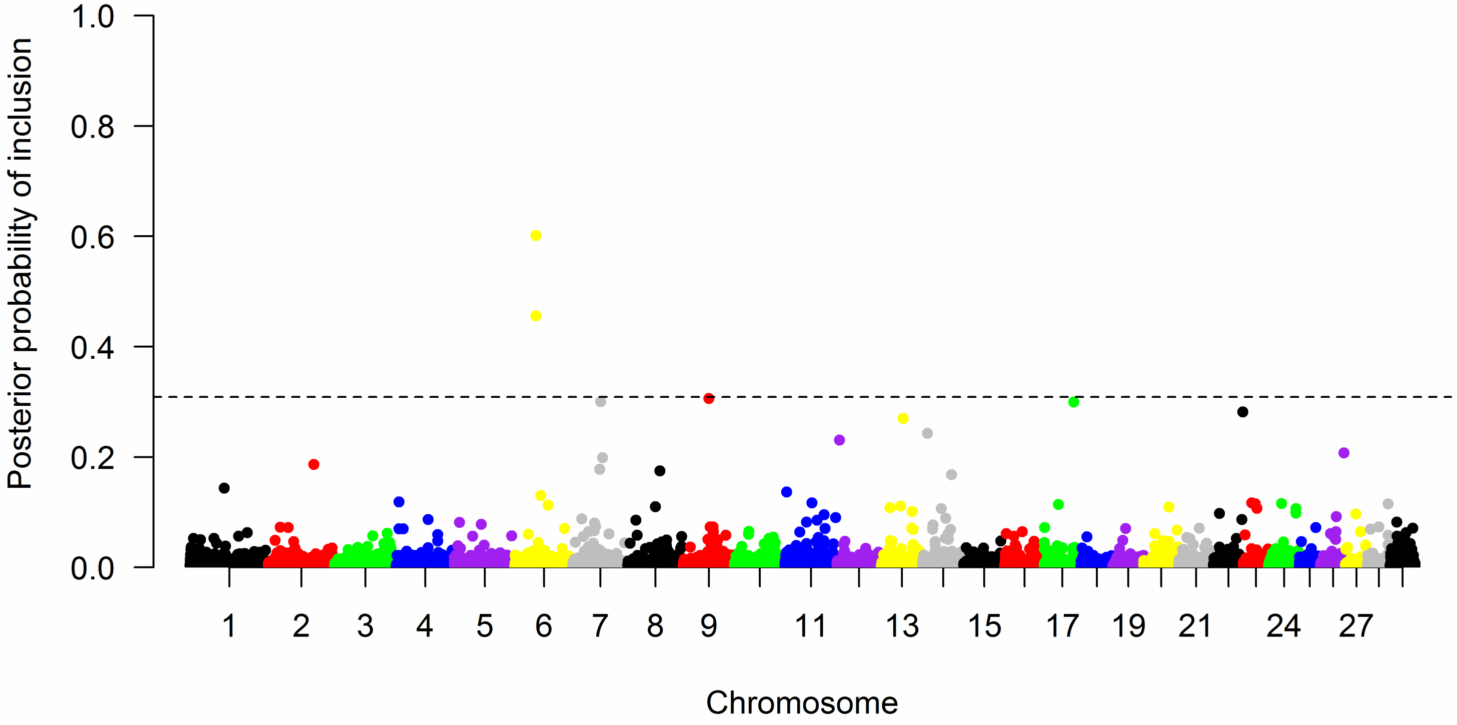

BFA in SQ

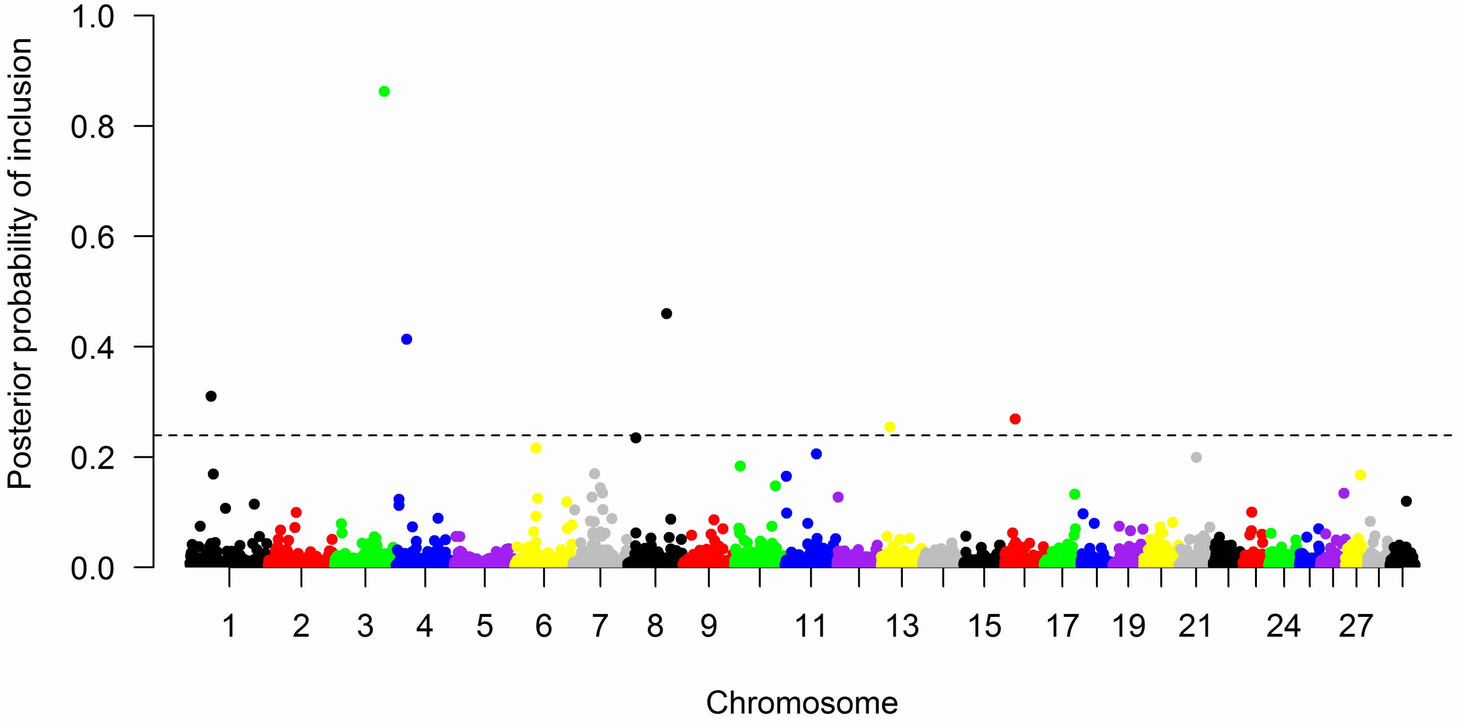

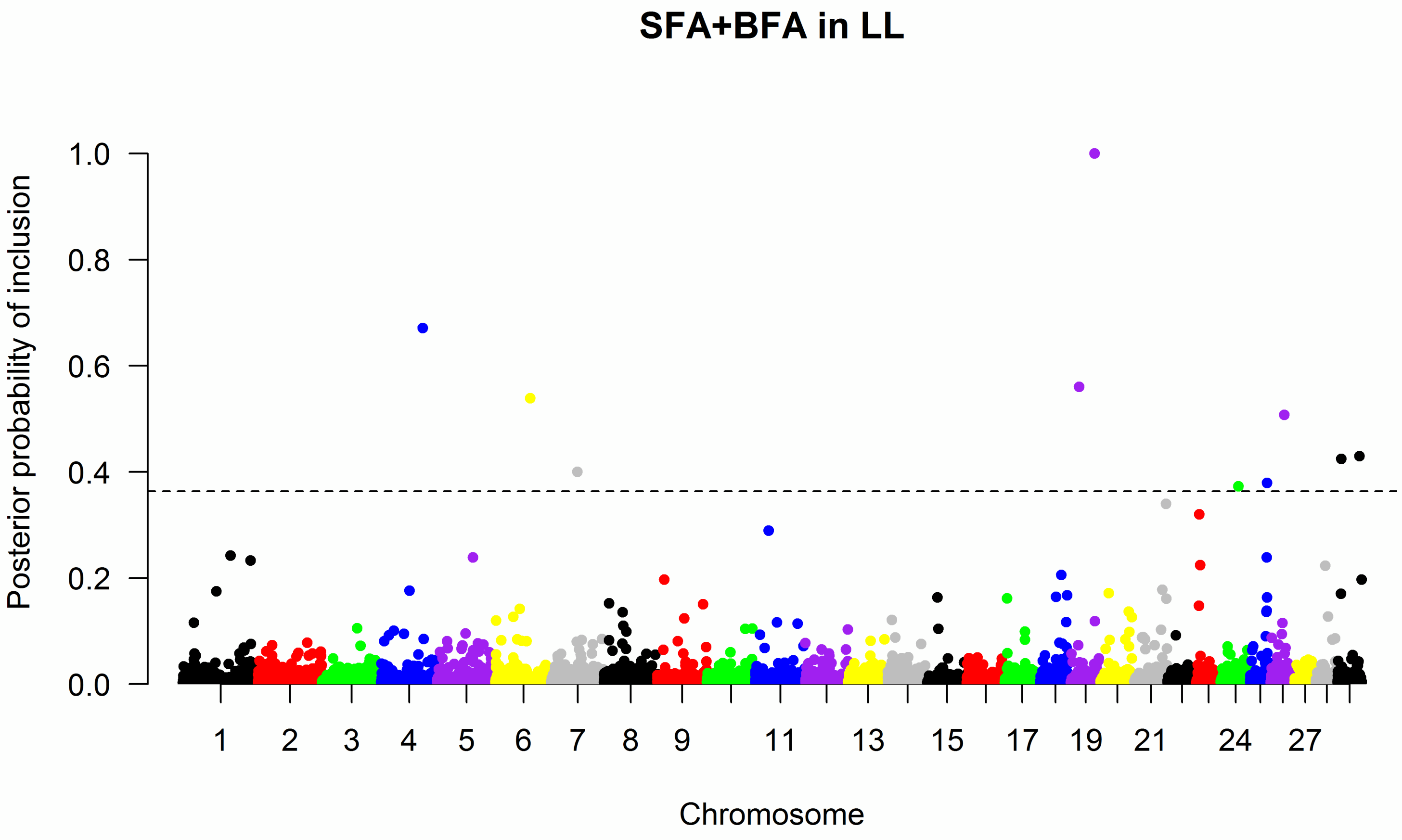
SFA+BFA in LL
Posterior probability of inclusion
0.0
0.2
0.4
0.6
0.8
1.0
Chromosome
1
2
3
4
5
6
7
8
9
11
13
15
17
19
21
24
27

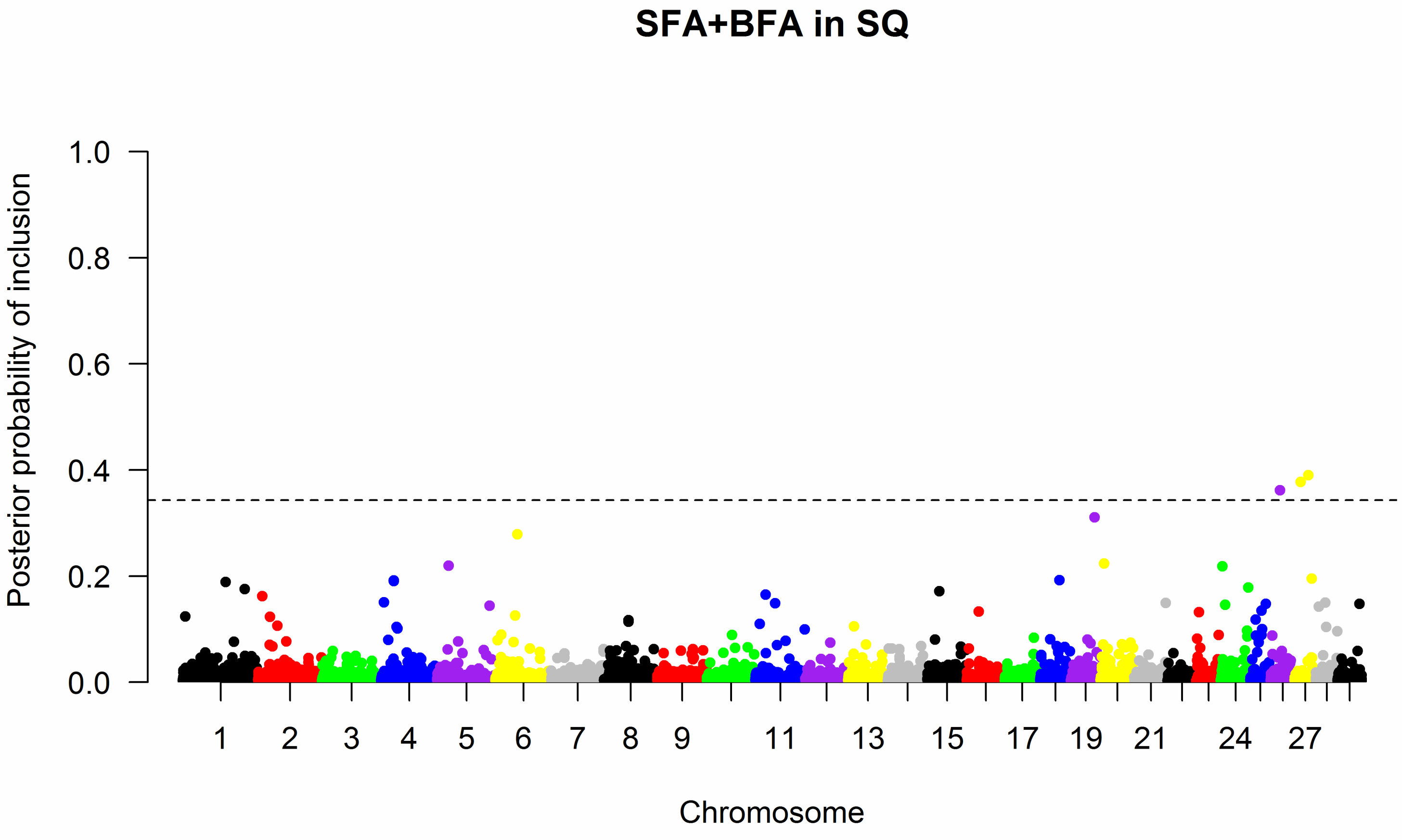
SFA+BFA in SQ
Posterior probability of inclusion
0.0
0.2
0.4
0.6
0.8
1.0
Chromosome
1
2
3
4
5
6
7
8
9
11
13
15
17
19
21
24
27

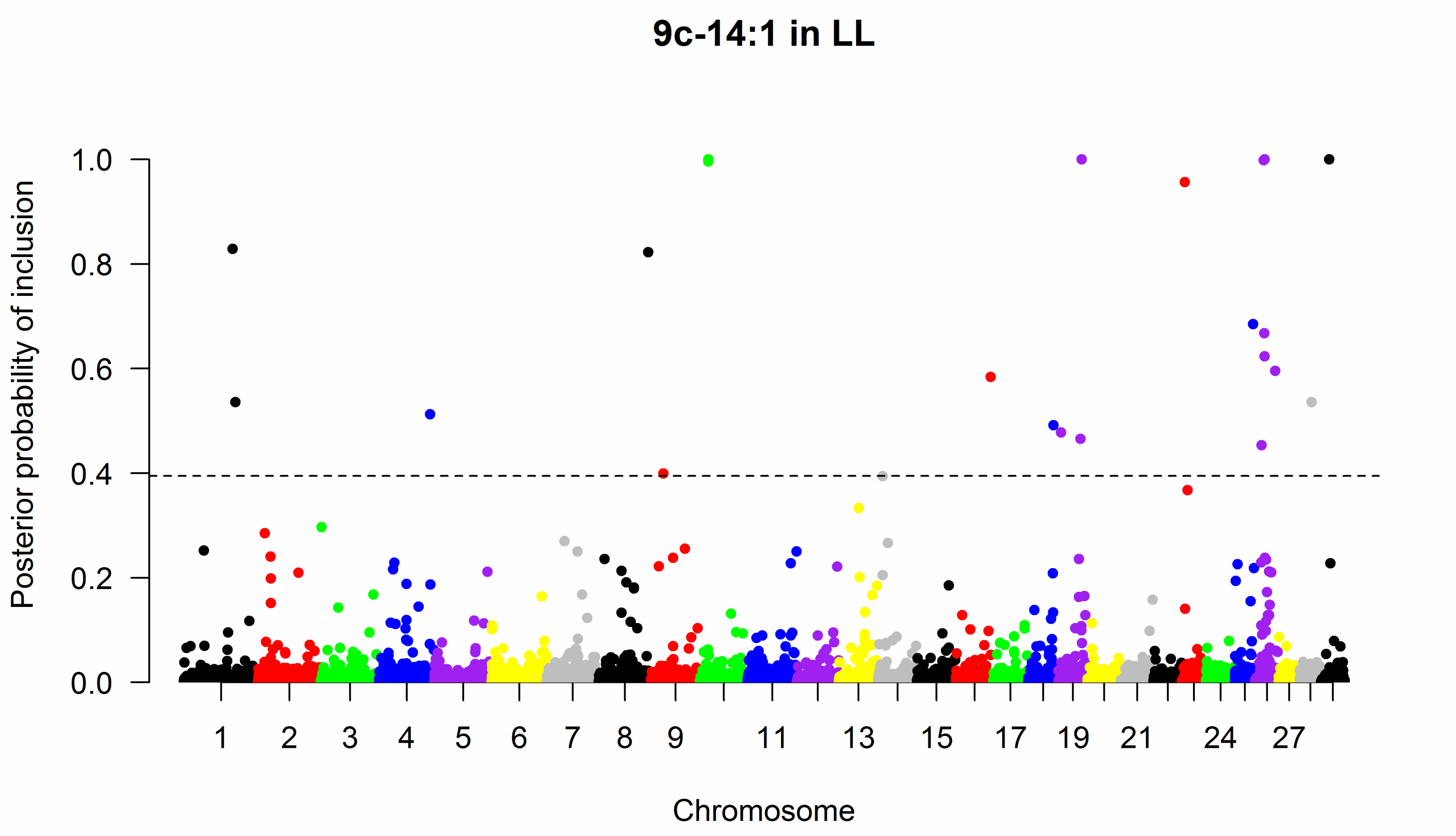

9c-14:1 in LL
Posterior probability of inclusion
0.0
0.2
0.4
0.6
0.8
1.0
1
2
3
4
5
6
7
8
9
11
13
15
17
19
21
24
27
Chromosome

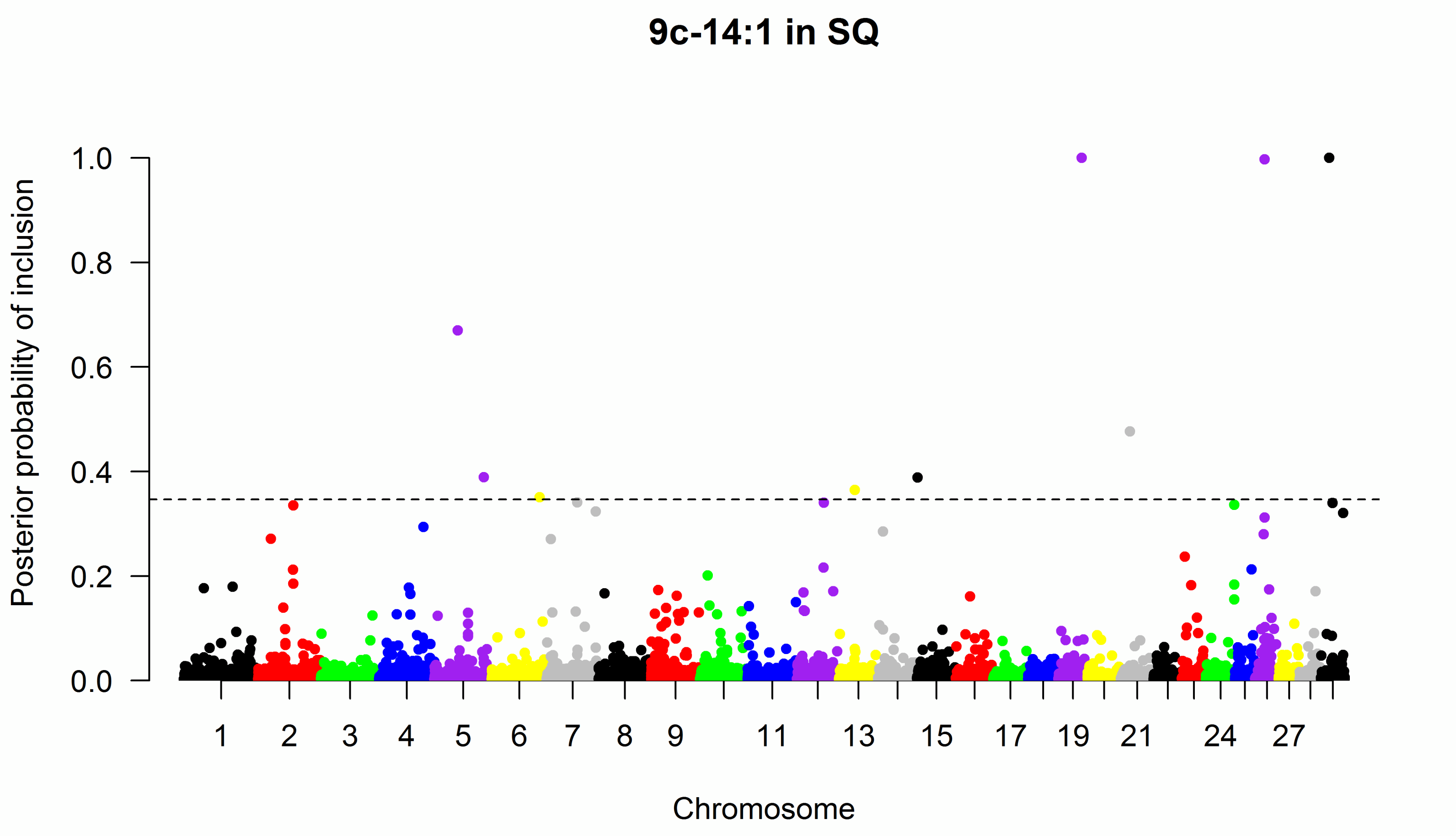

9c-14:1 in SQ
Posterior probability of inclusion
0.0
0.2
0.4
0.6
0.8
1.0
1
2
3
4
5
6
7
8
9
11
13
15
17
19
21
24
27
Chromosome

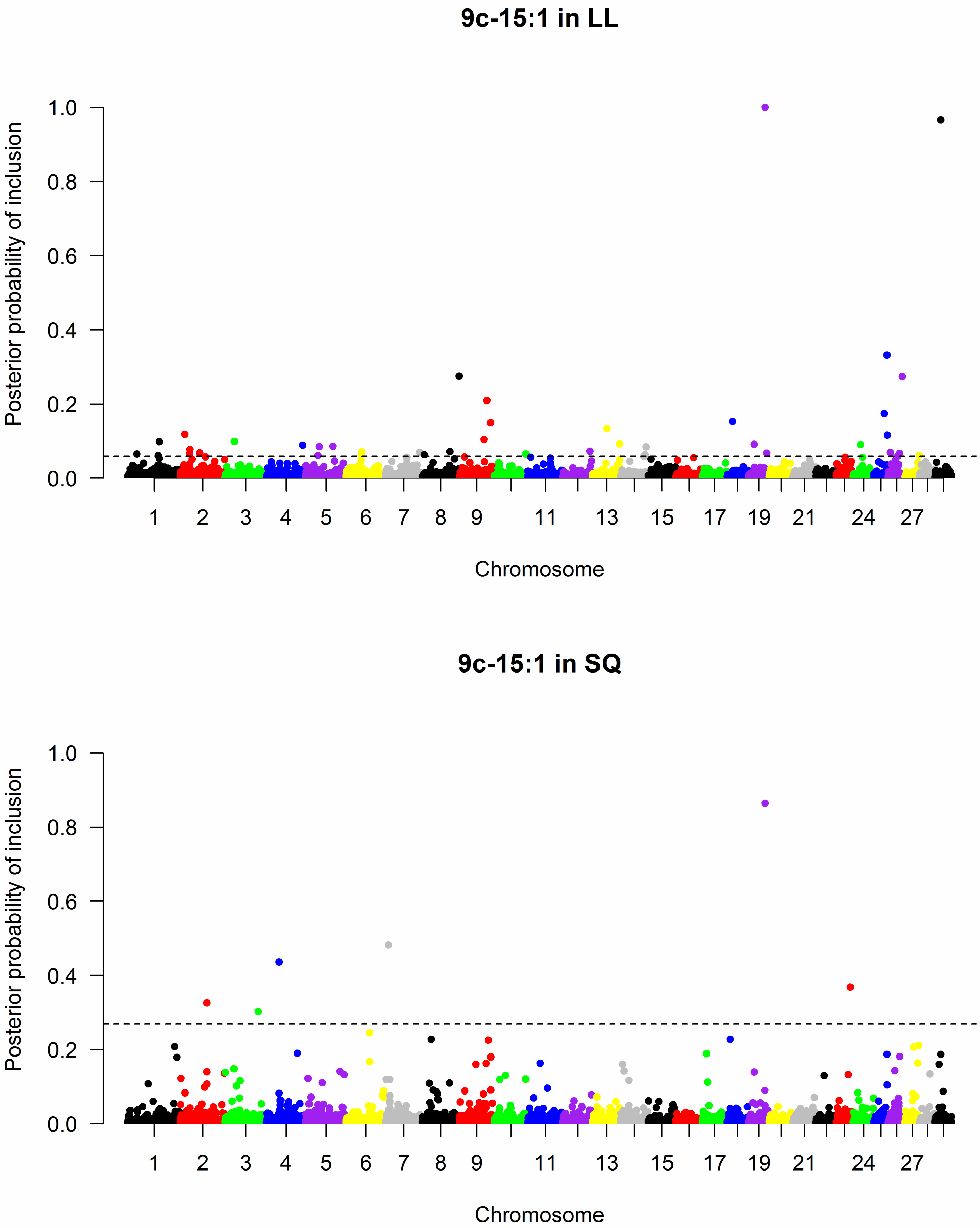
9c-15:1 in LL
Posterior probability of inclusion
1.0
0.8
0.6
0.4
0.2
0.0
1
2
3
4
5
6
7
8
9
11
13
15
17
19
21
24
27
Chromosome
9c-15:1 in SQ
Posterior probability of inclusion
1.0
0.8
0.6
0.4
0.2
0.0
1
2
3
4
5
6
7
8
9
11
13
15
17
19
21
24
27
Chromosome

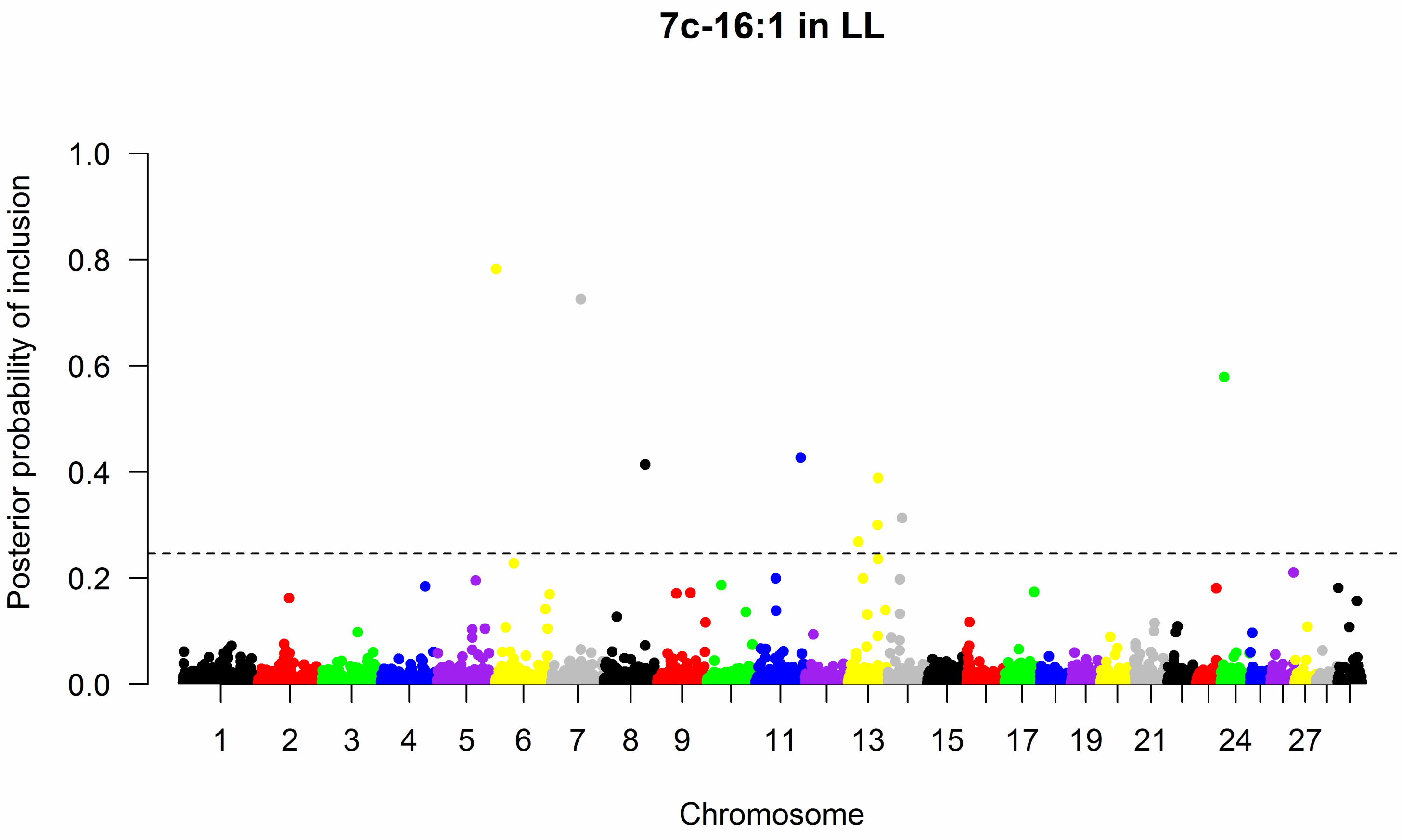
7c-16:1 in LL
Posterior probability of inclusion
1.0
0.8
0.6
0.4
0.2
0.0
1
2
3
4
5
6
7
8
9
11
13
15
17
19
21
24
27
Chromosome

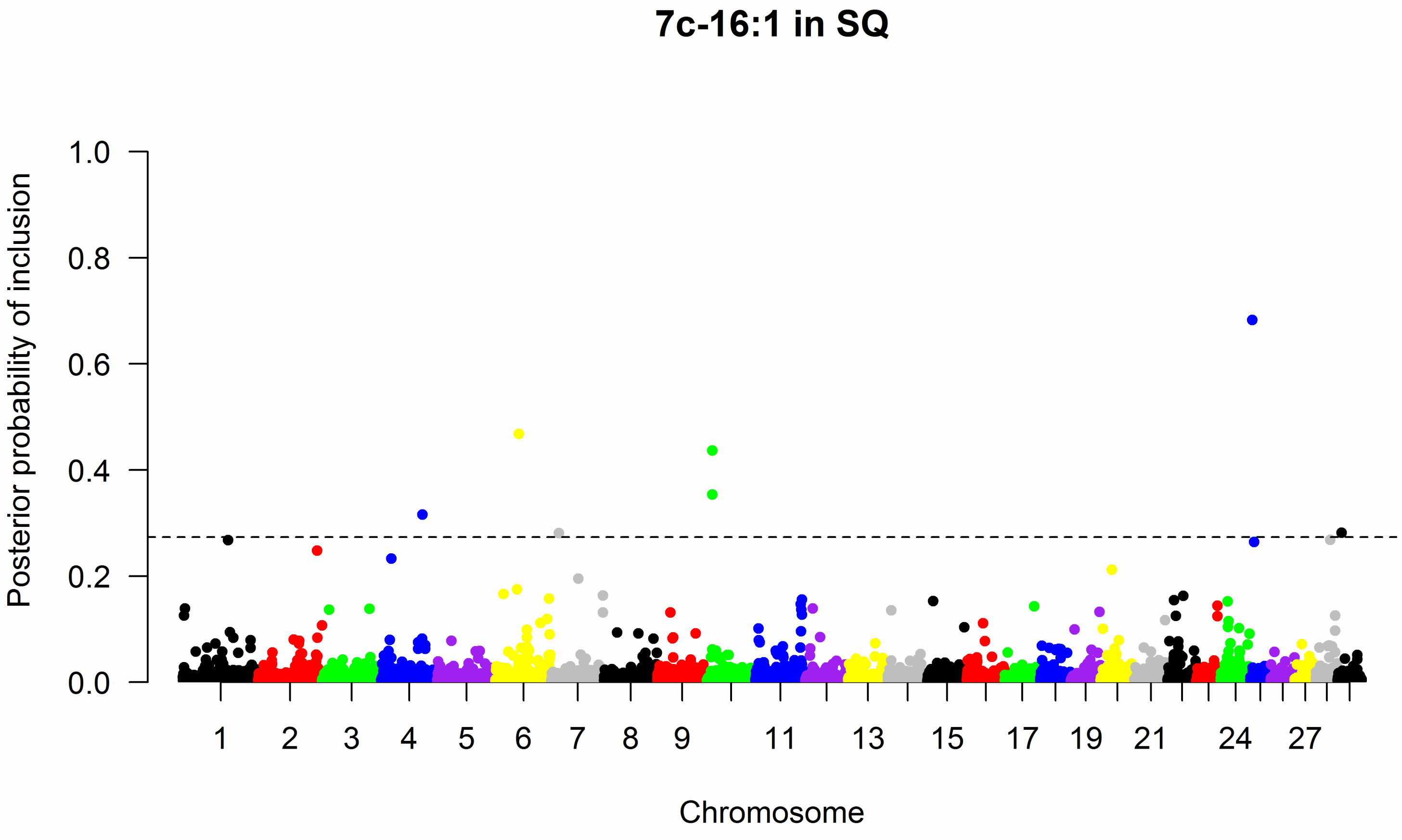
7c-16:1 in SQ
Posterior probability of inclusion
1.0
0.8
0.6
0.4
0.2
0.0
1
2
3
4
5
6
7
8
9
11
13
15
17
19
21
24
27
Chromosome

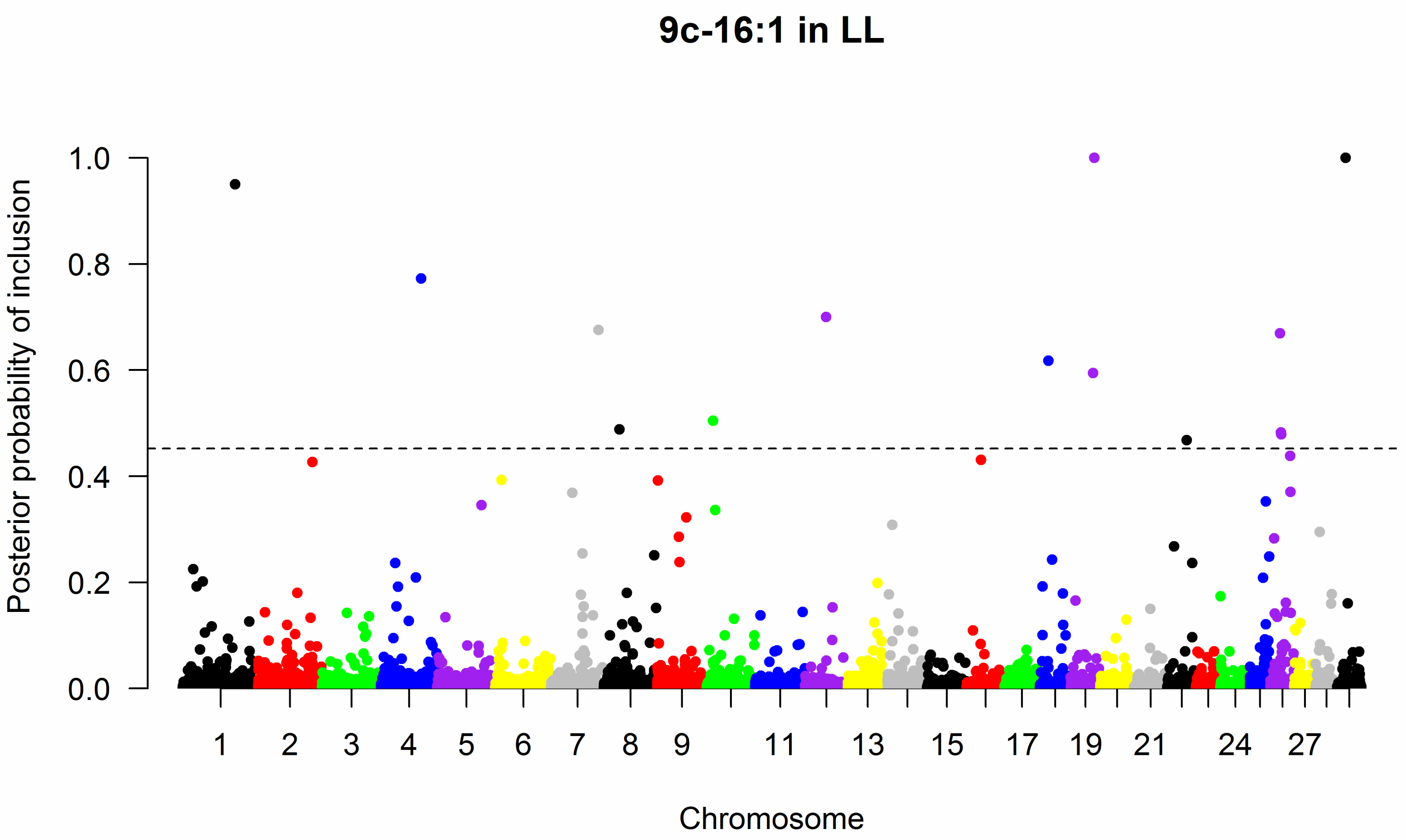
9c-16:1 in LL
Posterior probability of inclusion
1.0
0.8
0.6
0.4
0.2
0.0
1
2
3
4
5
6
7
8
9
11
13
15
17
19
21
24
27
Chromosome

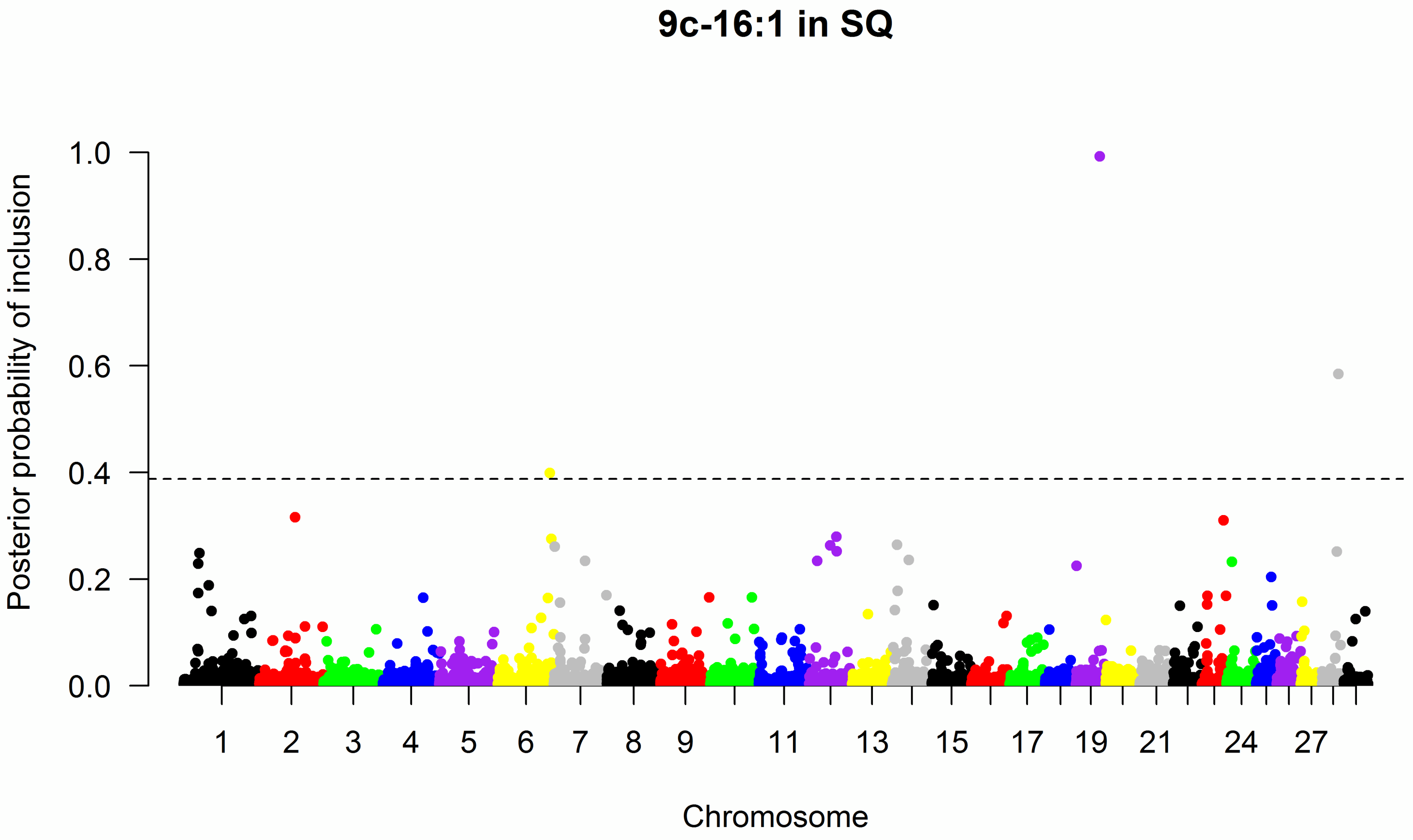
9c-16:1 in SQ
Posterior probability of inclusion
1.0
0.8
0.6
0.4
0.2
0.0
1
2
3
4
5
6
7
8
9
11
13
15
17
19
21
24
27
Chromosome

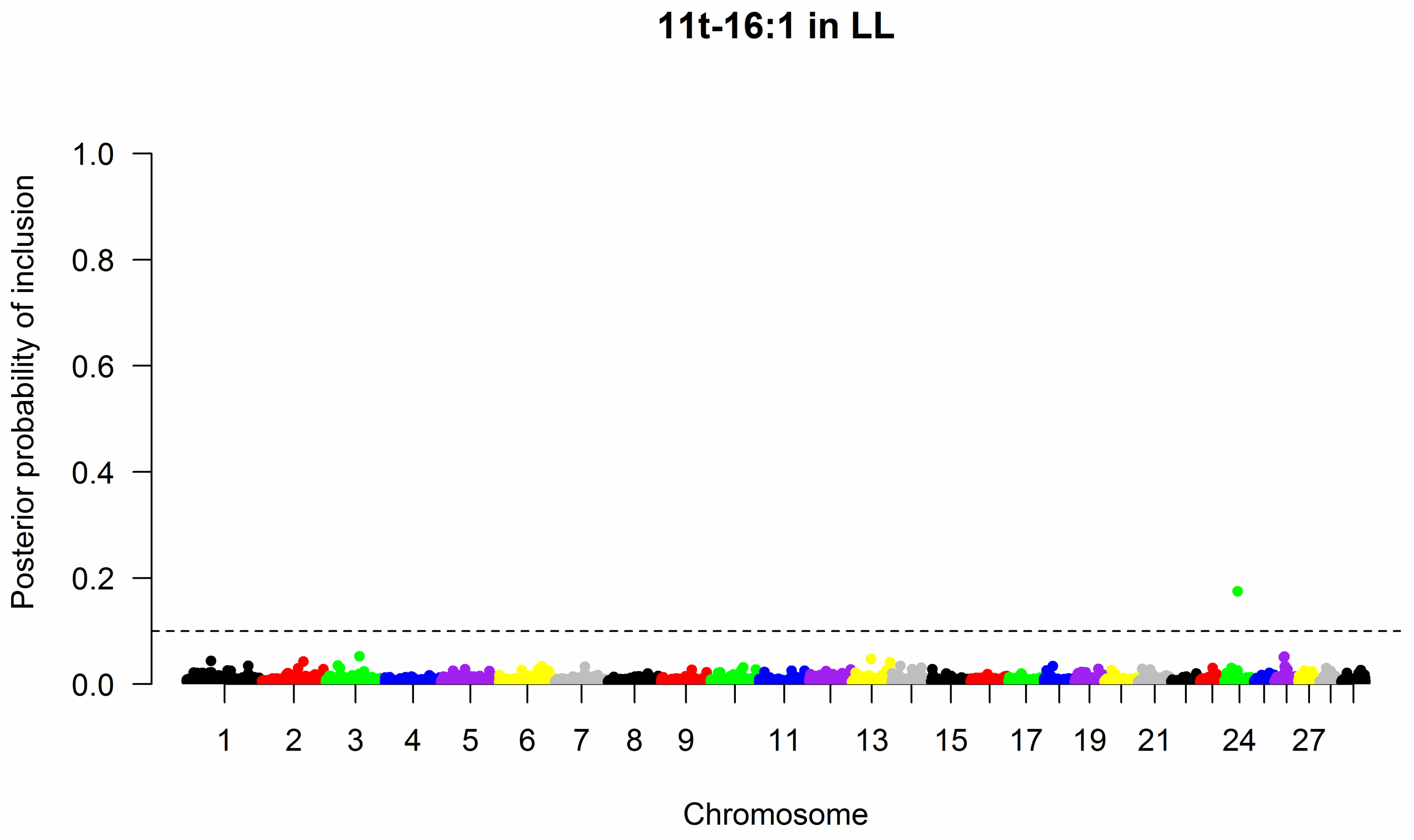
11t-16:1 in LL
Posterior probability of inclusion
0.0
0.2
0.4
0.6
0.8
1.0
1 2 3 4 5 6 7 8 9 11 13 15 17 19 21 24 27
Chromosome

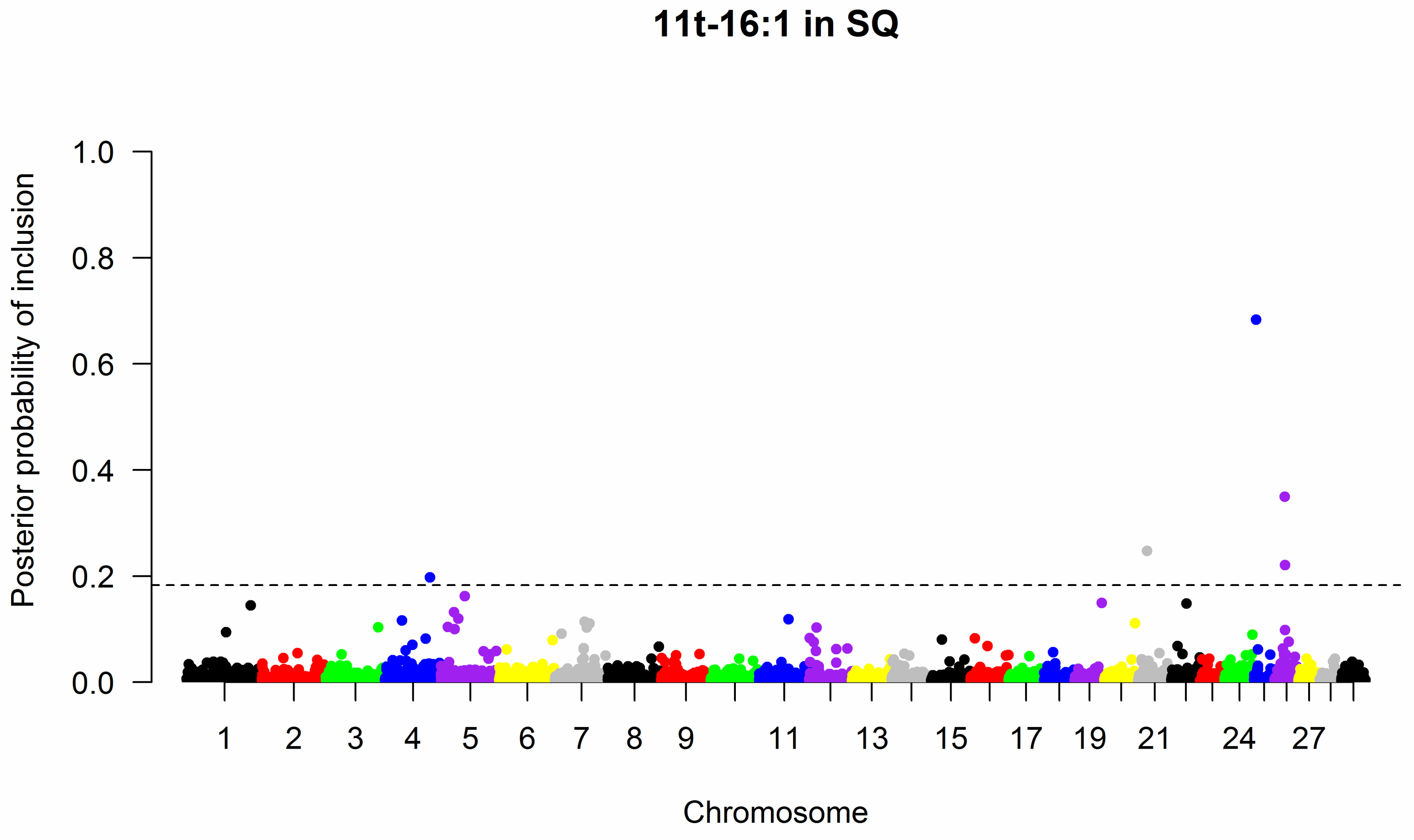
11t-16:1 in SQ
Posterior probability of inclusion
0.0
0.2
0.4
0.6
0.8
1.0
1 2 3 4 5 6 7 8 9 11 13 15 17 19 21 24 27
Chromosome

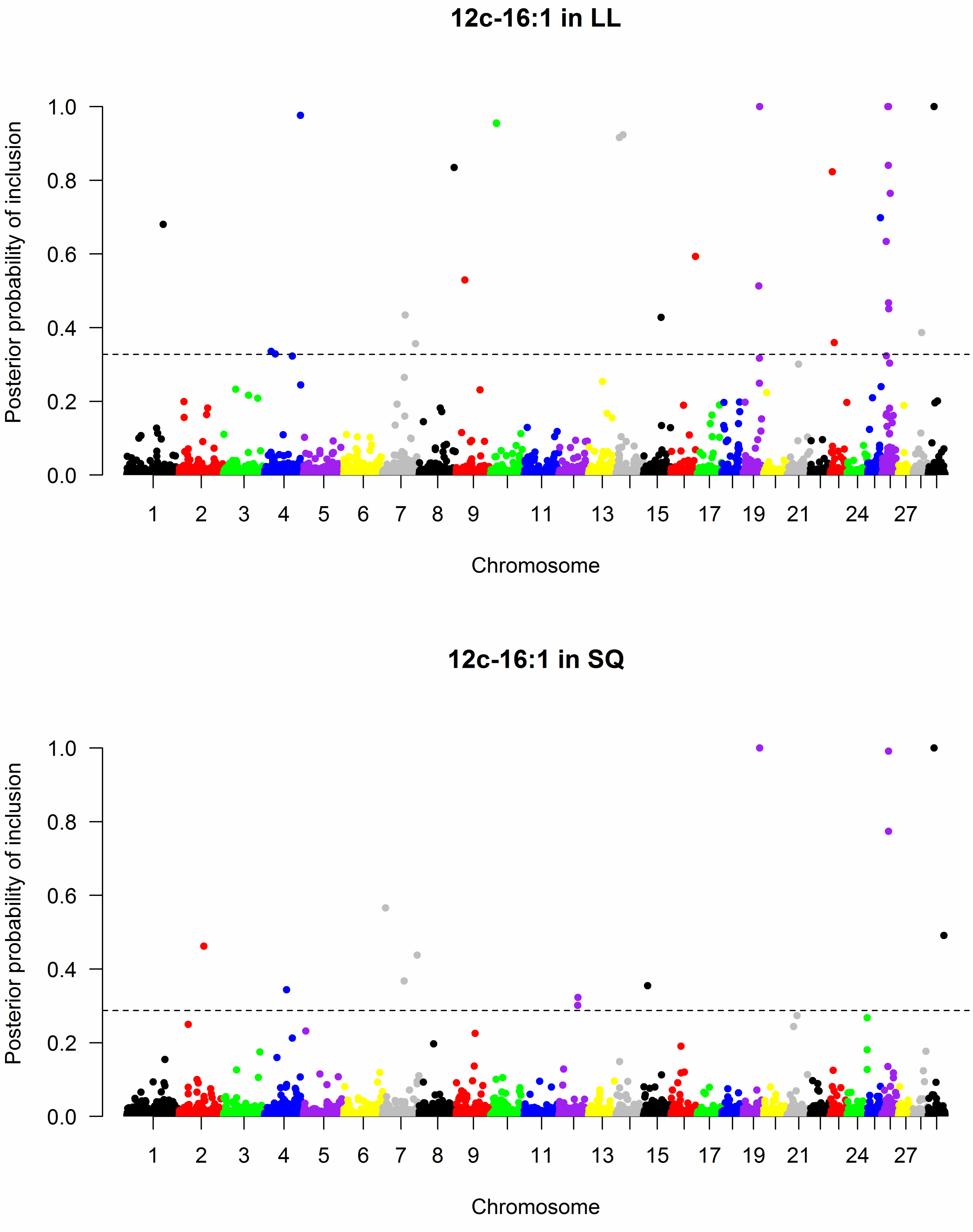

12c-16:1 in LL
Posterior probability of inclusion
0.0
0.2
0.4
0.6
0.8
1.0
1
2
3
4
5
6
7
8
9
11
13
15
17
19
21
24
27
Chromosome
12c-16:1 in SQ
Posterior probability of inclusion
0.0
0.2
0.4
0.6
0.8
1.0
1
2
3
4
5
6
7
8
9
11
13
15
17
19
21
24
27
Chromosome

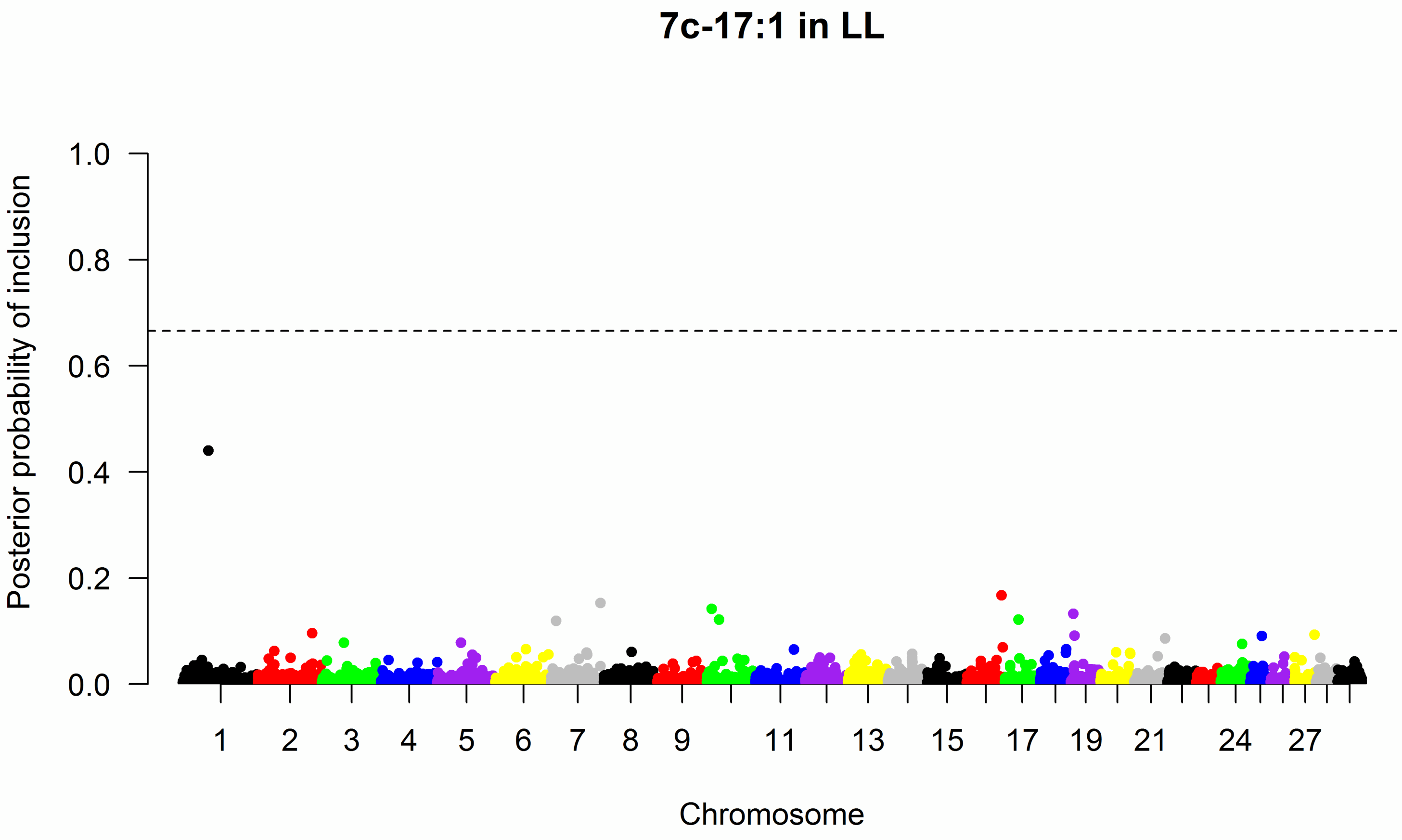
7c-17:1 in LL
Posterior probability of inclusion
1.0
0.8
0.6
0.4
0.2
0.0
1
2
3
4
5
6
7
8
9
11
13
15
17
19
21
24
27
Chromosome

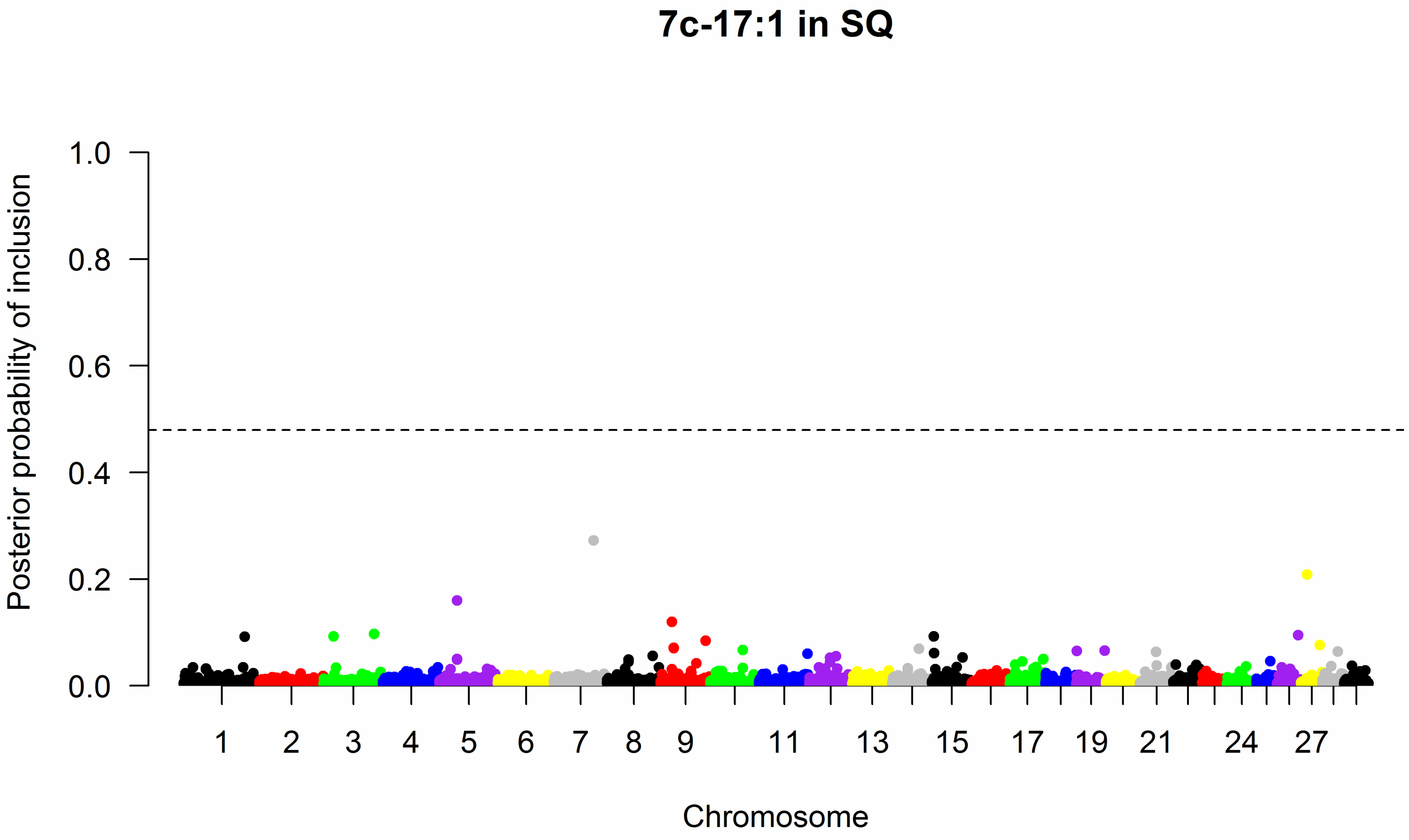
7c-17:1 in SQ
Posterior probability of inclusion
1.0
0.8
0.6
0.4
0.2
0.0
1
2
3
4
5
6
7
8
9
11
13
15
17
19
21
24
27
Chromosome

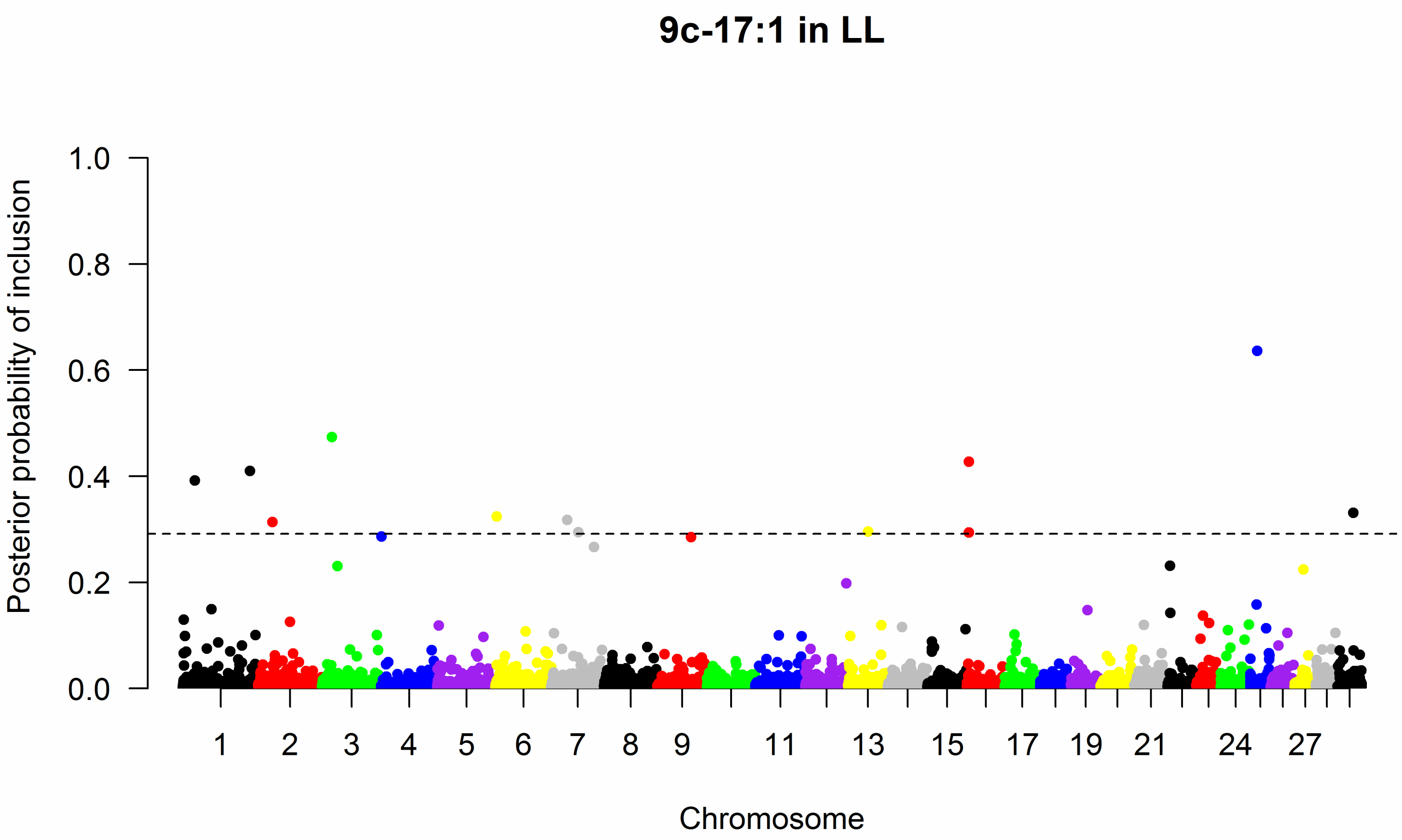
9c-17:1 in LL
Posterior probability of inclusion
1.0
0.8
0.6
0.4
0.2
0.0
1
2
3
4
5
6
7
8
9
11
13
15
17
19
21
24
27
Chromosome

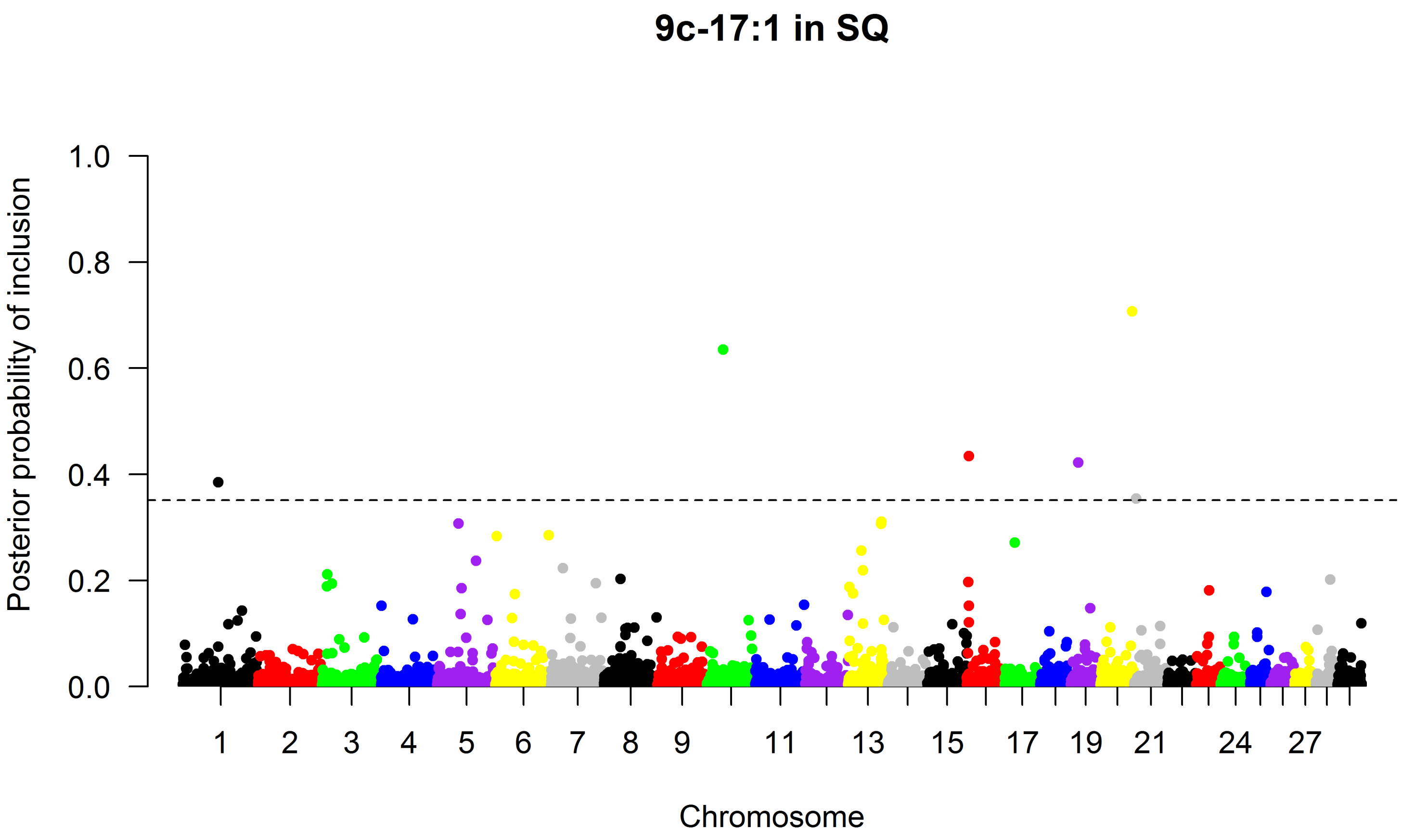
9c-17:1 in SQ
Posterior probability of inclusion
1.0
0.8
0.6
0.4
0.2
0.0
1
2
3
4
5
6
7
8
9
11
13
15
17
19
21
24
27
Chromosome

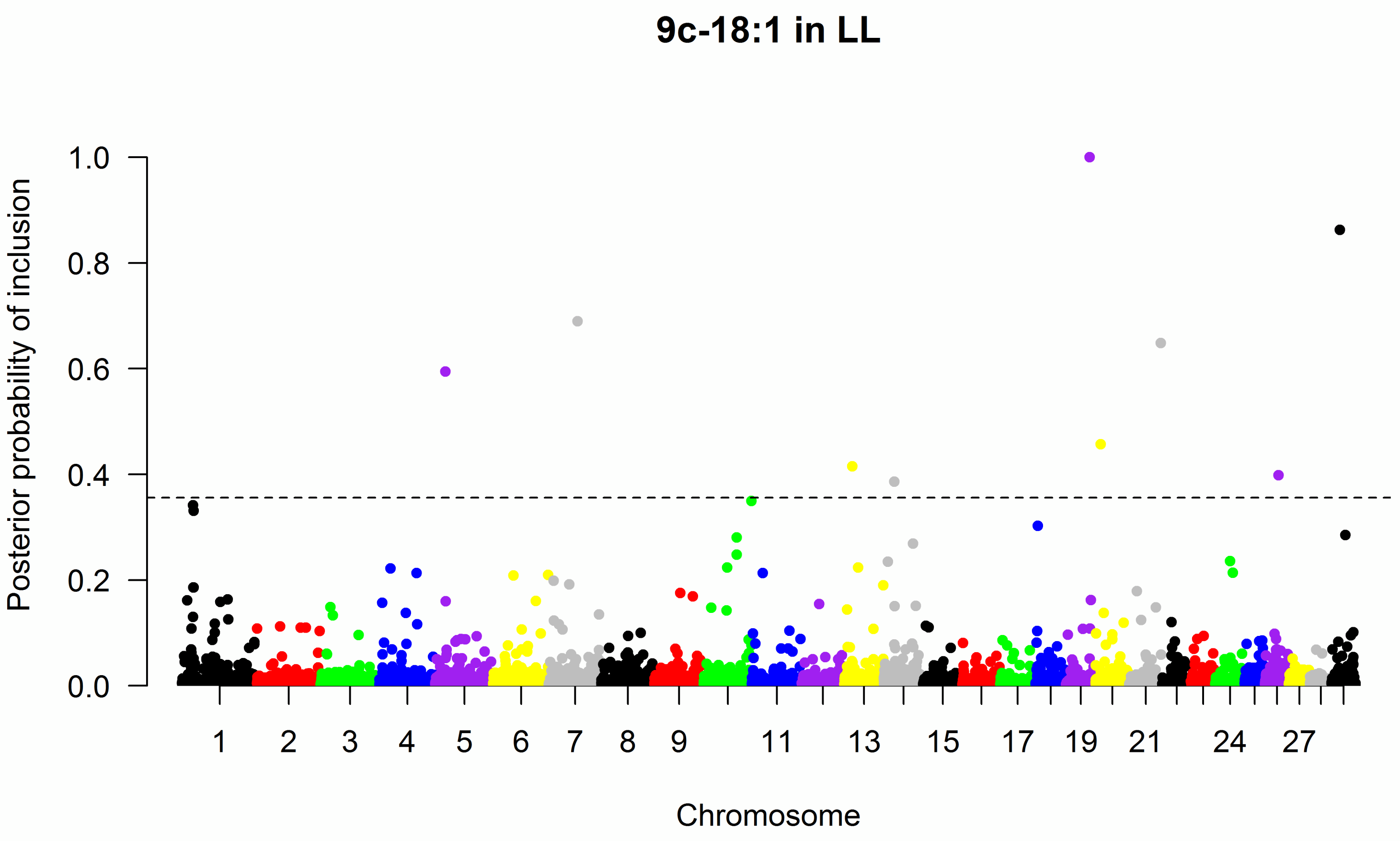
9c-18:1 in LL
Posterior probability of inclusion
1.0
0.8
0.6
0.4
0.2
0.0
1
2
3
4
5
6
7
8
9
11
13
15
17
19
21
24
27
Chromosome

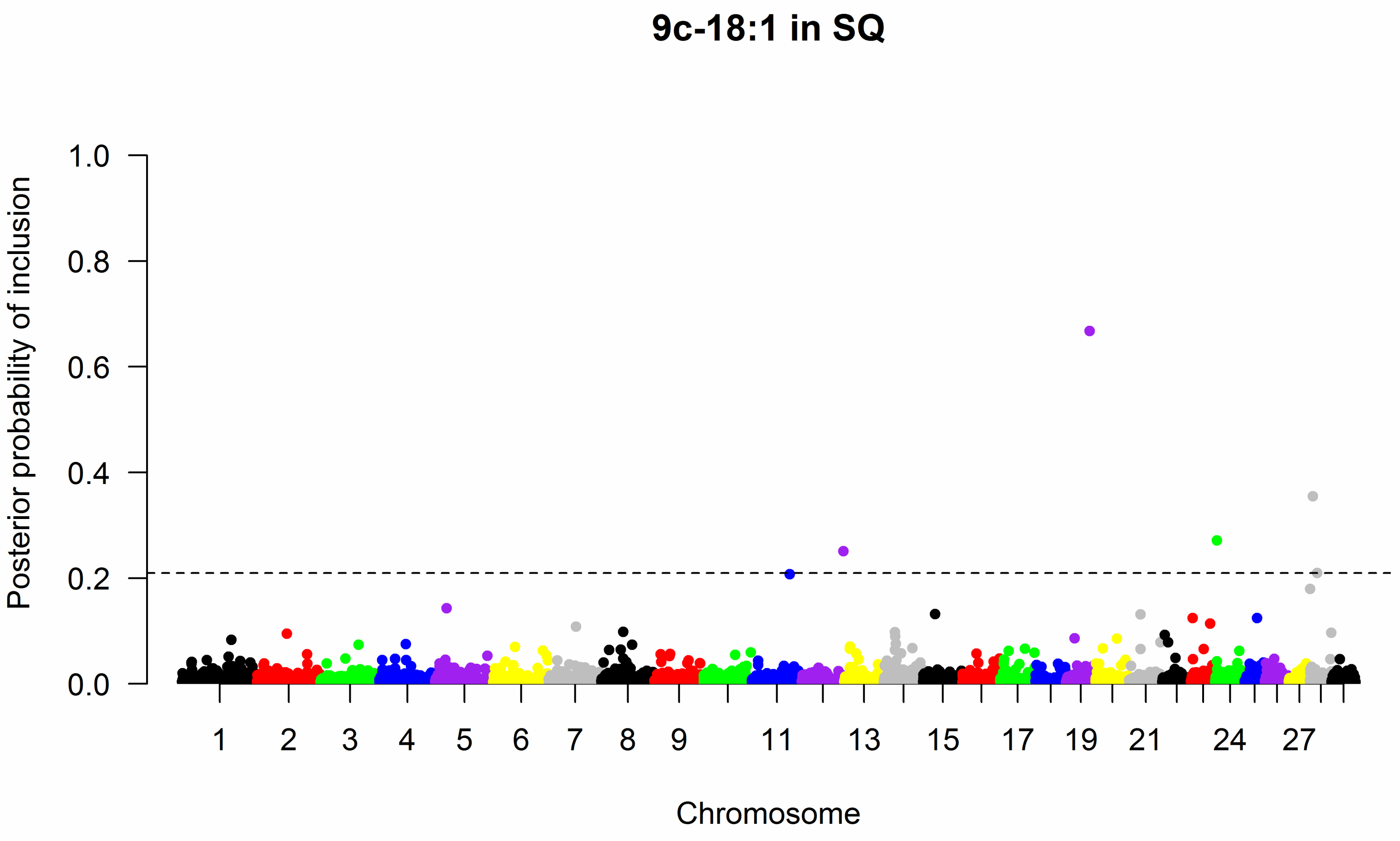
9c-18:1 in SQ
Posterior probability of inclusion
1.0
0.8
0.6
0.4
0.2
0.0
1
2
3
4
5
6
7
8
9
11
13
15
17
19
21
24
27
Chromosome

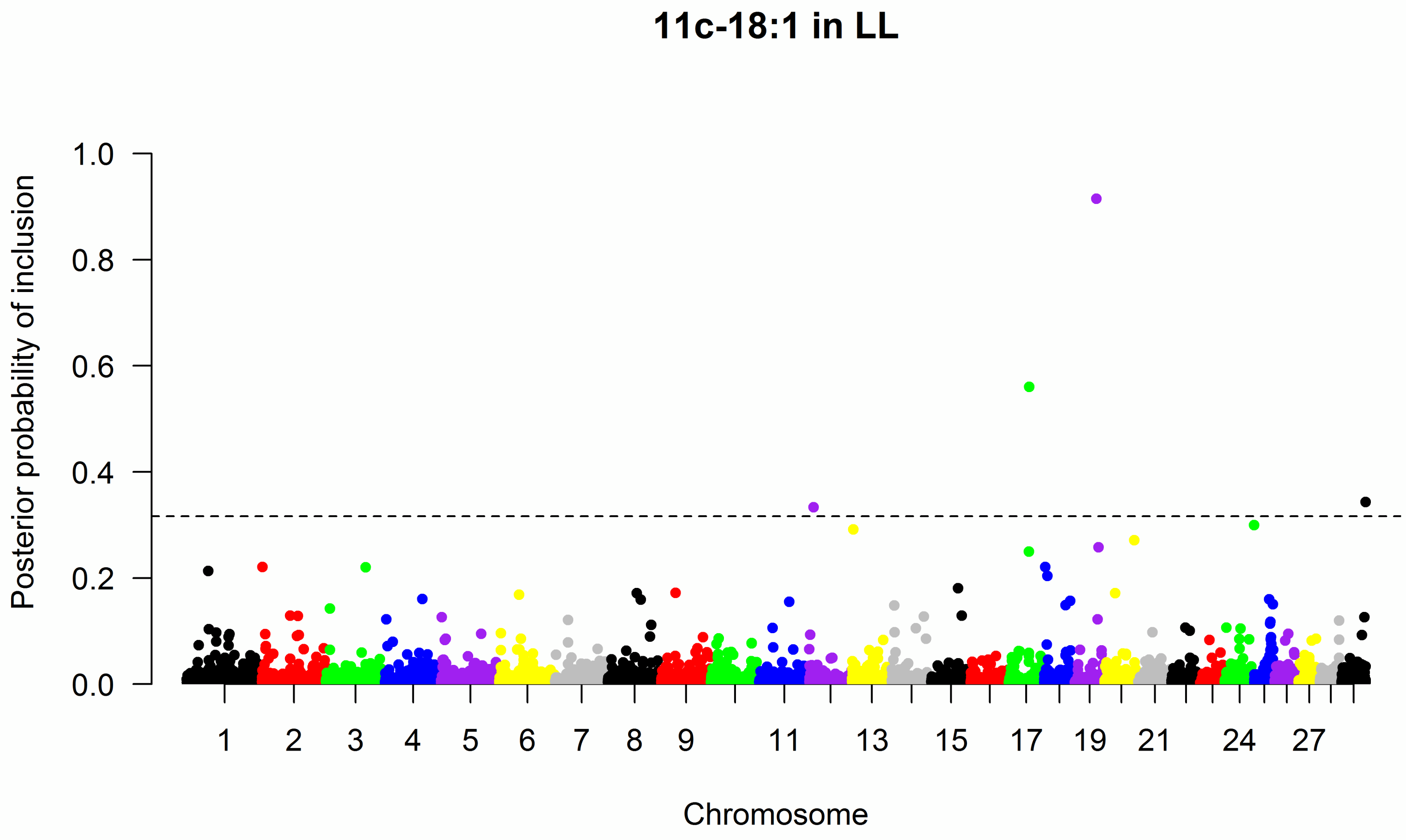
11c-18:1 in LL
Posterior probability of inclusion
1.0
0.8
0.6
0.4
0.2
0.0
1
2
3
4
5
6
7
8
9
11
13
15
17
19
21
24
27
Chromosome

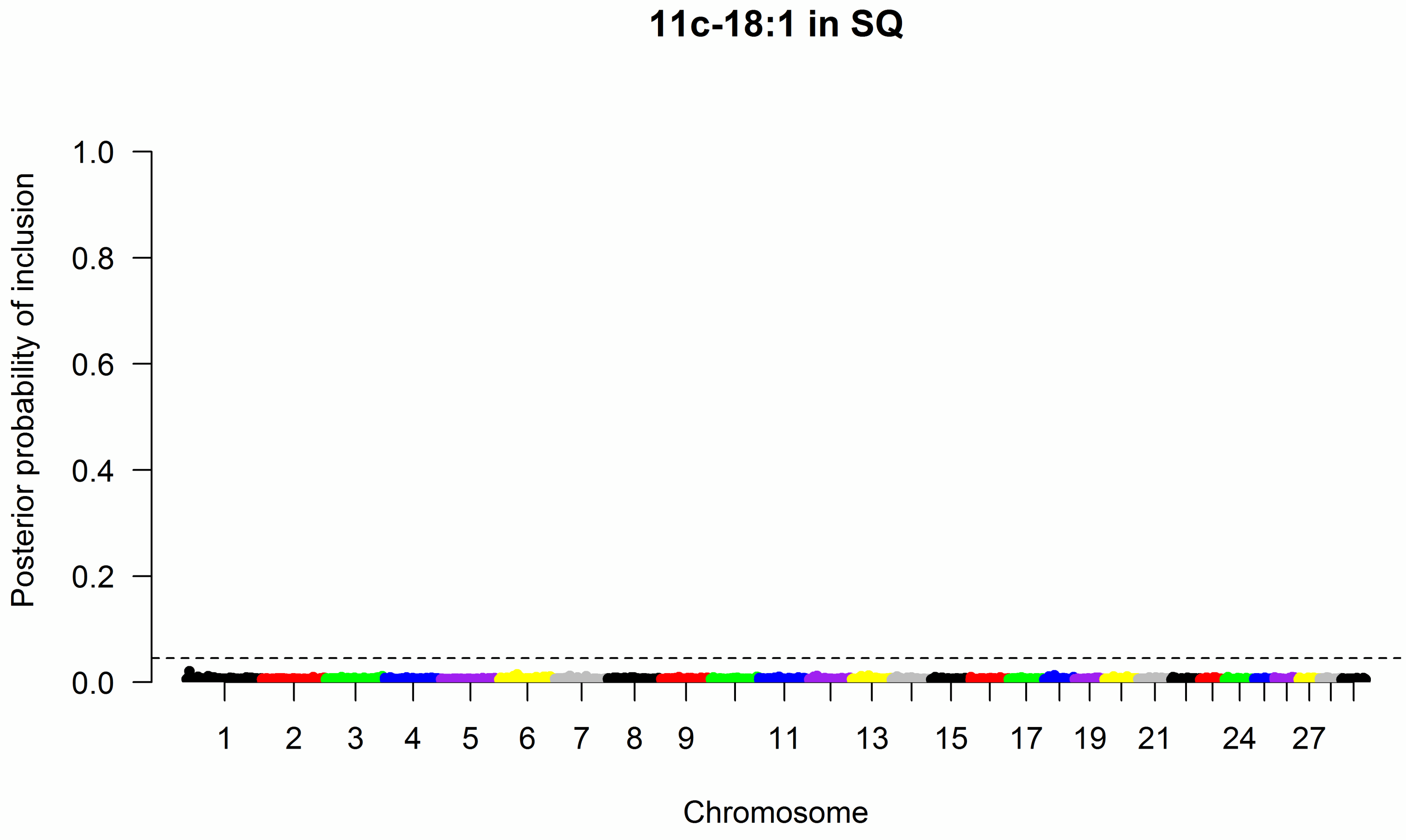
11c-18:1 in SQ
Posterior probability of inclusion
1.0
0.8
0.6
0.4
0.2
0.0
1
2
3
4
5
6
7
8
9
11
13
15
17
19
21
24
27
Chromosome

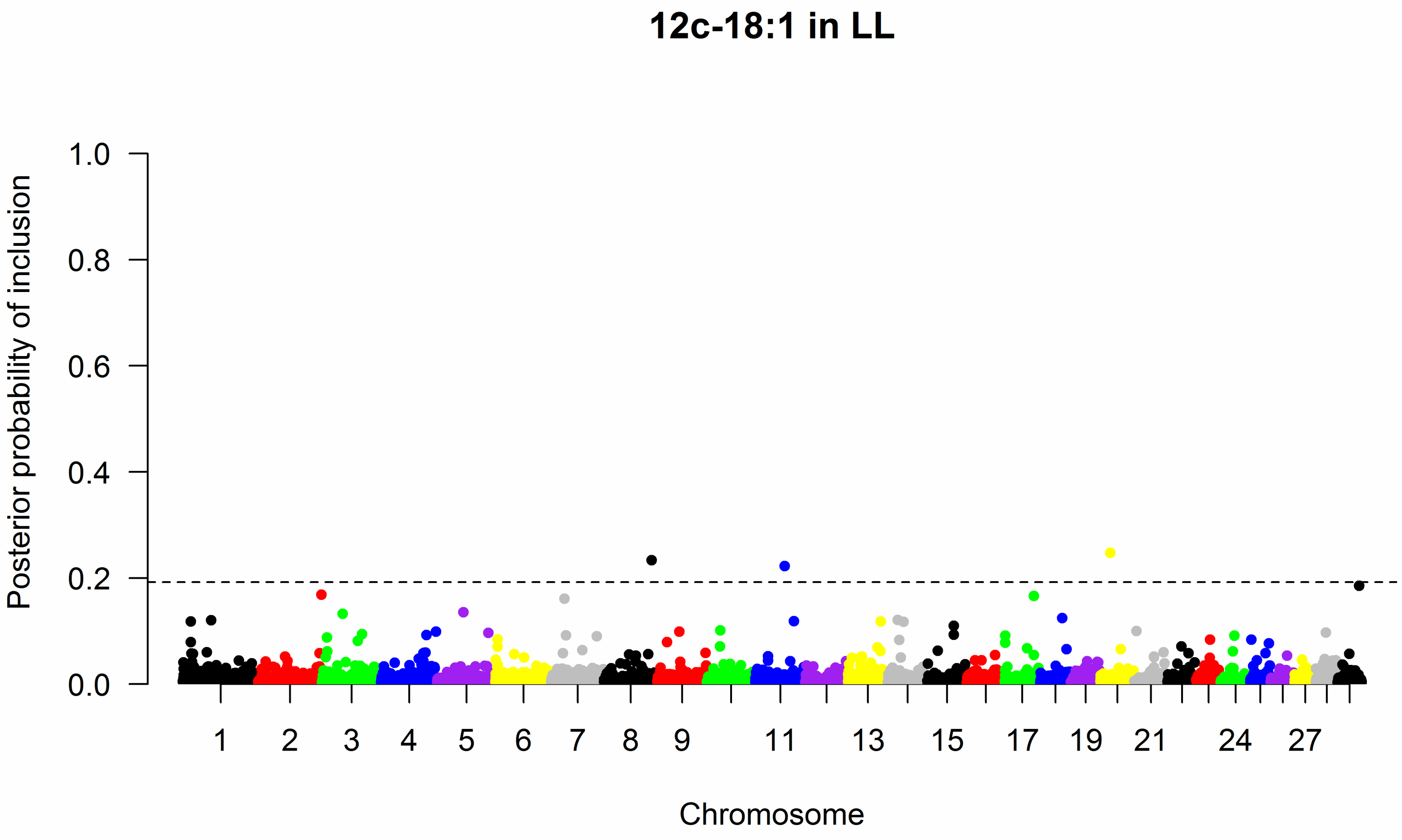
12c-18:1 in LL
Posterior probability of inclusion
1.0
0.8
0.6
0.4
0.2
0.0
1
2
3
4
5
6
7
8
9
11
13
15
17
19
21
24
27
Chromosome

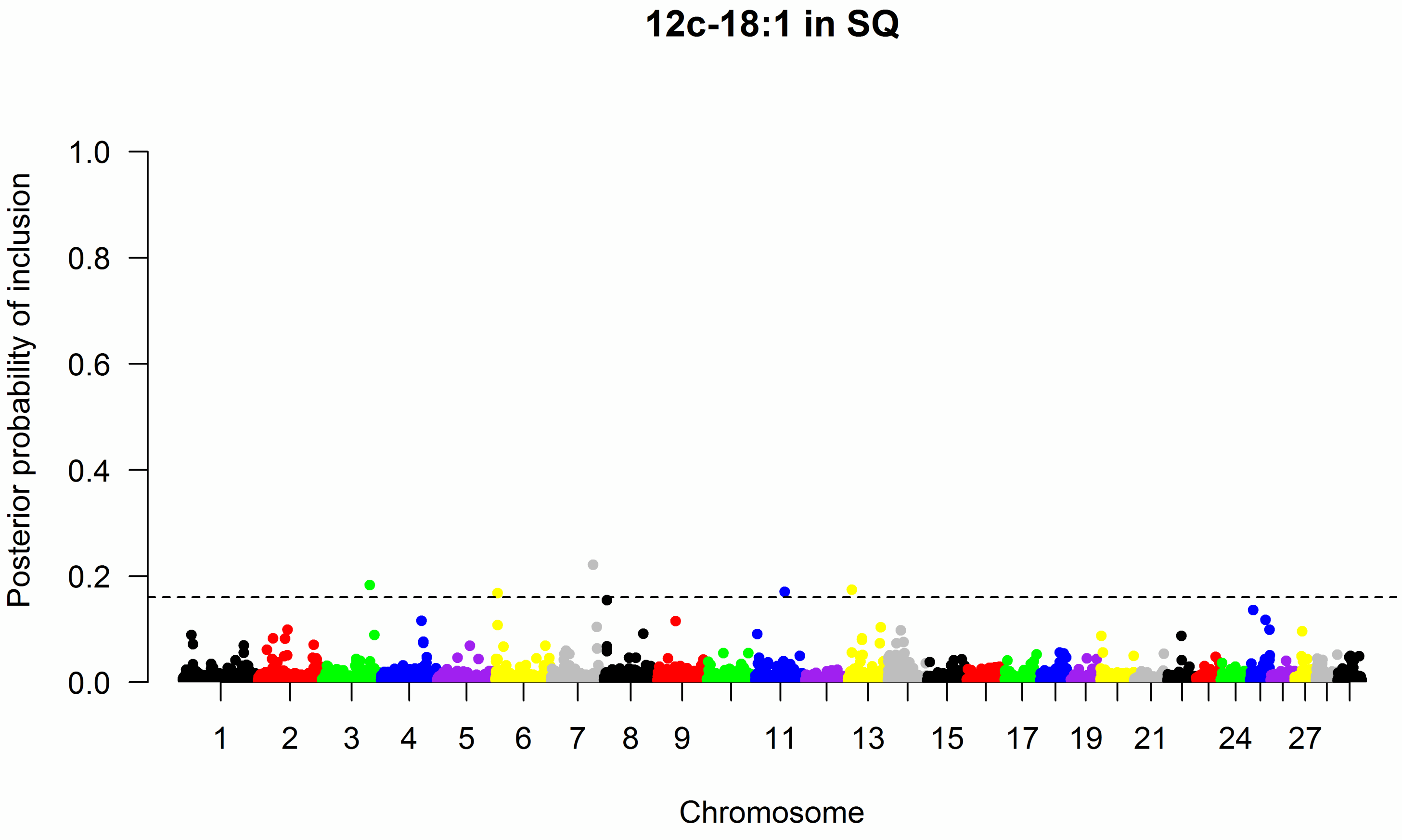
12c-18:1 in SQ
Posterior probability of inclusion
1.0
0.8
0.6
0.4
0.2
0.0
1
2
3
4
5
6
7
8
9
11
13
15
17
19
21
24
27
Chromosome

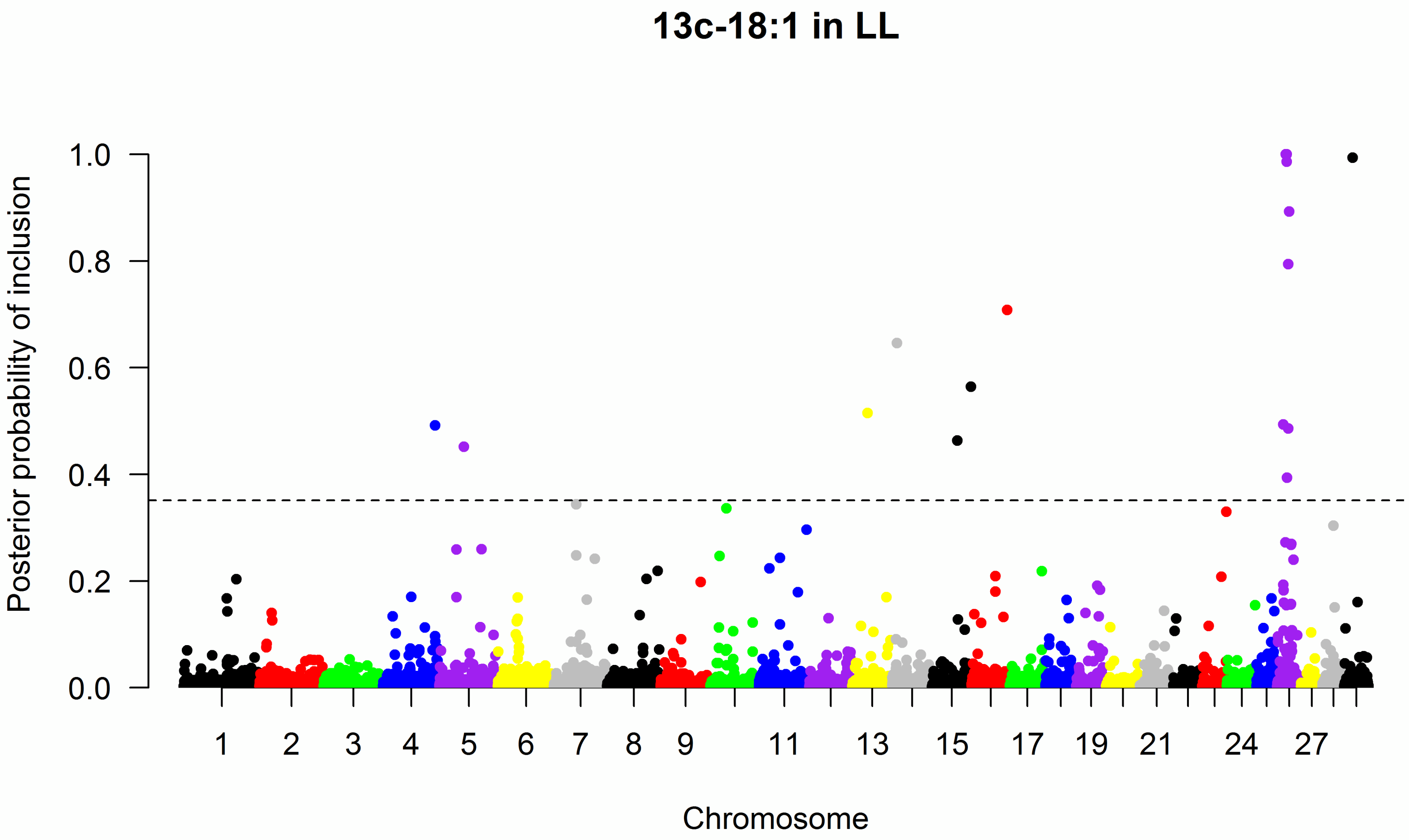
13c-18:1 in LL
Posterior probability of inclusion
0.0
0.2
0.4
0.6
0.8
1.0
1 2 3 4 5 6 7 8 9 11 13 15 17 19 21 24 27
Chromosome

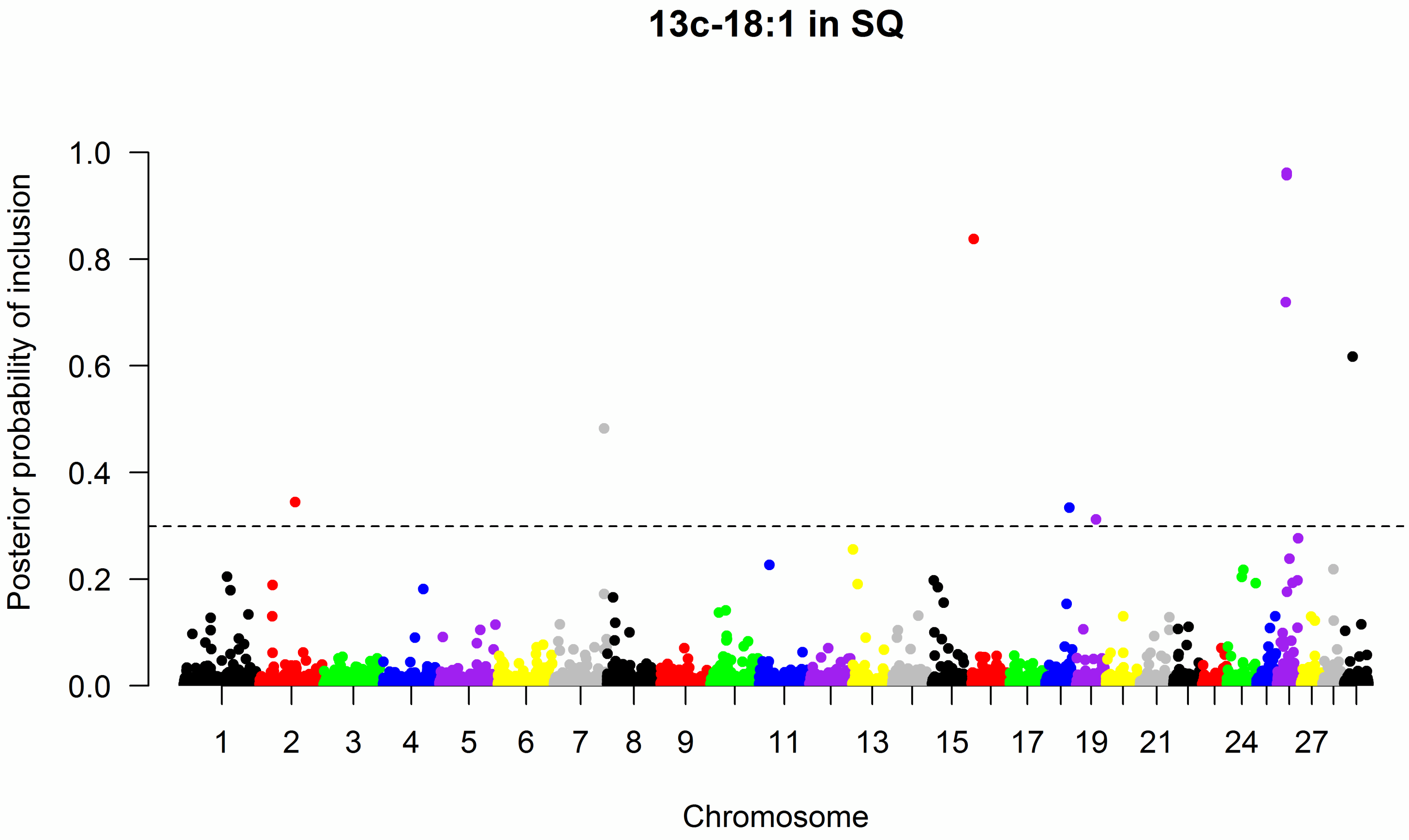
13c-18:1 in SQ
Posterior probability of inclusion
0.0
0.2
0.4
0.6
0.8
1.0
1 2 3 4 5 6 7 8 9 11 13 15 17 19 21 24 27
Chromosome

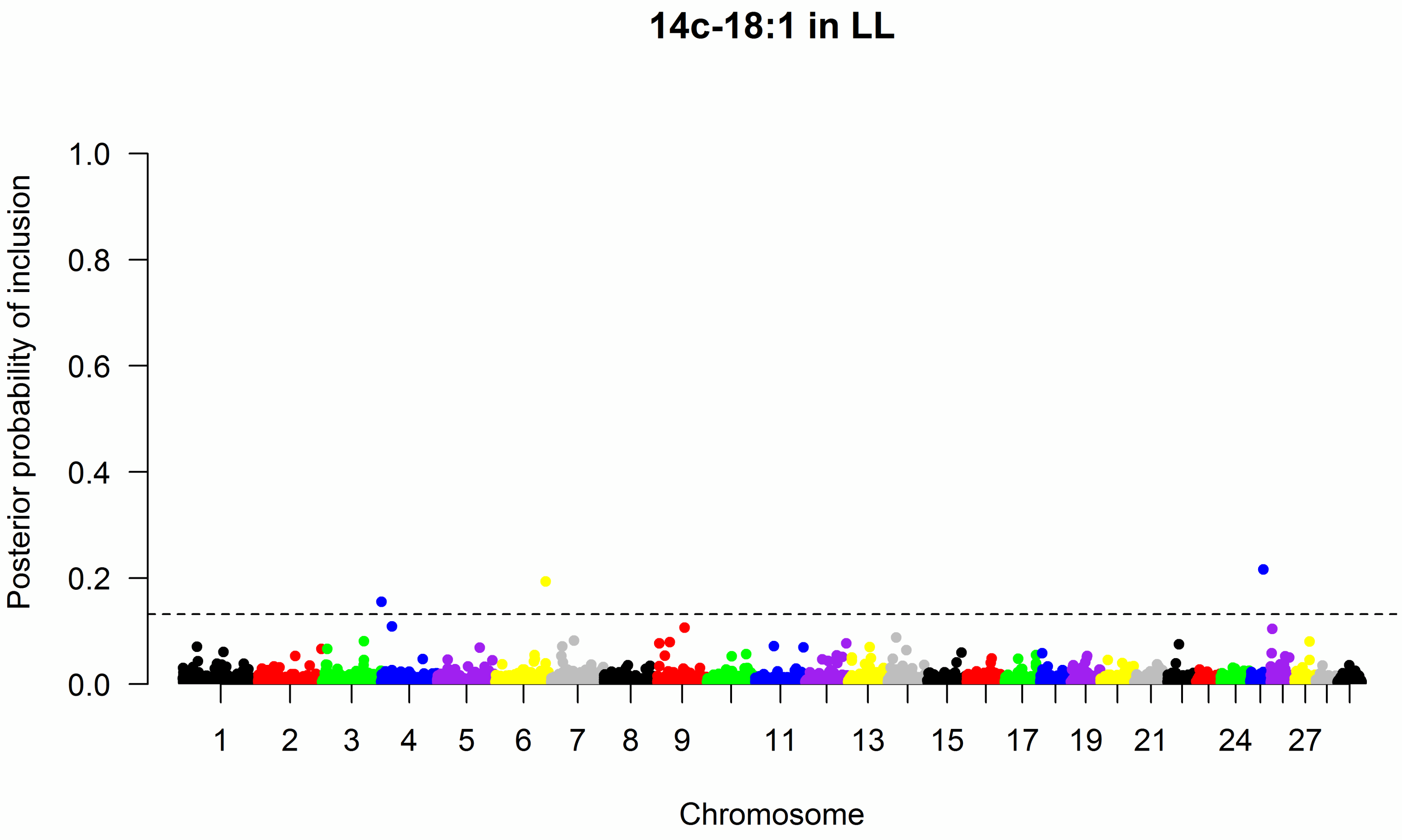
14c-18:1 in LL
1.0
0.8
0.6
0.4
0.2
0.0
Posterior probability of inclusion
1
2
3
4
5
6
7
8
9
11
13
15
17
19
21
24
27
Chromosome

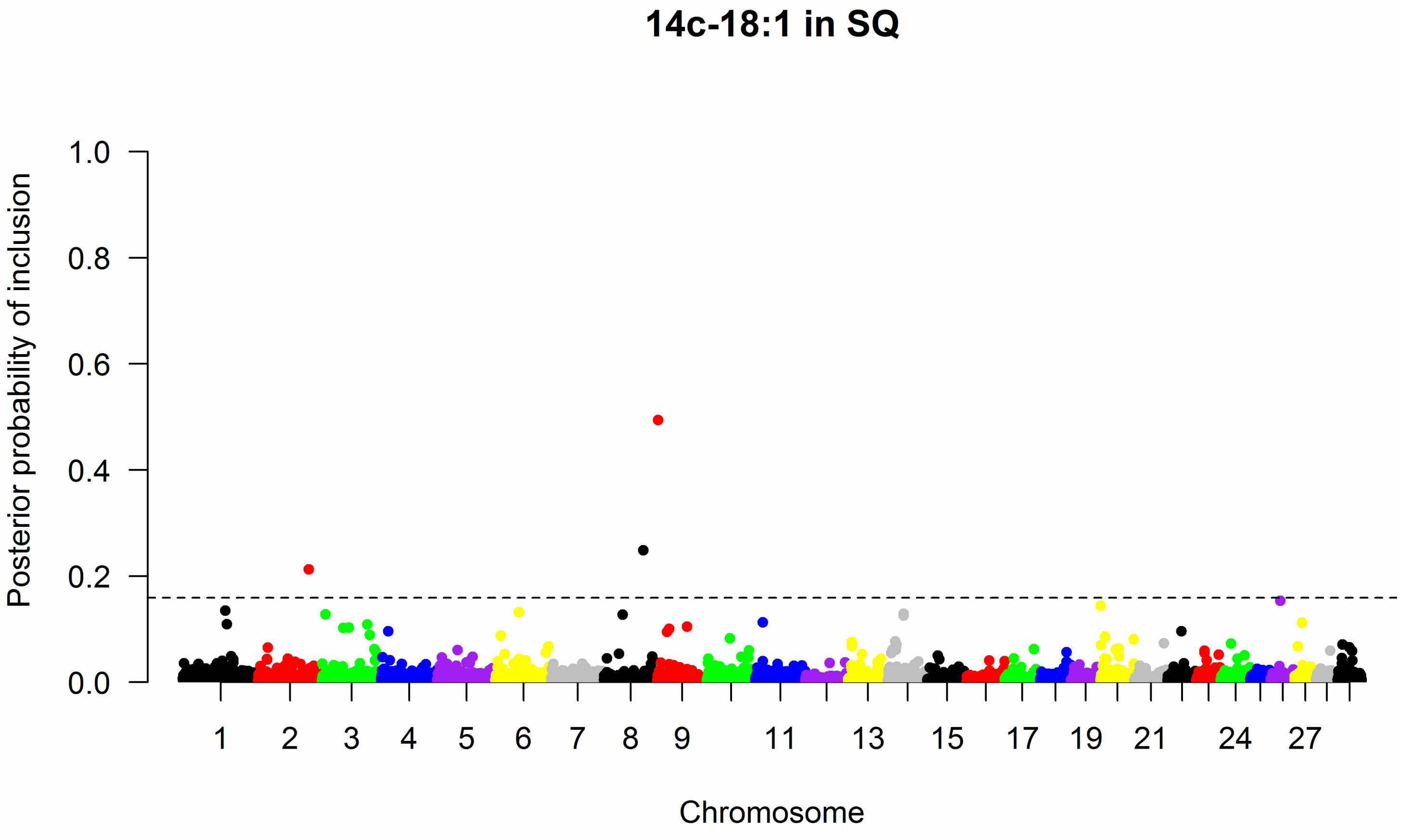
14c-18:1 in SQ
1.0
0.8
0.6
0.4
0.2
0.0
Posterior probability of inclusion
1
2
3
4
5
6
7
8
9
11
13
15
17
19
21
24
27
Chromosome

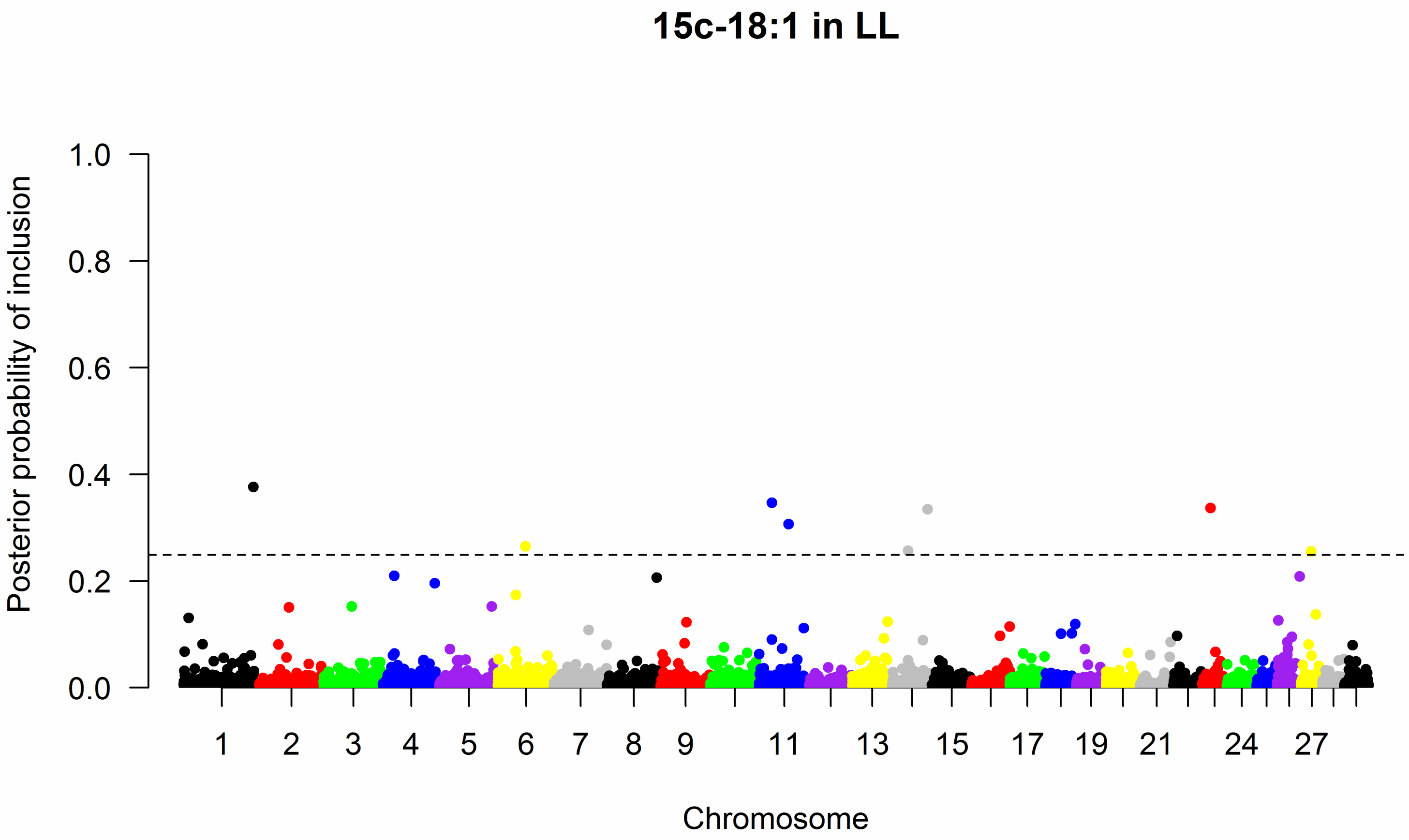
15c-18:1 in LL
Posterior probability of inclusion
1.0
0.8
0.6
0.4
0.2
0.0
1
2
3
4
5
6
7
8
9
11
13
15
17
19
21
24
27
Chromosome

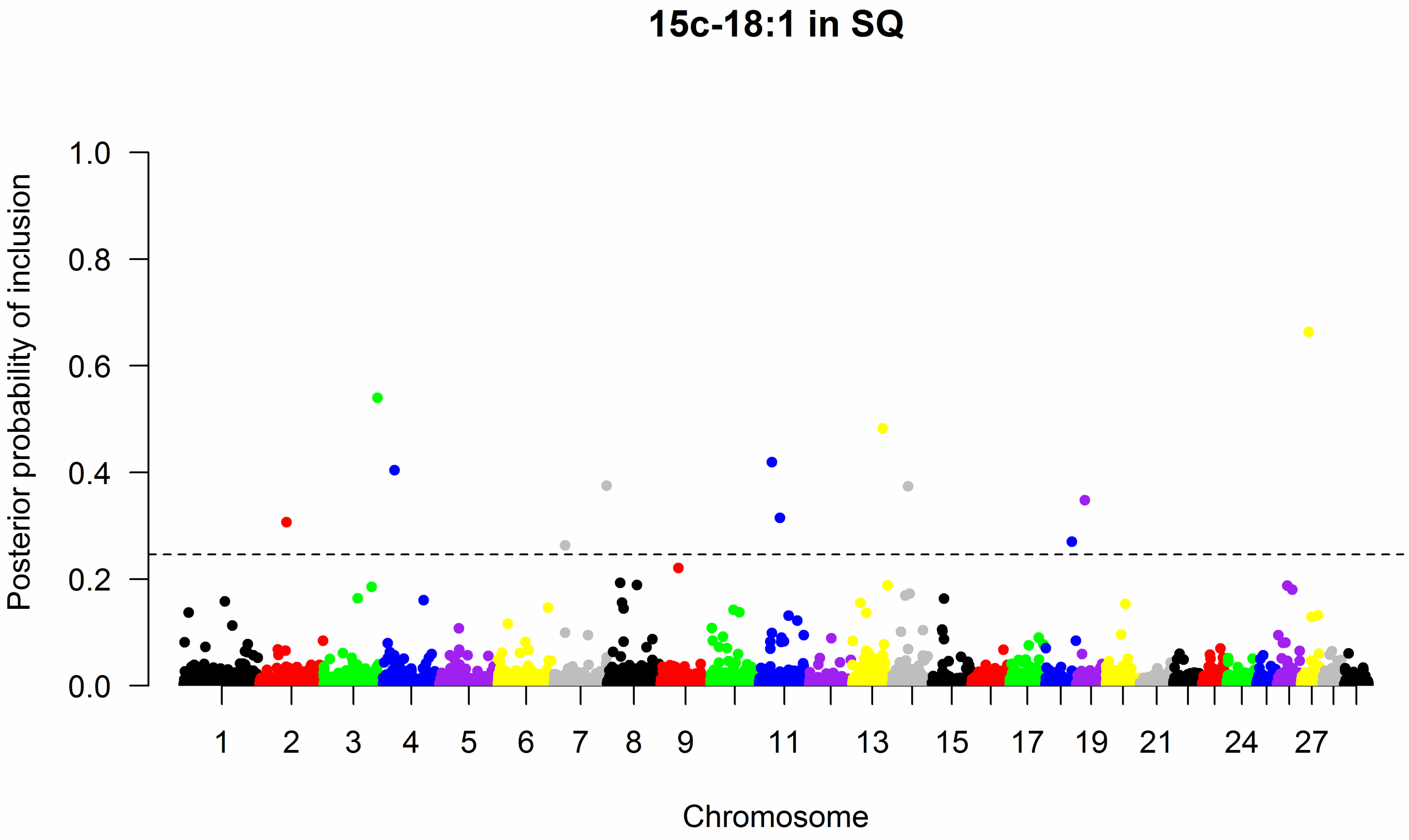
15c-18:1 in SQ
Posterior probability of inclusion
1.0
0.8
0.6
0.4
0.2
0.0
1
2
3
4
5
6
7
8
9
11
13
15
17
19
21
24
27
Chromosome

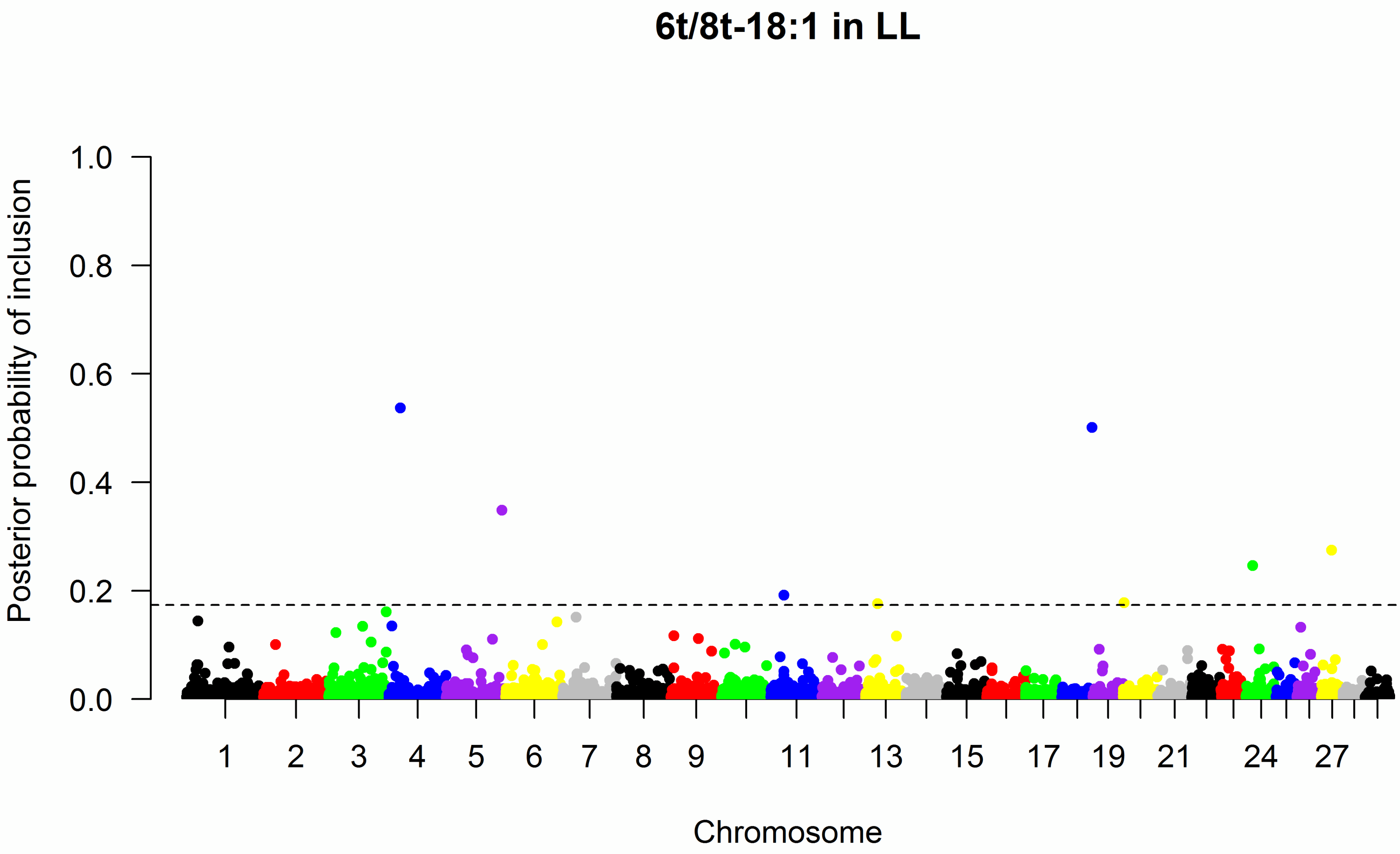

6t/8t-18:1 in LL
Posterior probability of inclusion
0.0
0.2
0.4
0.6
0.8
1.0
1
2
3
4
5
6
7
8
9
11
13
15
17
19
21
24
27
Chromosome

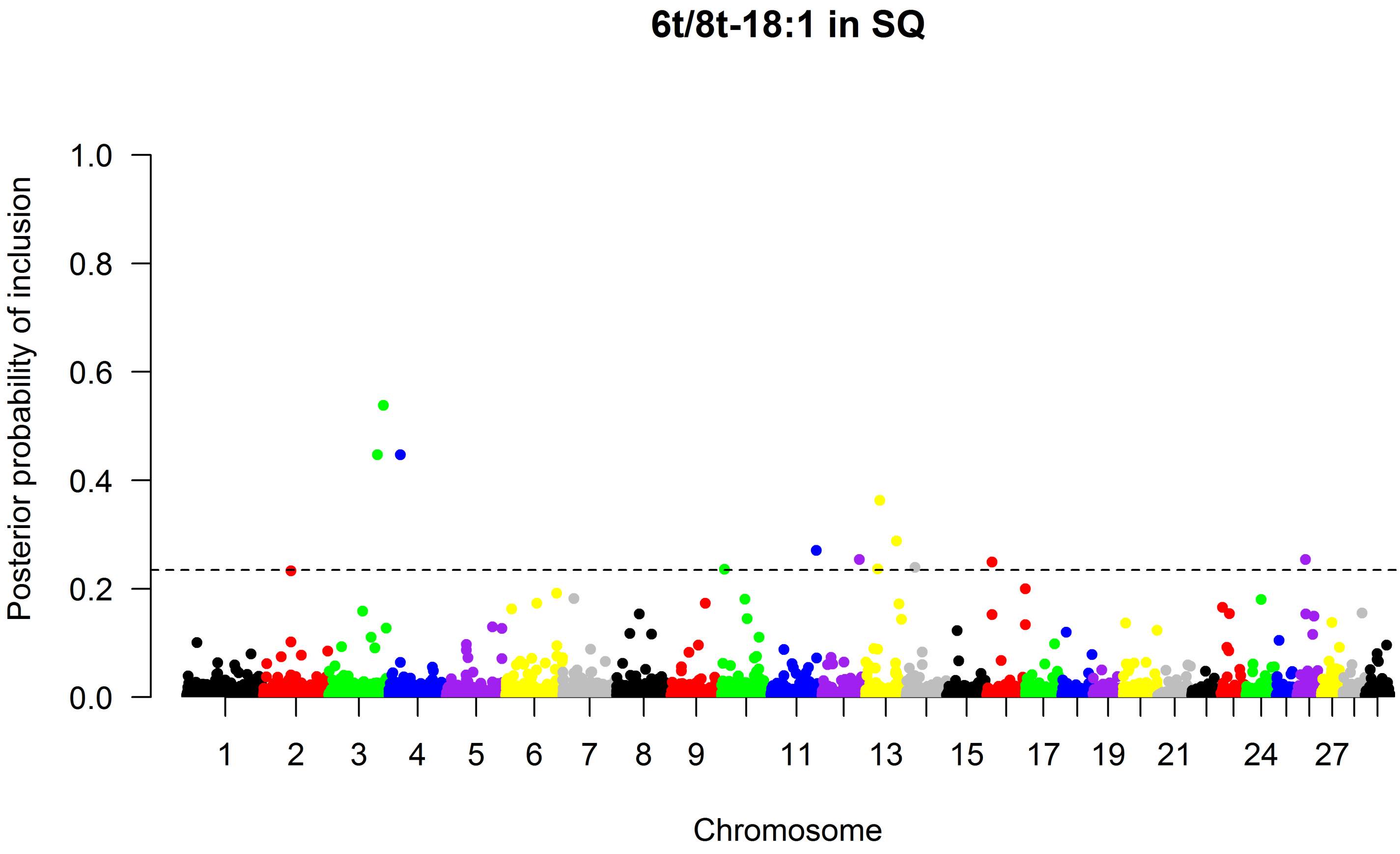

6t/8t-18:1 in SQ
Posterior probability of inclusion
0.0
0.2
0.4
0.6
0.8
1.0
1
2
3
4
5
6
7
8
9
11
13
15
17
19
21
24
27
Chromosome

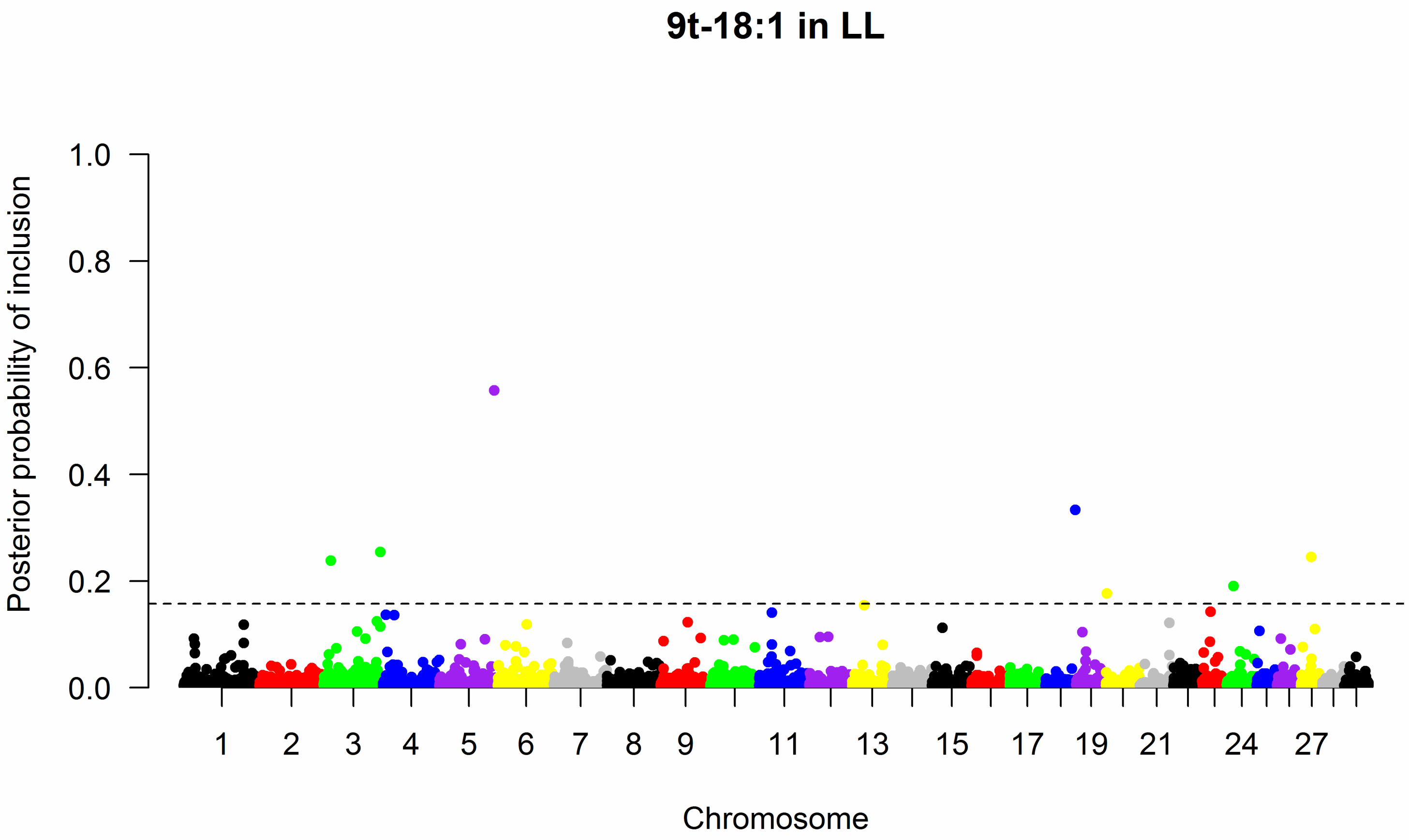
9t-18:1 in LL
Posterior probability of inclusion
1.0
0.8
0.6
0.4
0.2
0.0
1
2
3
4
5
6
7
8
9
11
13
15
17
19
21
24
27
Chromosome

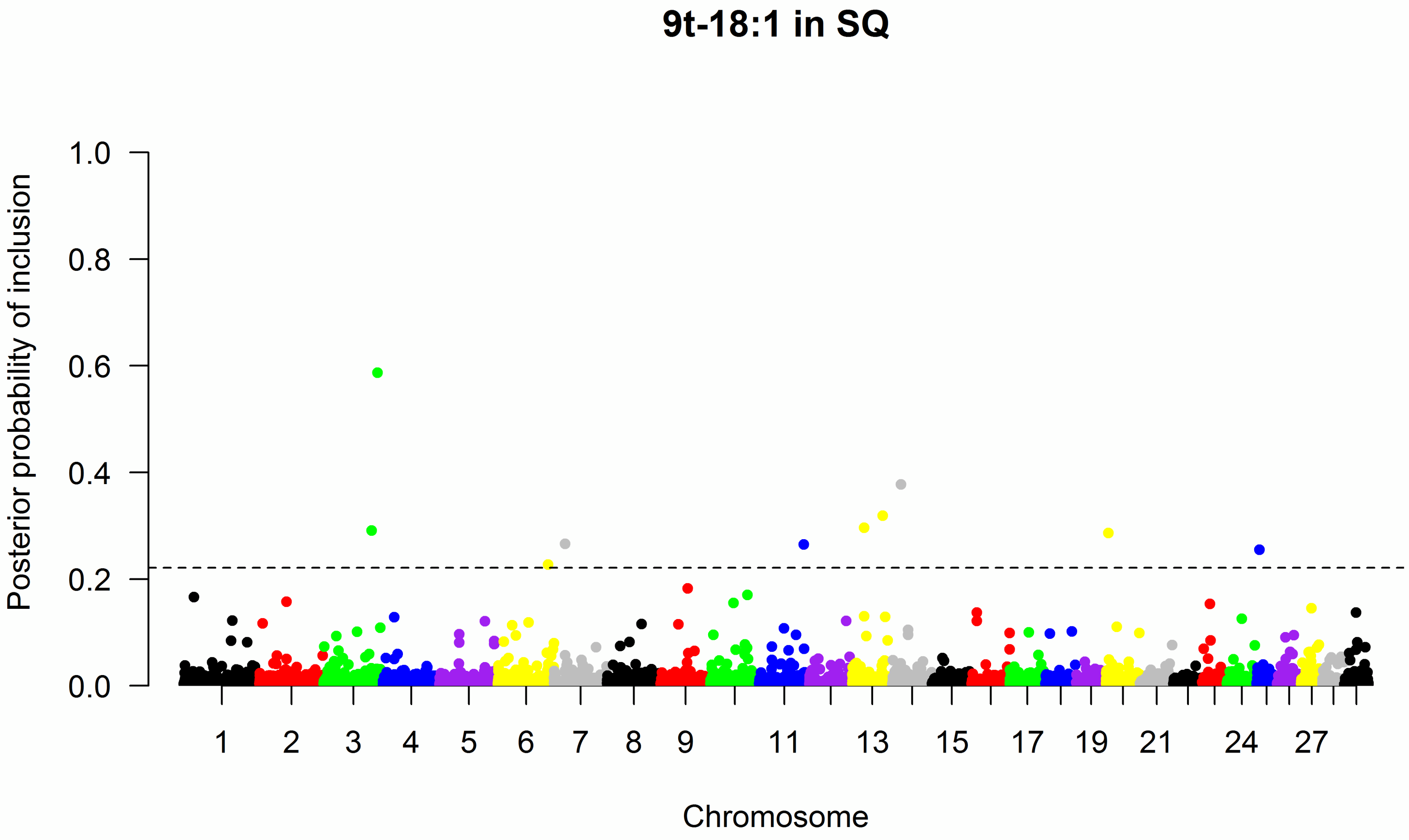
9t-18:1 in SQ
Posterior probability of inclusion
1.0
0.8
0.6
0.4
0.2
0.0
1
2
3
4
5
6
7
8
9
11
13
15
17
19
21
24
27
Chromosome

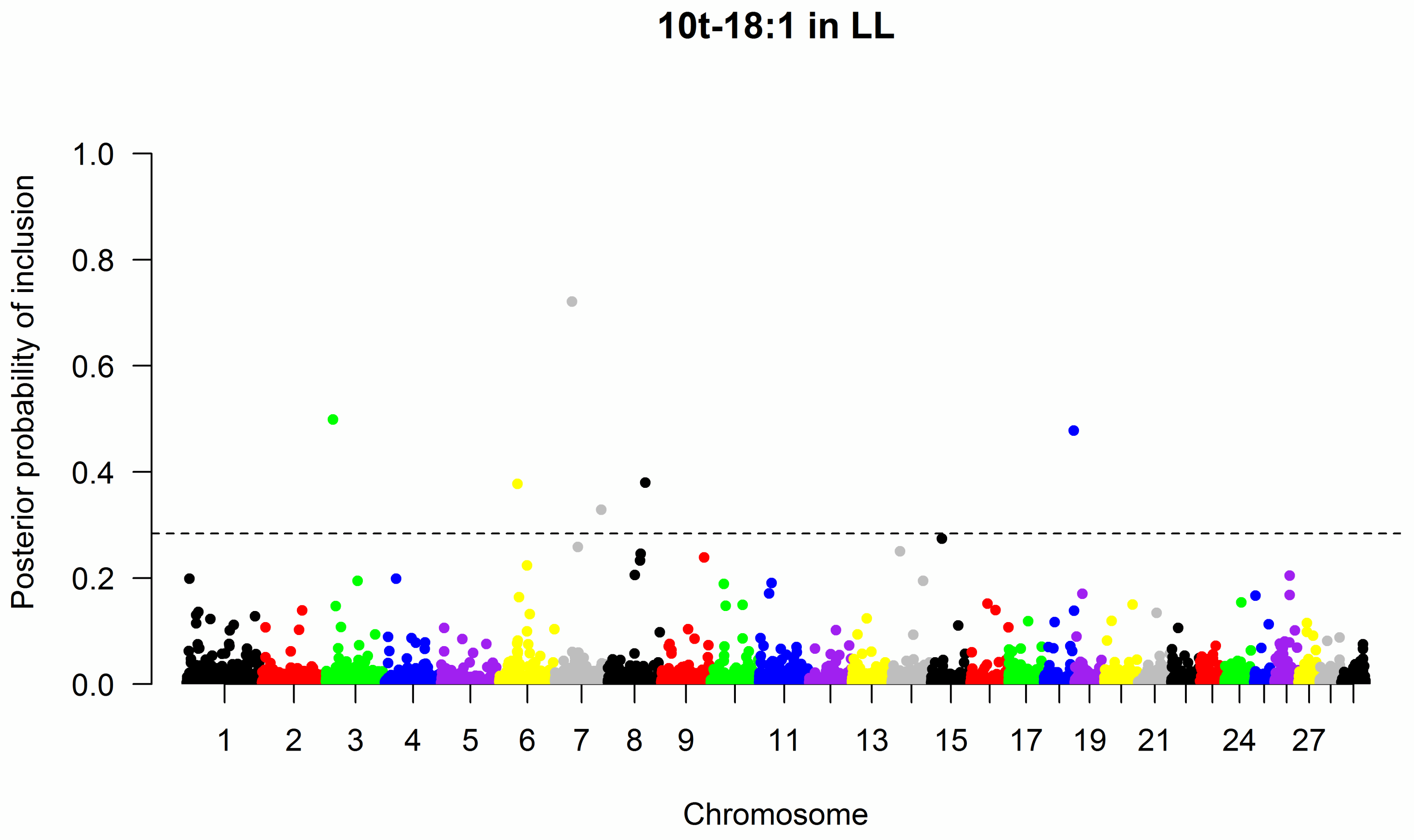
10t-18:1 in LL
Posterior probability of inclusion
0.0
0.2
0.4
0.6
0.8
1.0
1
2
3
4
5
6
7
8
9
11
13
15
17
19
21
24
27
Chromosome

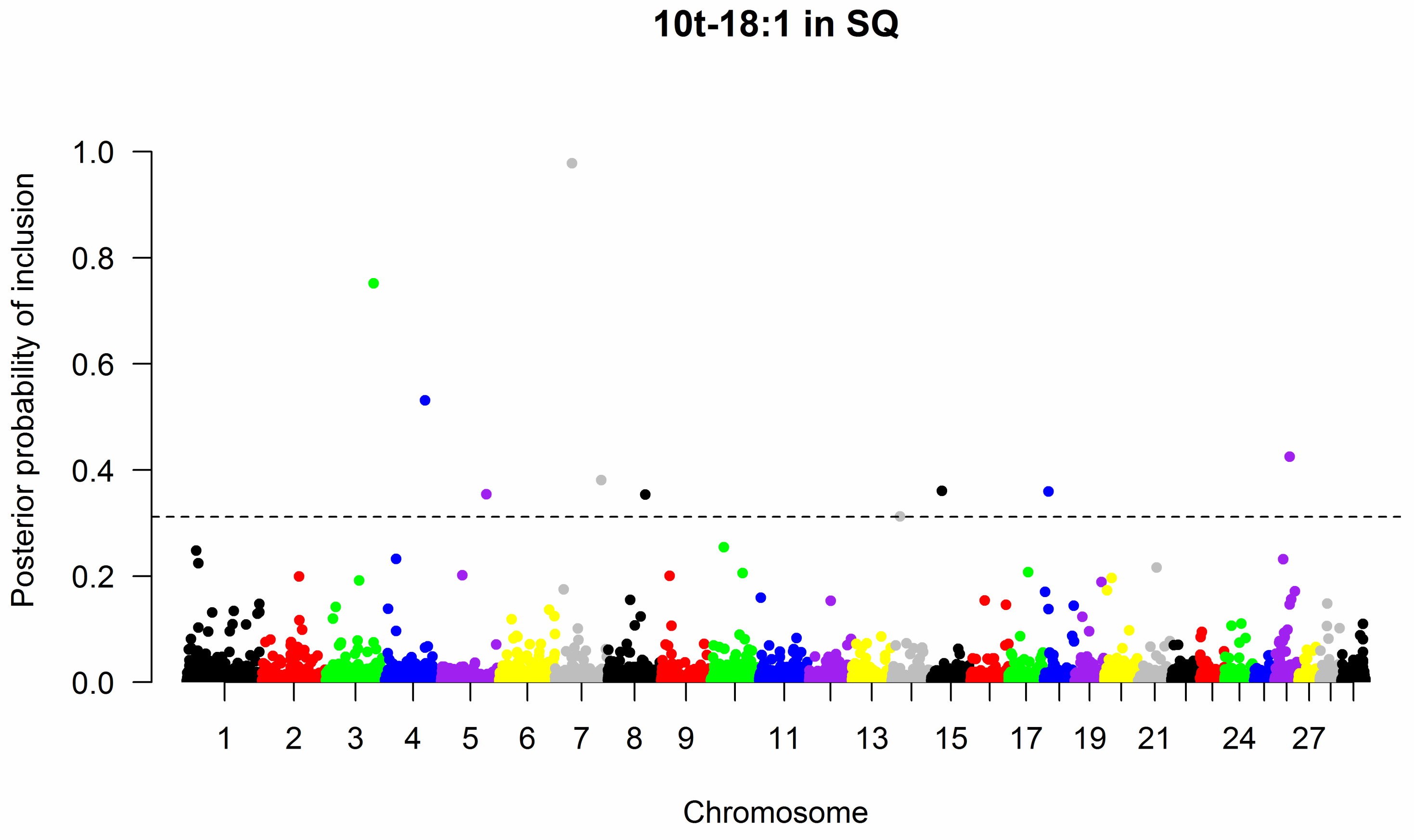
10t-18:1 in SQ
Posterior probability of inclusion
0.0
0.2
0.4
0.6
0.8
1.0
1
2
3
4
5
6
7
8
9
11
13
15
17
19
21
24
27
Chromosome

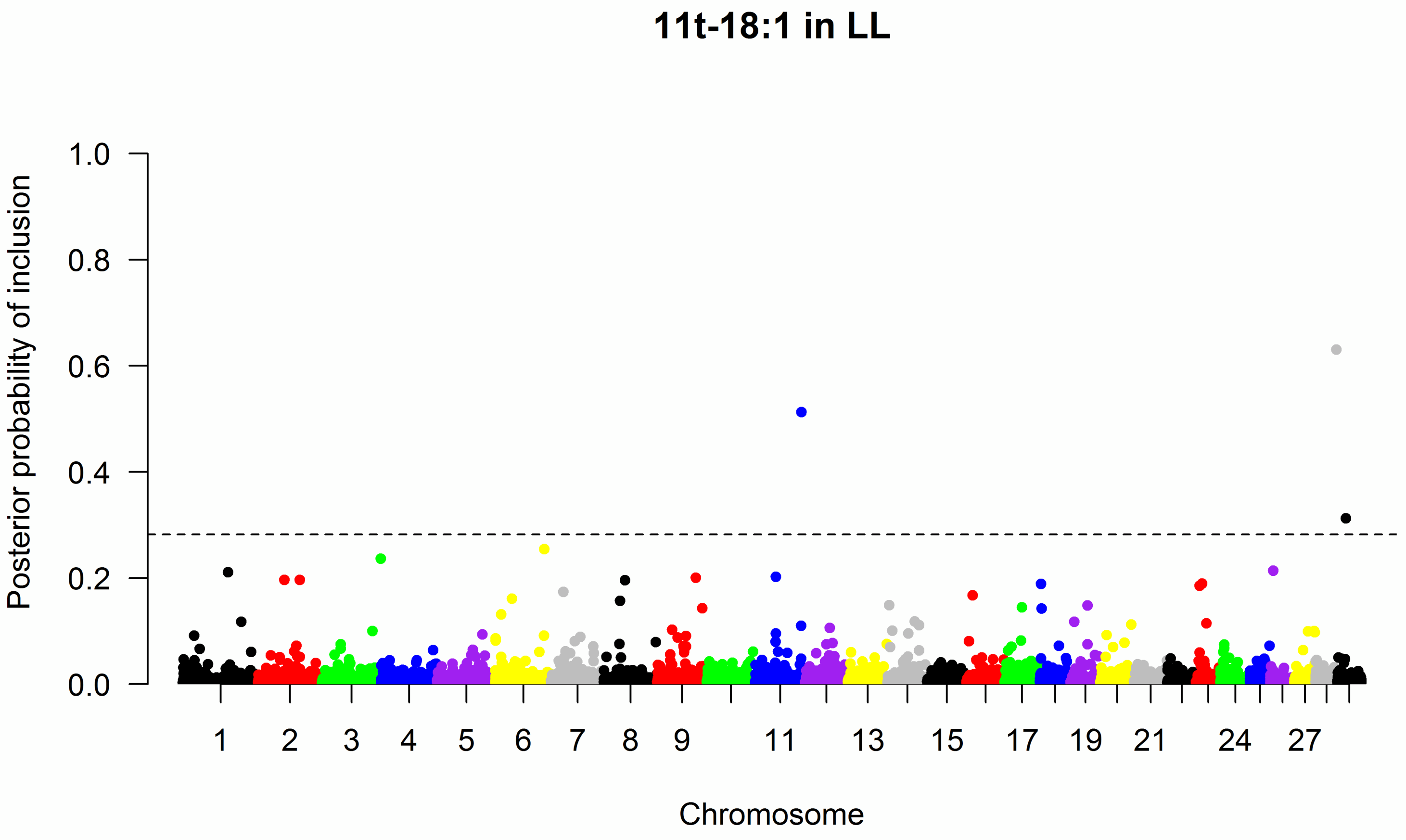

11t-18:1 in LL
Posterior probability of inclusion
0.0
0.2
0.4
0.6
0.8
1.0
1
2
3
4
5
6
7
8
9
11
13
15
17
19
21
24
27
Chromosome

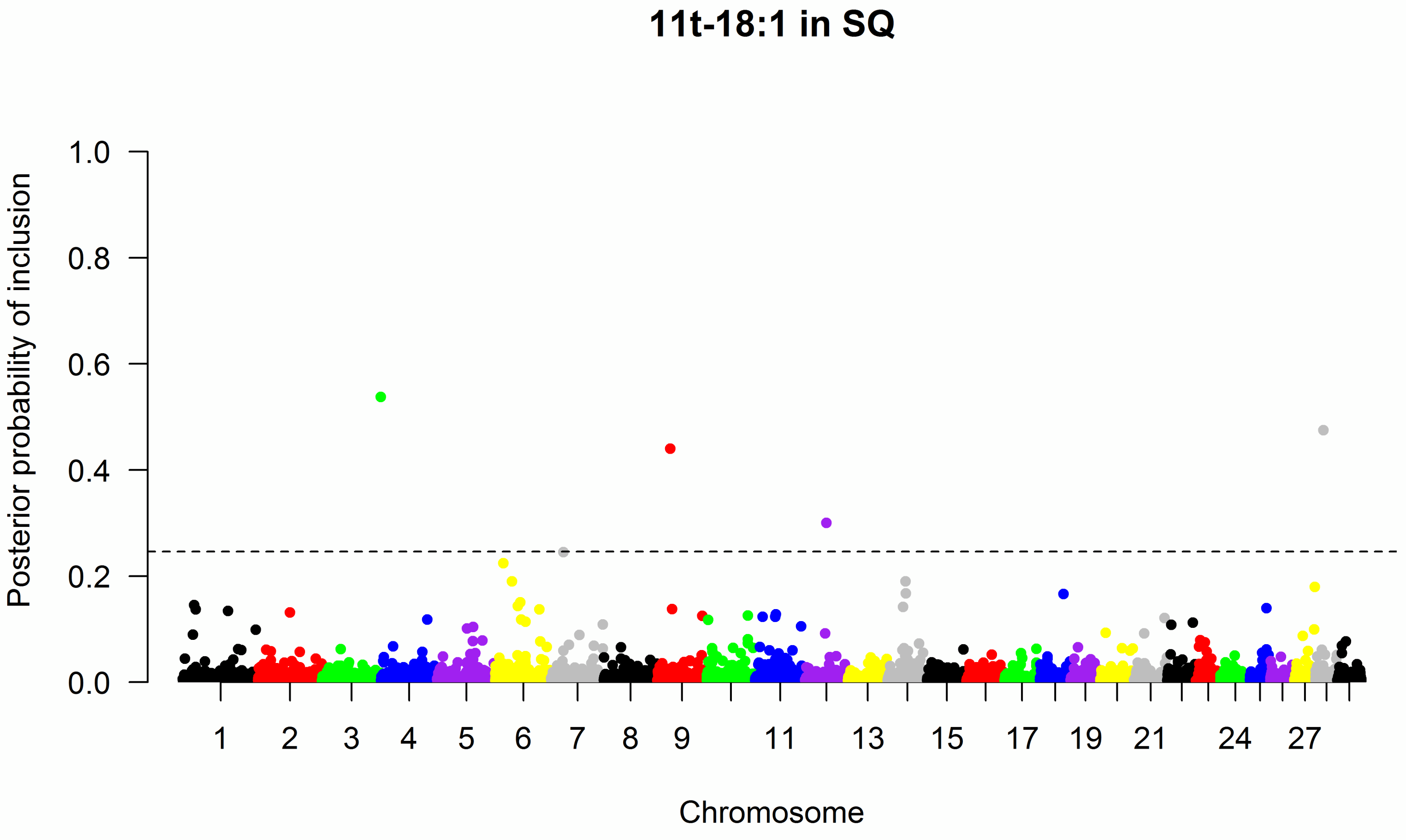

11t-18:1 in SQ
Posterior probability of inclusion
0.0
0.2
0.4
0.6
0.8
1.0
1
2
3
4
5
6
7
8
9
11
13
15
17
19
21
24
27
Chromosome

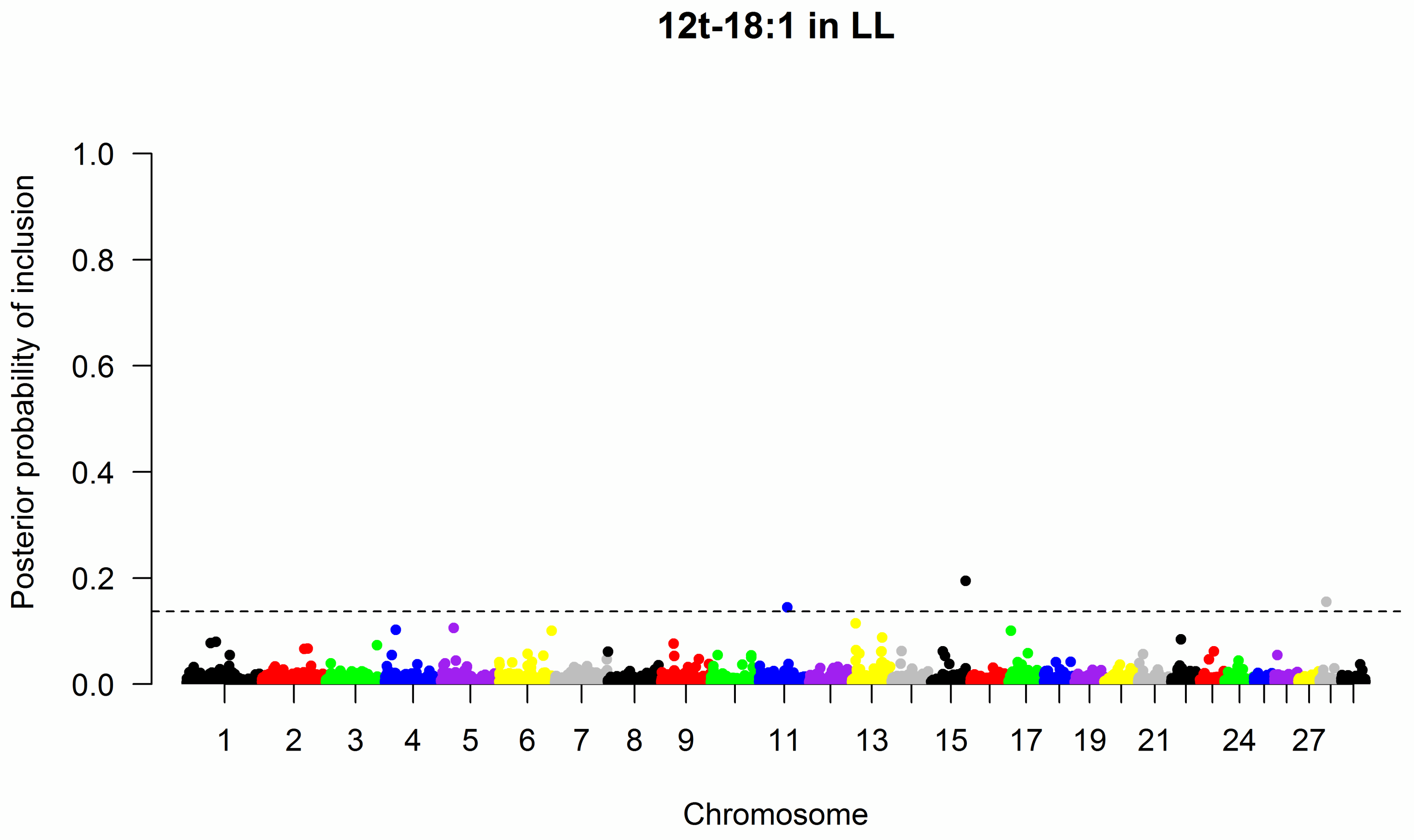
12t-18:1 in LL
Posterior probability of inclusion
1.0
0.8
0.6
0.4
0.2
0.0
1
2
3
4
5
6
7
8
9
11
13
15
17
19
21
24
27
Chromosome

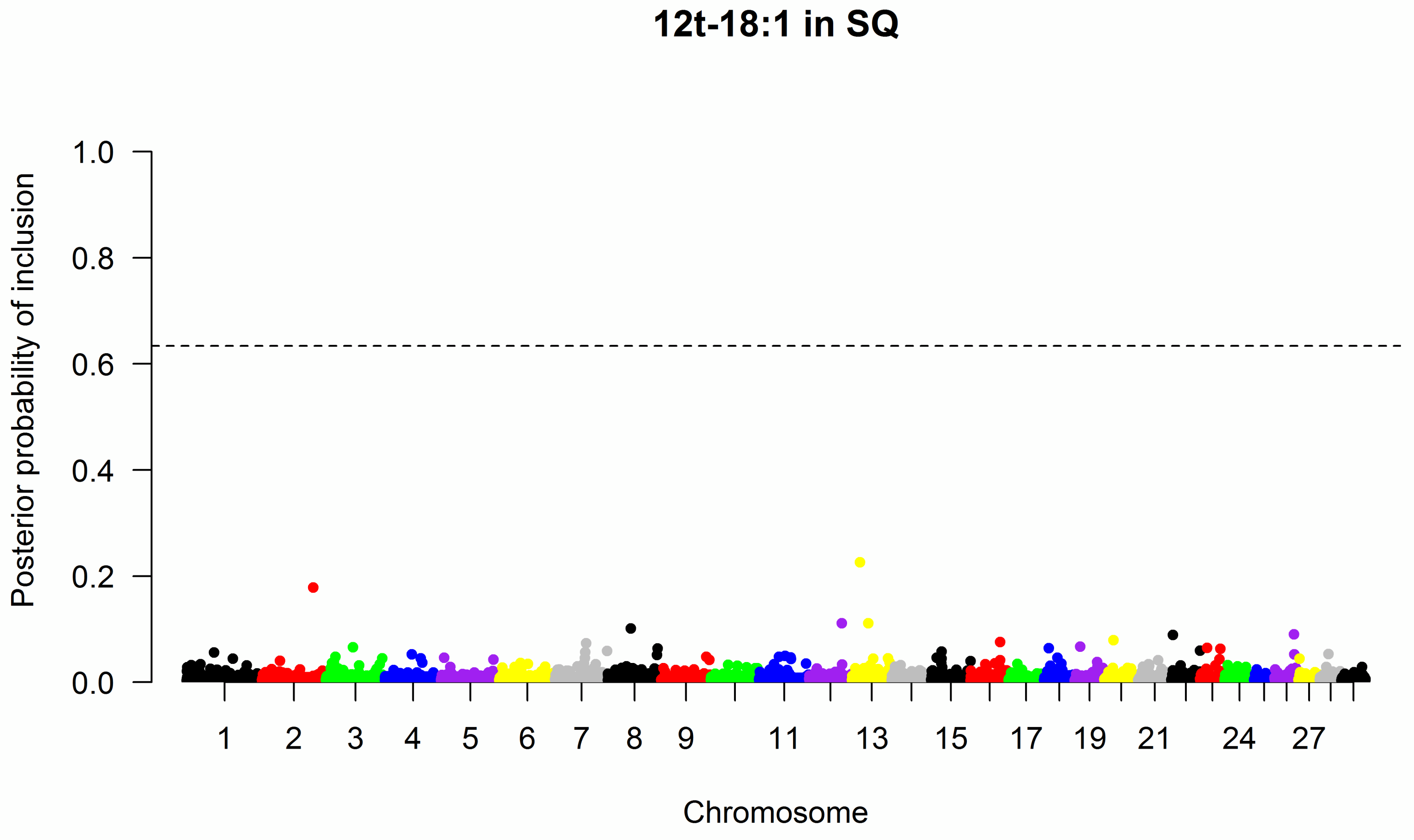
12t-18:1 in SQ
Posterior probability of inclusion
1.0
0.8
0.6
0.4
0.2
0.0
1
2
3
4
5
6
7
8
9
11
13
15
17
19
21
24
27
Chromosome

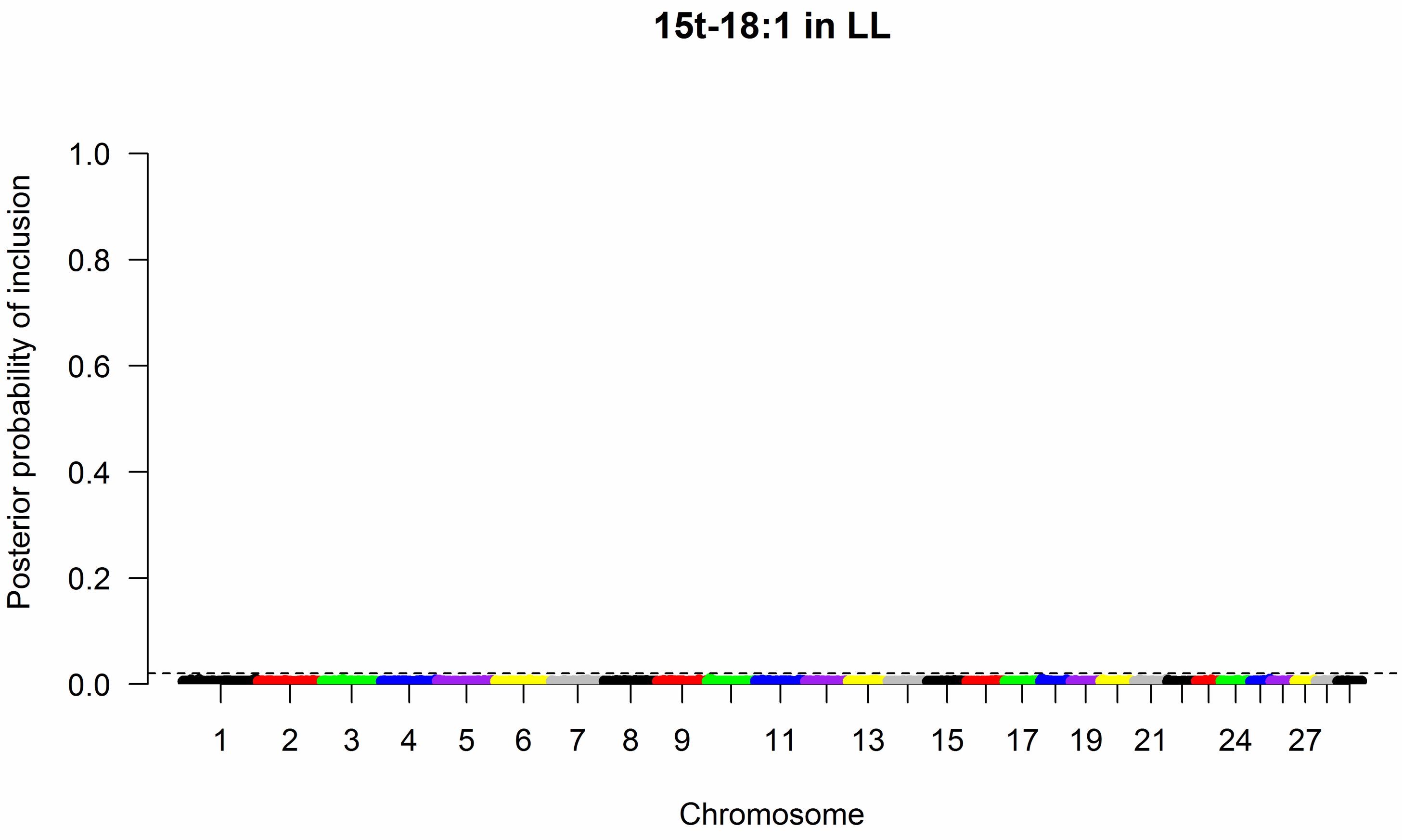
15t-18:1 in LL
Posterior probability of inclusion
0.0
0.2
0.4
0.6
0.8
1.0
1
2
3
4
5
6
7
8
9
11
13
15
17
19
21
24
27
Chromosome

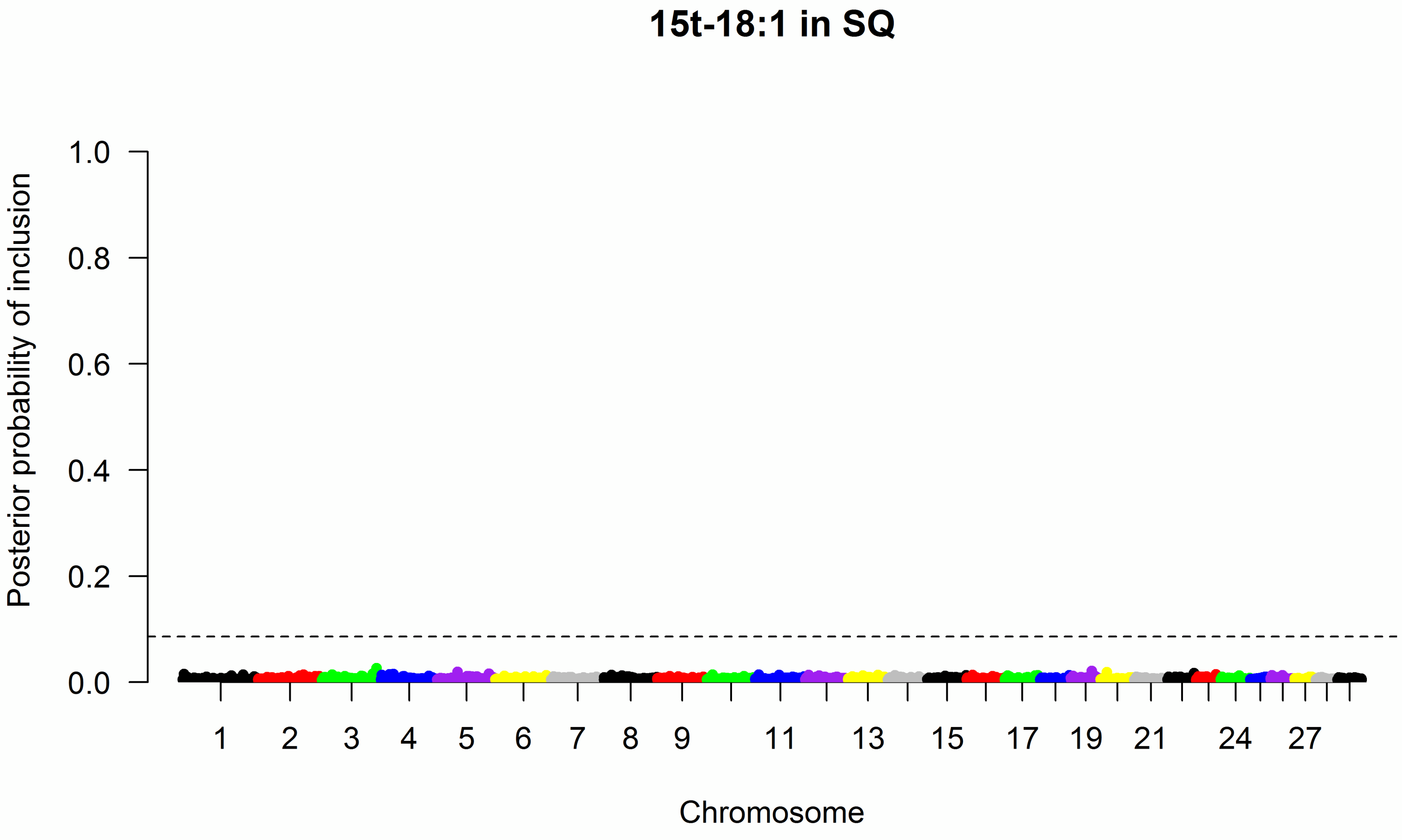
15t-18:1 in SQ
Posterior probability of inclusion
0.0
0.2
0.4
0.6
0.8
1.0
1
2
3
4
5
6
7
8
9
11
13
15
17
19
21
24
27
Chromosome

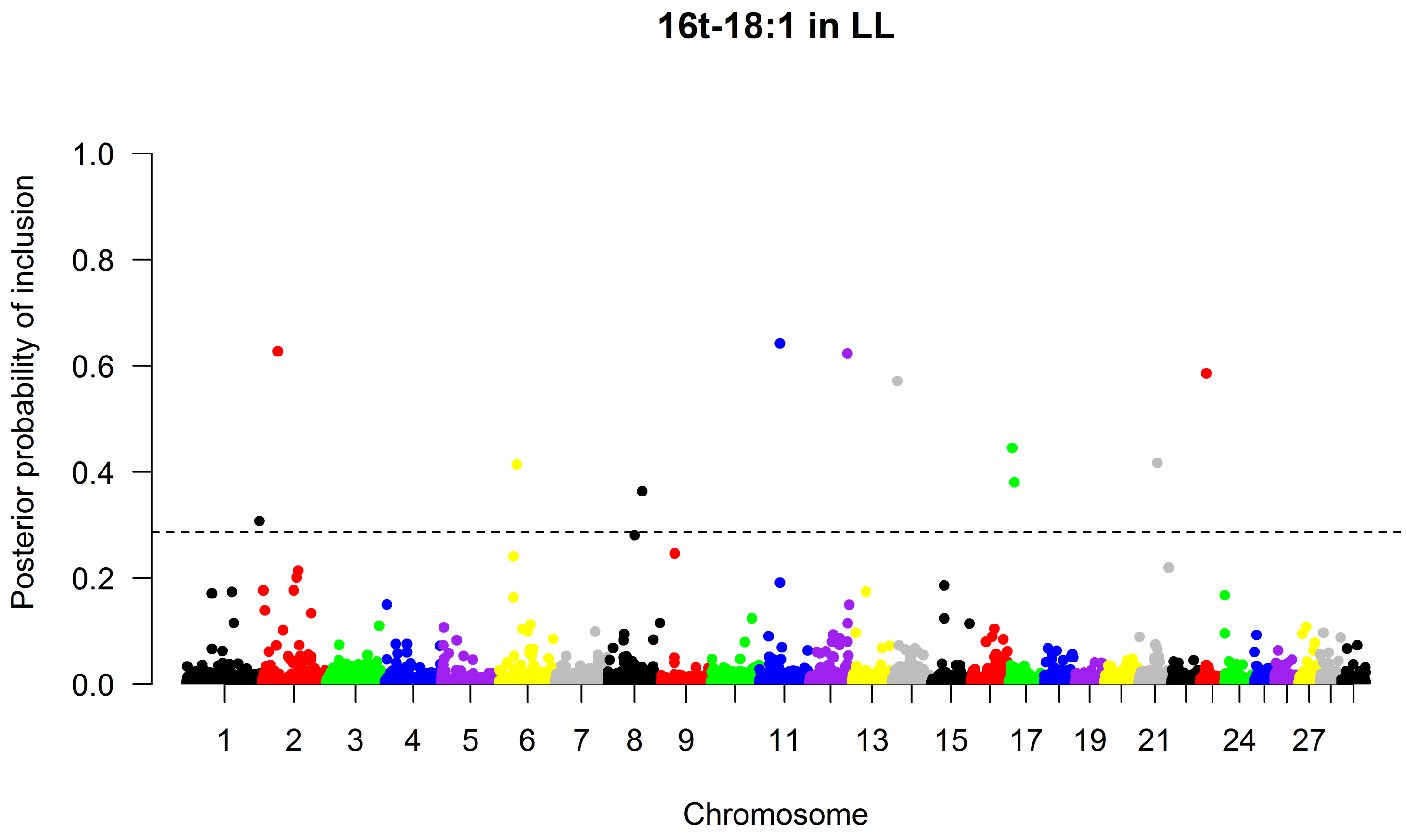
16t-18:1 in LL
Posterior probability of inclusion
1.0
0.8
0.6
0.4
0.2
0.0
1
2
3
4
5
6
7
8
9
11
13
15
17
19
21
24
27
Chromosome

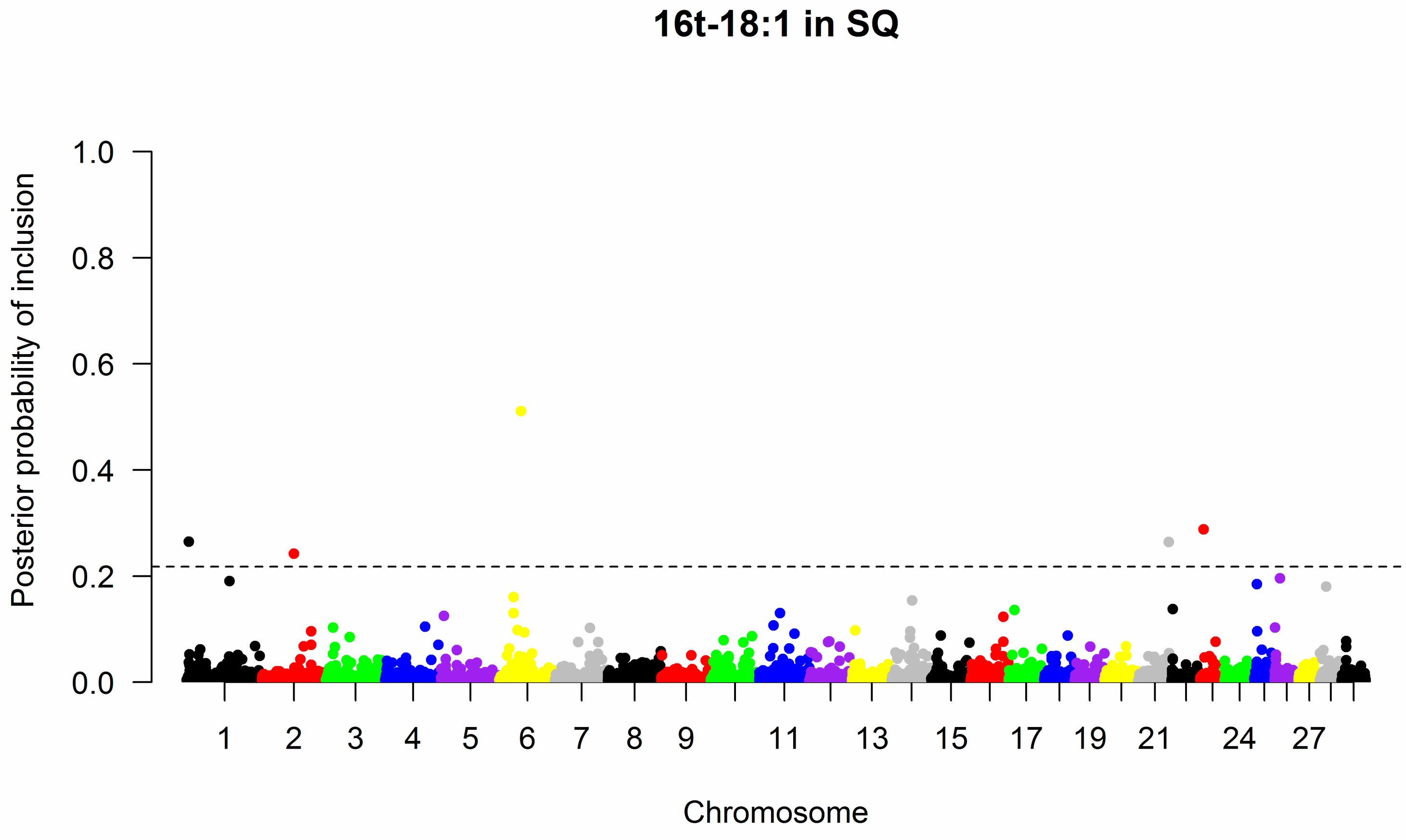
16t-18:1 in SQ
Posterior probability of inclusion
1.0
0.8
0.6
0.4
0.2
0.0
1
2
3
4
5
6
7
8
9
11
13
15
17
19
21
24
27
Chromosome

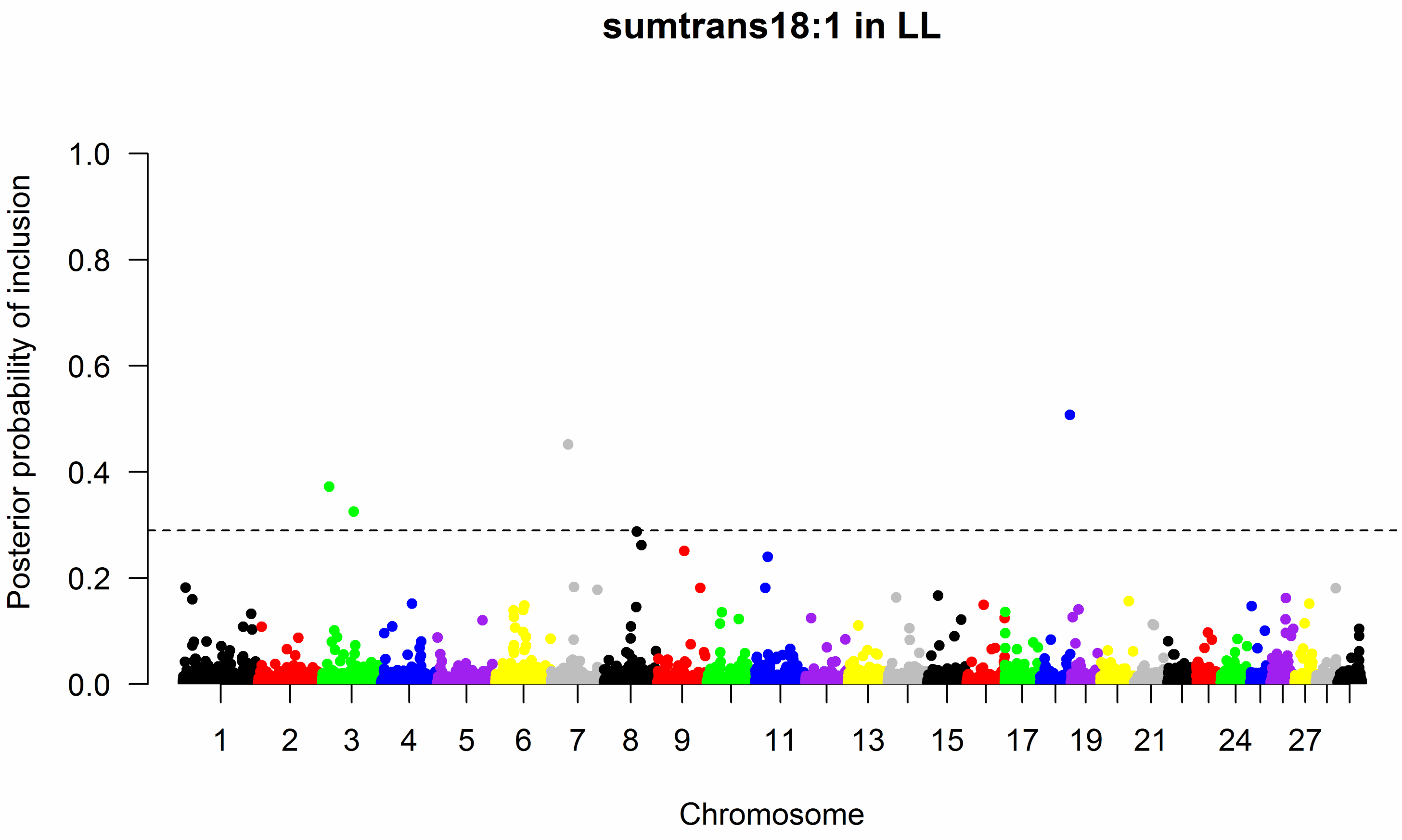
sumtrans18:1 in LL
Posterior probability of inclusion
1.0
0.8
0.6
0.4
0.2
0.0
1
2
3
4
5
6
7
8
9
11
13
15
17
19
21
24
27
Chromosome

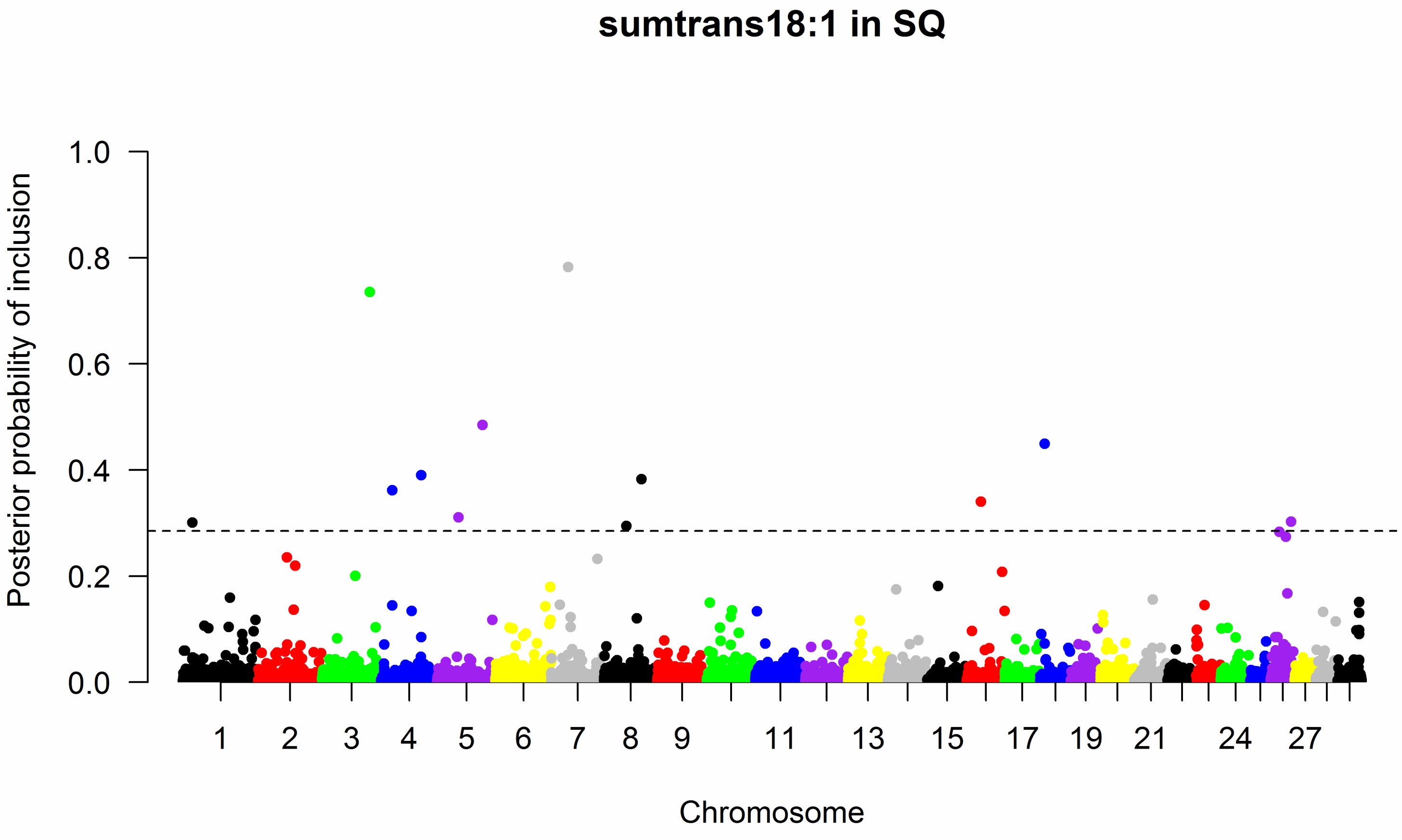
sumtrans18:1 in SQ
Posterior probability of inclusion
1.0
0.8
0.6
0.4
0.2
0.0
1
2
3
4
5
6
7
8
9
11
13
15
17
19
21
24
27
Chromosome

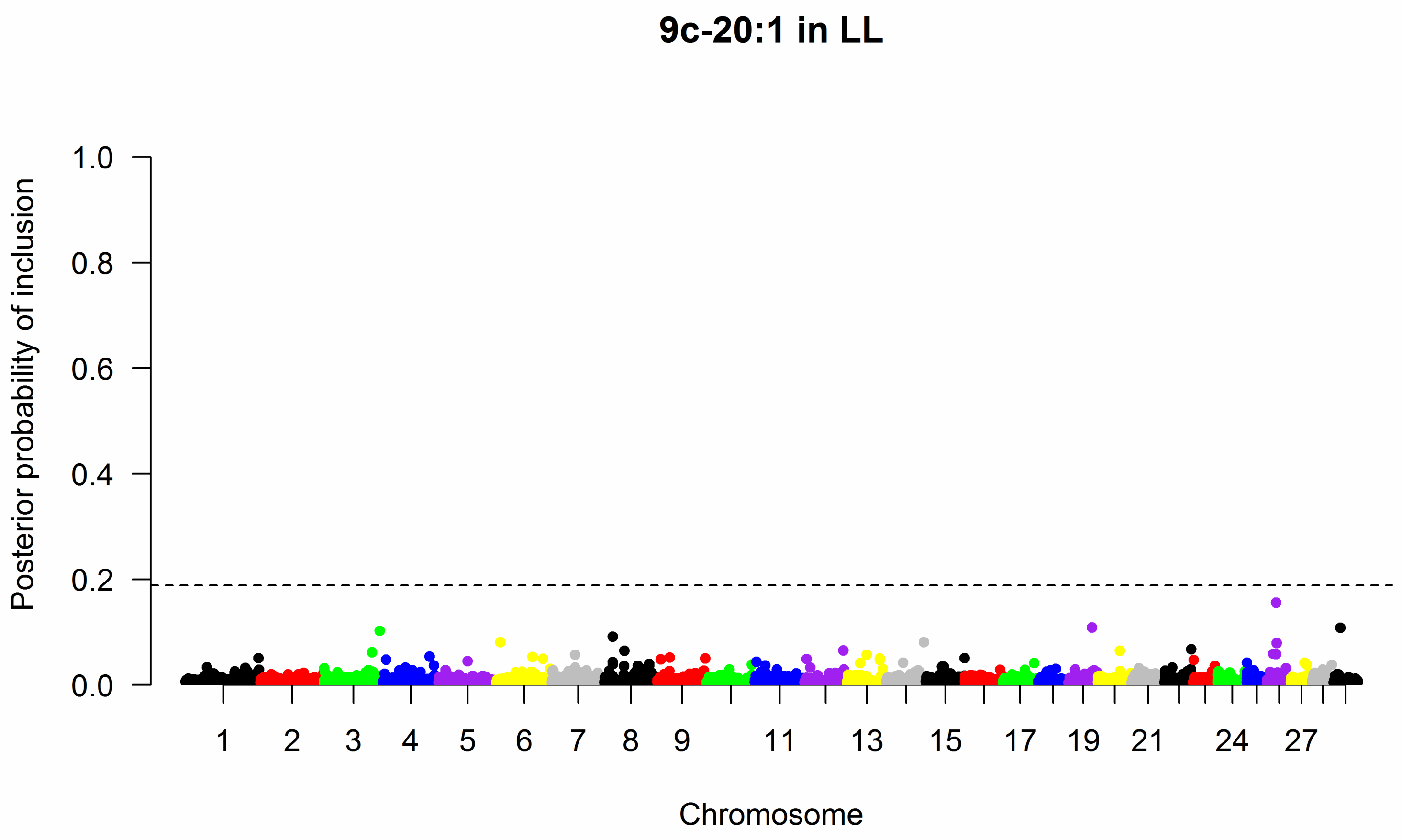
9c-20:1 in LL
Posterior probability of inclusion
1.0
0.8
0.6
0.4
0.2
0.0
1
2
3
4
5
6
7
8
9
11
13
15
17
19
21
24
27
Chromosome

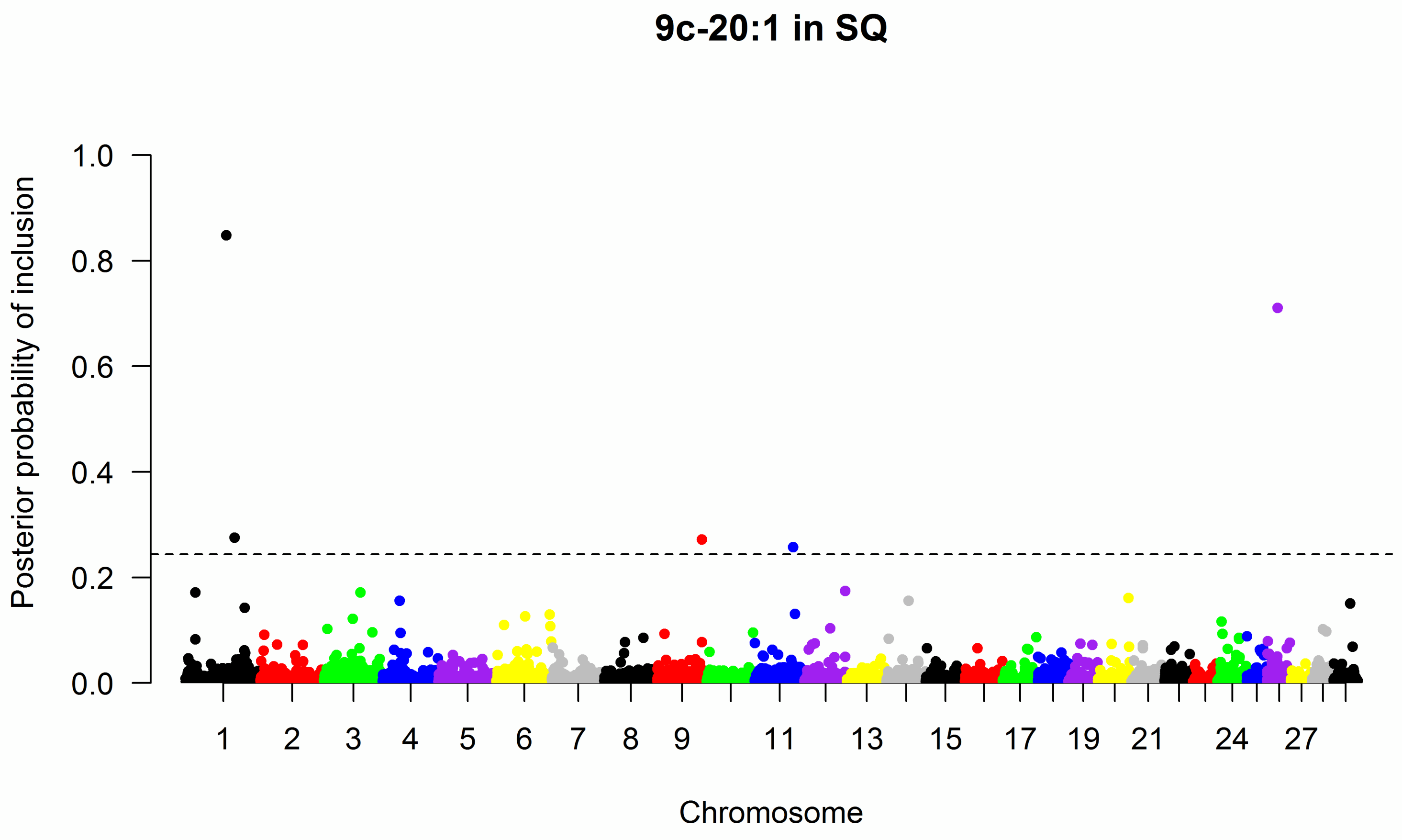
9c-20:1 in SQ
Posterior probability of inclusion
1.0
0.8
0.6
0.4
0.2
0.0
1
2
3
4
5
6
7
8
9
11
13
15
17
19
21
24
27
Chromosome

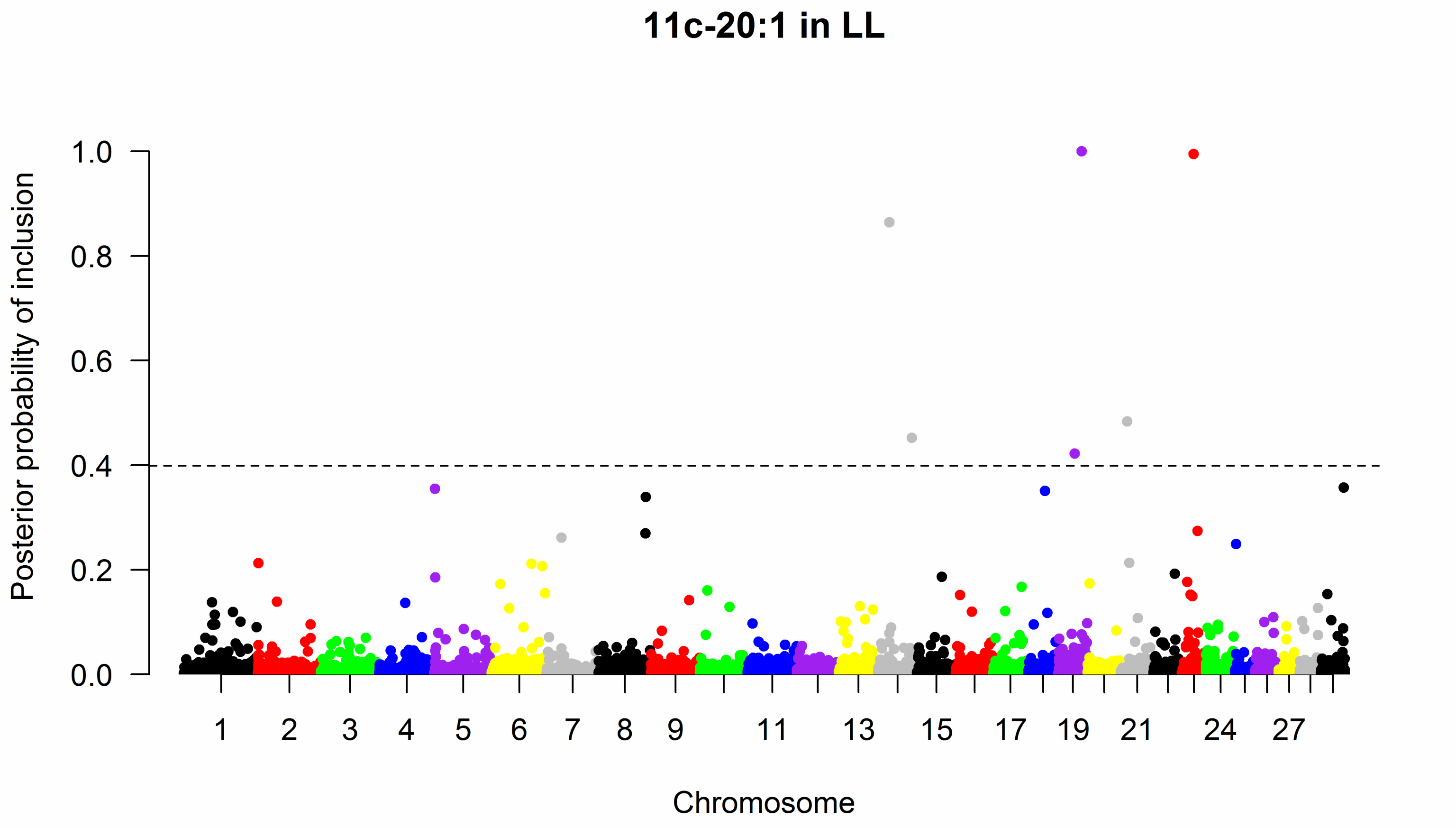
11c-20:1 in LL
Posterior probability of inclusion
1.0
0.8
0.6
0.4
0.2
0.0
1
2
3
4
5
6
7
8
9
11
13
15
17
19
21
24
27
Chromosome

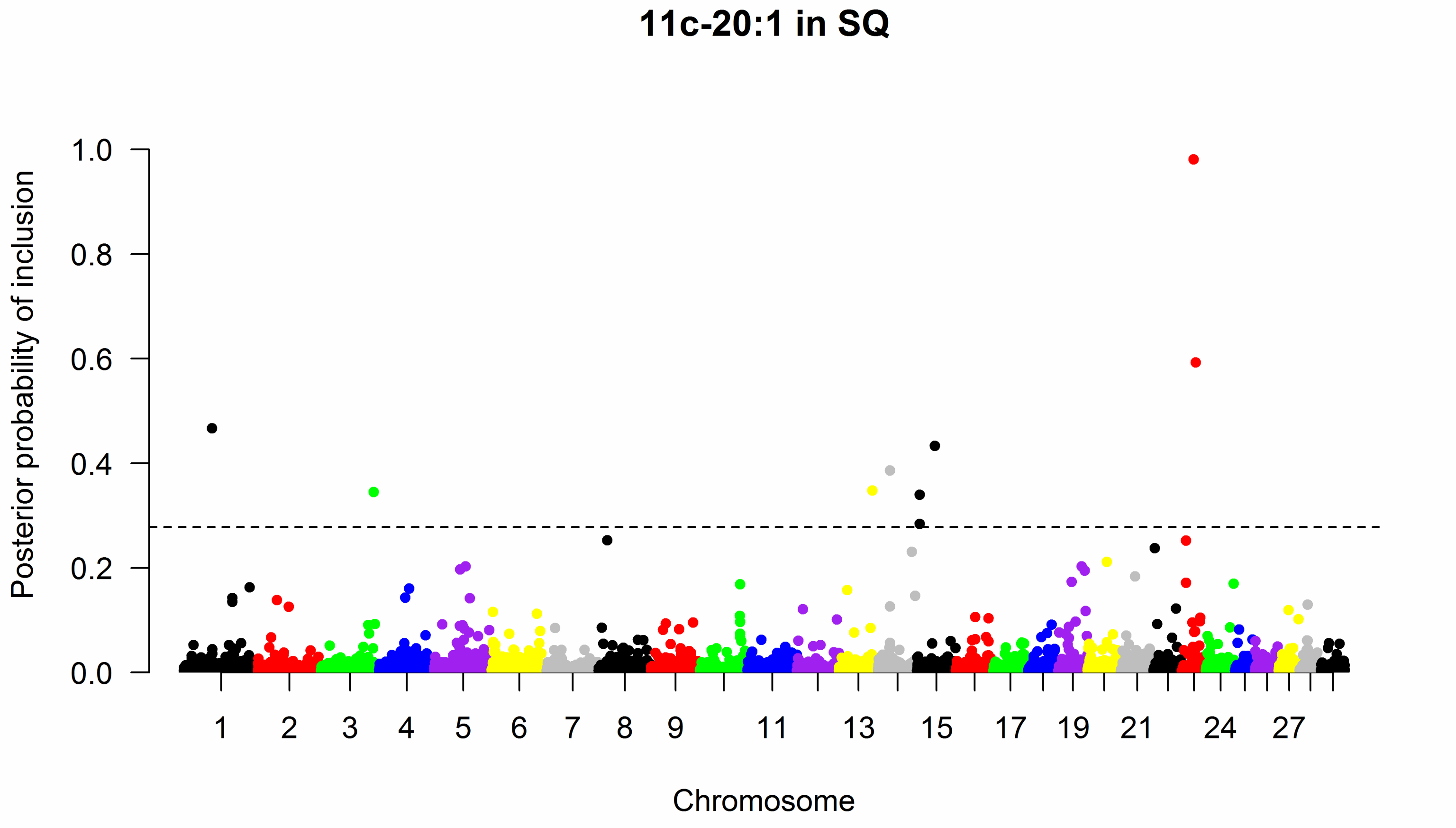
11c-20:1 in SQ
Posterior probability of inclusion
1.0
0.8
0.6
0.4
0.2
0.0
1
2
3
4
5
6
7
8
9
11
13
15
17
19
21
24
27
Chromosome

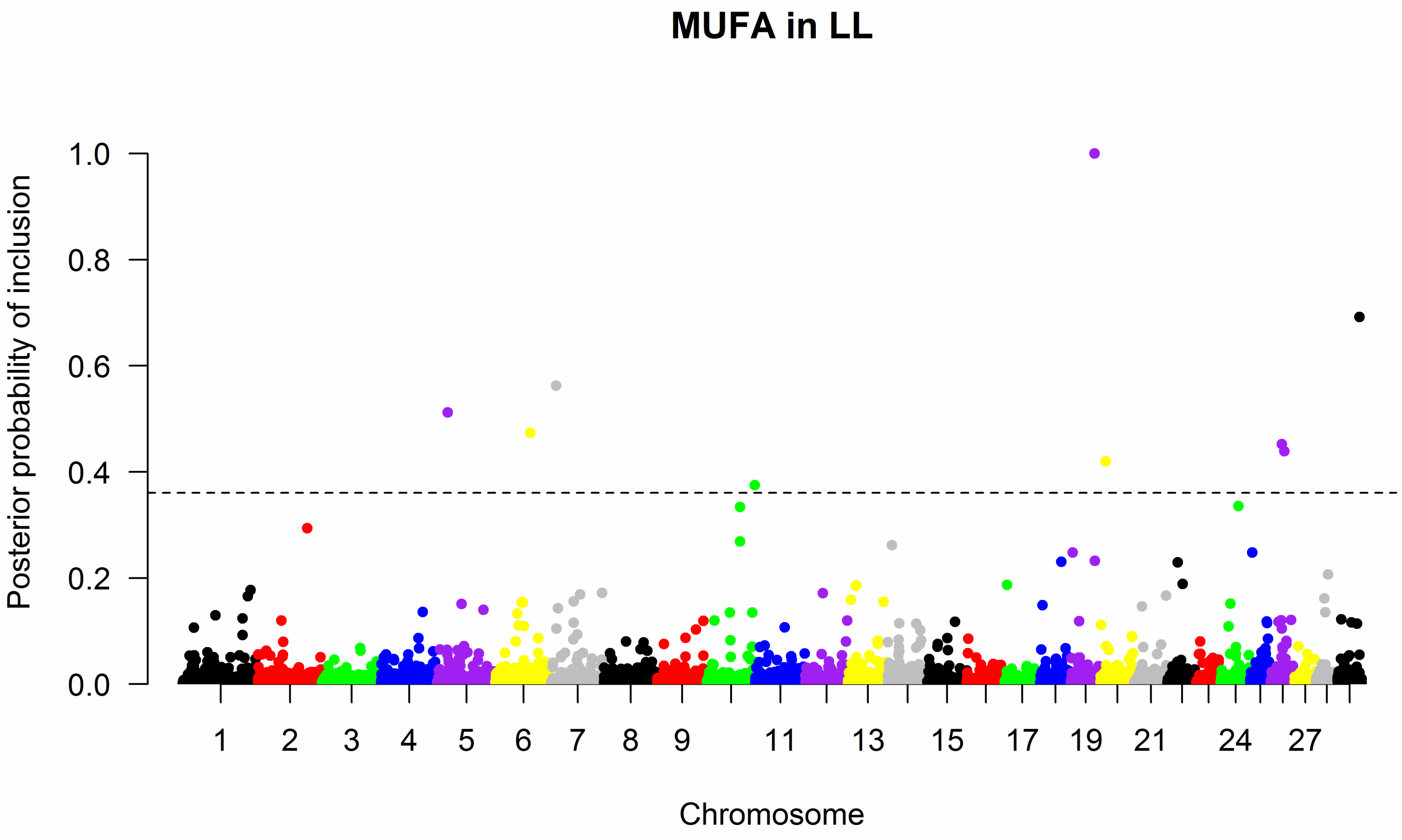
MUFA in LL
Posterior probability of inclusion
1.0
0.8
0.6
0.4
0.2
0.0
1
2
3
4
5
6
7
8
9
11
13
15
17
19
21
24
27
Chromosome

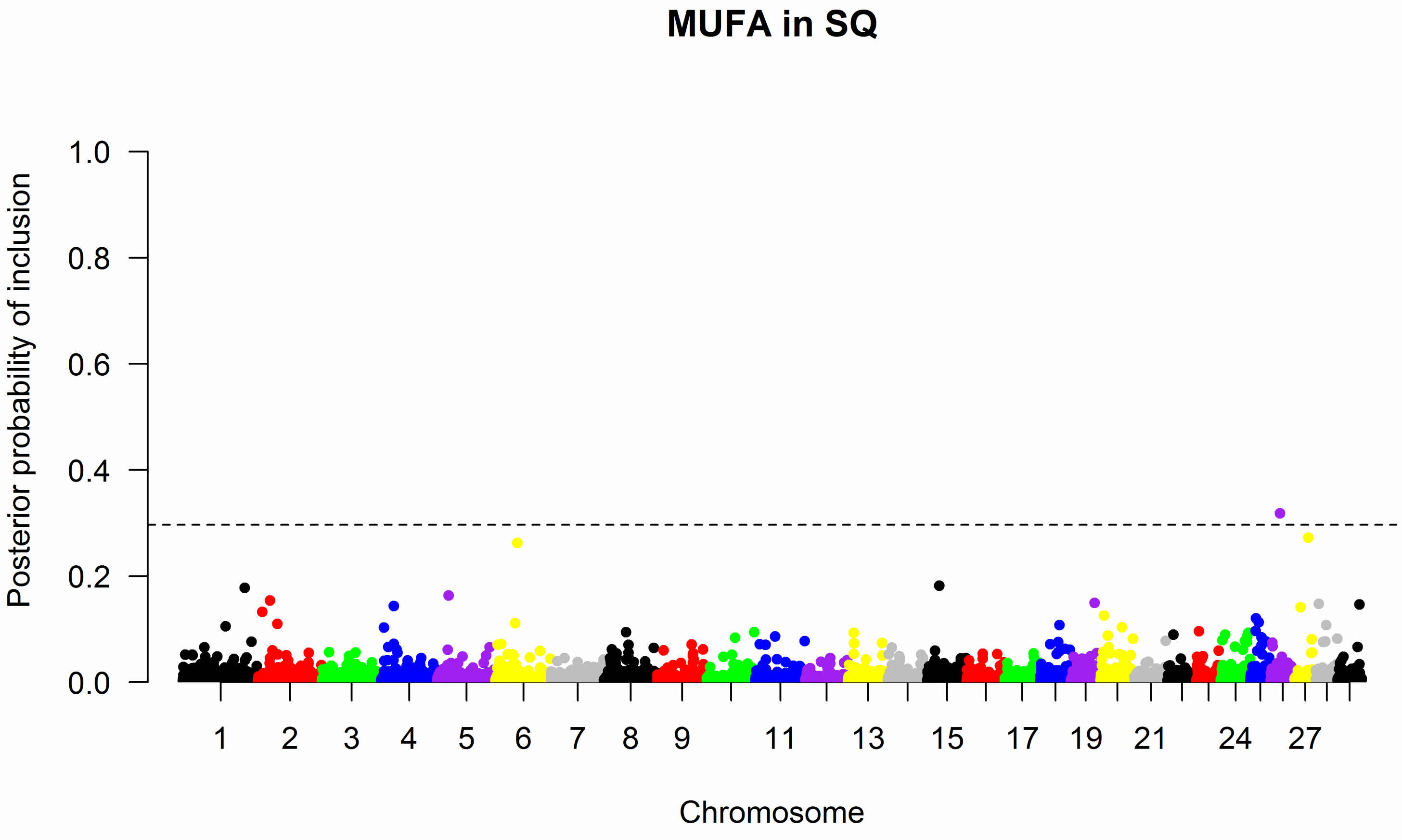
MUFA in SQ
Posterior probability of inclusion
1.0
0.8
0.6
0.4
0.2
0.0
1
2
3
4
5
6
7
8
9
11
13
15
17
19
21
24
27
Chromosome

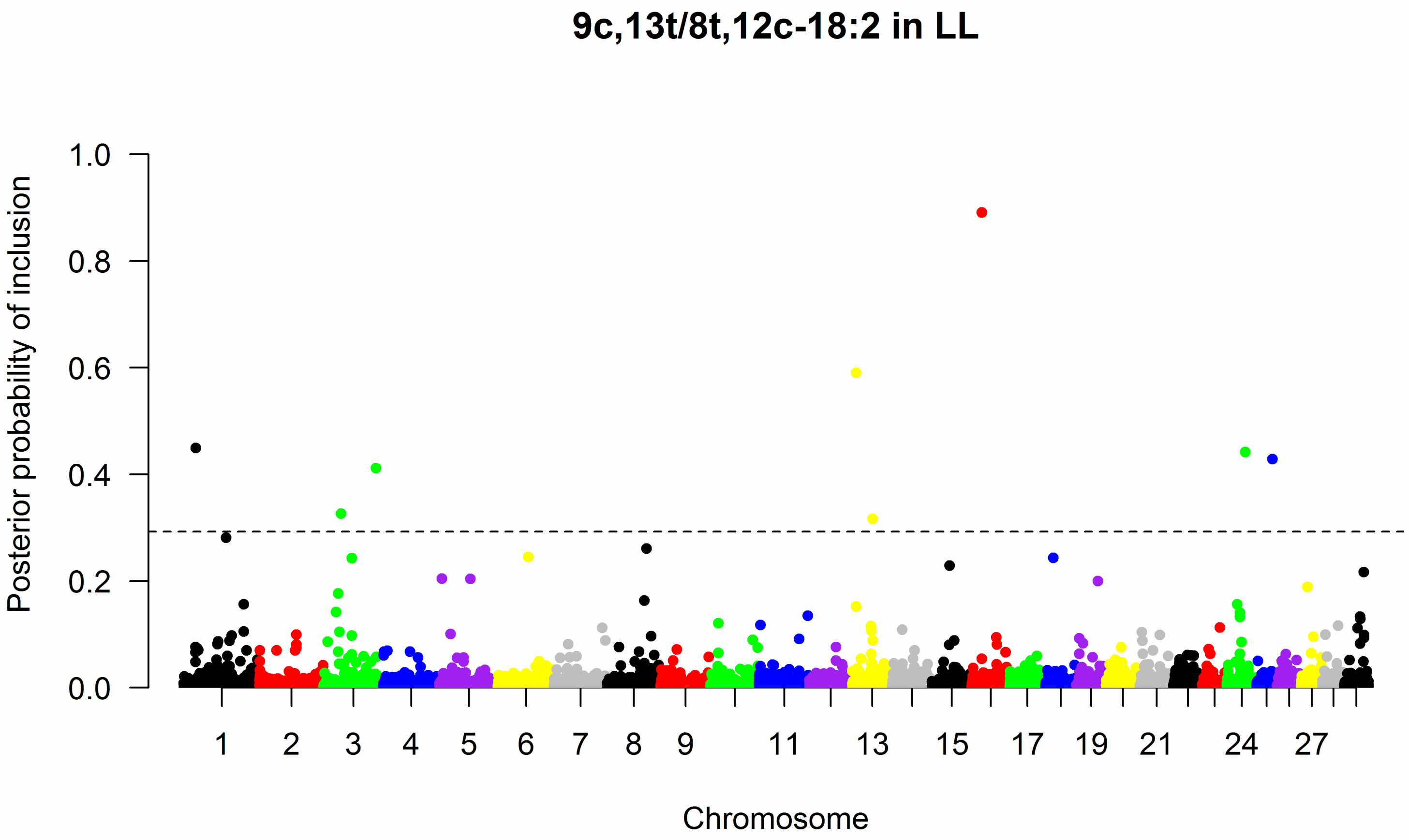
9c,13t/8t,12c-18:2 in LL
Posterior probability of inclusion
Chromosome
1.0
0.8
0.6
0.4
0.2
0.0
1
2
3
4
5
6
7
8
9
11
13
15
17
19
21
24
27

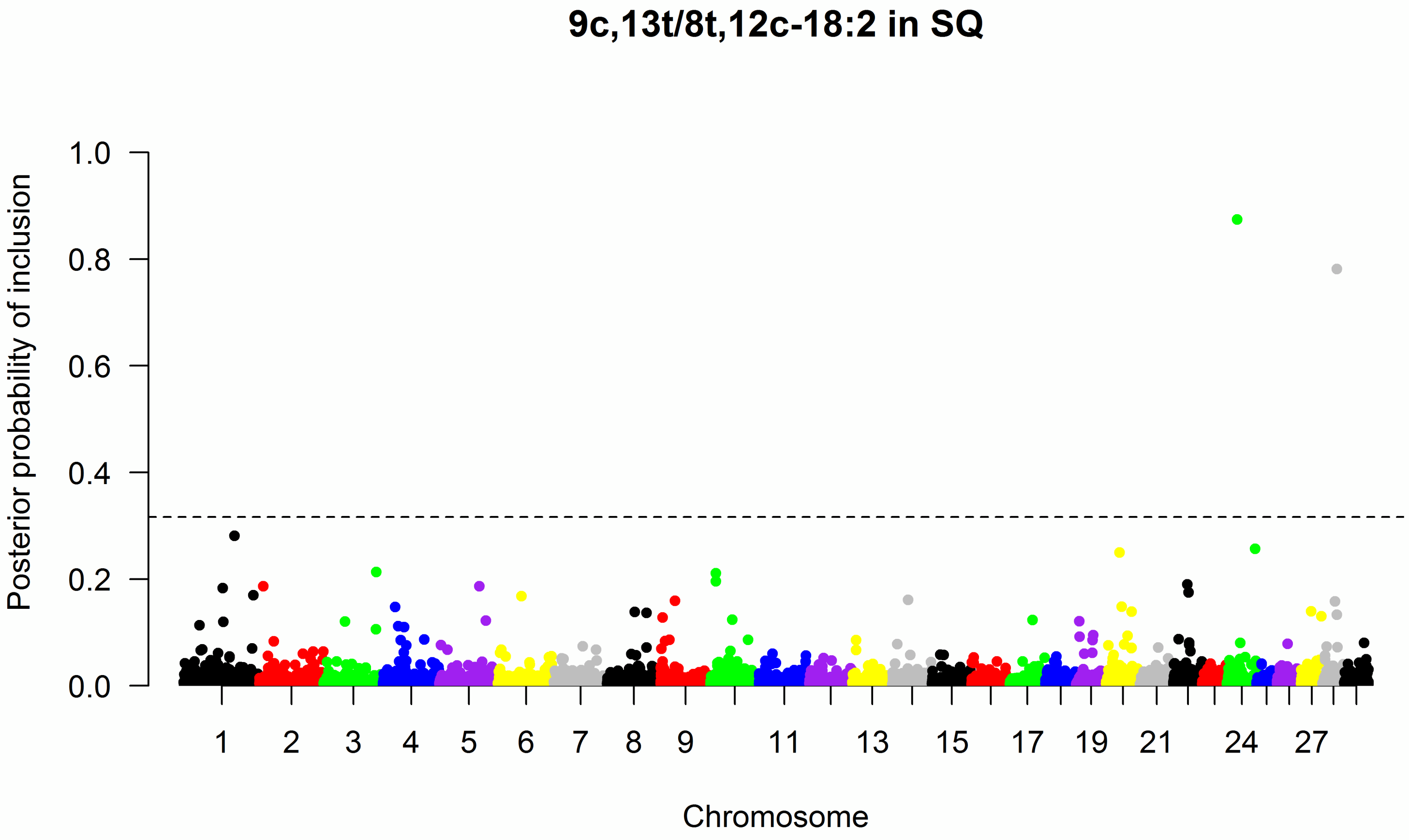
9c,13t/8t,12c-18:2 in SQ
Posterior probability of inclusion
Chromosome
1.0
0.8
0.6
0.4
0.2
0.0
1
2
3
4
5
6
7
8
9
11
13
15
17
19
21
24
27

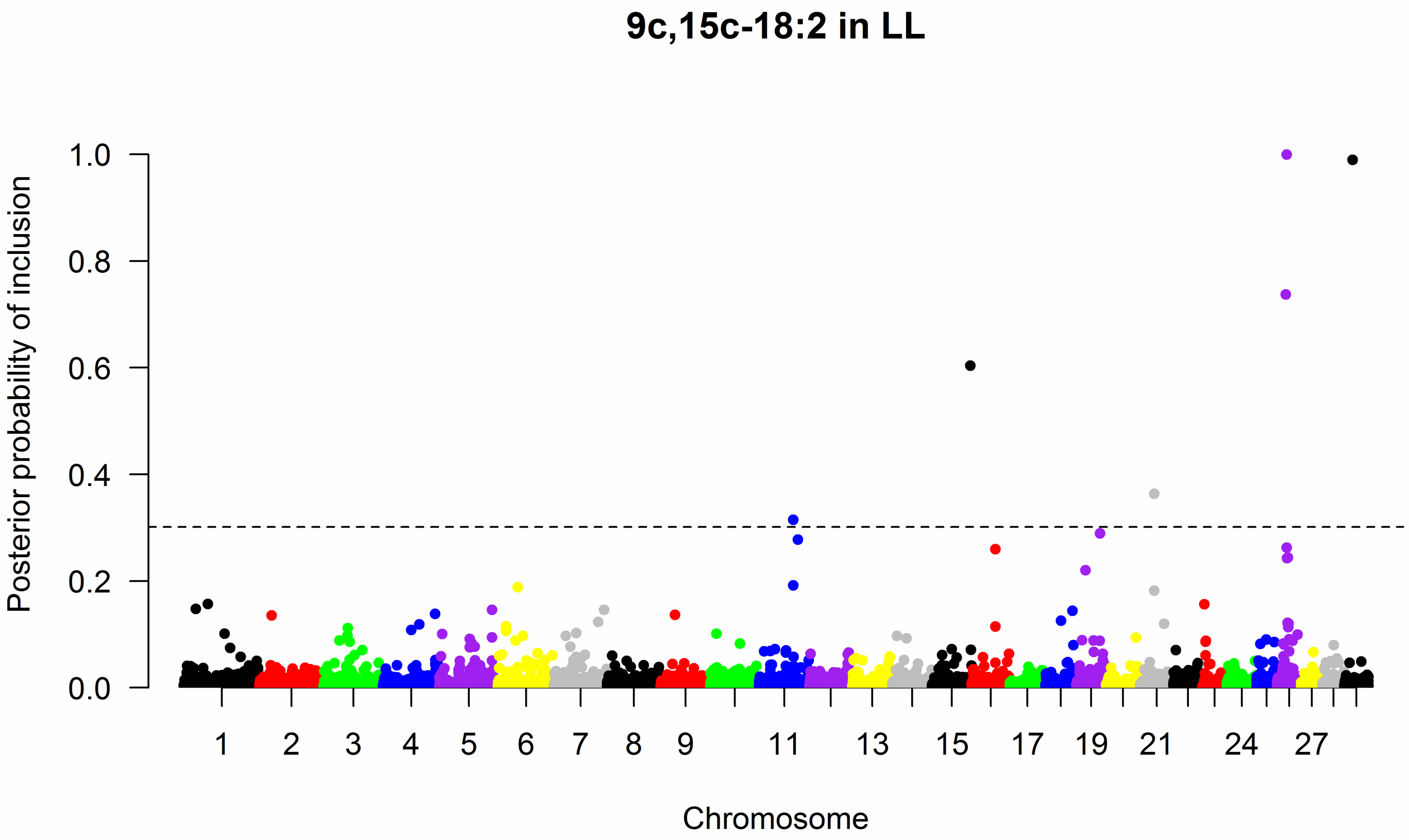
9c,15c-18:2 in LL
Posterior probability of inclusion
1.0
0.8
0.6
0.4
0.2
0.0
1
2
3
4
5
6
7
8
9
11
13
15
17
19
21
24
27
Chromosome

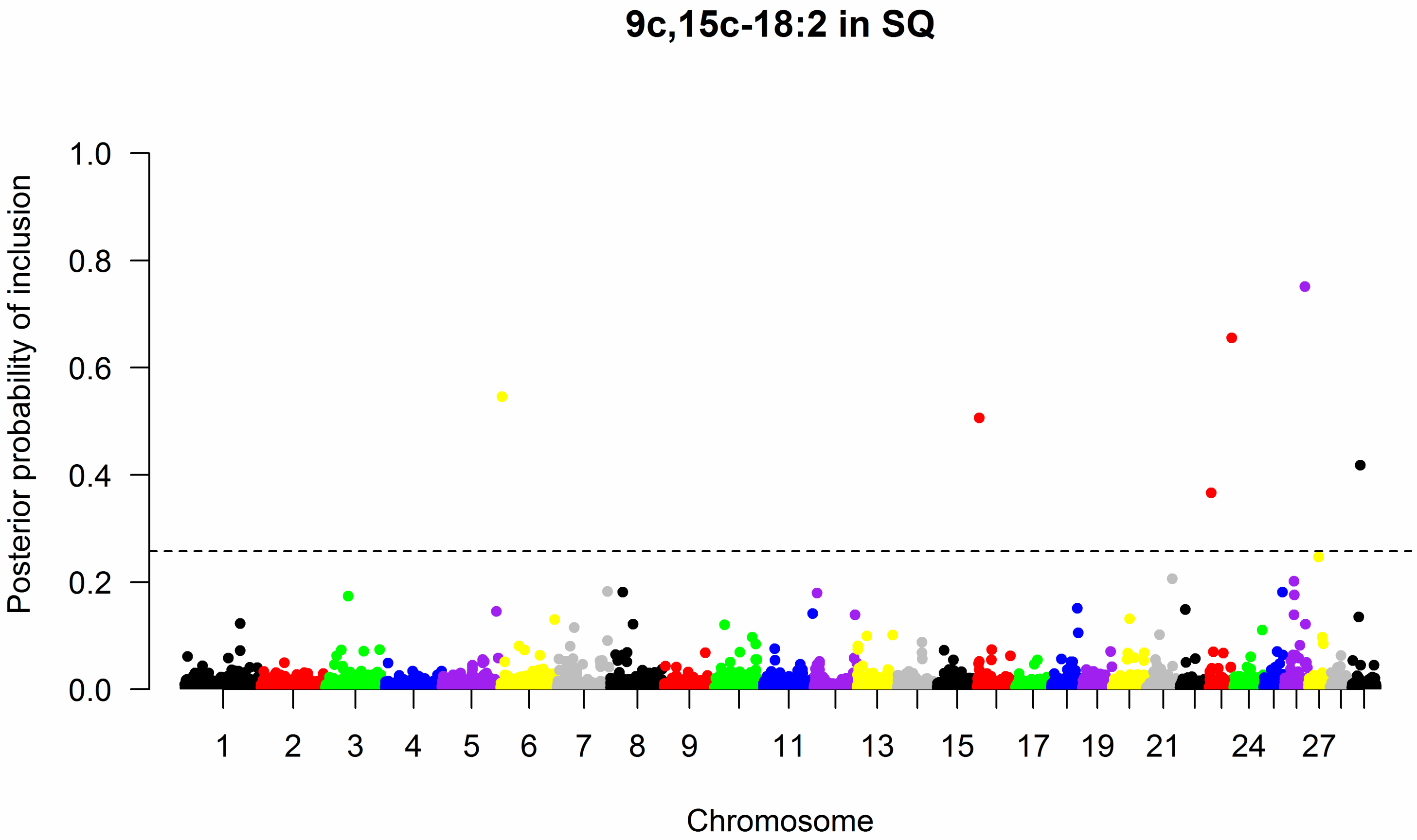
9c,15c-18:2 in SQ
Posterior probability of inclusion
1.0
0.8
0.6
0.4
0.2
0.0
1
2
3
4
5
6
7
8
9
11
13
15
17
19
21
24
27
Chromosome

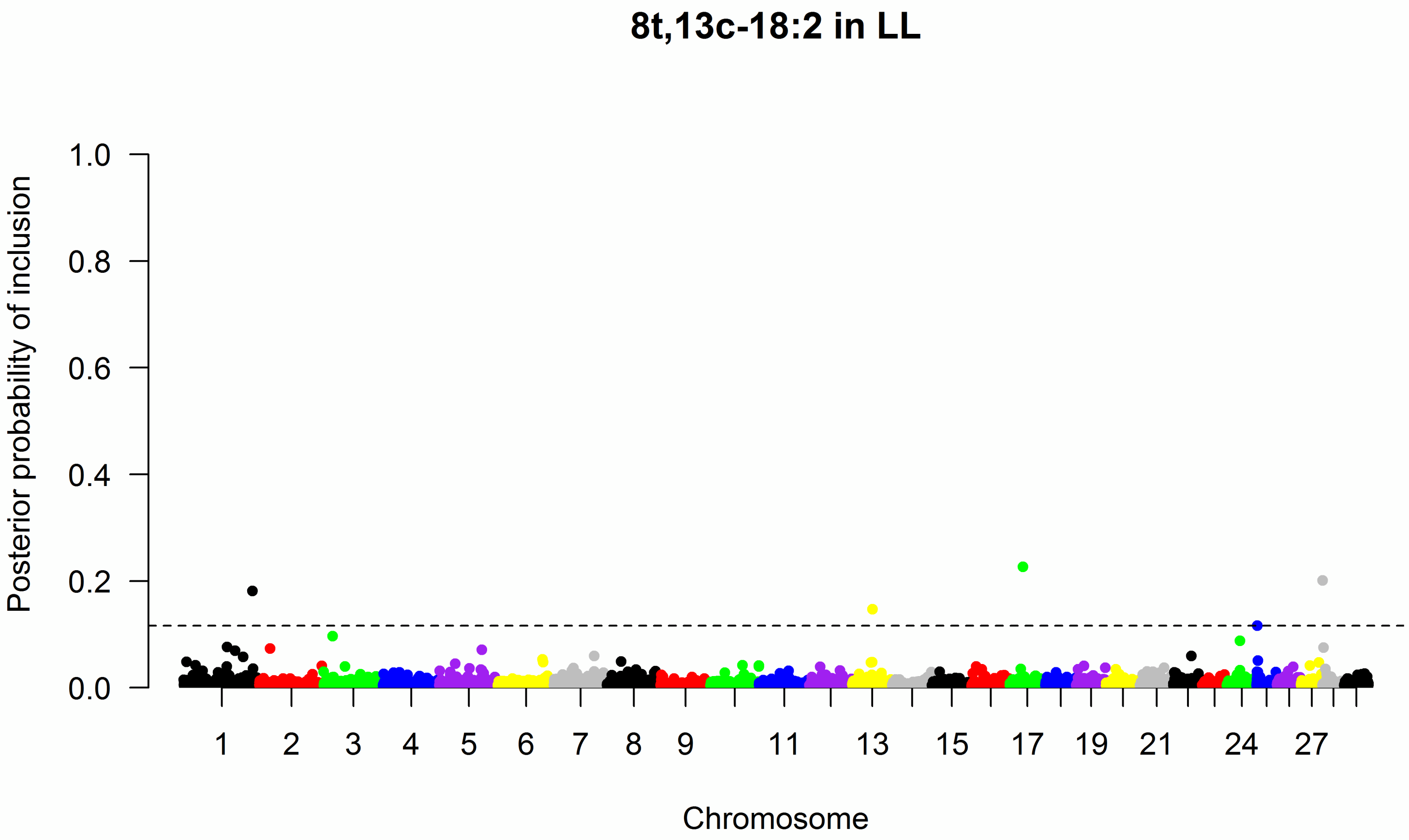

8t,13c-18:2 in LL
Posterior probability of inclusion
1.0
0.8
0.6
0.4
0.2
0.0
1 2 3 4 5 6 7 8 9 11 13 15 17 19 21 24 27
Chromosome

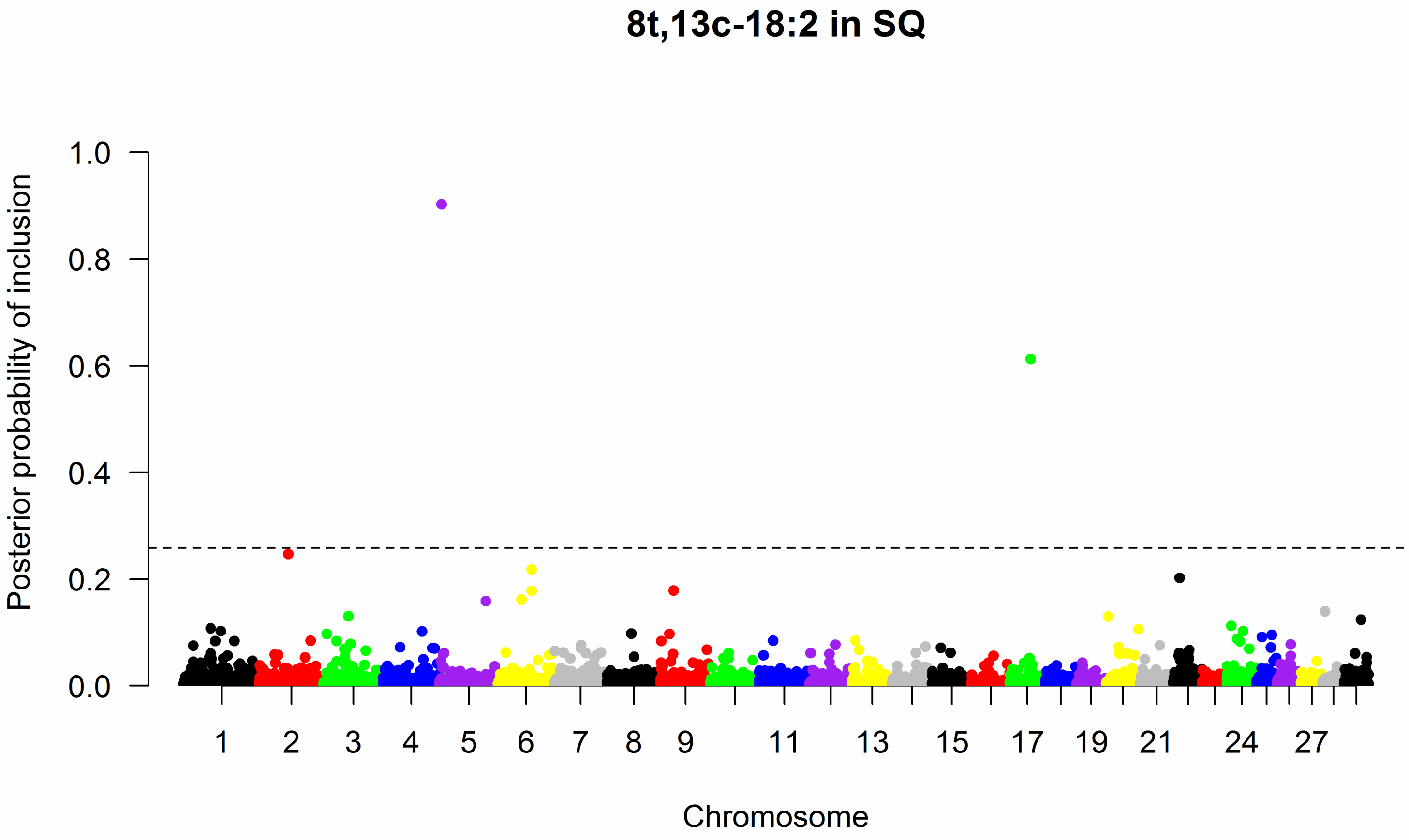

8t,13c-18:2 in SQ
Posterior probability of inclusion
1.0
0.8
0.6
0.4
0.2
0.0
1 2 3 4 5 6 7 8 9 11 13 15 17 19 21 24 27
Chromosome

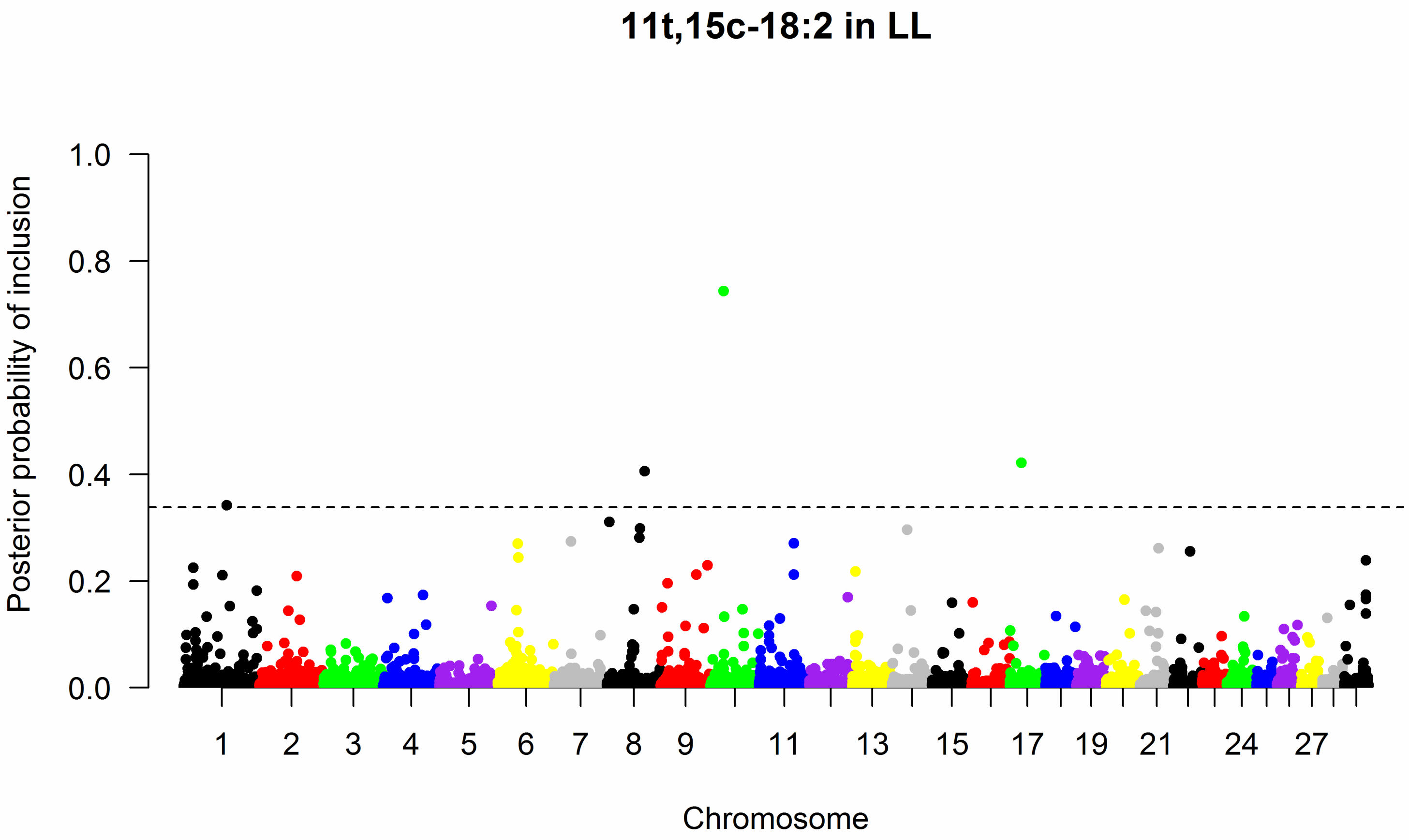

11t,15c-18:2 in LL
Posterior probability of inclusion
0.0
0.2
0.4
0.6
0.8
1.0
1
2
3
4
5
6
7
8
9
11
13
15
17
19
21
24
27
Chromosome

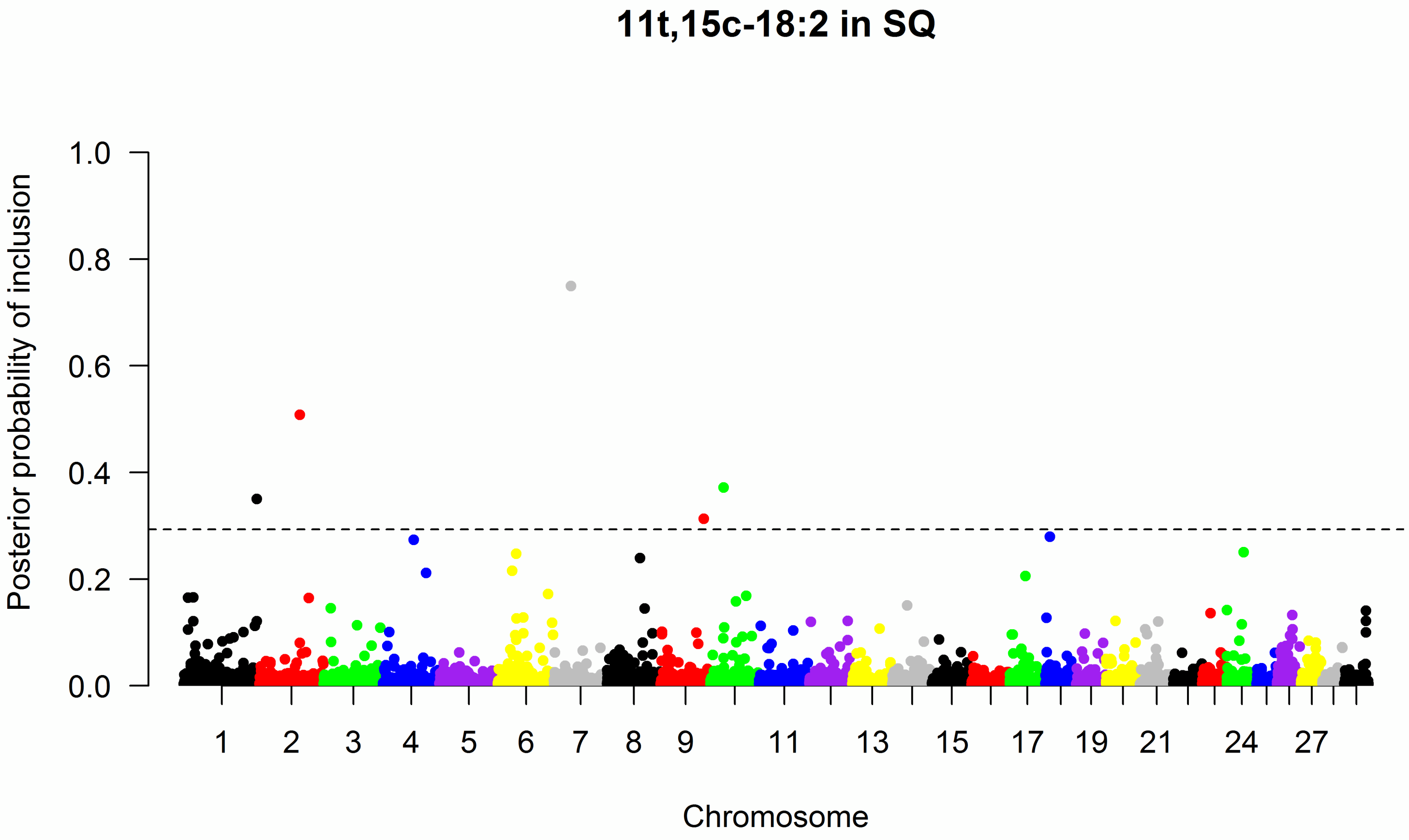

11t,15c-18:2 in SQ
Posterior probability of inclusion
0.0
0.2
0.4
0.6
0.8
1.0
1
2
3
4
5
6
7
8
9
11
13
15
17
19
21
24
27
Chromosome

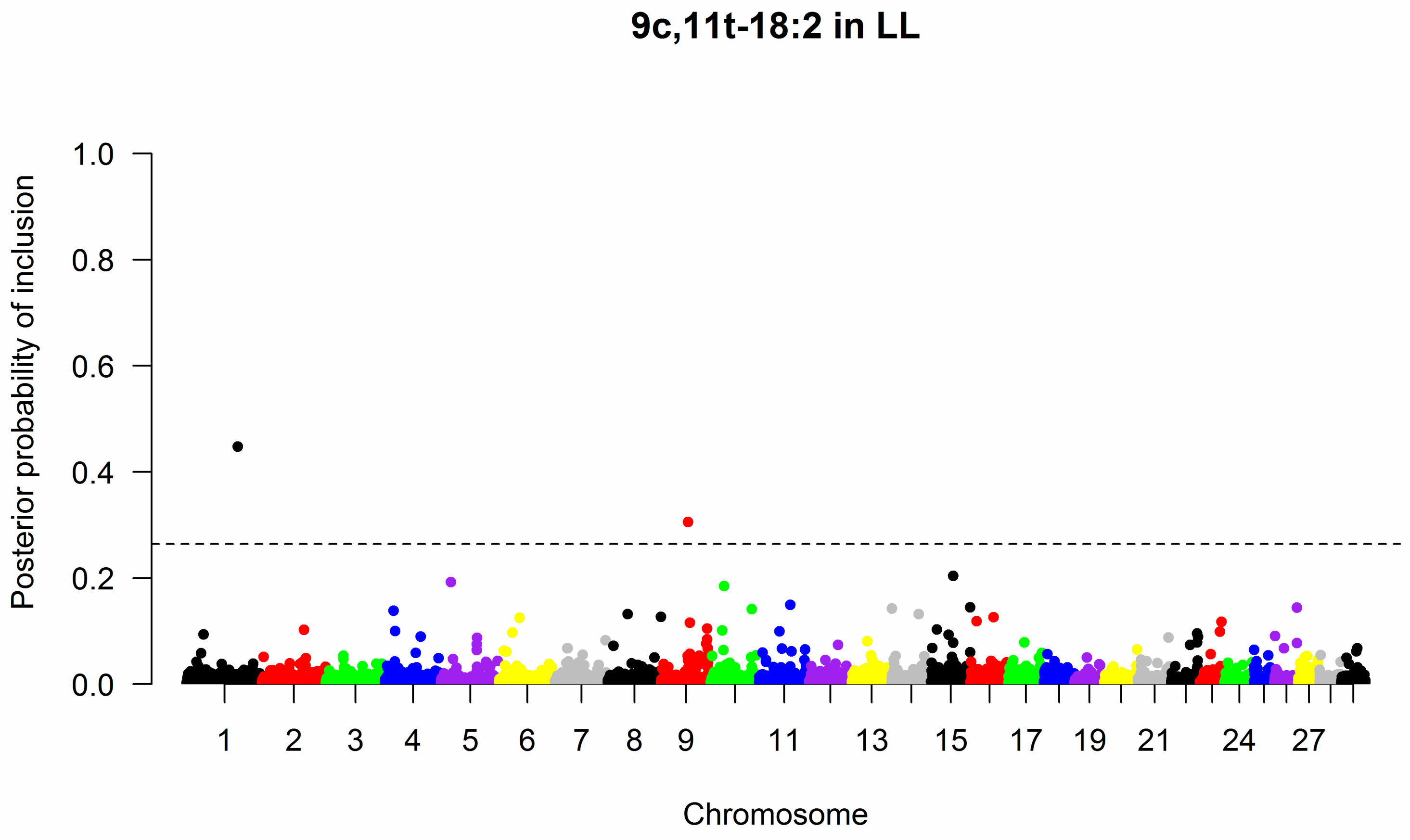
9c,11t-18:2 in LL
Posterior probability of inclusion
1.0
0.8
0.6
0.4
0.2
0.0
1 2 3 4 5 6 7 8 9 11 13 15 17 19 21 24 27
Chromosome

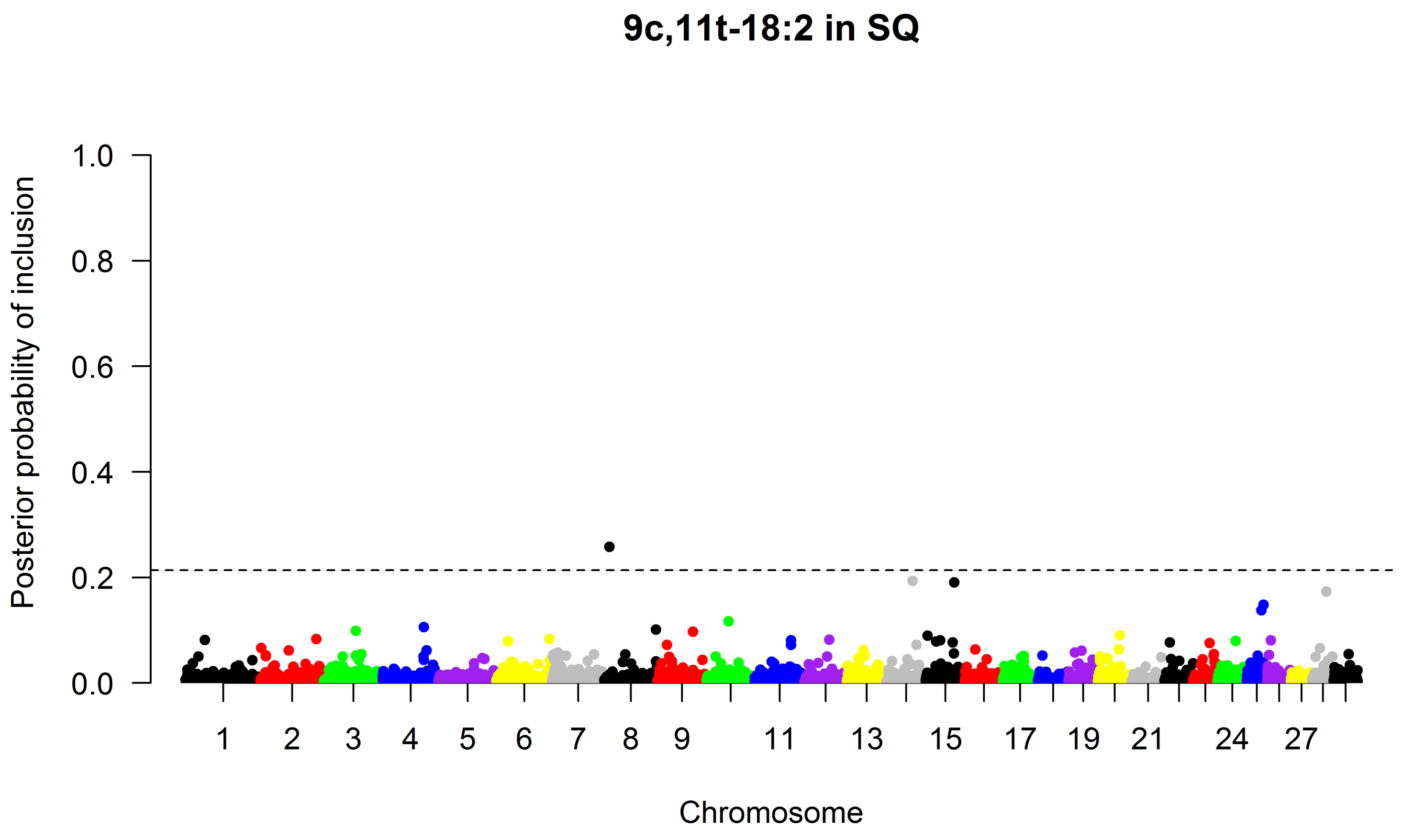
9c,11t-18:2 in SQ
Posterior probability of inclusion
1.0
0.8
0.6
0.4
0.2
0.0
1 2 3 4 5 6 7 8 9 11 13 15 17 19 21 24 27
Chromosome

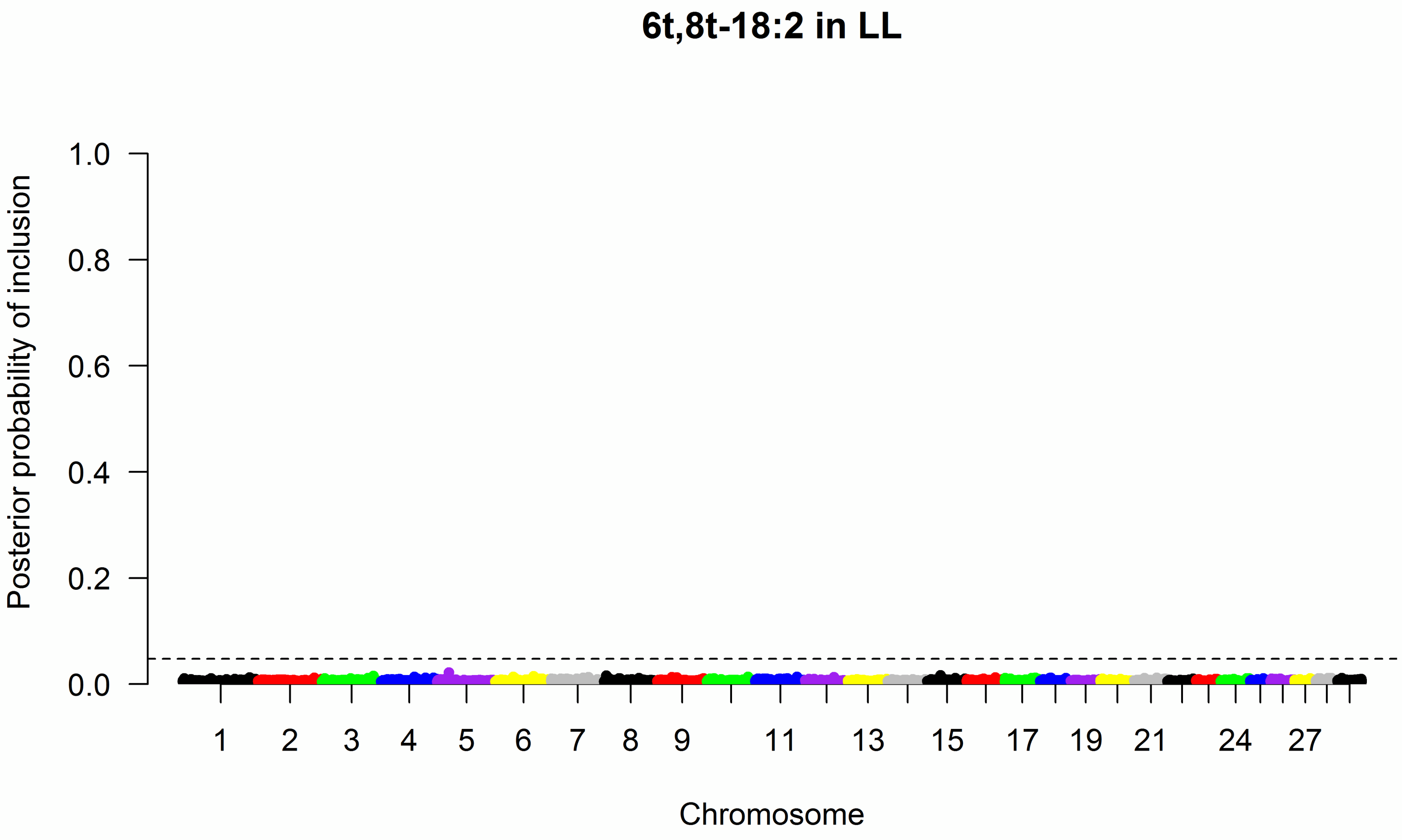
6t,8t-18:2 in LL
Posterior probability of inclusion
1.0
0.8
0.6
0.4
0.2
0.0
1 2 3 4 5 6 7 8 9 11 13 15 17 19 21 24 27
Chromosome

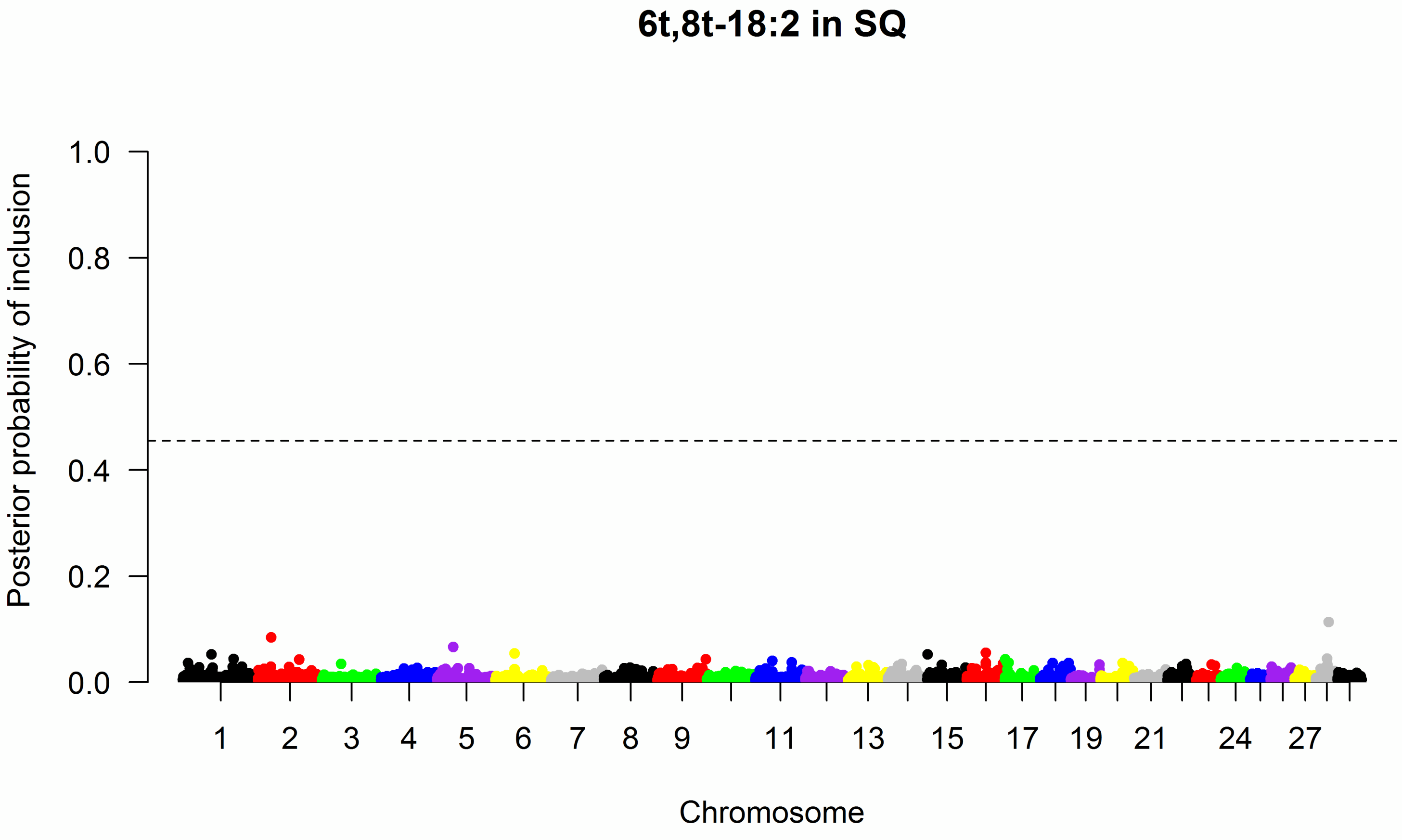
6t,8t-18:2 in SQ
Posterior probability of inclusion
1.0
0.8
0.6
0.4
0.2
0.0
1 2 3 4 5 6 7 8 9 11 13 15 17 19 21 24 27
Chromosome

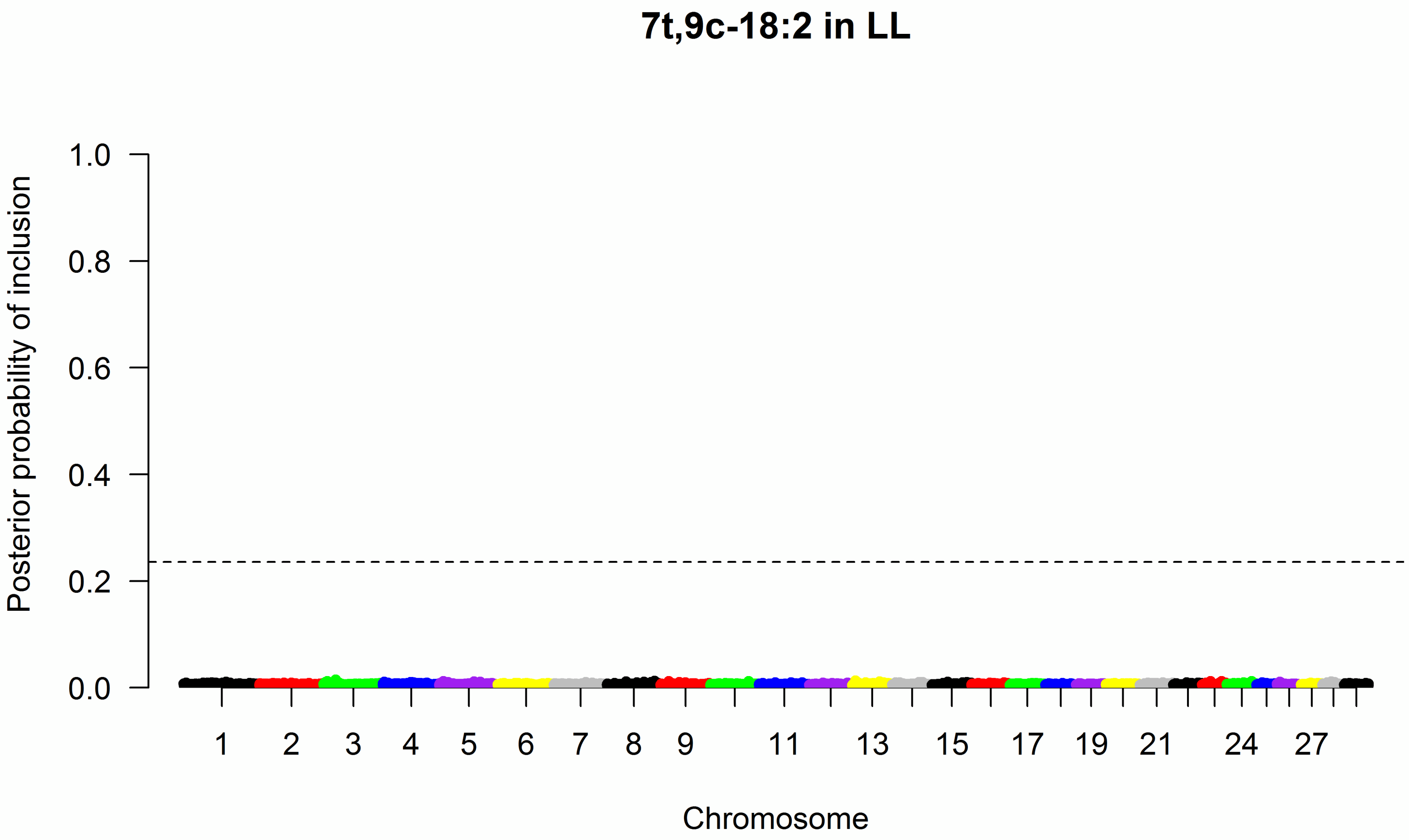
7t,9c-18:2 in LL
Posterior probability of inclusion
1.0
0.8
0.6
0.4
0.2
0.0
1
2
3
4
5
6
7
8
9
11
13
15
17
19
21
24
27
Chromosome

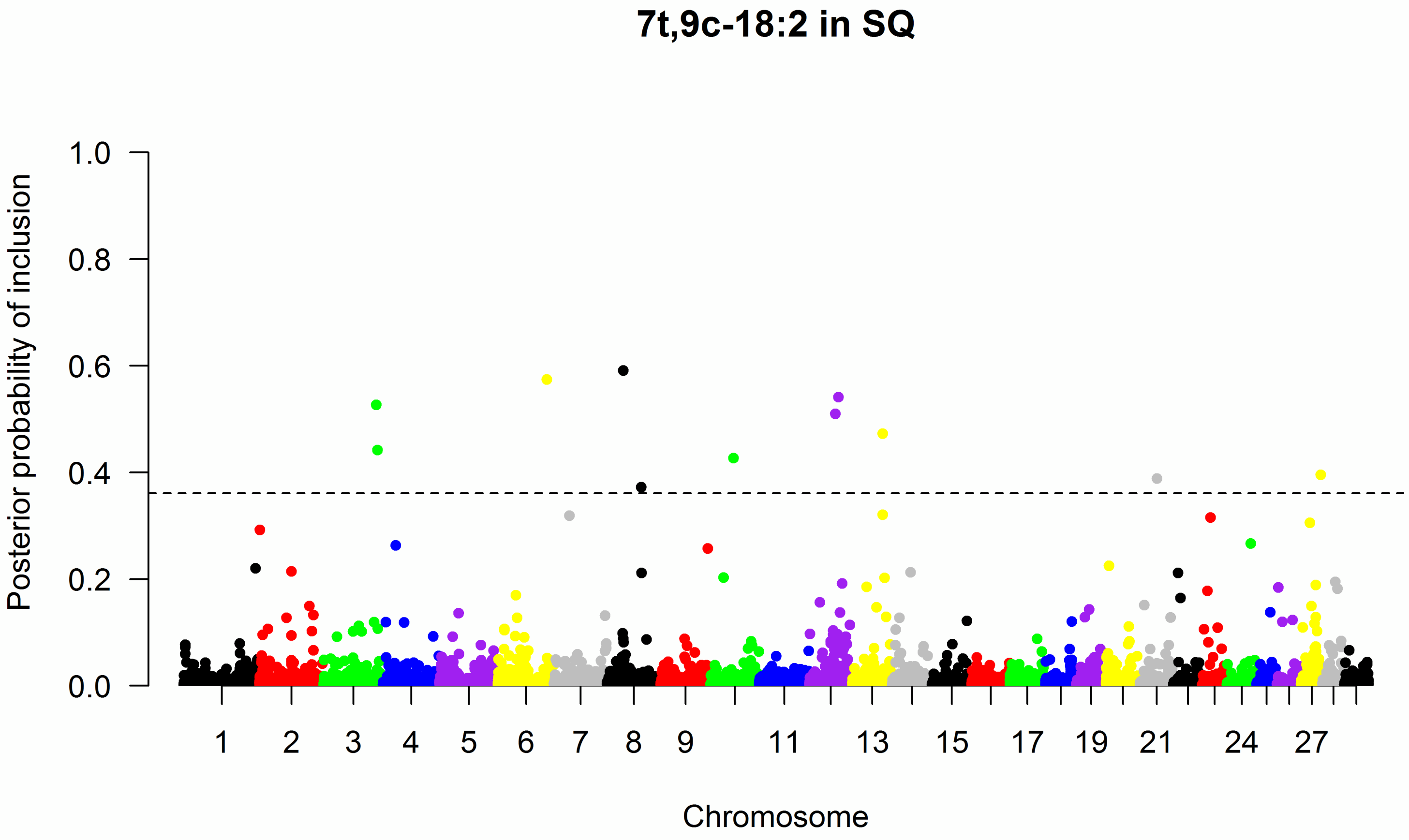
7t,9c-18:2 in SQ
Posterior probability of inclusion
1.0
0.8
0.6
0.4
0.2
0.0
1
2
3
4
5
6
7
8
9
11
13
15
17
19
21
24
27
Chromosome

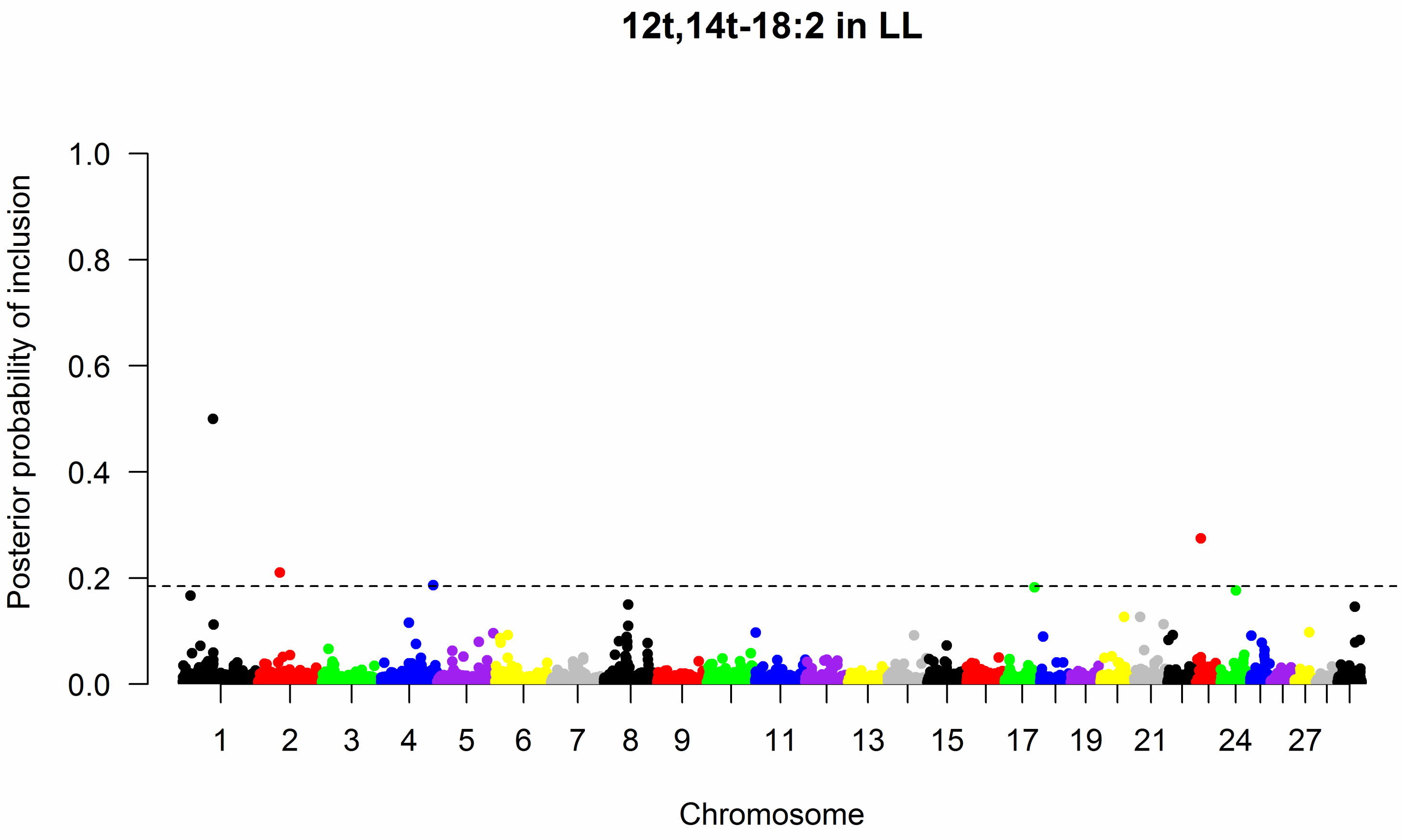
12t,14t-18:2 in LL
Posterior probability of inclusion
0.0
0.2
0.4
0.6
0.8
1.0
1
2
3
4
5
6
7
8
9
11
13
15
17
19
21
24
27
Chromosome

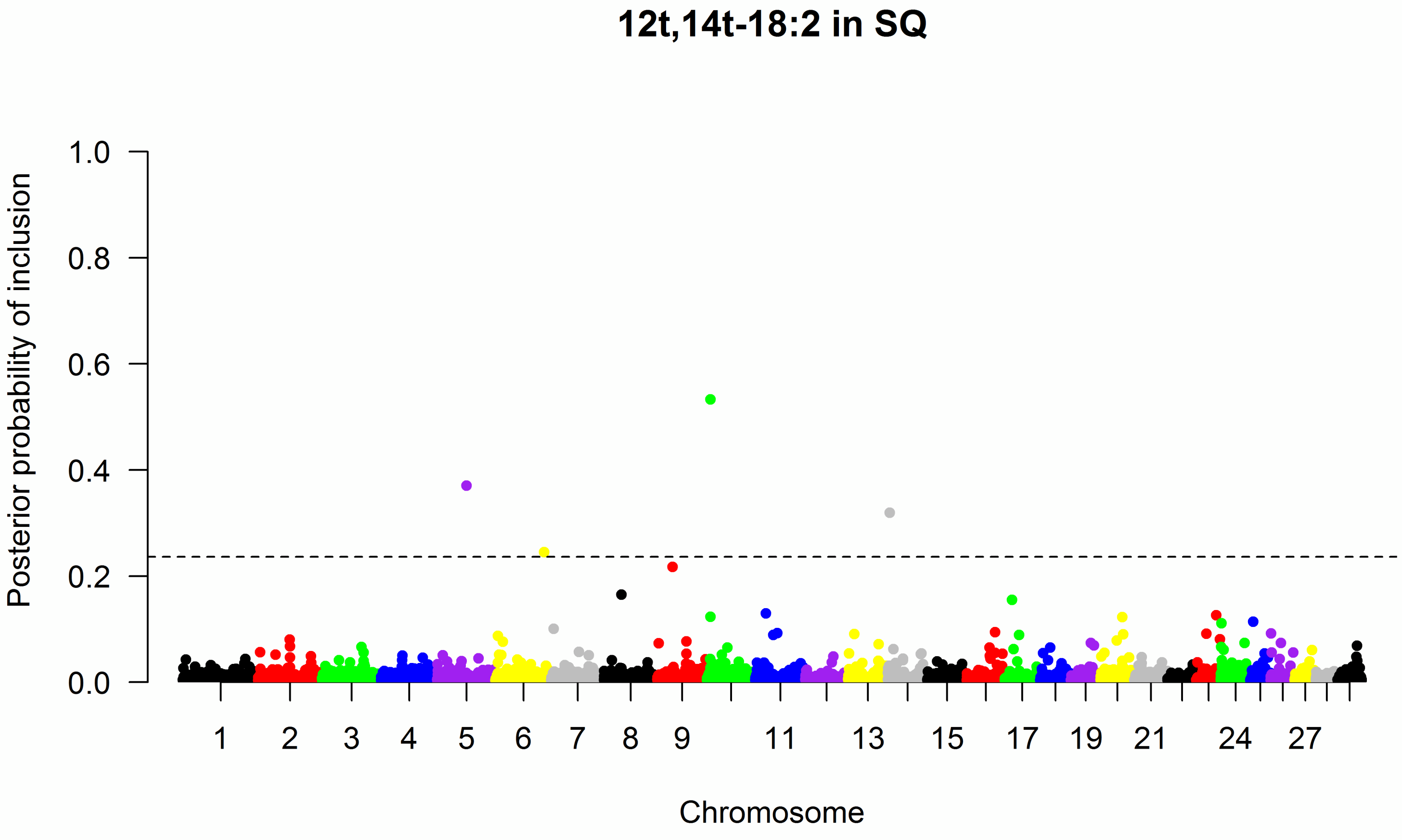
12t,14t-18:2 in SQ
Posterior probability of inclusion
0.0
0.2
0.4
0.6
0.8
1.0
1
2
3
4
5
6
7
8
9
11
13
15
17
19
21
24
27
Chromosome

11t,13t-18:2 in LL

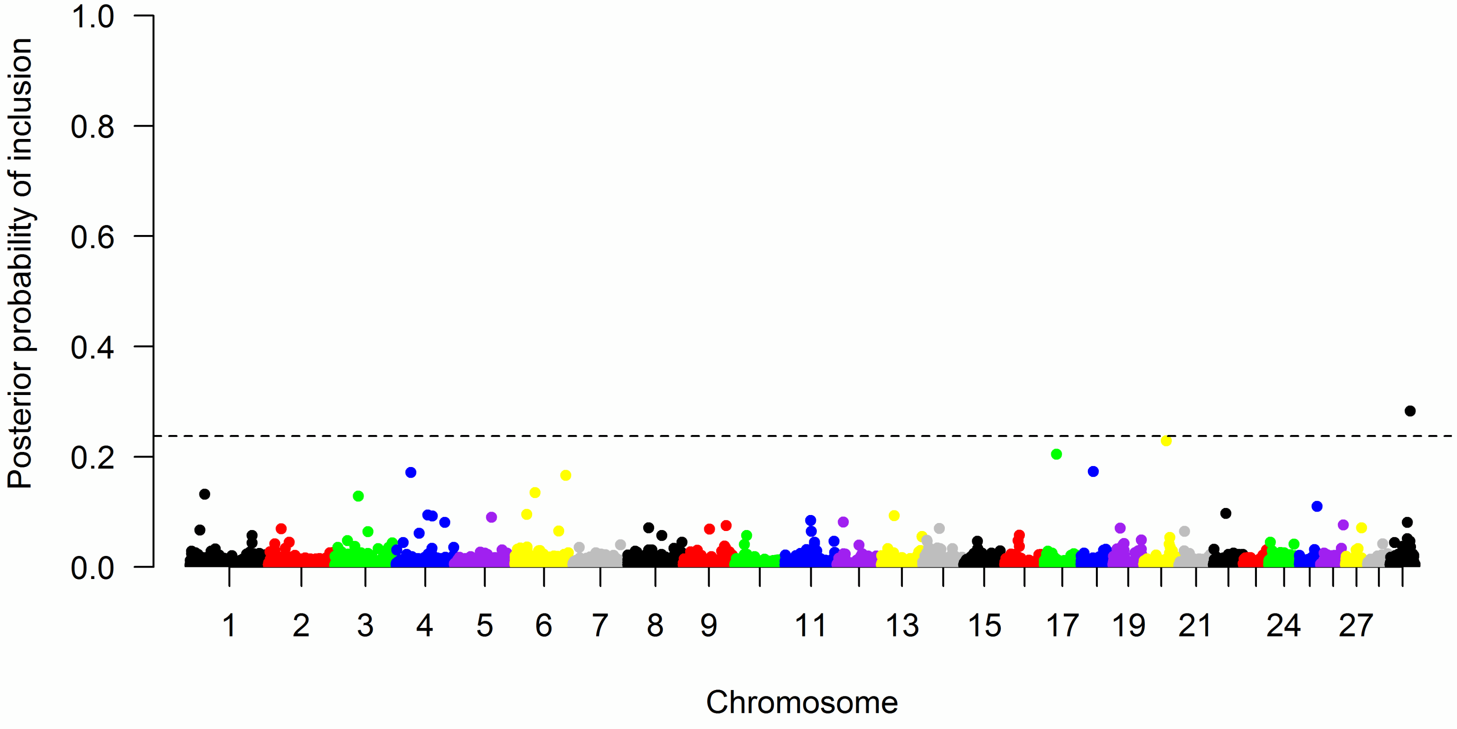

11t,13t-18:2 in SQ

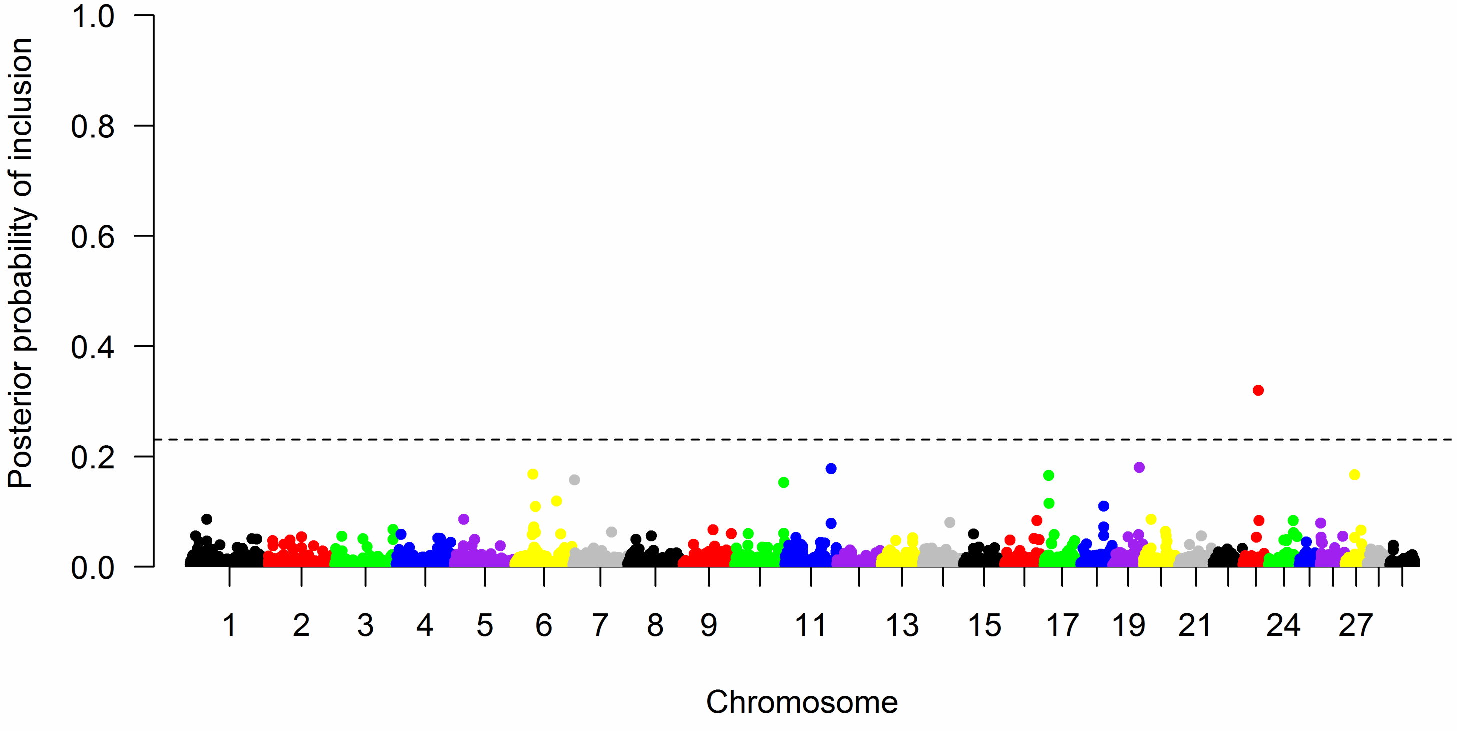

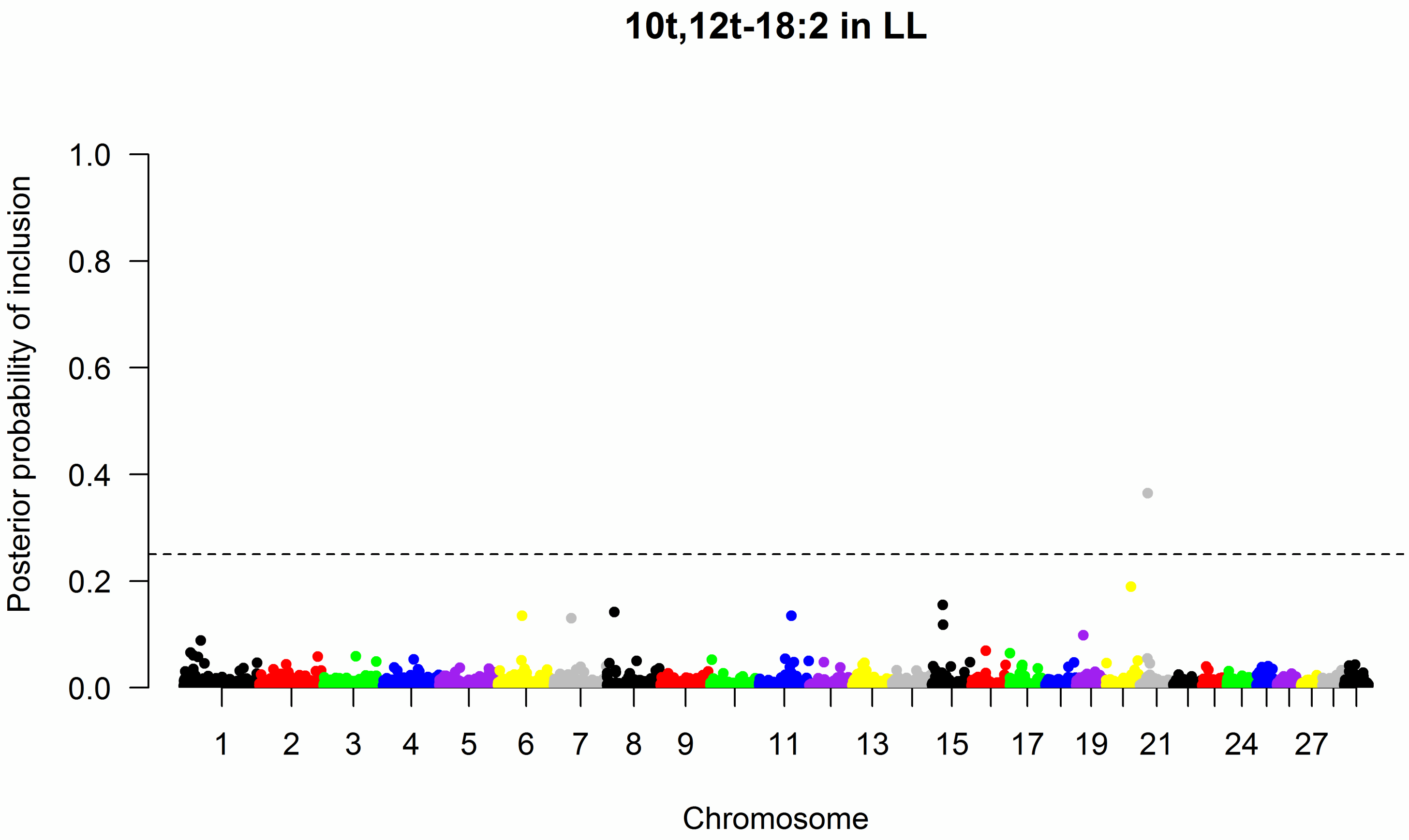
10t,12t-18:2 in LL
Posterior probability of inclusion
0.0
0.2
0.4
0.6
0.8
1.0
1
2
3
4
5
6
7
8
9
11
13
15
17
19
21
24
27
Chromosome

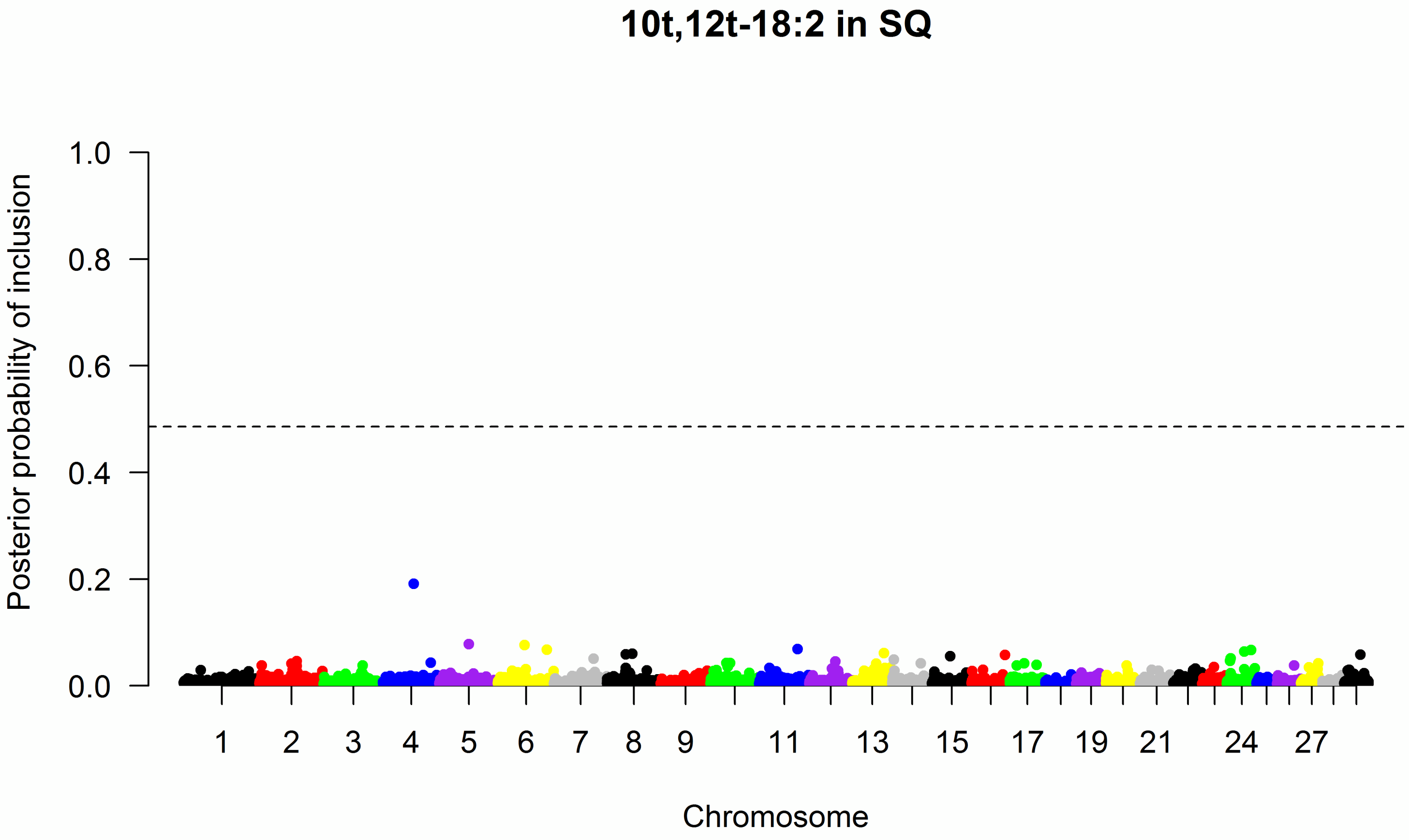
10t,12t-18:2 in SQ
Posterior probability of inclusion
0.0
0.2
0.4
0.6
0.8
1.0
1
2
3
4
5
6
7
8
9
11
13
15
17
19
21
24
27
Chromosome

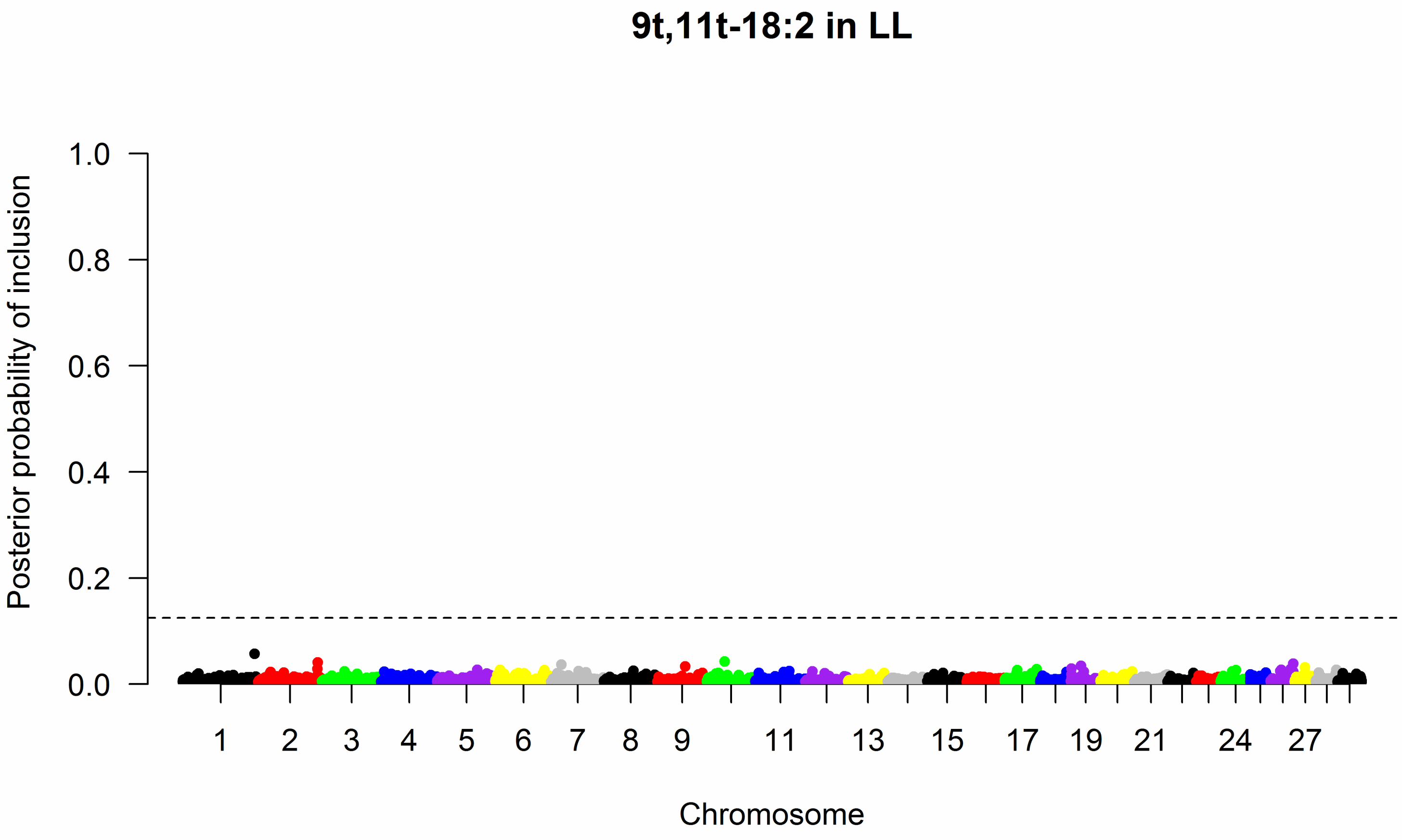
9t,11t-18:2 in LL
Posterior probability of inclusion
1.0
0.8
0.6
0.4
0.2
0.0
1
2
3
4
5
6
7
8
9
11
13
15
17
19
21
24
27
Chromosome

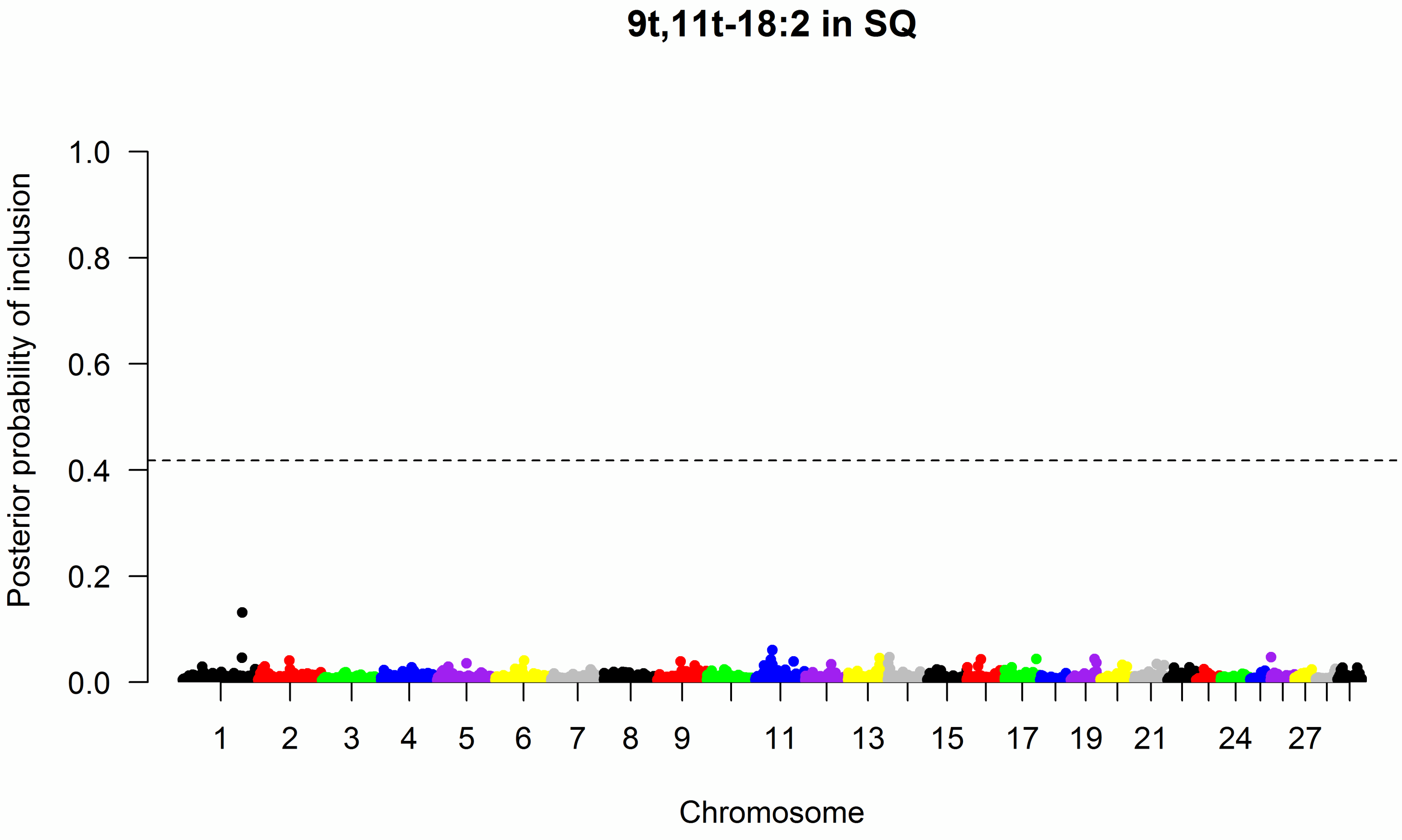
9t,11t-18:2 in SQ
Posterior probability of inclusion
1.0
0.8
0.6
0.4
0.2
0.0
1
2
3
4
5
6
7
8
9
11
13
15
17
19
21
24
27
Chromosome

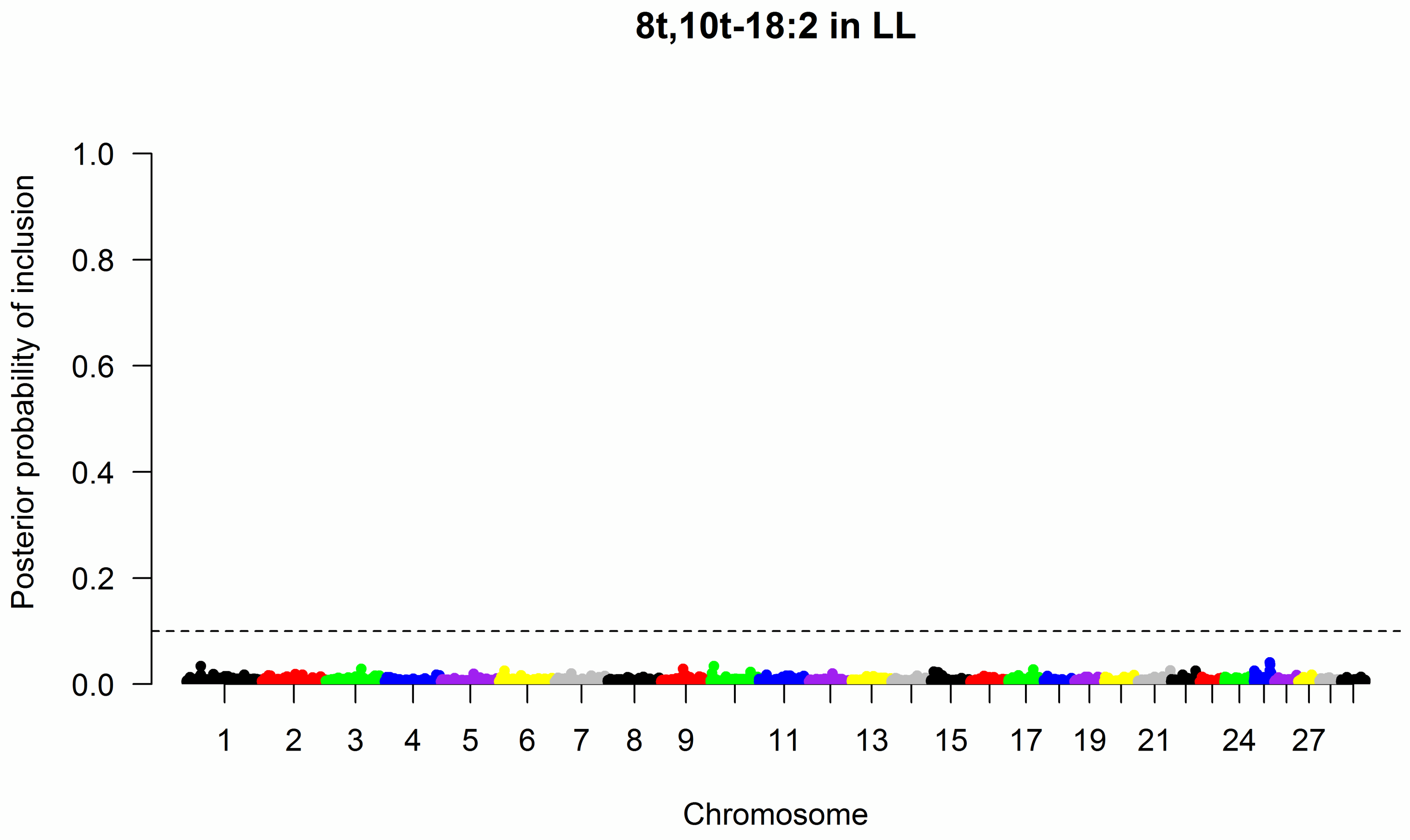

8t,10t-18:2 in LL
Posterior probability of inclusion
1.0
0.8
0.6
0.4
0.2
0.0
1
2
3
4
5
6
7
8
9
11
13
15
17
19
21
24
27
Chromosome

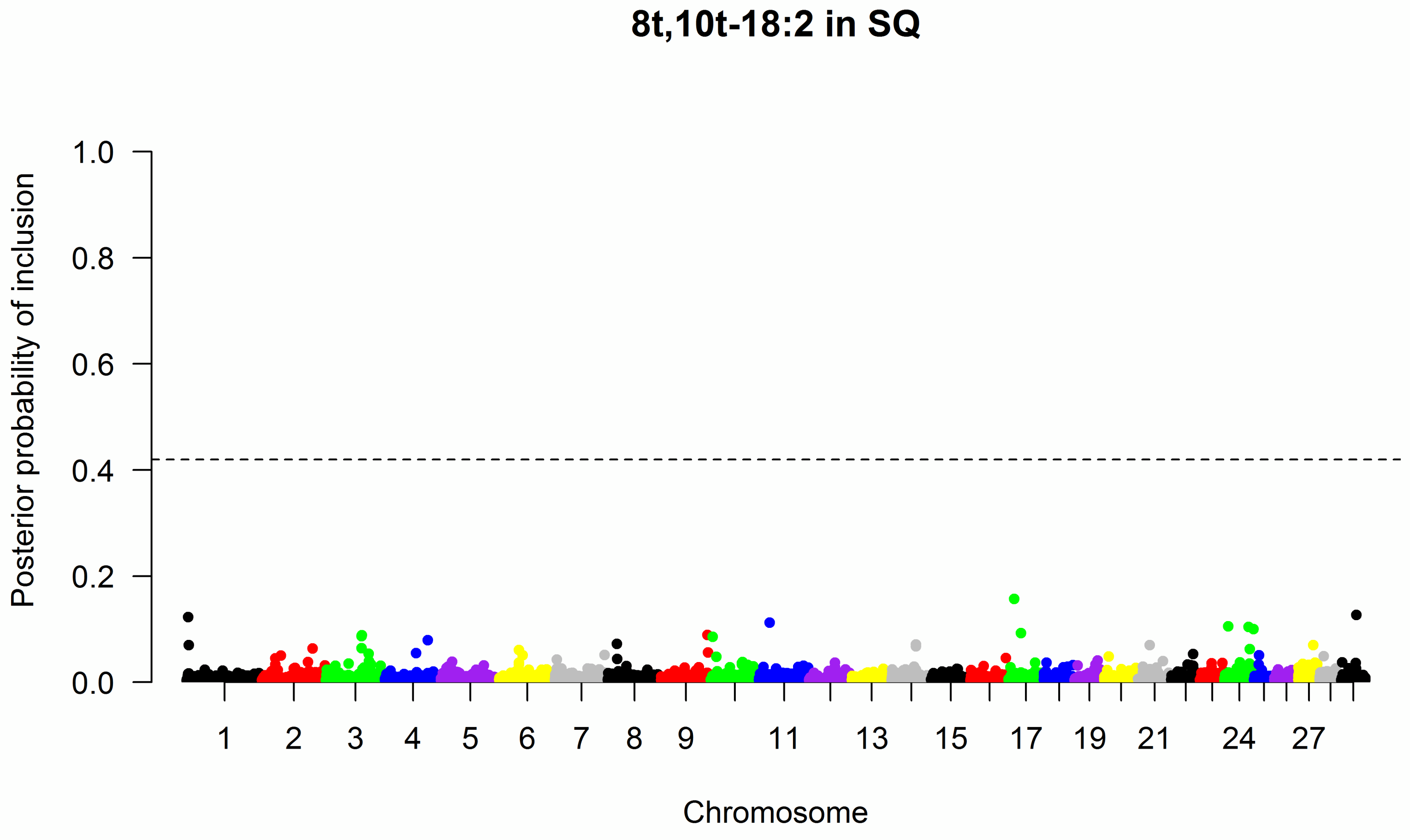

8t,10t-18:2 in SQ
Posterior probability of inclusion
1.0
0.8
0.6
0.4
0.2
0.0
1
2
3
4
5
6
7
8
9
11
13
15
17
19
21
24
27
Chromosome

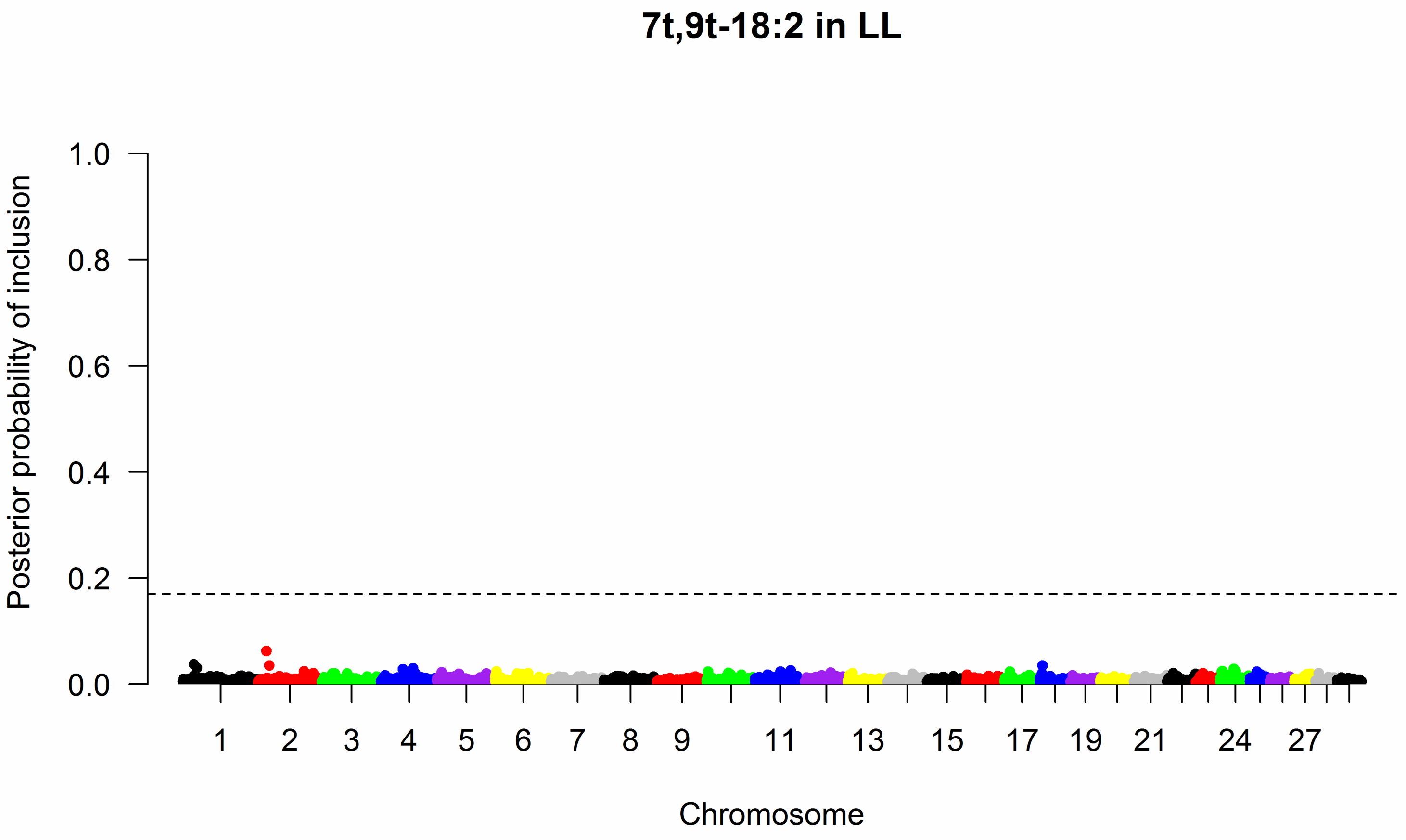

7t,9t-18:2 in LL
Posterior probability of inclusion
1.0
0.8
0.6
0.4
0.2
0.0
1
2
3
4
5
6
7
8
9
11
13
15
17
19
21
24
27
Chromosome

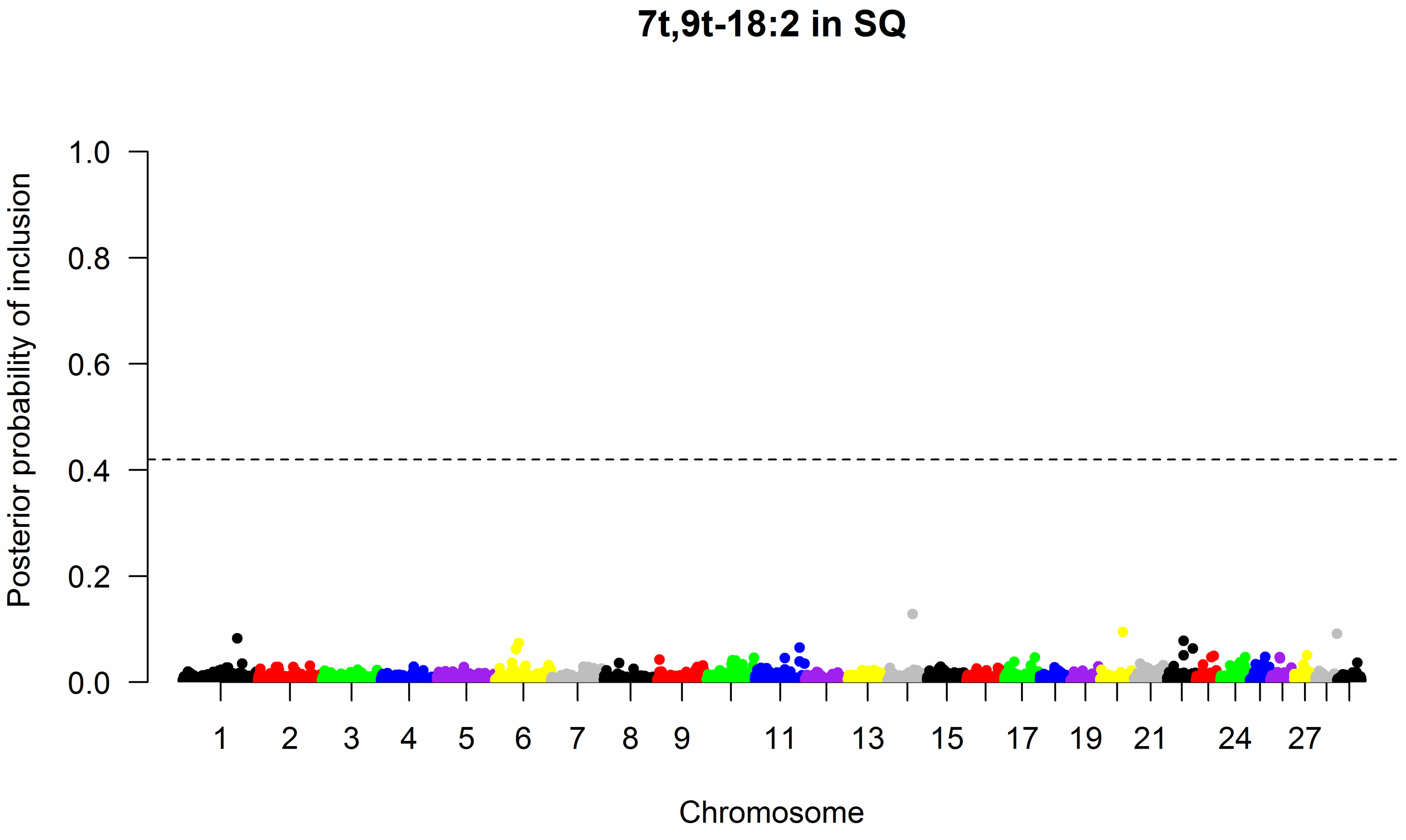

7t,9t-18:2 in SQ
Posterior probability of inclusion
1.0
0.8
0.6
0.4
0.2
0.0
1
2
3
4
5
6
7
8
9
11
13
15
17
19
21
24
27
Chromosome

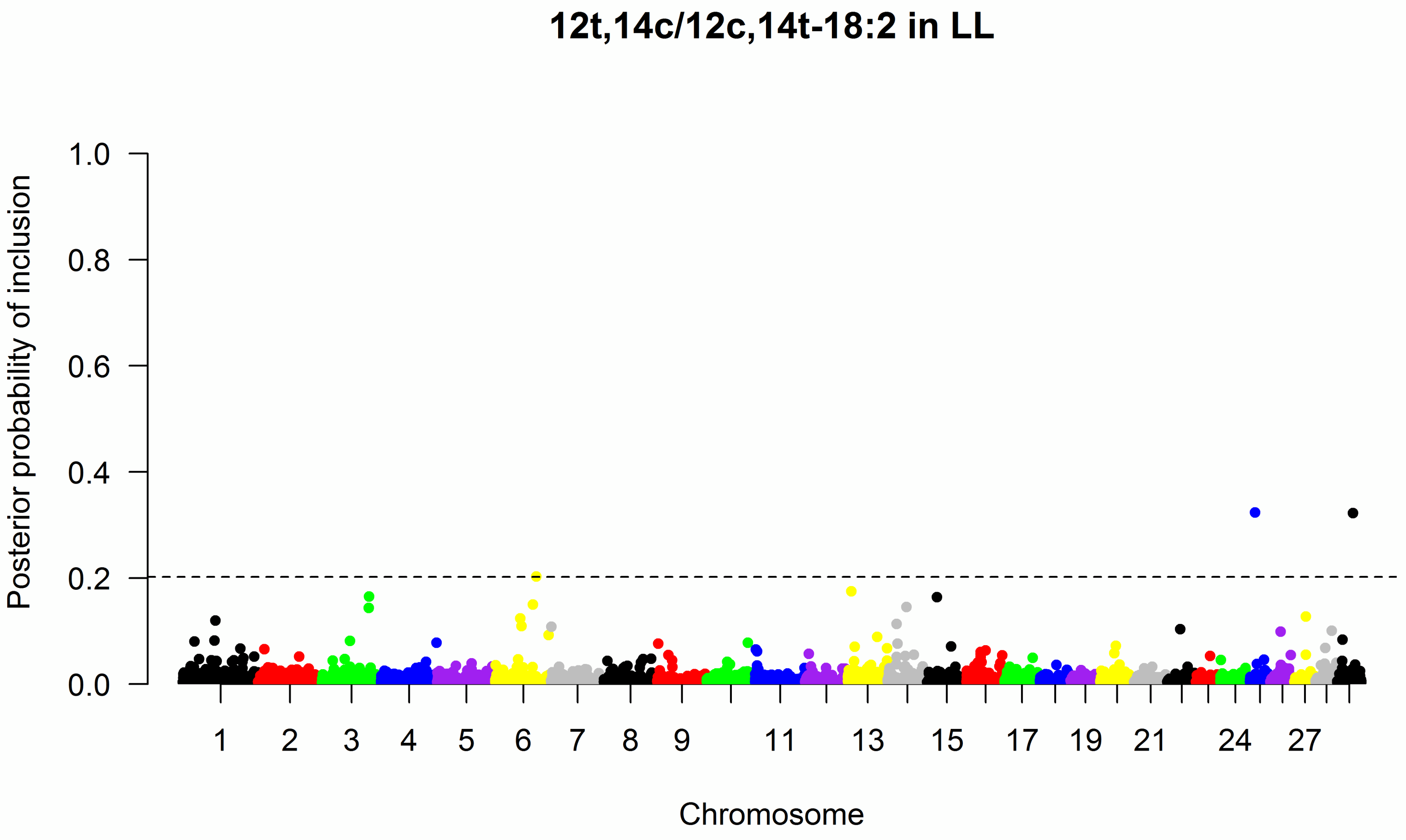
12t,14c/12c,14t-18:2 in LL
Posterior probability of inclusion
0.0
0.2
0.4
0.6
0.8
1.0
1
2
3
4
5
6
7
8
9
11
13
15
17
19
21
24
27
Chromosome

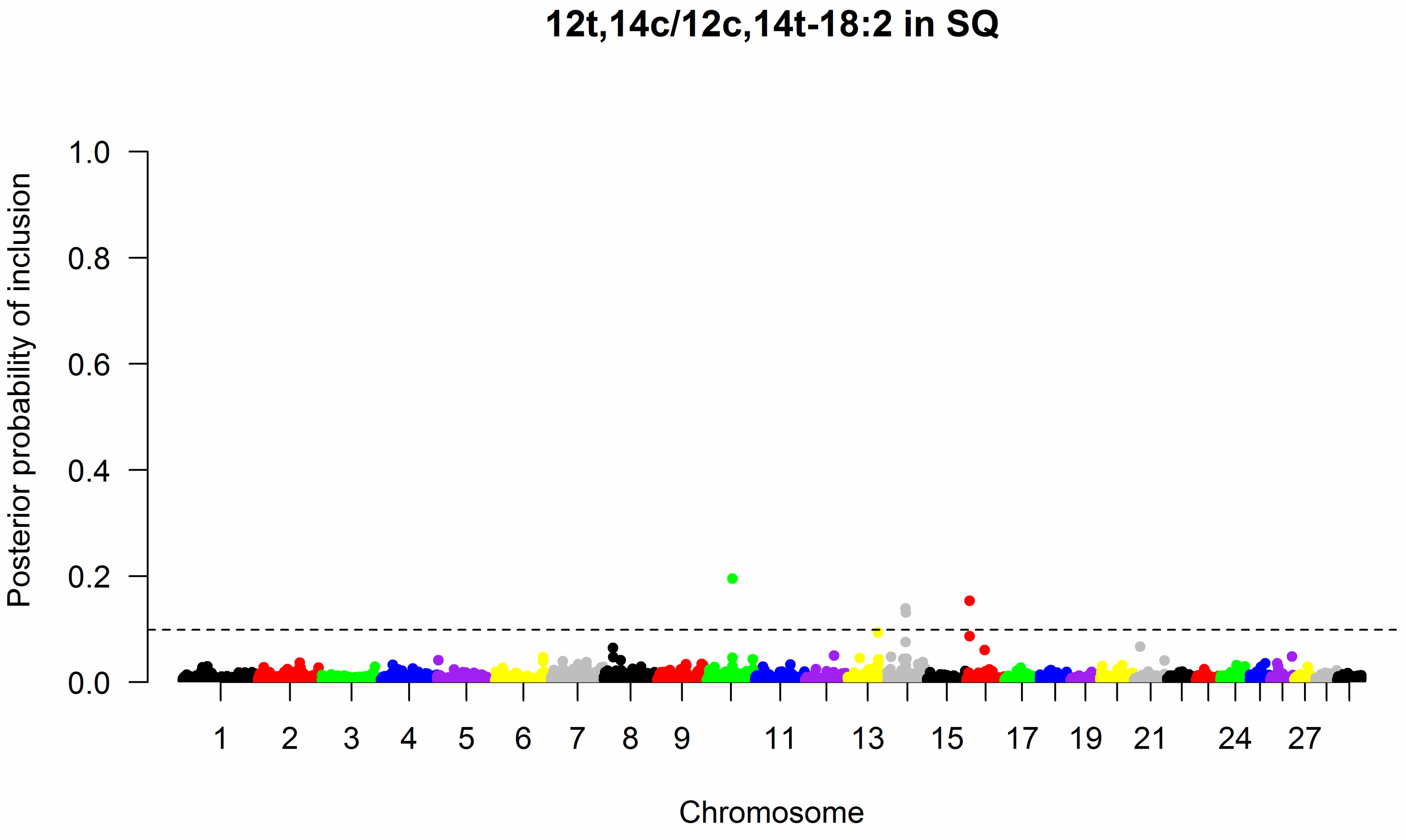
12t,14c/12c,14t-18:2 in SQ
Posterior probability of inclusion
0.0
0.2
0.4
0.6
0.8
1.0
1
2
3
4
5
6
7
8
9
11
13
15
17
19
21
24
27
Chromosome

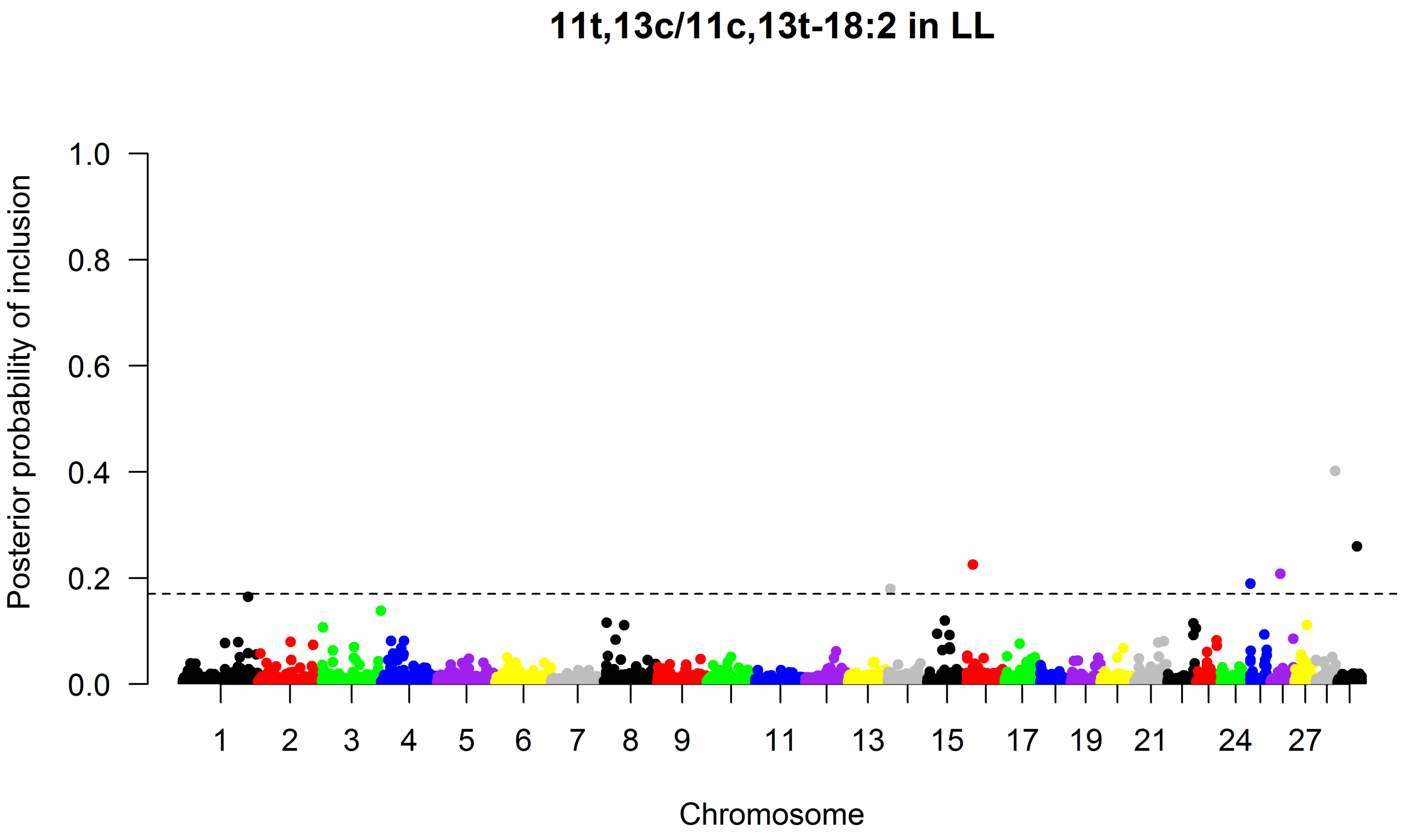
11t,13c/11c,13t-18:2 in LL
Posterior probability of inclusion
0.0
0.2
0.4
0.6
0.8
1.0
1
2
3
4
5
6
7
8
9
11
13
15
17
19
21
24
27
Chromosome

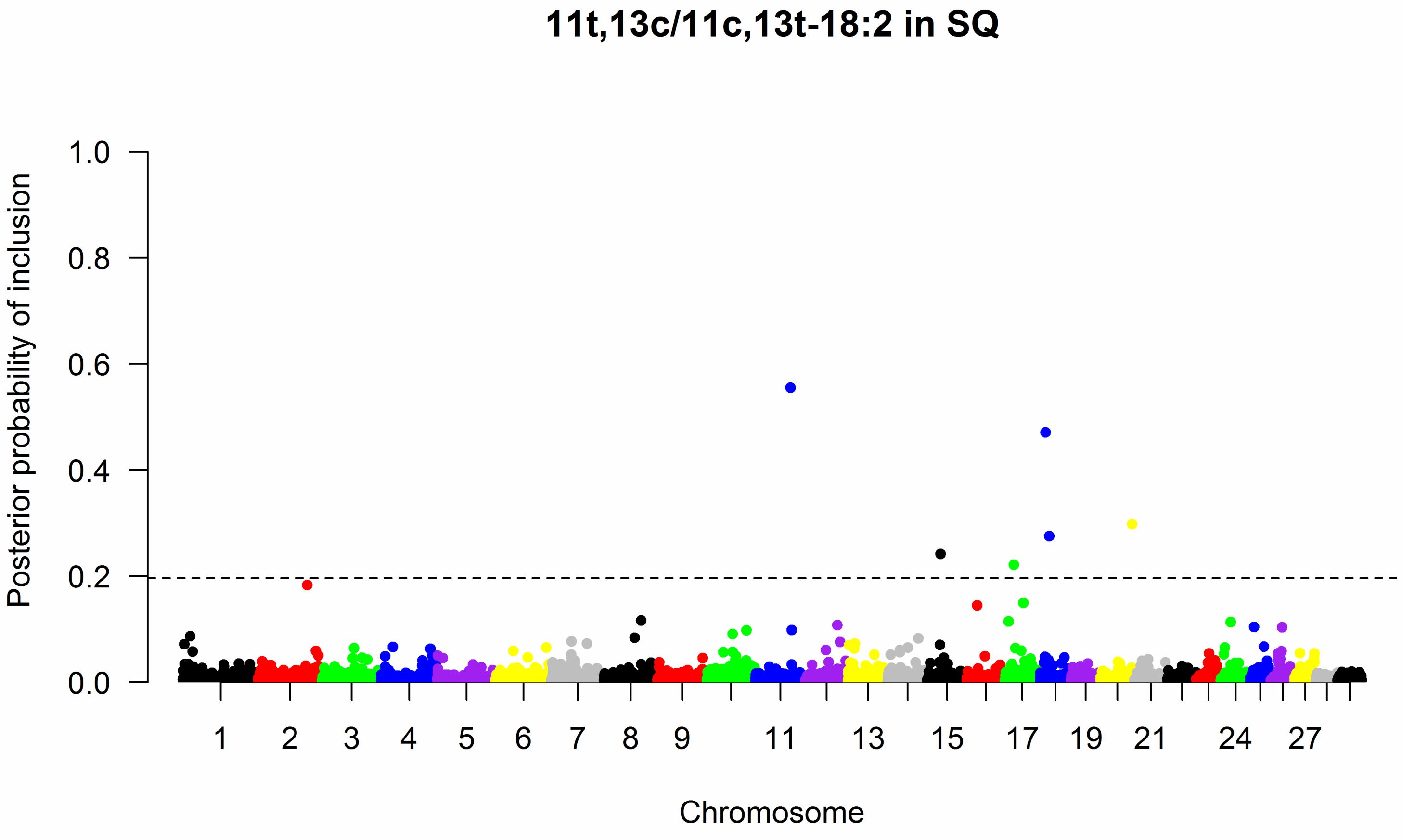
11t,13c/11c,13t-18:2 in SQ
Posterior probability of inclusion
0.0
0.2
0.4
0.6
0.8
1.0
1
2
3
4
5
6
7
8
9
11
13
15
17
19
21
24
27
Chromosome

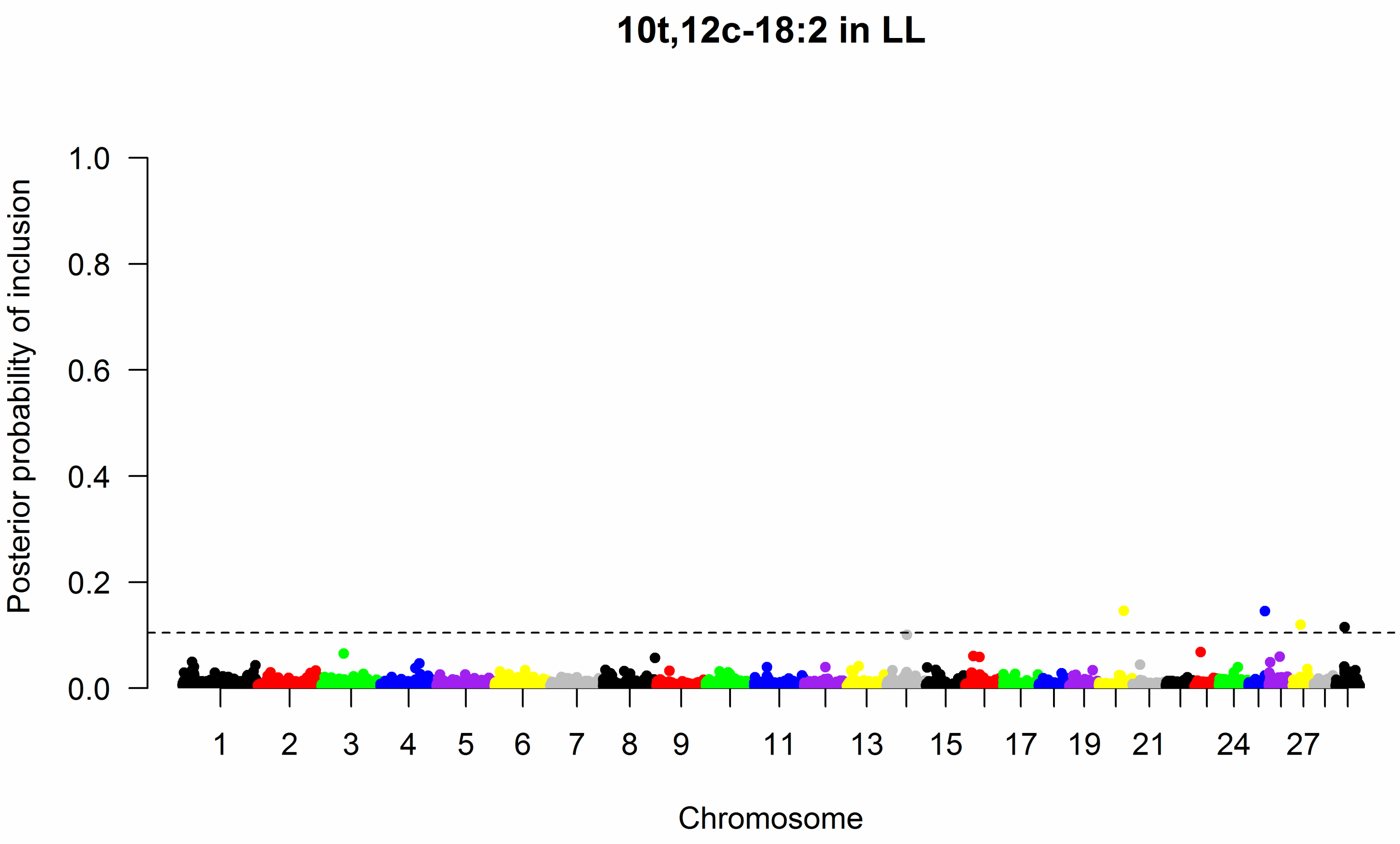
10t,12c-18:2 in LL
Posterior probability of inclusion
1.0
0.8
0.6
0.4
0.2
0.0
1
2
3
4
5
6
7
8
9
11
13
15
17
19
21
24
27
Chromosome

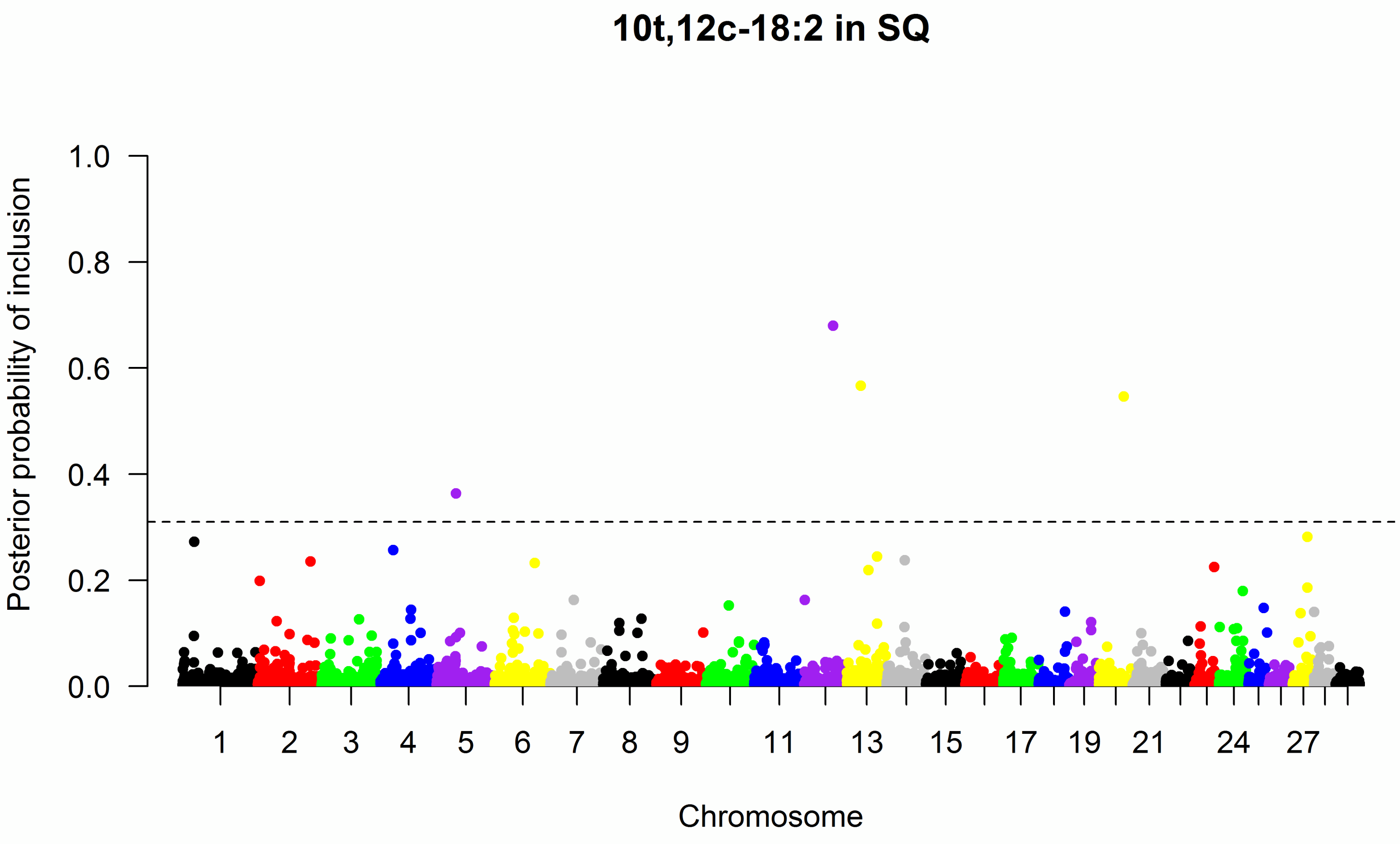
10t,12c-18:2 in SQ
Posterior probability of inclusion
1.0
0.8
0.6
0.4
0.2
0.0
1
2
3
4
5
6
7
8
9
11
13
15
17
19
21
24
27
Chromosome

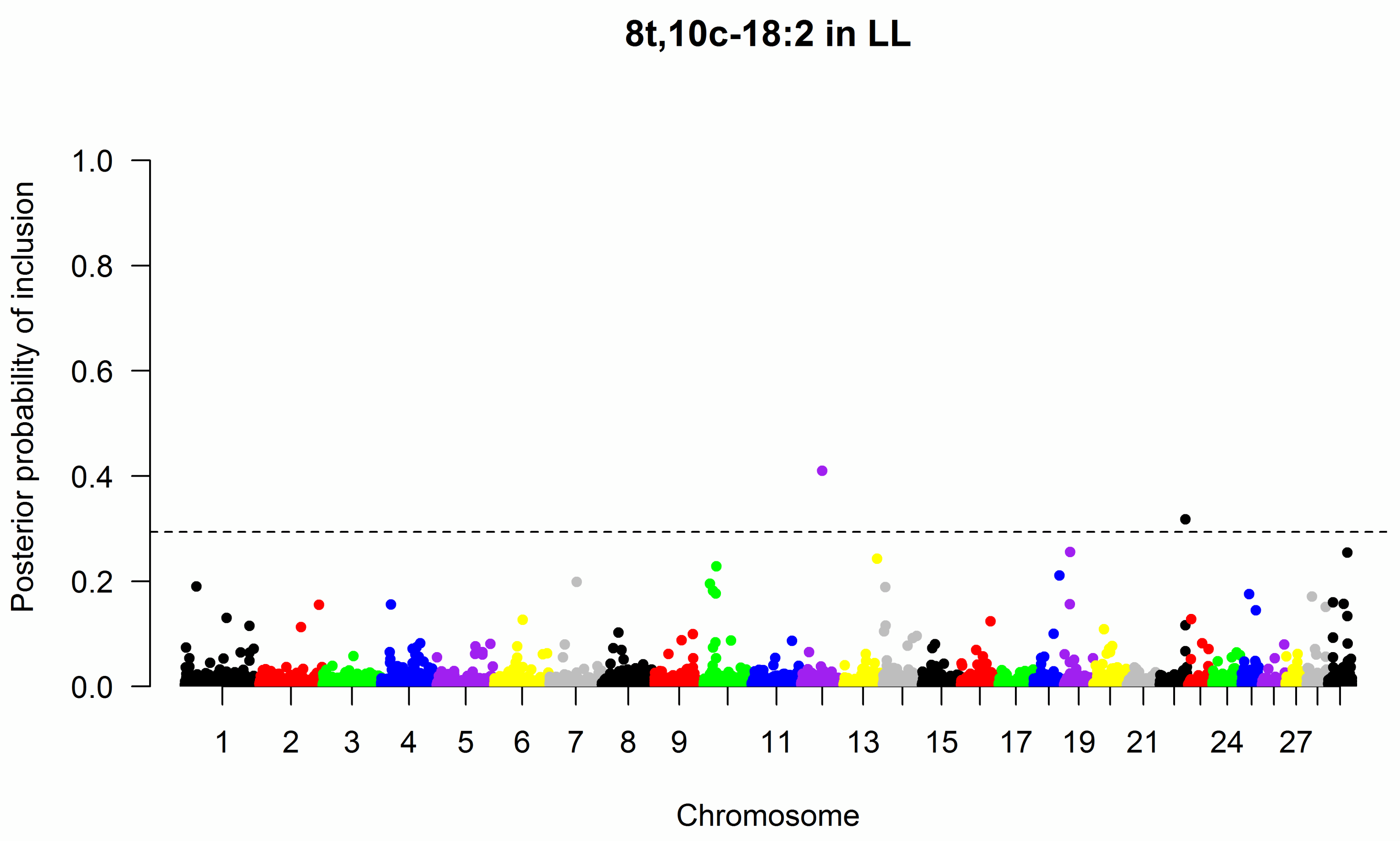
8t,10c-18:2 in LL
Posterior probability of inclusion
1.0
0.8
0.6
0.4
0.2
0.0
1
2
3
4
5
6
7
8
9
11
13
15
17
19
21
24
27
Chromosome

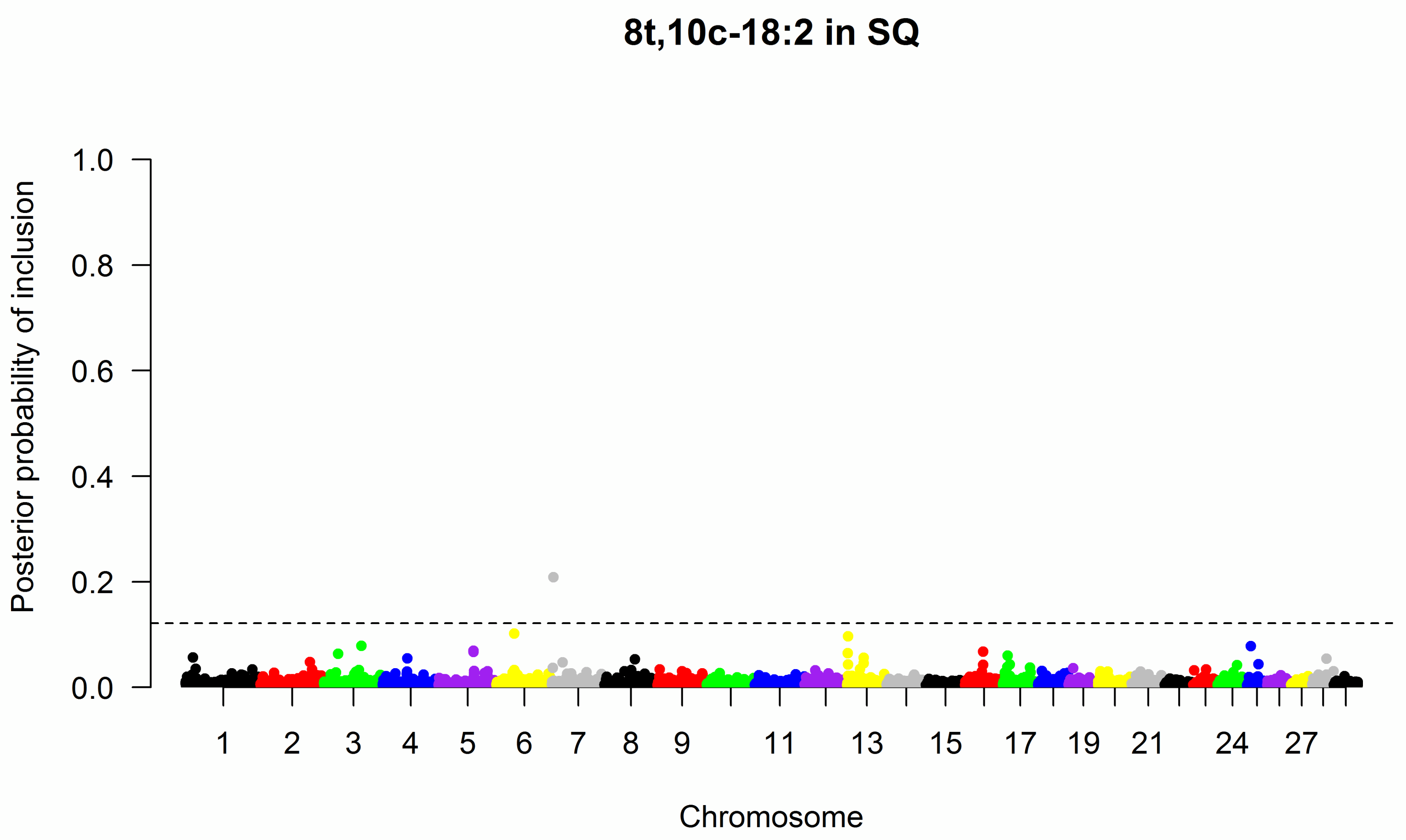
8t,10c-18:2 in SQ
Posterior probability of inclusion
1.0
0.8
0.6
0.4
0.2
0.0
1
2
3
4
5
6
7
8
9
11
13
15
17
19
21
24
27
Chromosome

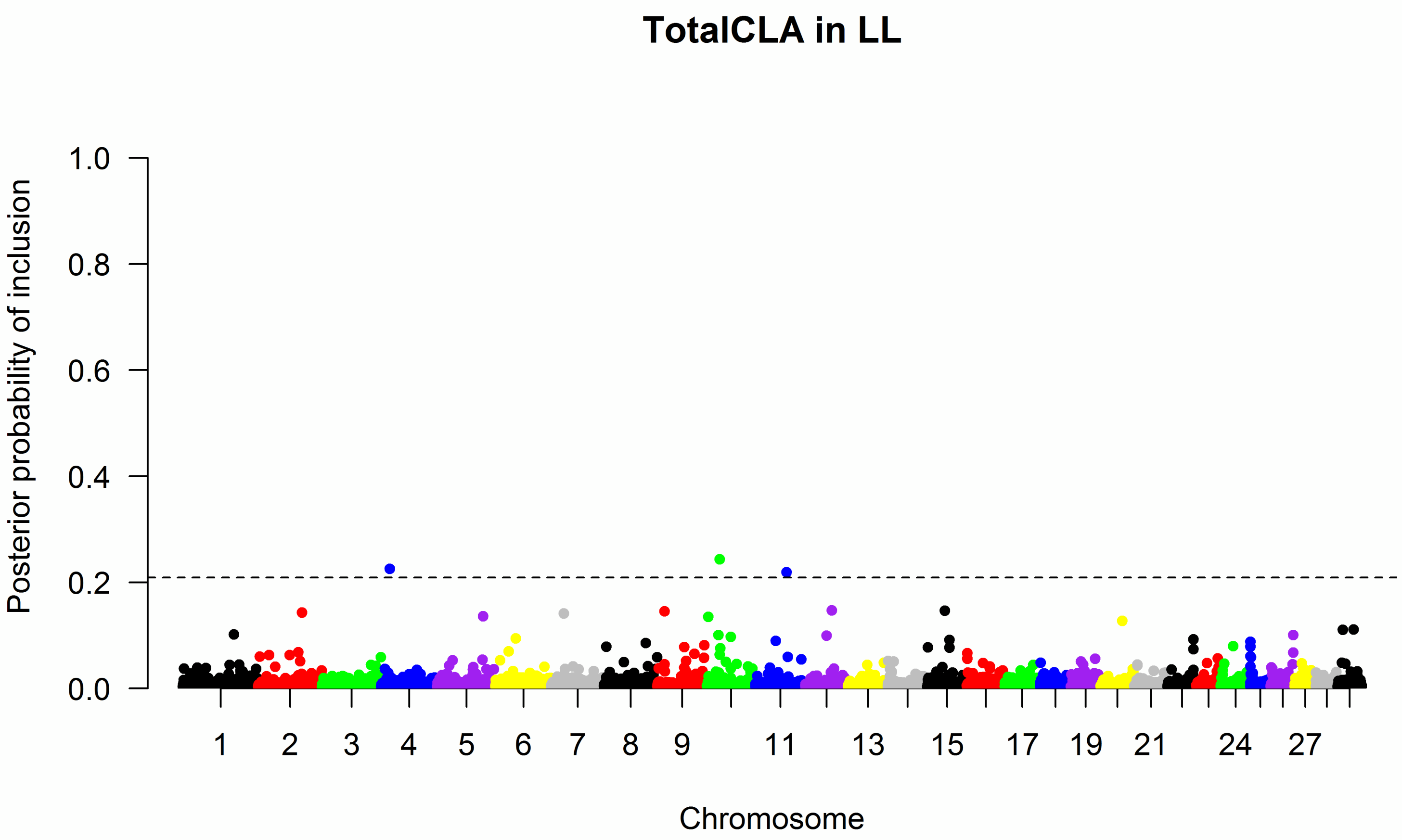

TotalCLA in LL
Posterior probability of inclusion
1.0
0.8
0.6
0.4
0.2
0.0
1
2
3
4
5
6
7
8
9
11
13
15
17
19
21
24
27
Chromosome

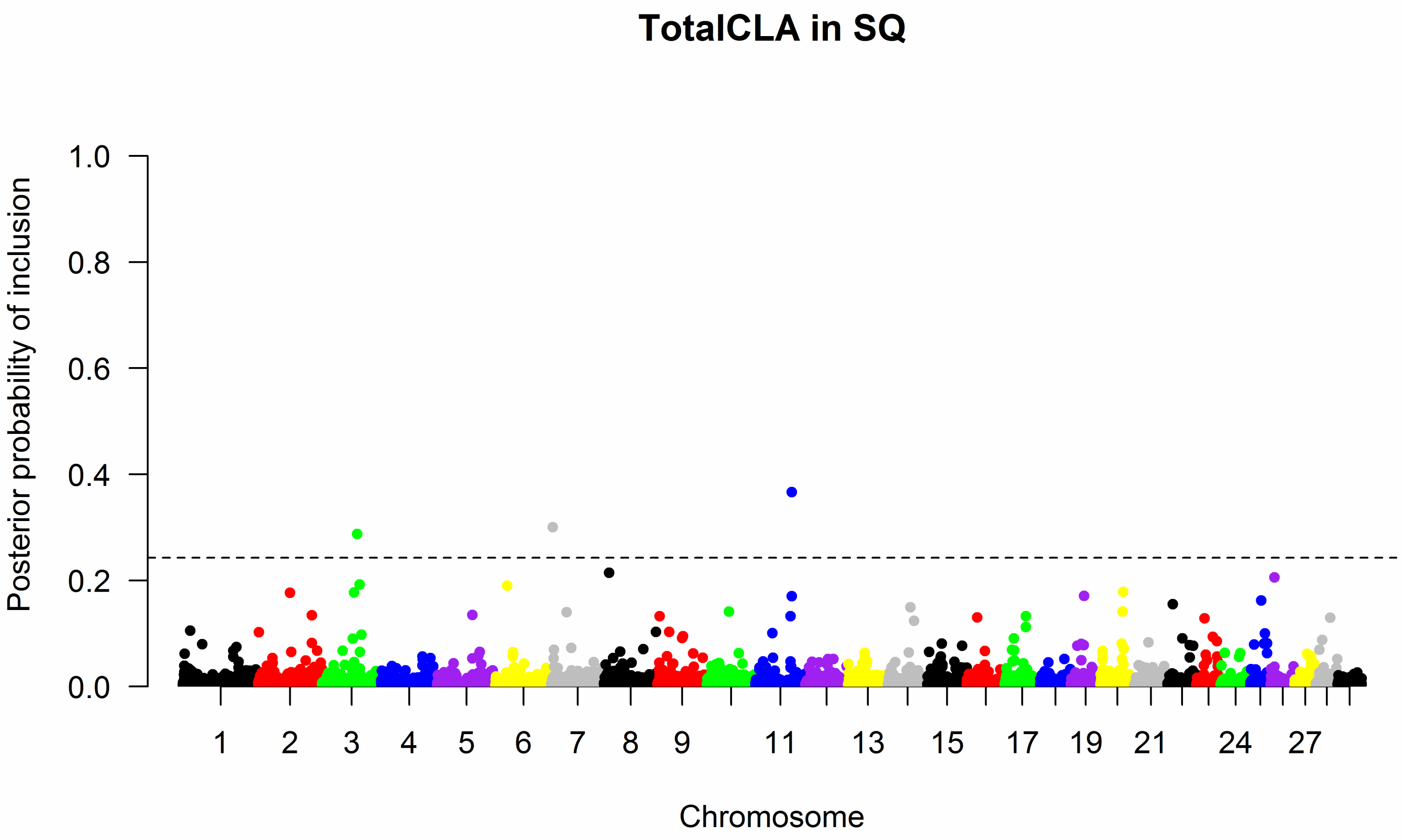

TotalCLA in SQ
Posterior probability of inclusion
1.0
0.8
0.6
0.4
0.2
0.0
1
2
3
4
5
6
7
8
9
11
13
15
17
19
21
24
27
Chromosome

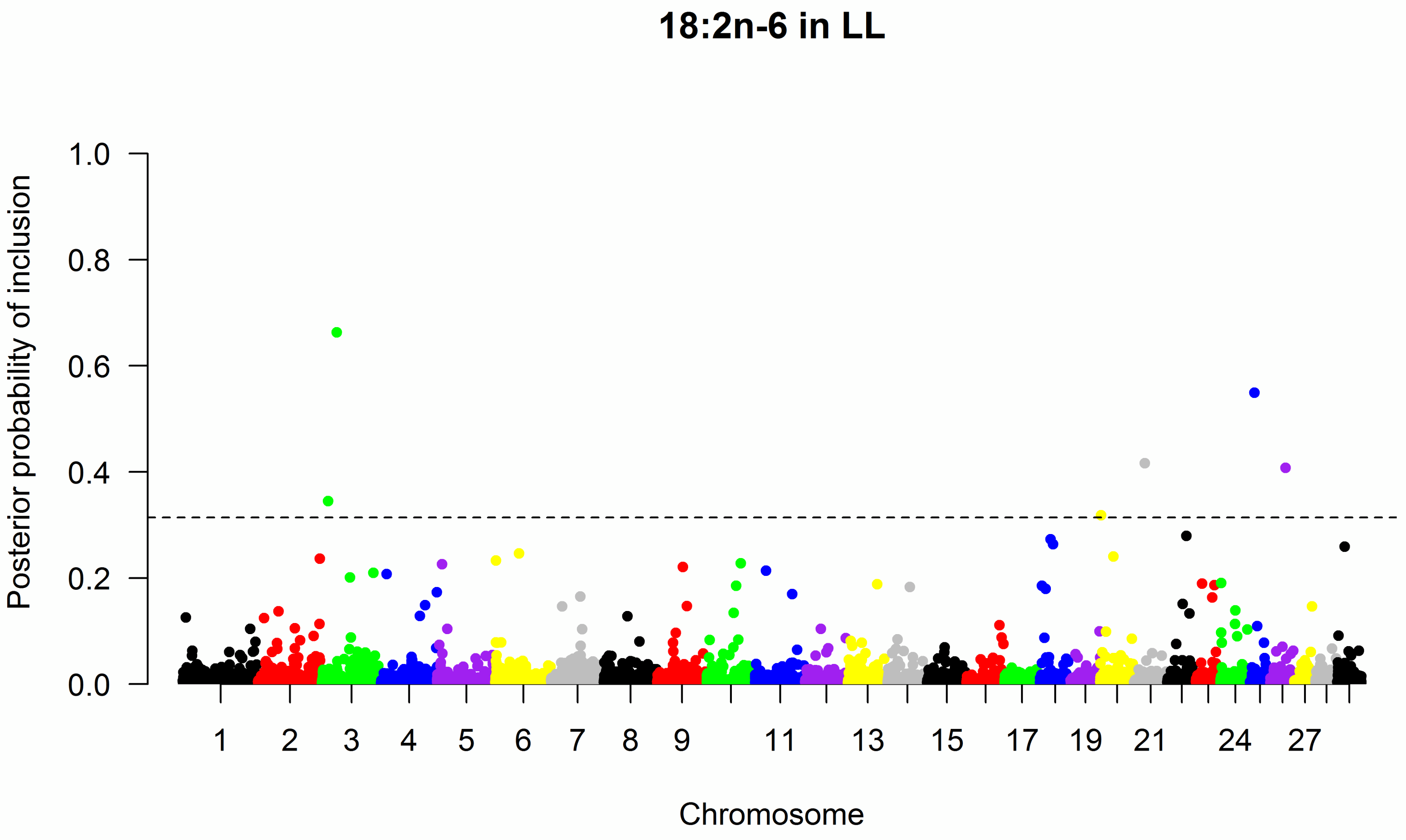
18:2n-6 in LL
1.0
0.8
0.6
0.4
0.2
0.0
Posterior probability of inclusion
1
2
3
4
5
6
7
8
9
11
13
15
17
19
21
24
27
Chromosome

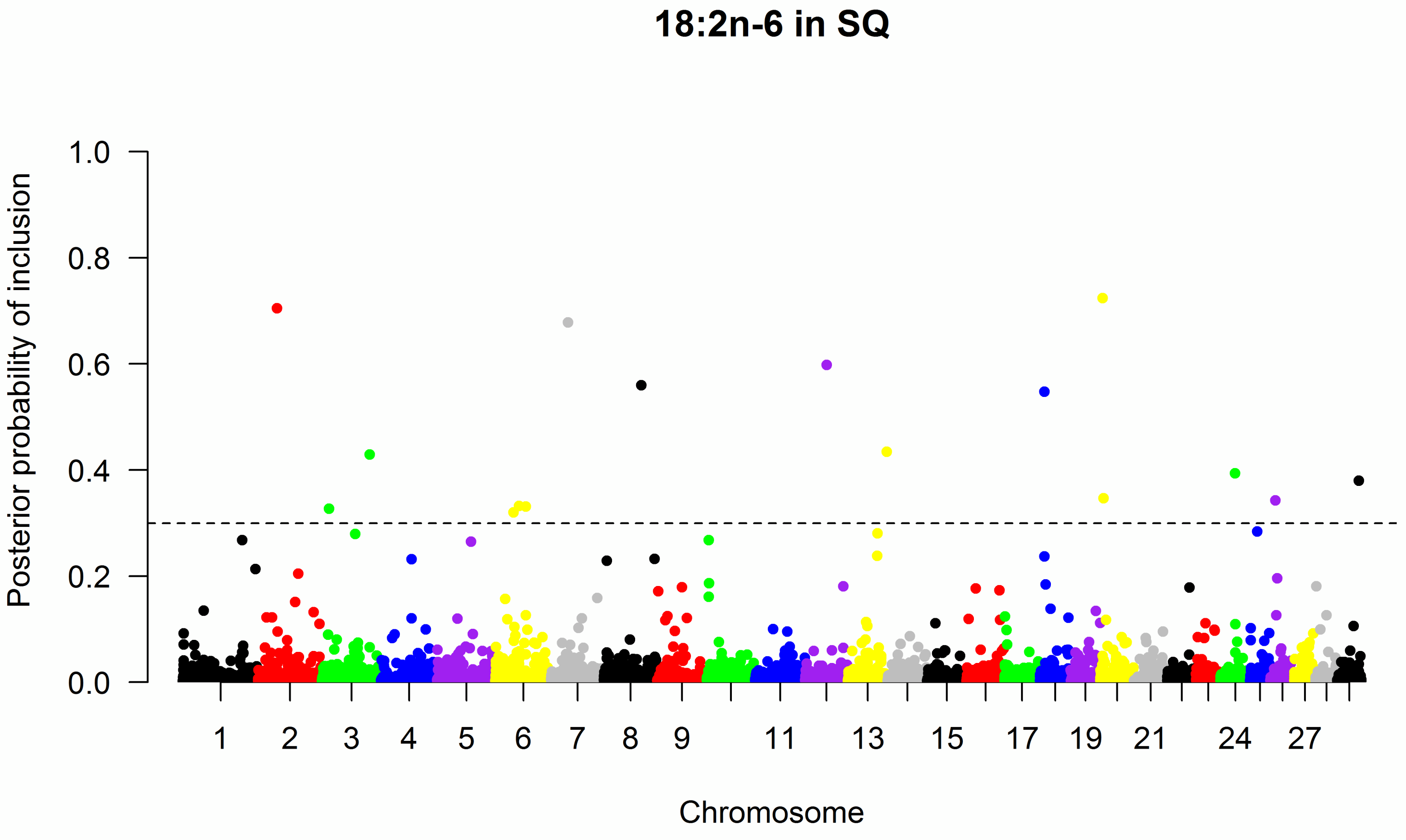
18:2n-6 in SQ
1.0
0.8
0.6
0.4
0.2
0.0
Posterior probability of inclusion
1
2
3
4
5
6
7
8
9
11
13
15
17
19
21
24
27
Chromosome

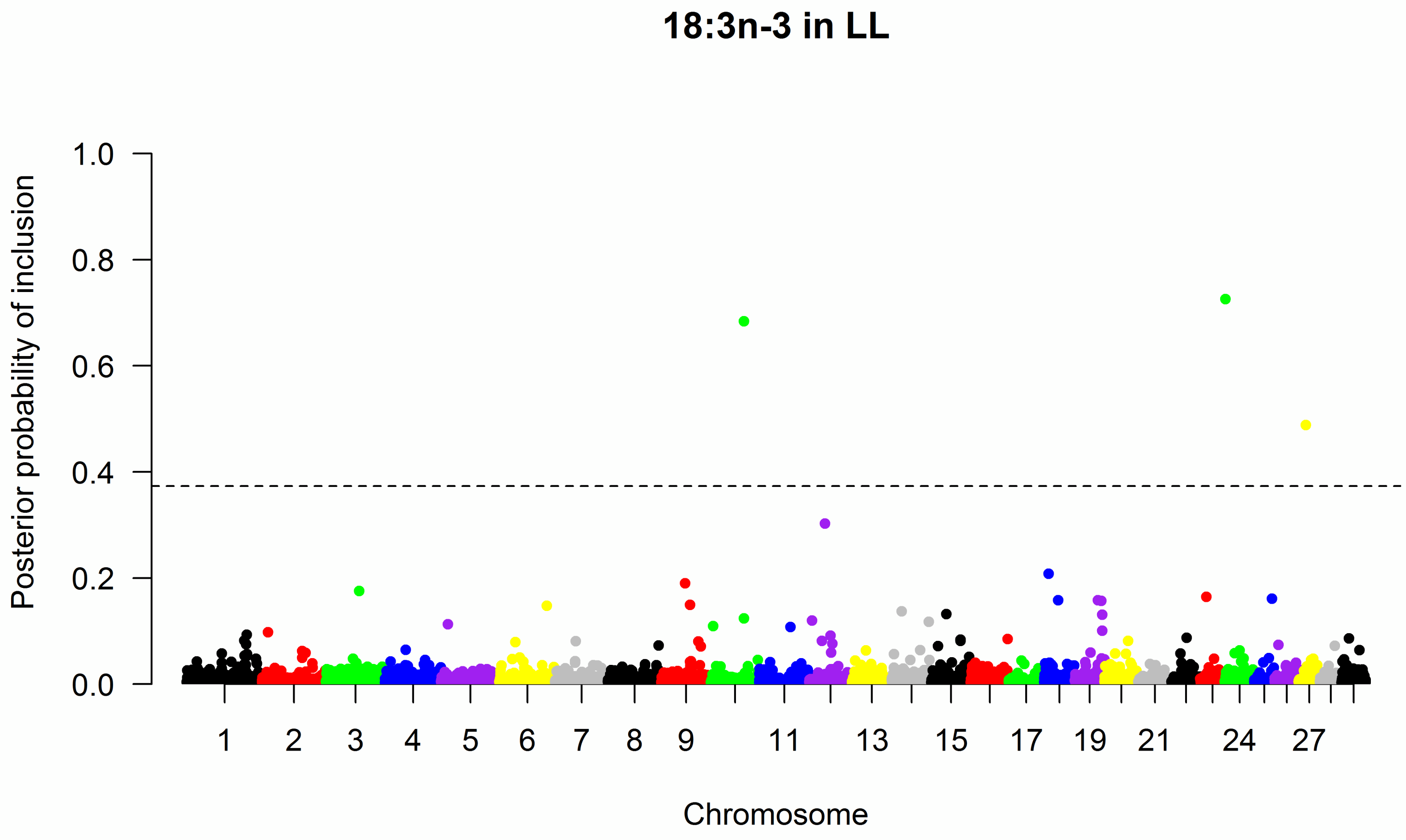
18:3n-3 in LL
Posterior probability of inclusion
0.0
0.2
0.4
0.6
0.8
1.0
1 2 3 4 5 6 7 8 9 11 13 15 17 19 21 24 27
Chromosome

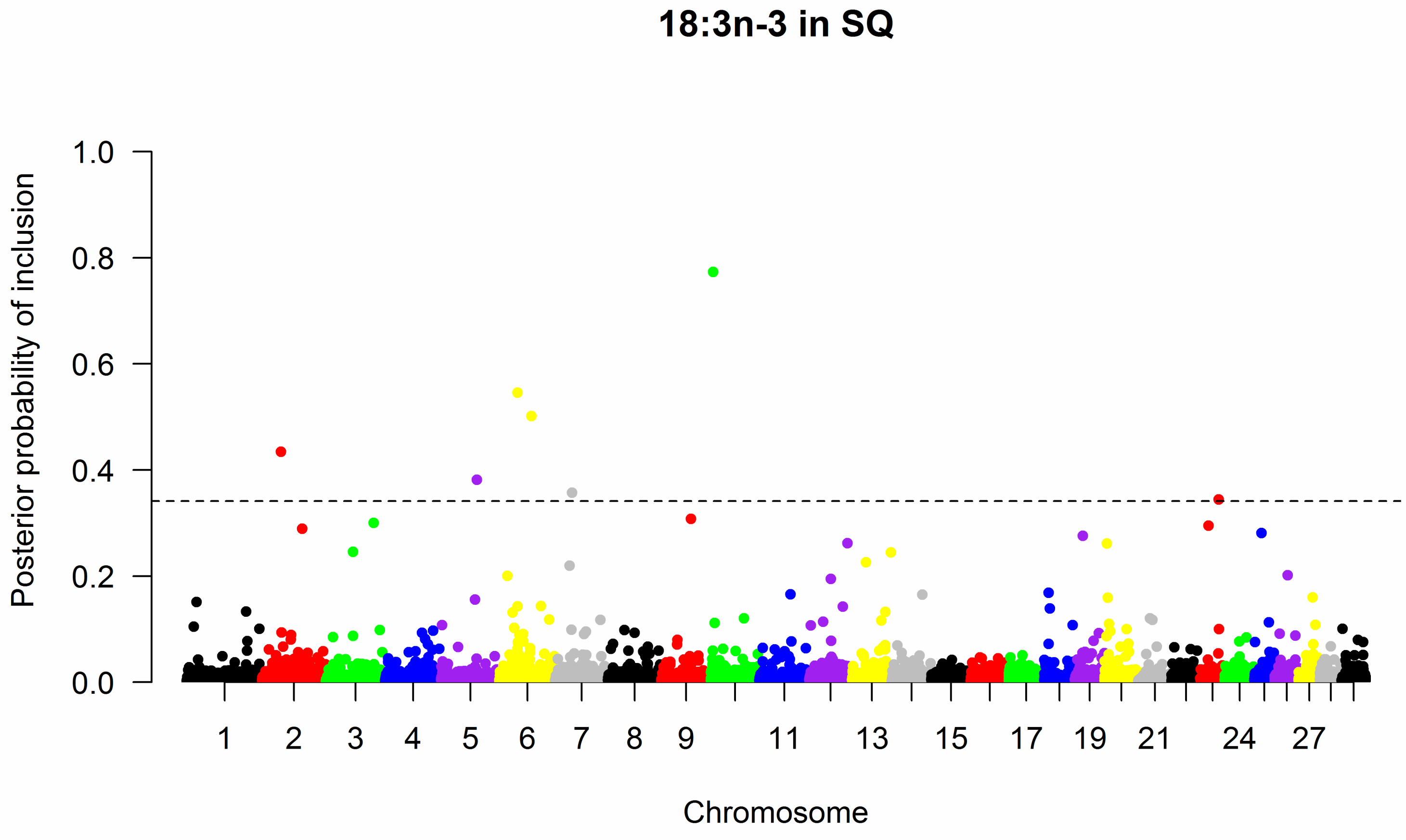
18:3n-3 in SQ
Posterior probability of inclusion
0.0
0.2
0.4
0.6
0.8
1.0
1 2 3 4 5 6 7 8 9 11 13 15 17 19 21 24 27
Chromosome

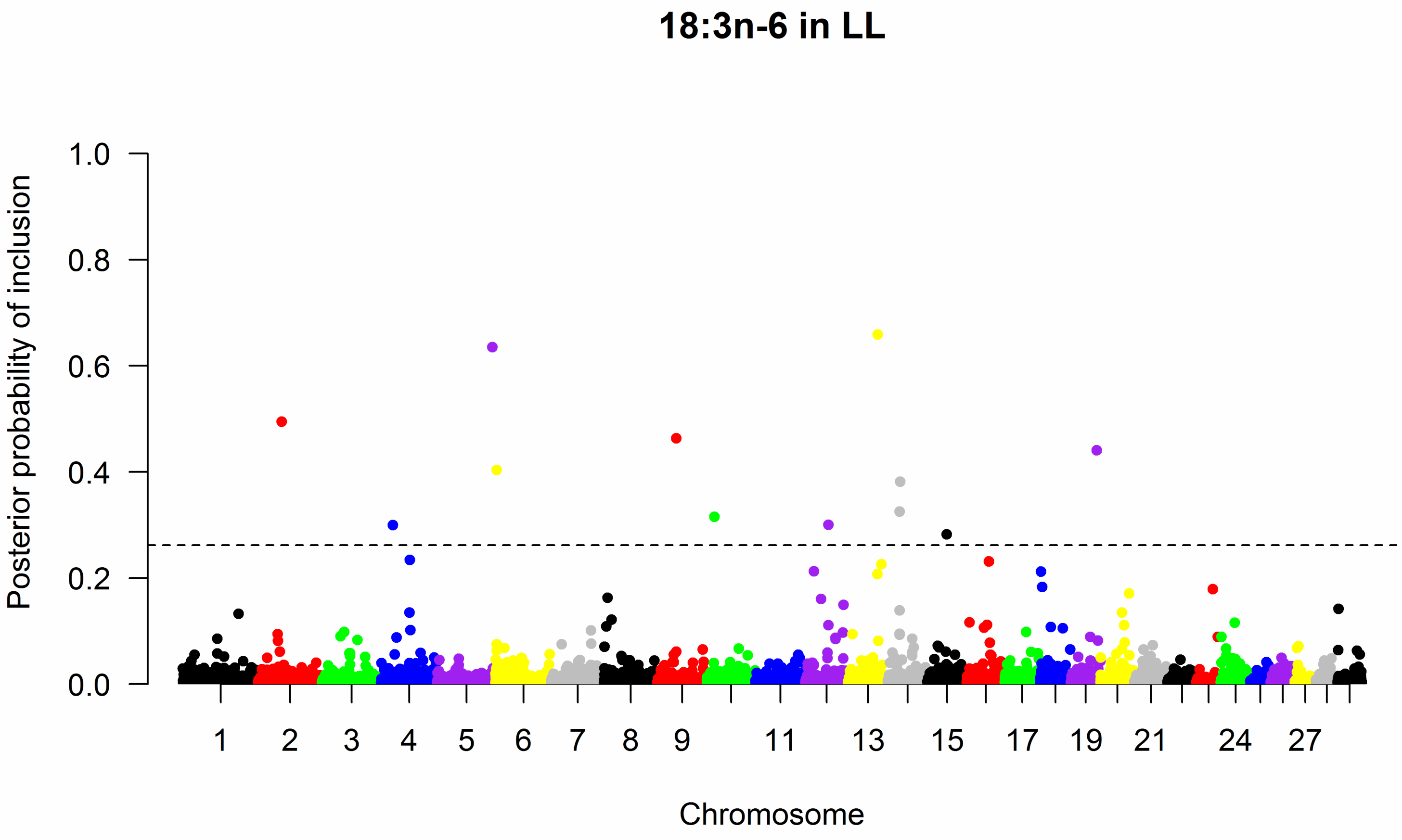
18:3n-6 in LL
Posterior probability of inclusion
1.0
0.8
0.6
0.4
0.2
0.0
1
2
3
4
5
6
7
8
9
11
13
15
17
19
21
24
27
Chromosome

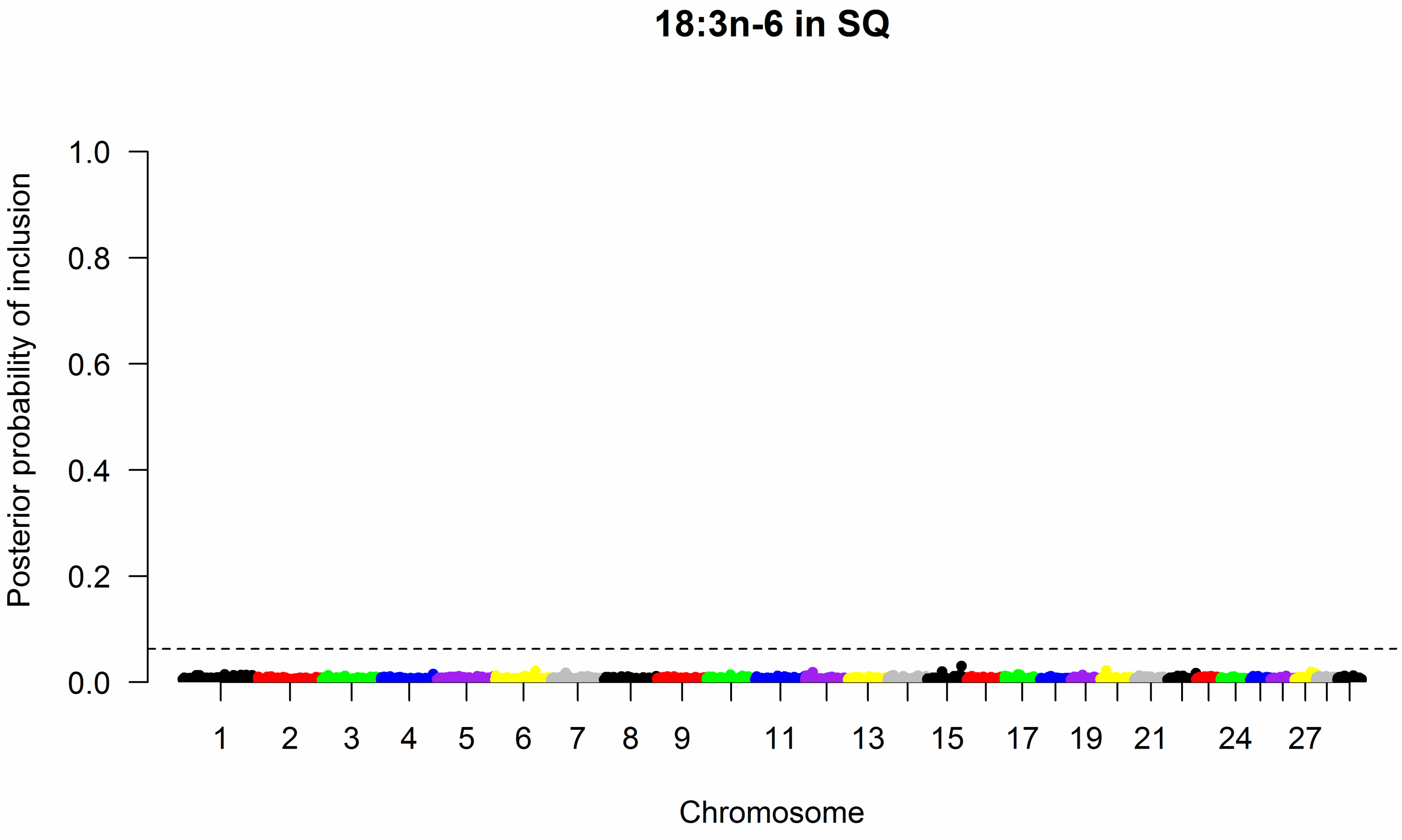
18:3n-6 in SQ
Posterior probability of inclusion
1.0
0.8
0.6
0.4
0.2
0.0
1
2
3
4
5
6
7
8
9
11
13
15
17
19
21
24
27
Chromosome

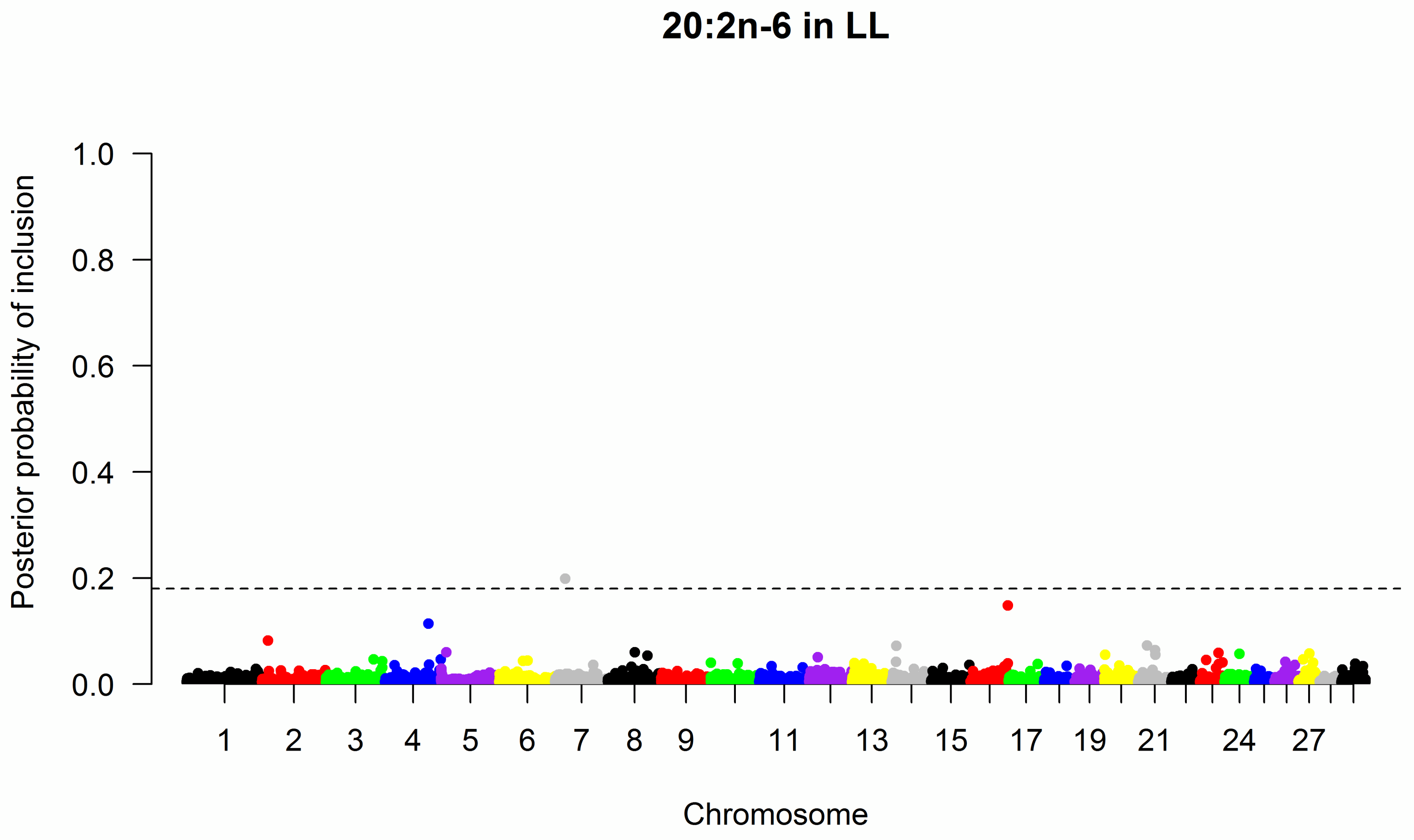
20:2n-6 in LL
Posterior probability of inclusion
0.0
0.2
0.4
0.6
0.8
1.0
Chromosome
1
2
3
4
5
6
7
8
9
11
13
15
17
19
21
24
27

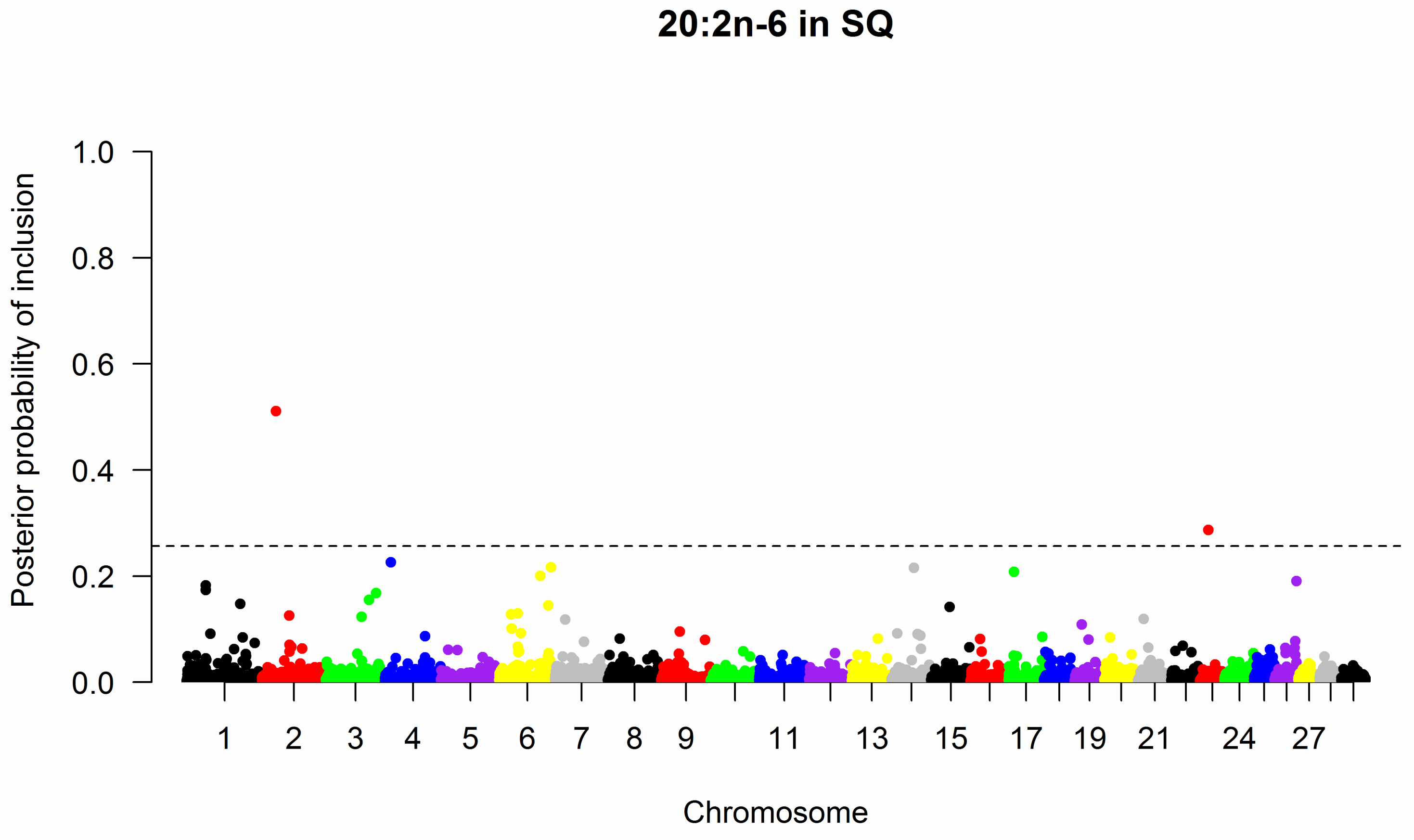
20:2n-6 in SQ
Posterior probability of inclusion
0.0
0.2
0.4
0.6
0.8
1.0
Chromosome
1
2
3
4
5
6
7
8
9
11
13
15
17
19
21
24
27

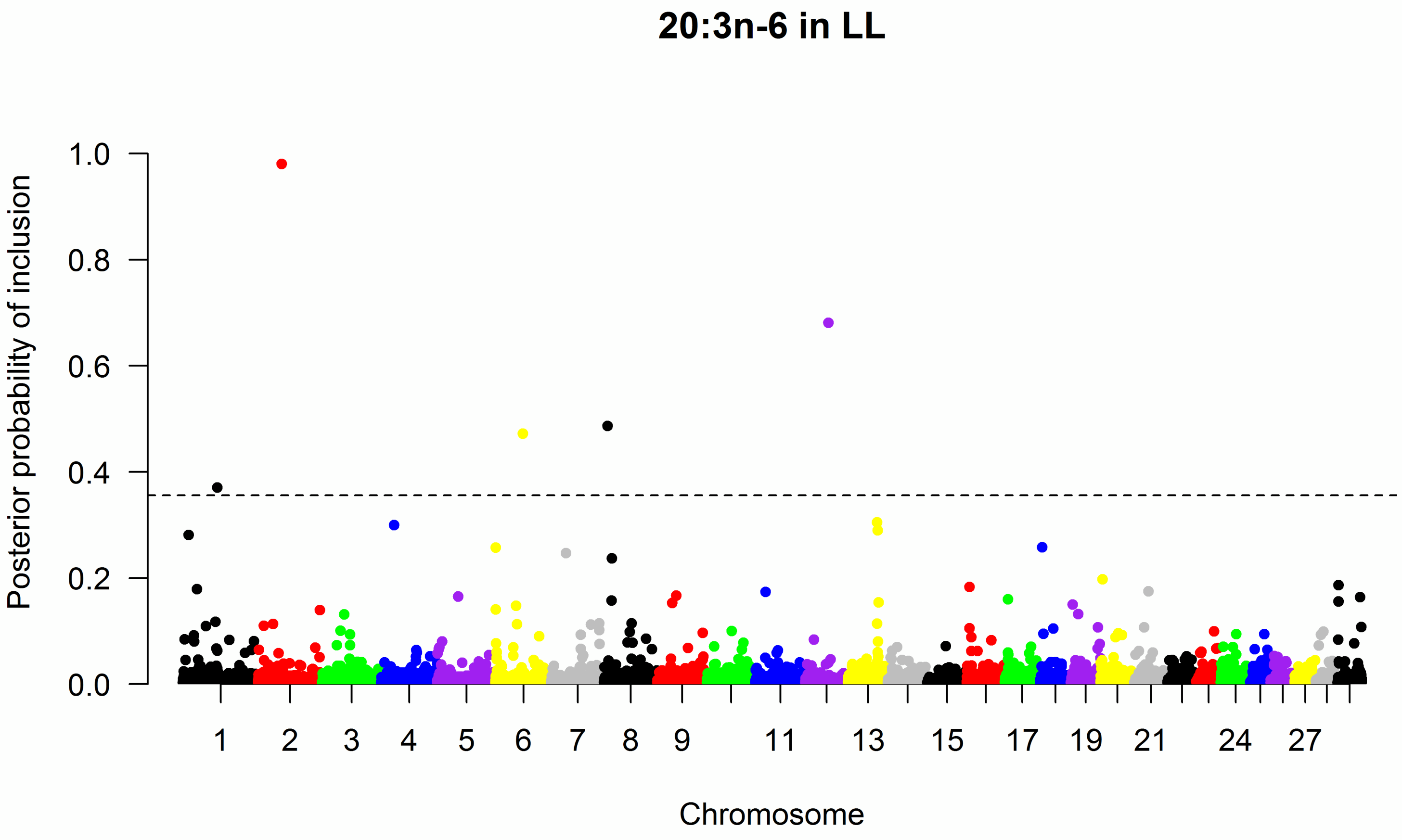
20:3n-6 in LL
Posterior probability of inclusion
1.0
0.8
0.6
0.4
0.2
0.0
1
2
3
4
5
6
7
8
9
11
13
15
17
19
21
24
27
Chromosome

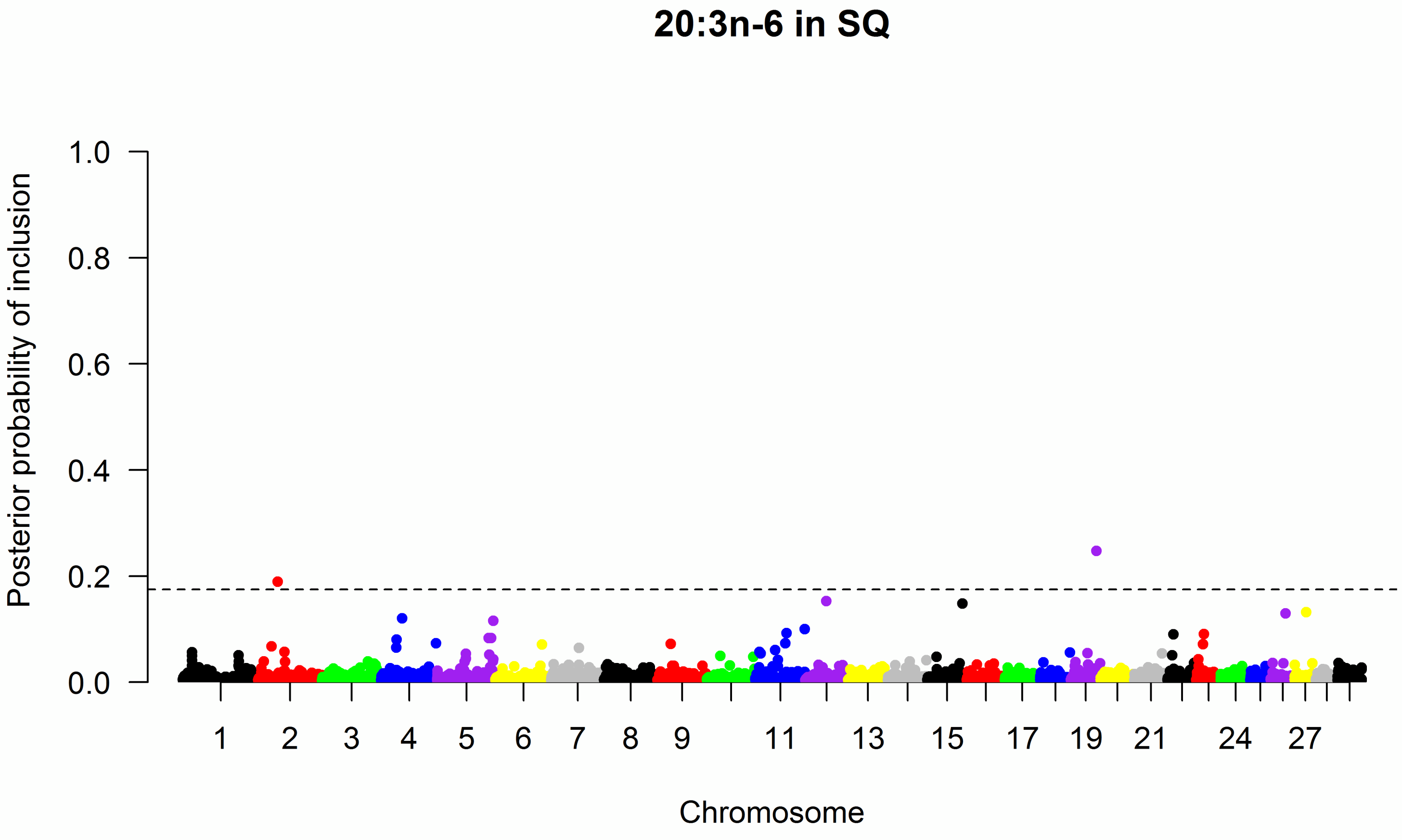
20:3n-6 in SQ
Posterior probability of inclusion
1.0
0.8
0.6
0.4
0.2
0.0
1
2
3
4
5
6
7
8
9
11
13
15
17
19
21
24
27
Chromosome

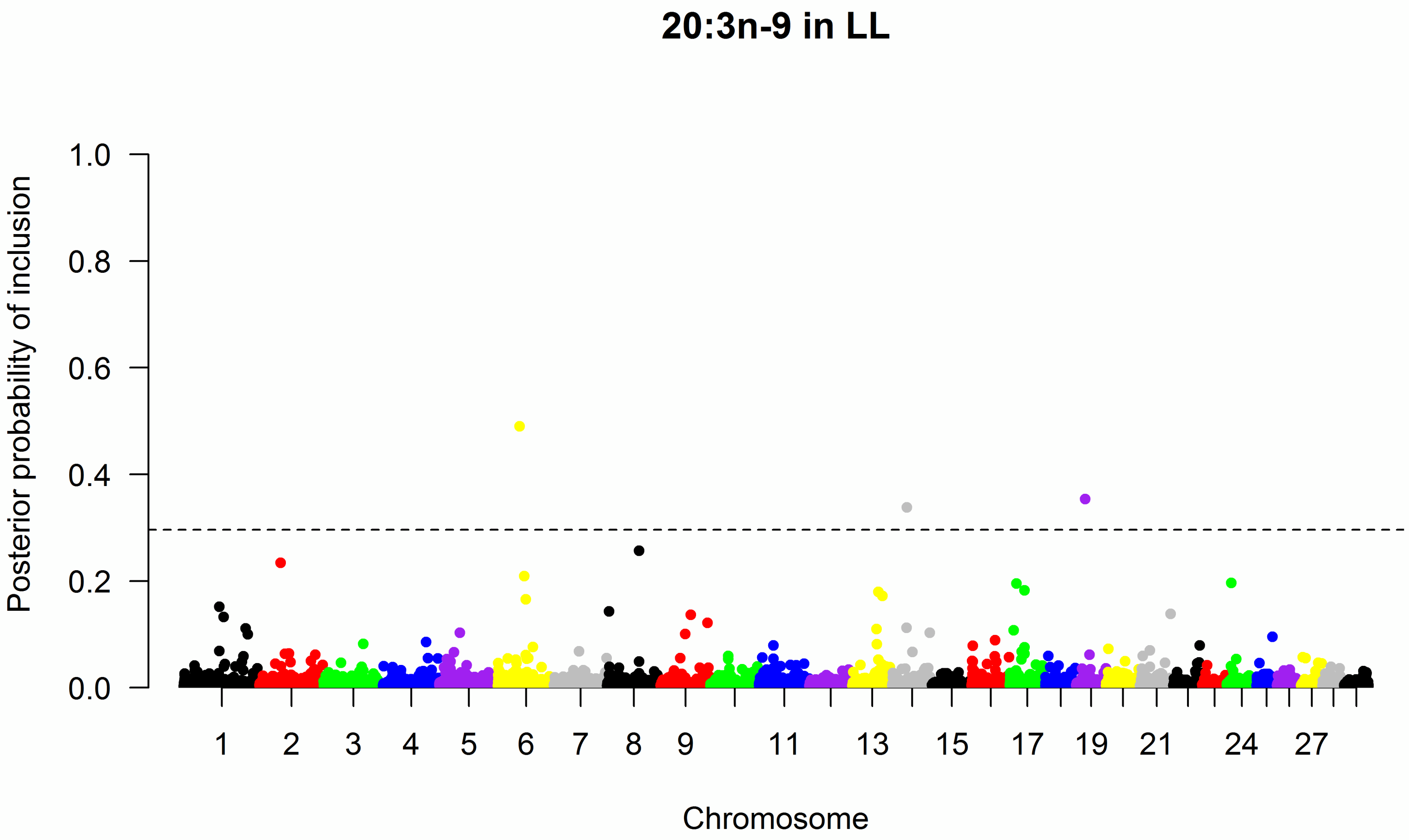
20:3n-9 in LL
Posterior probability of inclusion
1.0
0.8
0.6
0.4
0.2
0.0
1
2
3
4
5
6
7
8
9
11
13
15
17
19
21
24
27
Chromosome

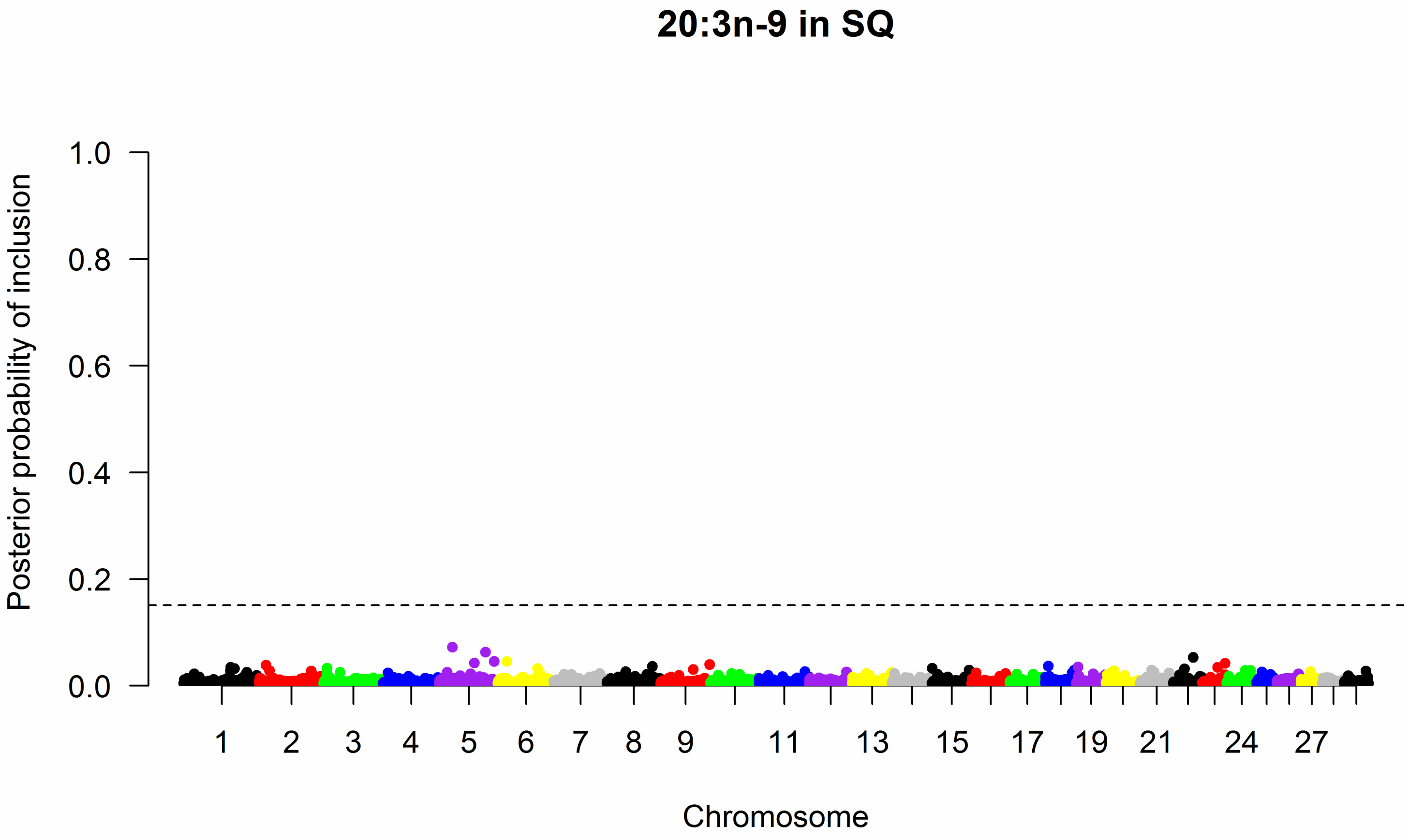
20:3n-9 in SQ
Posterior probability of inclusion
1.0
0.8
0.6
0.4
0.2
0.0
1
2
3
4
5
6
7
8
9
11
13
15
17
19
21
24
27
Chromosome

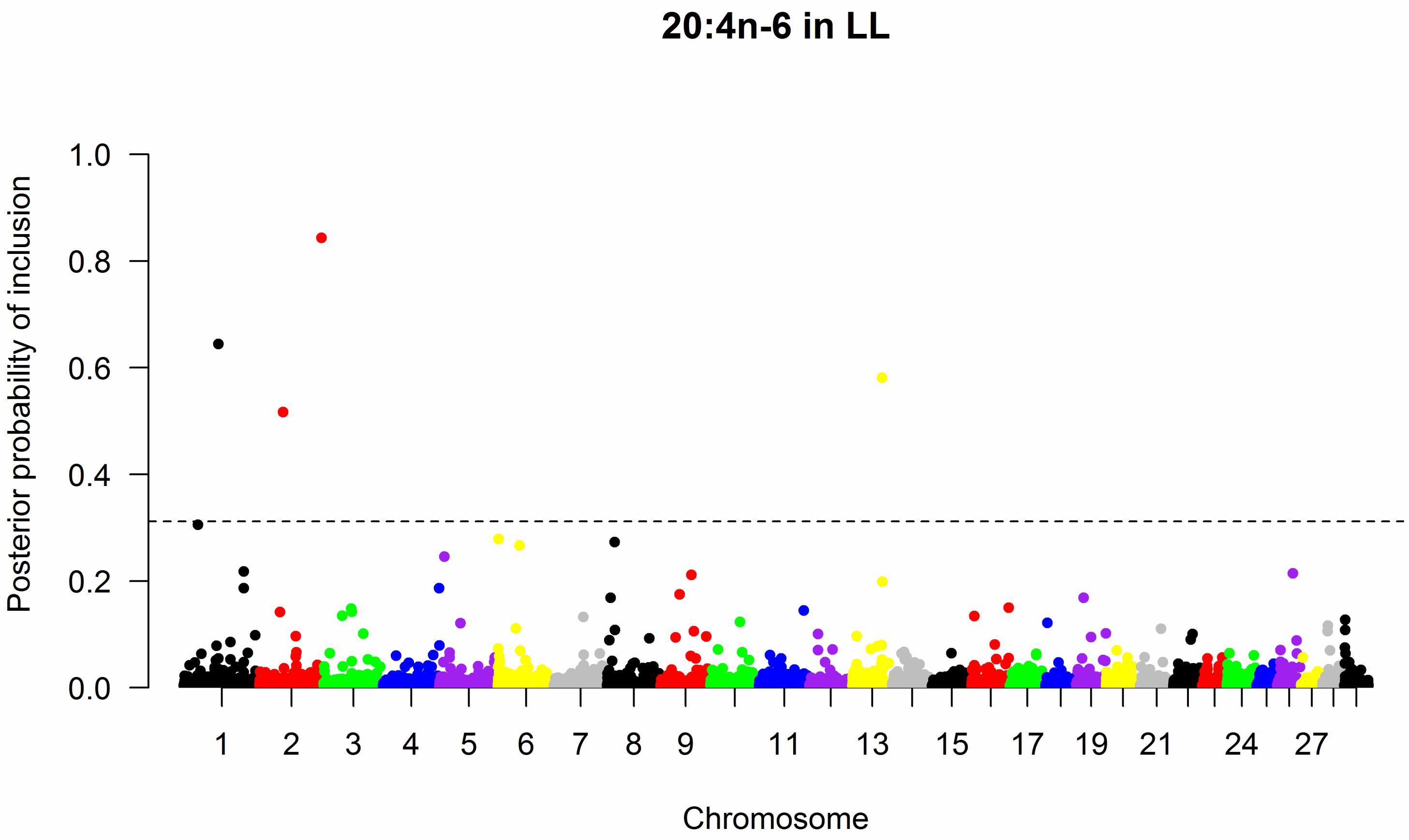
20:4n-6 in LL
Posterior probability of inclusion
1.0
0.8
0.6
0.4
0.2
0.0
1
2
3
4
5
6
7
8
9
11
13
15
17
19
21
24
27
Chromosome

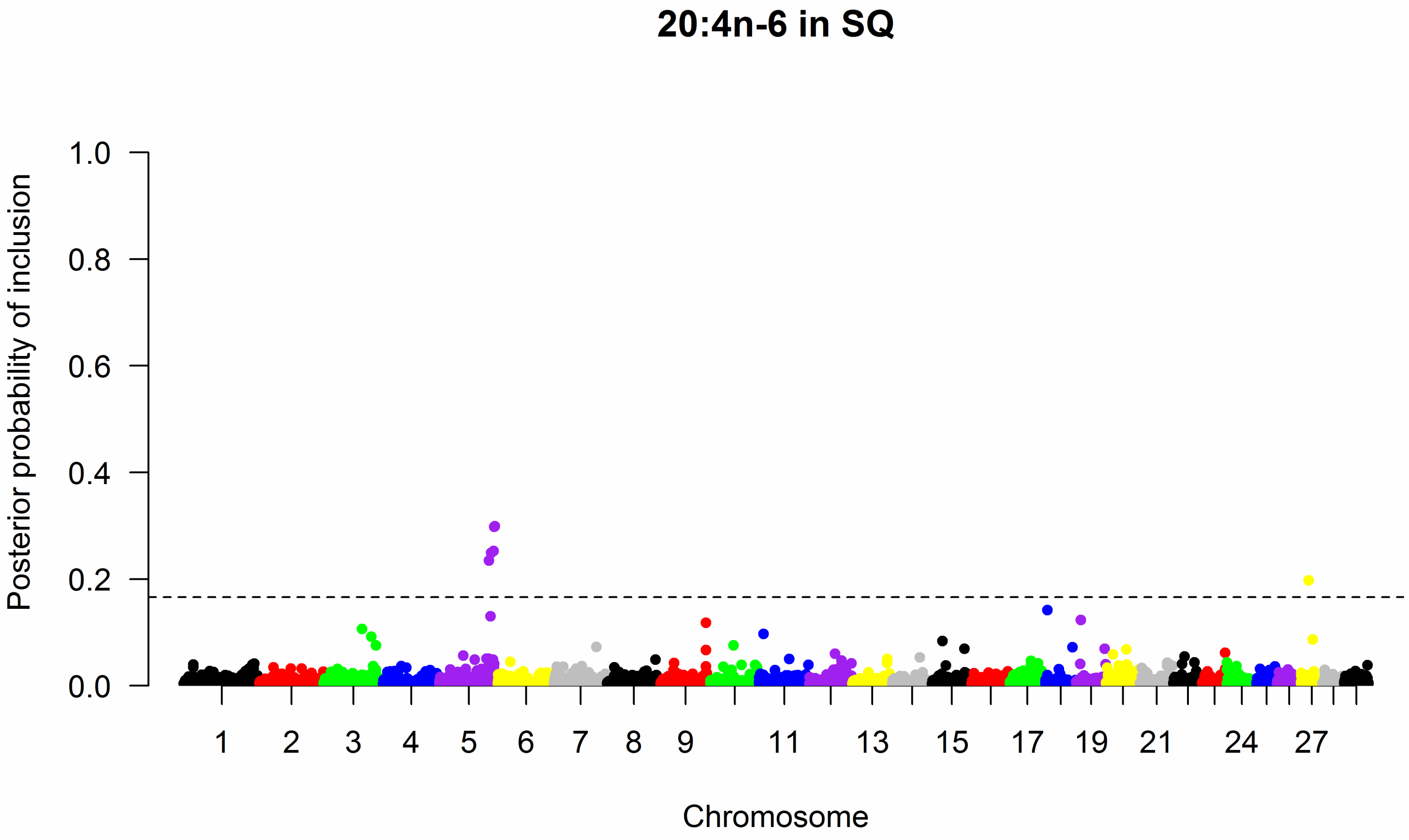
20:4n-6 in SQ
Posterior probability of inclusion
1.0
0.8
0.6
0.4
0.2
0.0
1
2
3
4
5
6
7
8
9
11
13
15
17
19
21
24
27
Chromosome

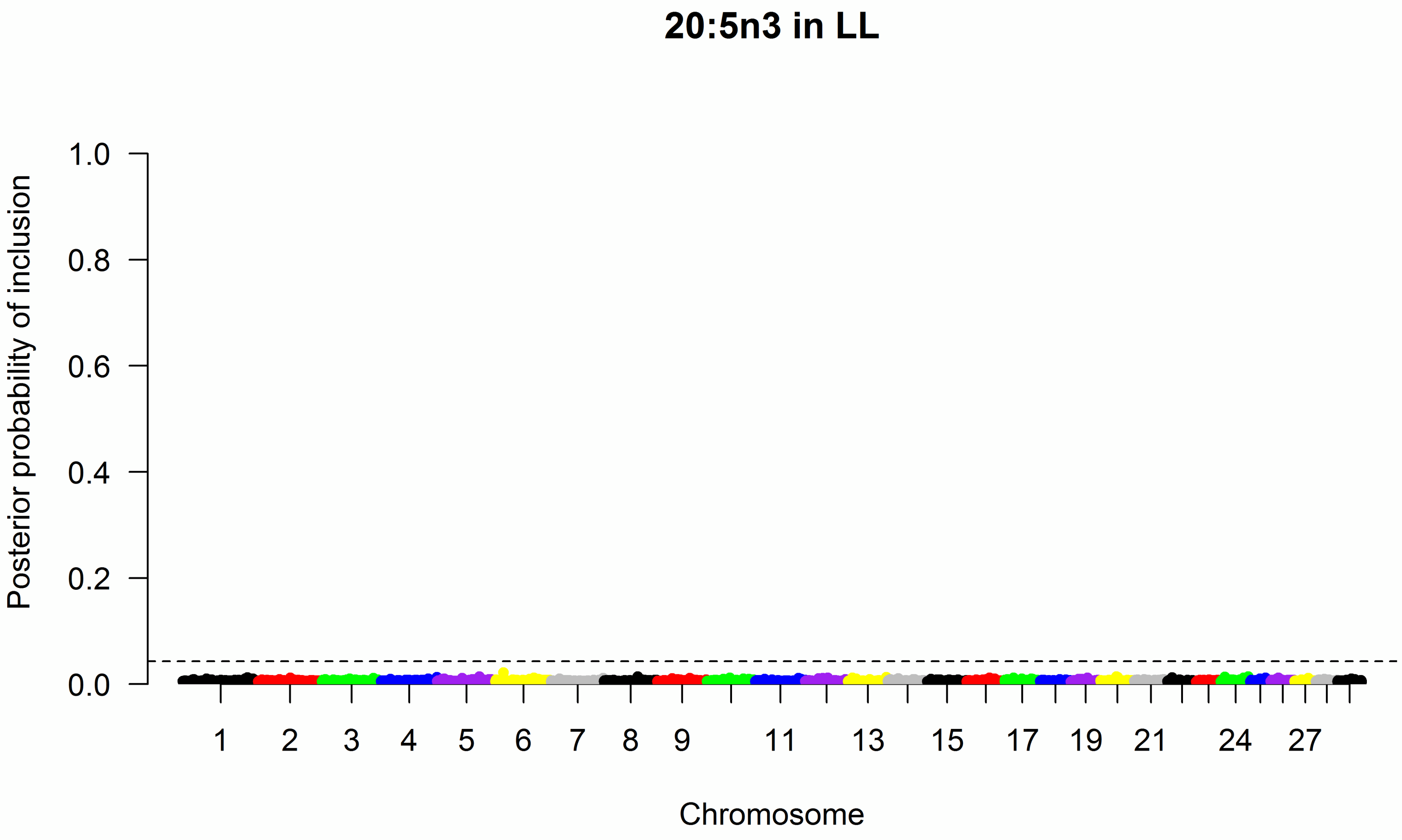
20:5n3 in LL
Posterior probability of inclusion
1.0
0.8
0.6
0.4
0.2
0.0
1
2
3
4
5
6
7
8
9
11
13
15
17
19
21
24
27
Chromosome

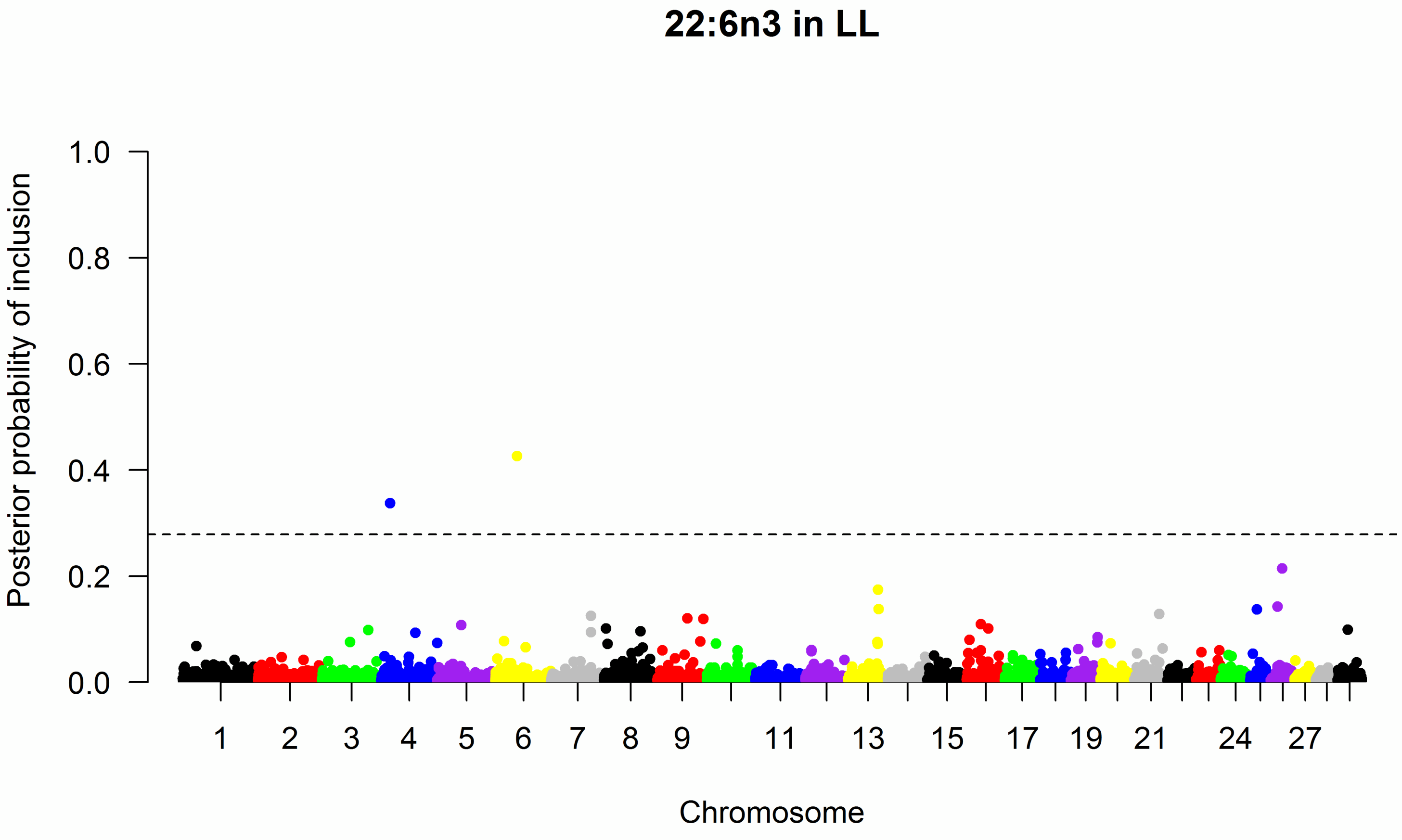
22:6n3 in LL
Posterior probability of inclusion
1.0
0.8
0.6
0.4
0.2
0.0
1
2
3
4
5
6
7
8
9
11
13
15
17
19
21
24
27
Chromosome

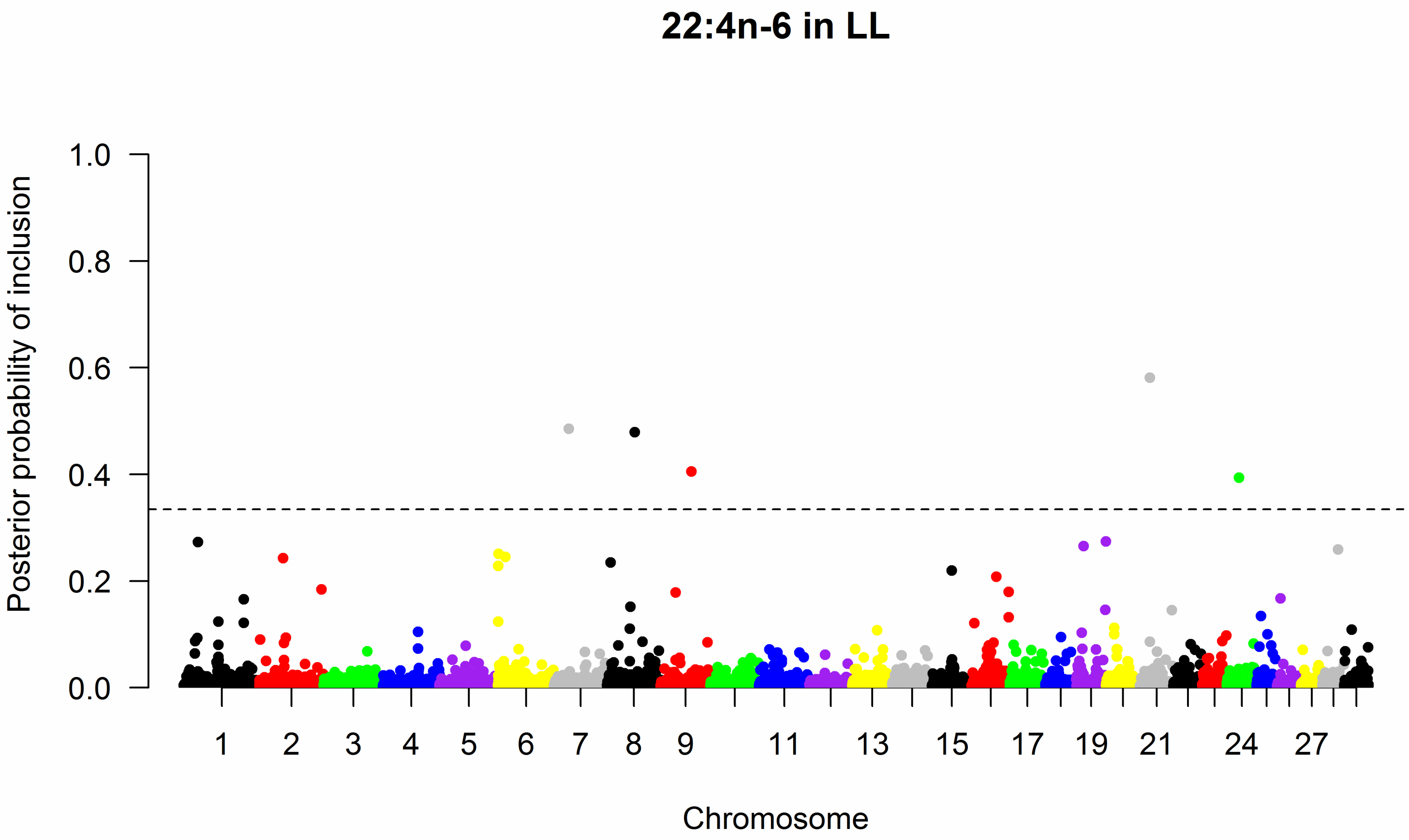
22:4n-6 in LL
Posterior probability of inclusion
1.0
0.8
0.6
0.4
0.2
0.0
1
2
3
4
5
6
7
8
9
11
13
15
17
19
21
24
27
Chromosome

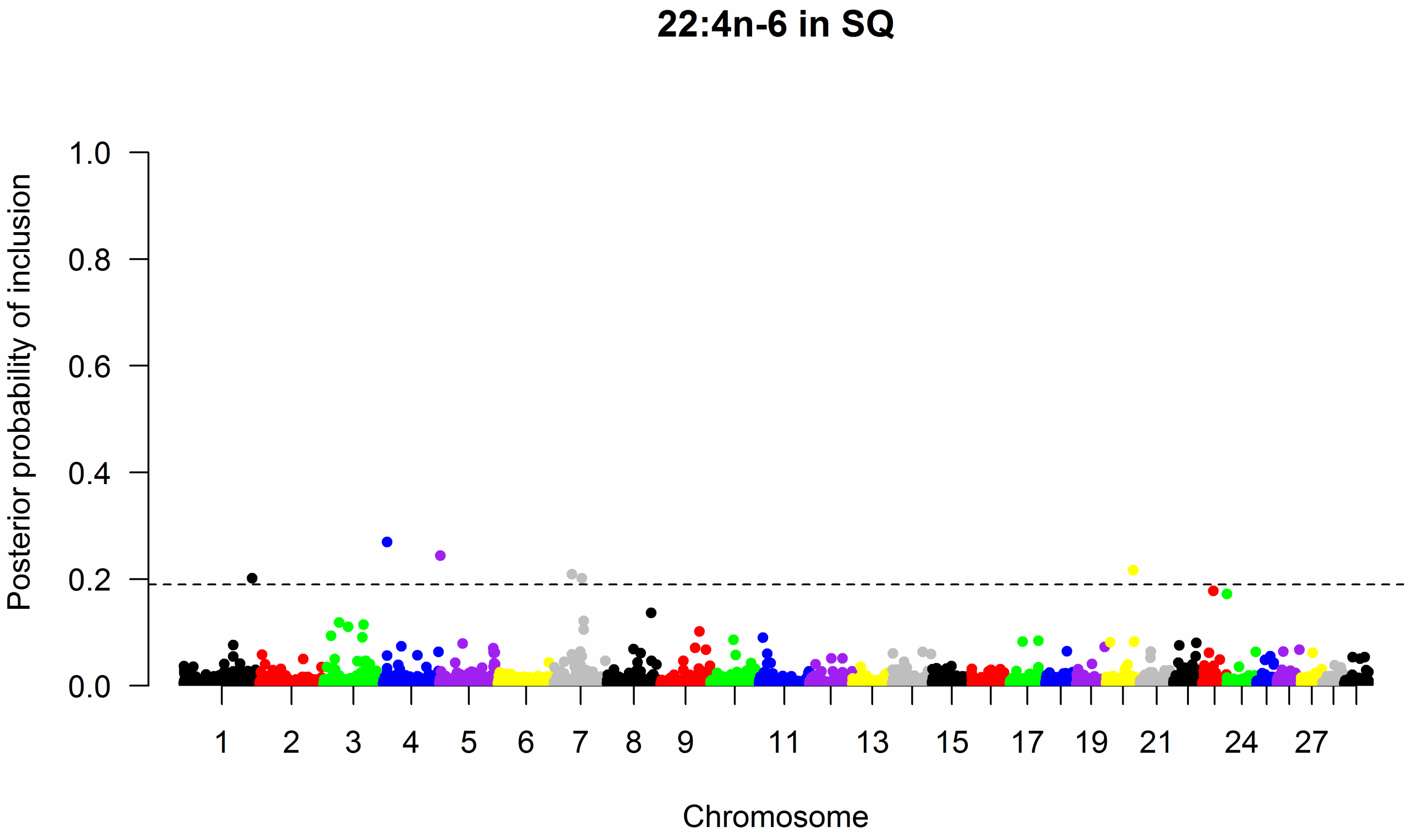
22:4n-6 in SQ
Posterior probability of inclusion
1.0
0.8
0.6
0.4
0.2
0.0
1
2
3
4
5
6
7
8
9
11
13
15
17
19
21
24
27
Chromosome

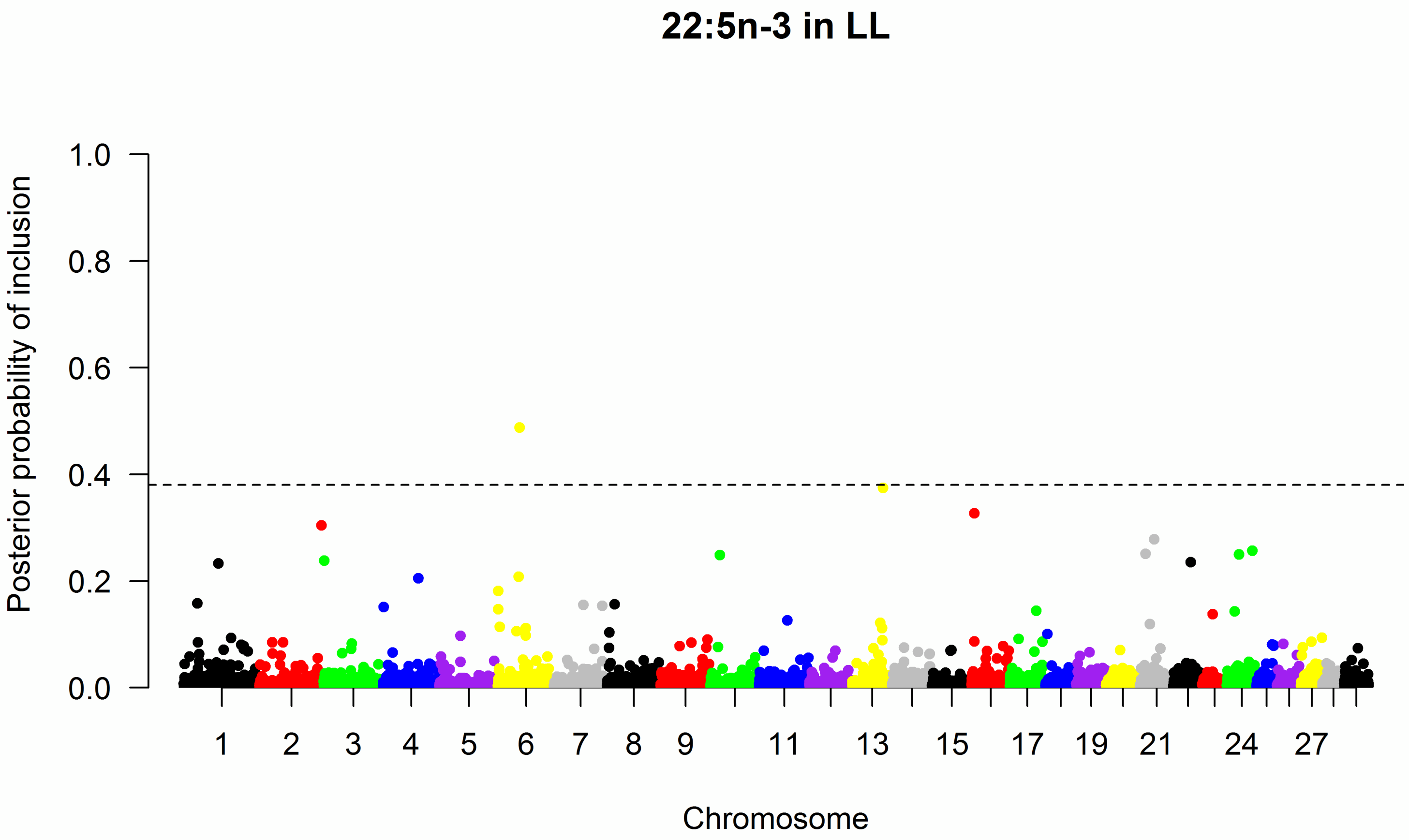

22:5n-3 in LL
Posterior probability of inclusion
1.0
0.8
0.6
0.4
0.2
0.0
1
2
3
4
5
6
7
8
9
11
13
15
17
19
21
24
27
Chromosome

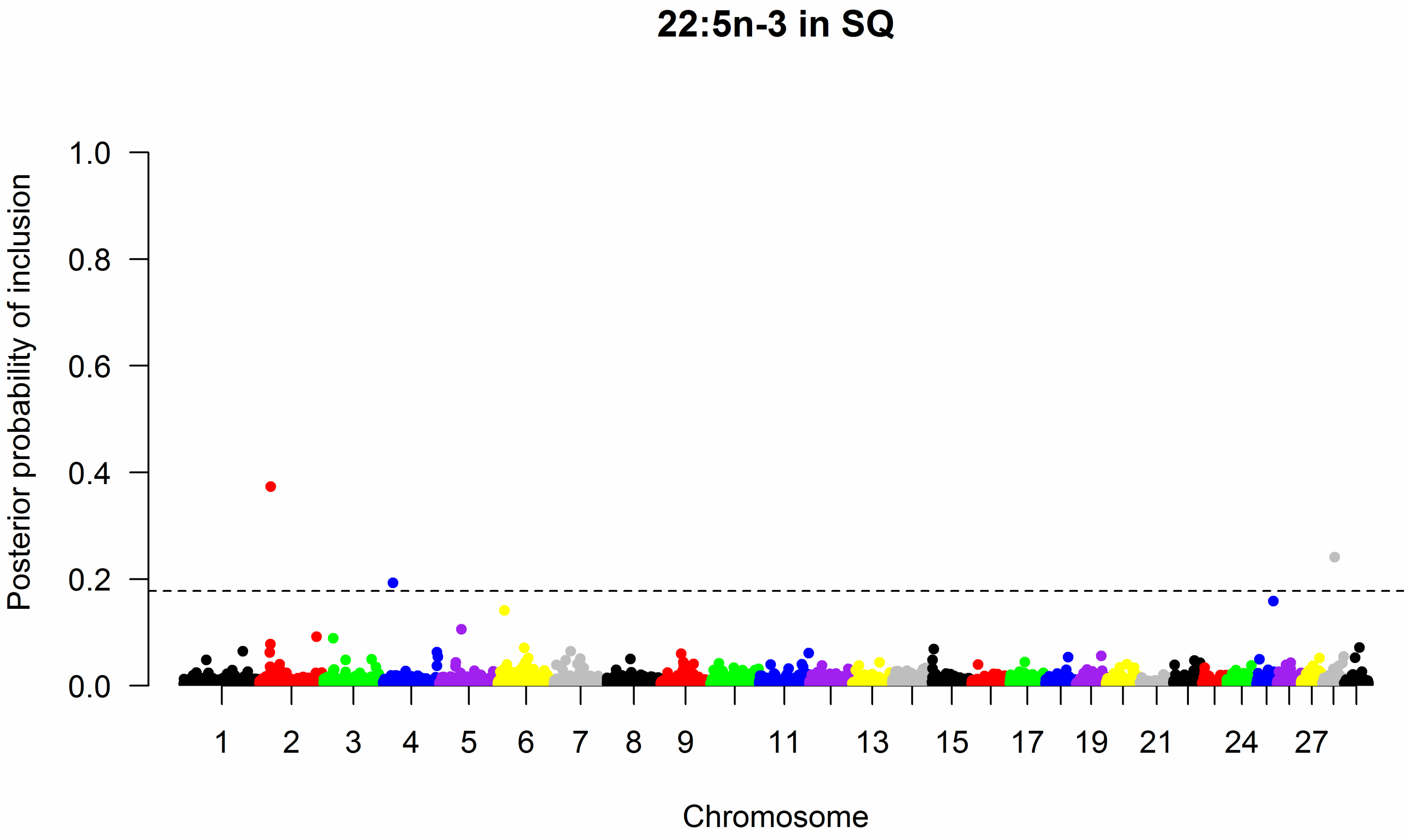

22:5n-3 in SQ
Posterior probability of inclusion
1.0
0.8
0.6
0.4
0.2
0.0
1
2
3
4
5
6
7
8
9
11
13
15
17
19
21
24
27
Chromosome

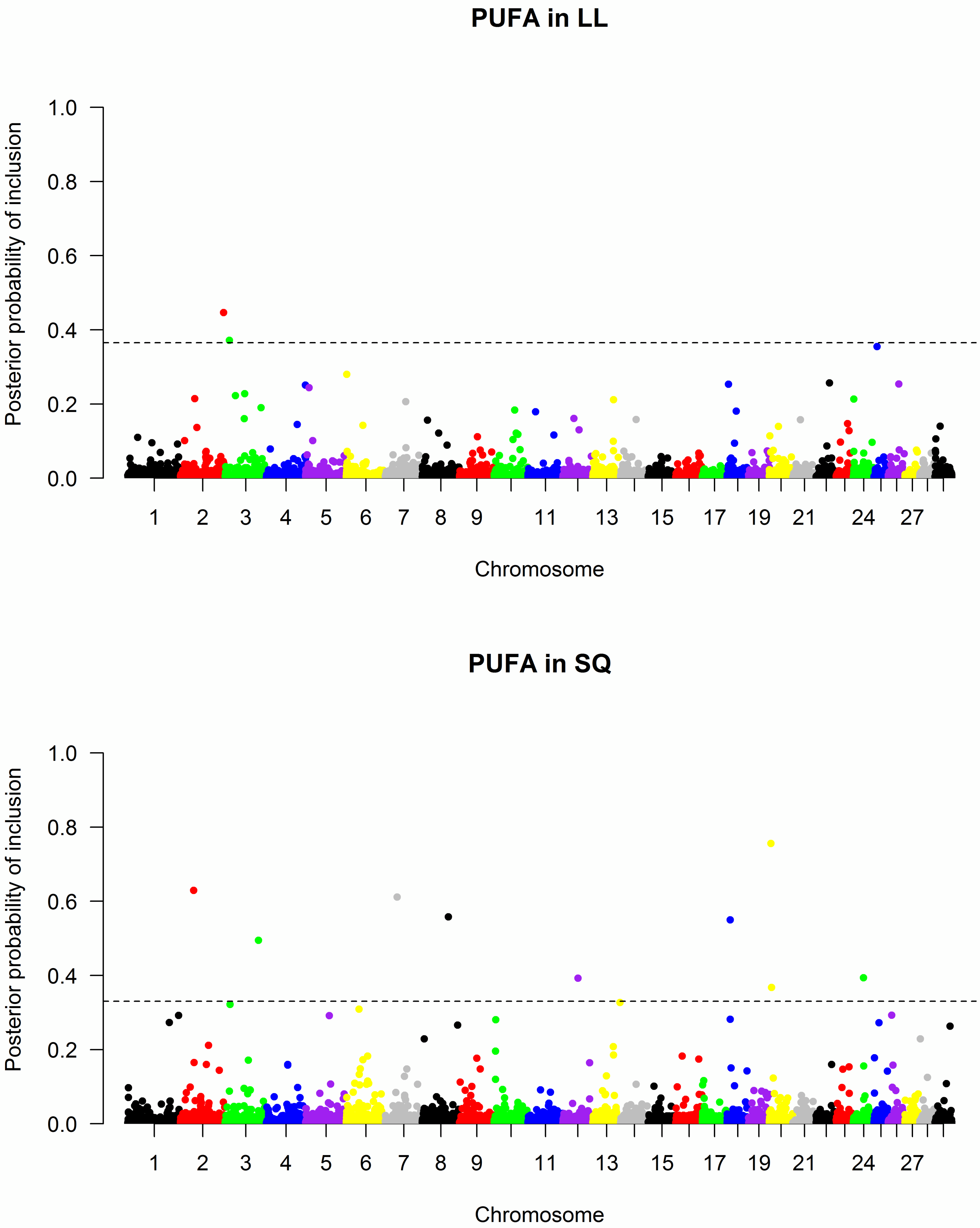
PUFA in LL
Posterior probability of inclusion
1.0
0.8
0.6
0.4
0.2
0.0
1
2
3
4
5
6
7
8
9
11
13
15
17
19
21
24
27
Chromosome
PUFA in SQ
Posterior probability of inclusion
1.0
0.8
0.6
0.4
0.2
0.0
1
2
3
4
5
6
7
8
9
11
13
15
17
19
21
24
27
Chromosome

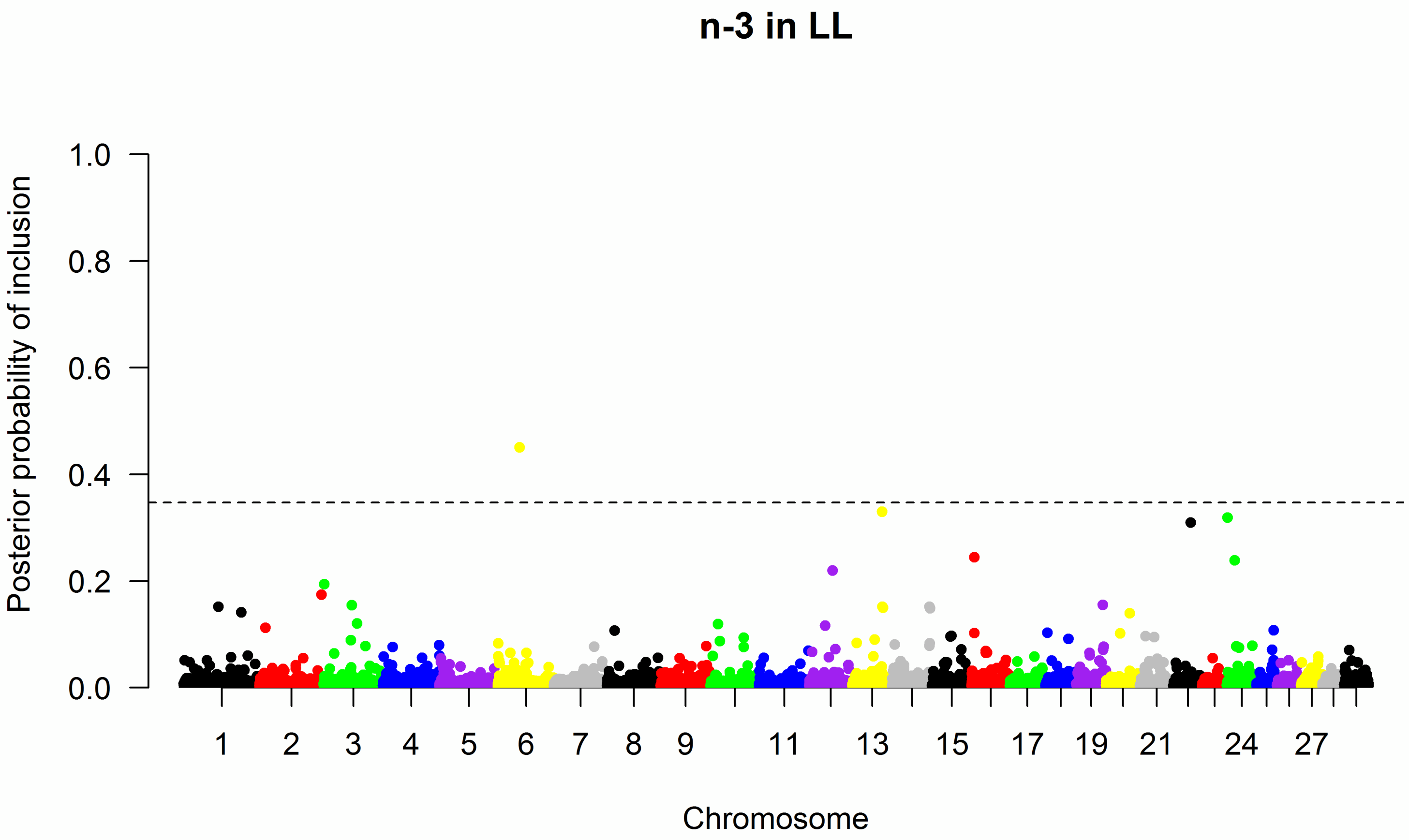
n-3 in LL
Posterior probability of inclusion
1.0
0.8
0.6
0.4
0.2
0.0
1
2
3
4
5
6
7
8
9
11
13
15
17
19
21
24
27
Chromosome

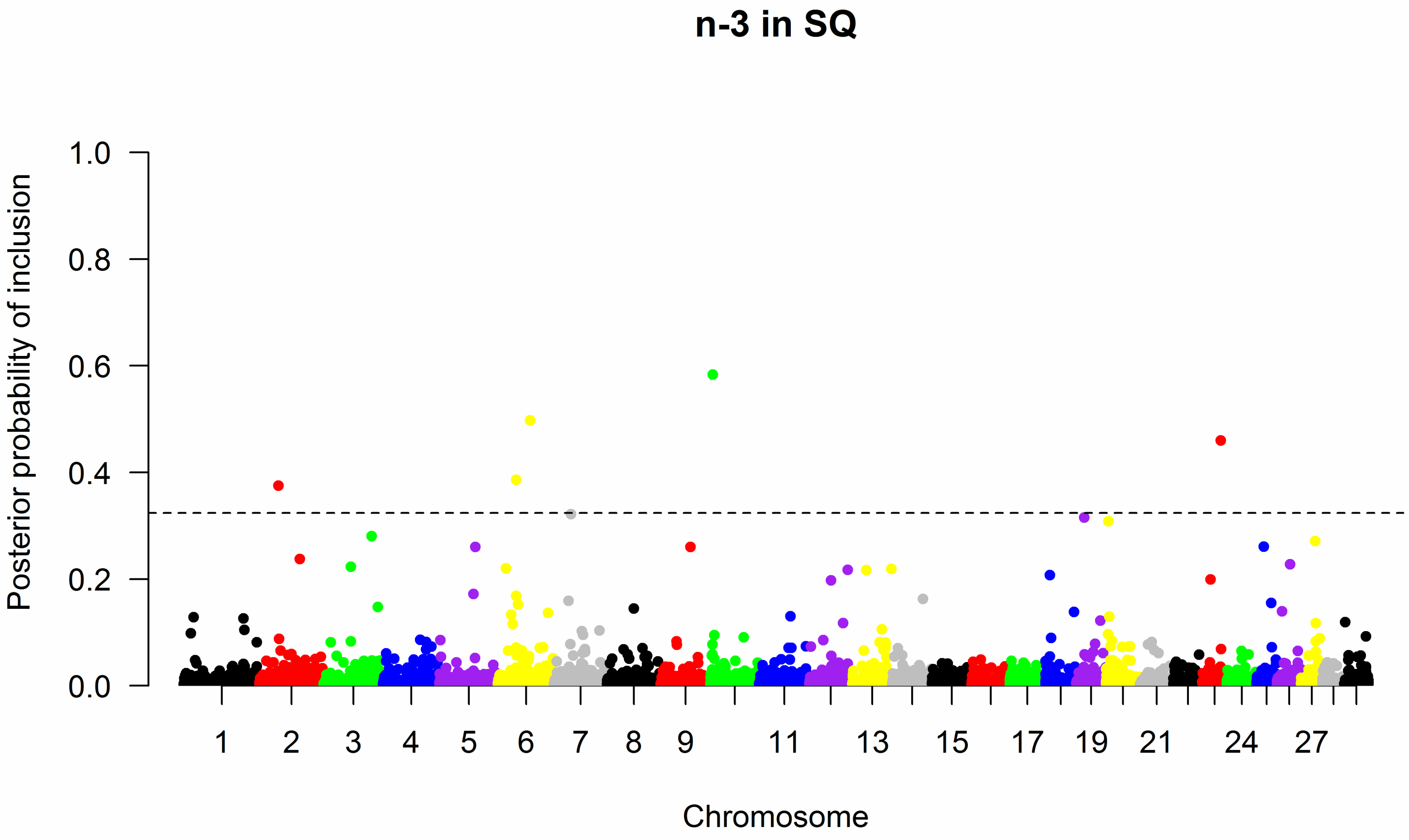
n-3 in SQ
Posterior probability of inclusion
1.0
0.8
0.6
0.4
0.2
0.0
1
2
3
4
5
6
7
8
9
11
13
15
17
19
21
24
27
Chromosome

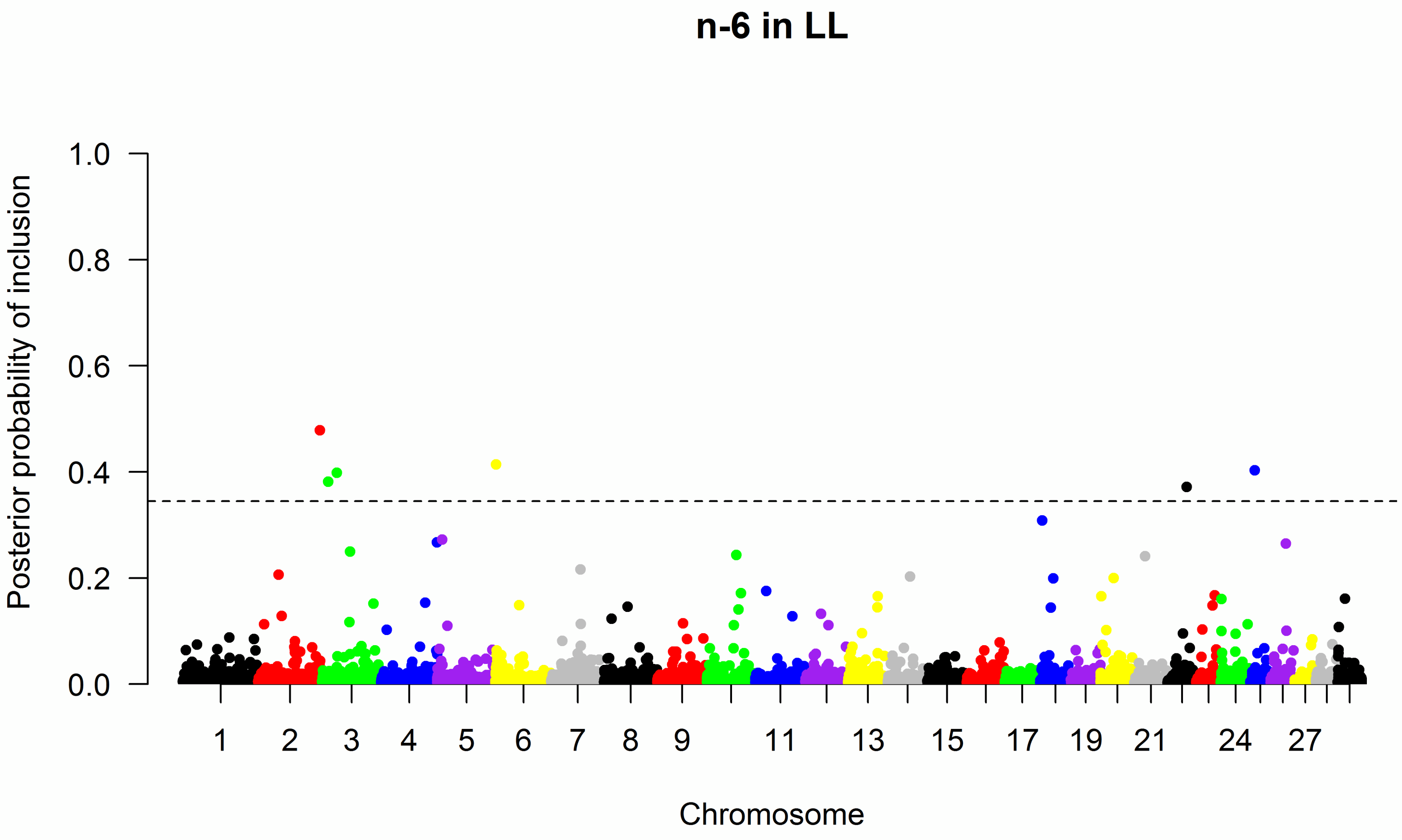
n-6 in LL
Posterior probability of inclusion
0.0
0.2
0.4
0.6
0.8
1.0
1
2
3
4
5
6
7
8
9
11
13
15
17
19
21
24
27
Chromosome

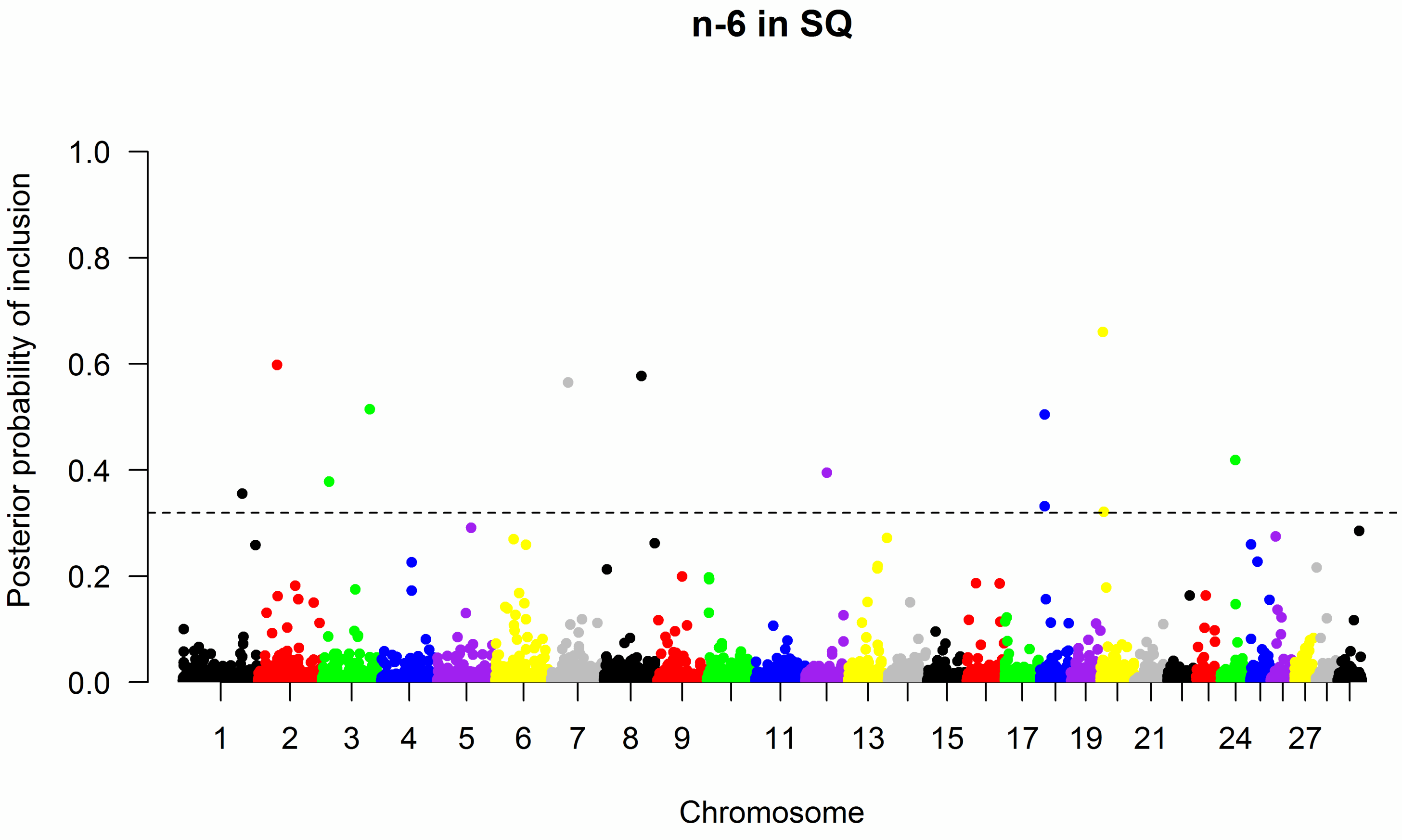
n-6 in SQ
Posterior probability of inclusion
0.0
0.2
0.4
0.6
0.8
1.0
1
2
3
4
5
6
7
8
9
11
13
15
17
19
21
24
27
Chromosome

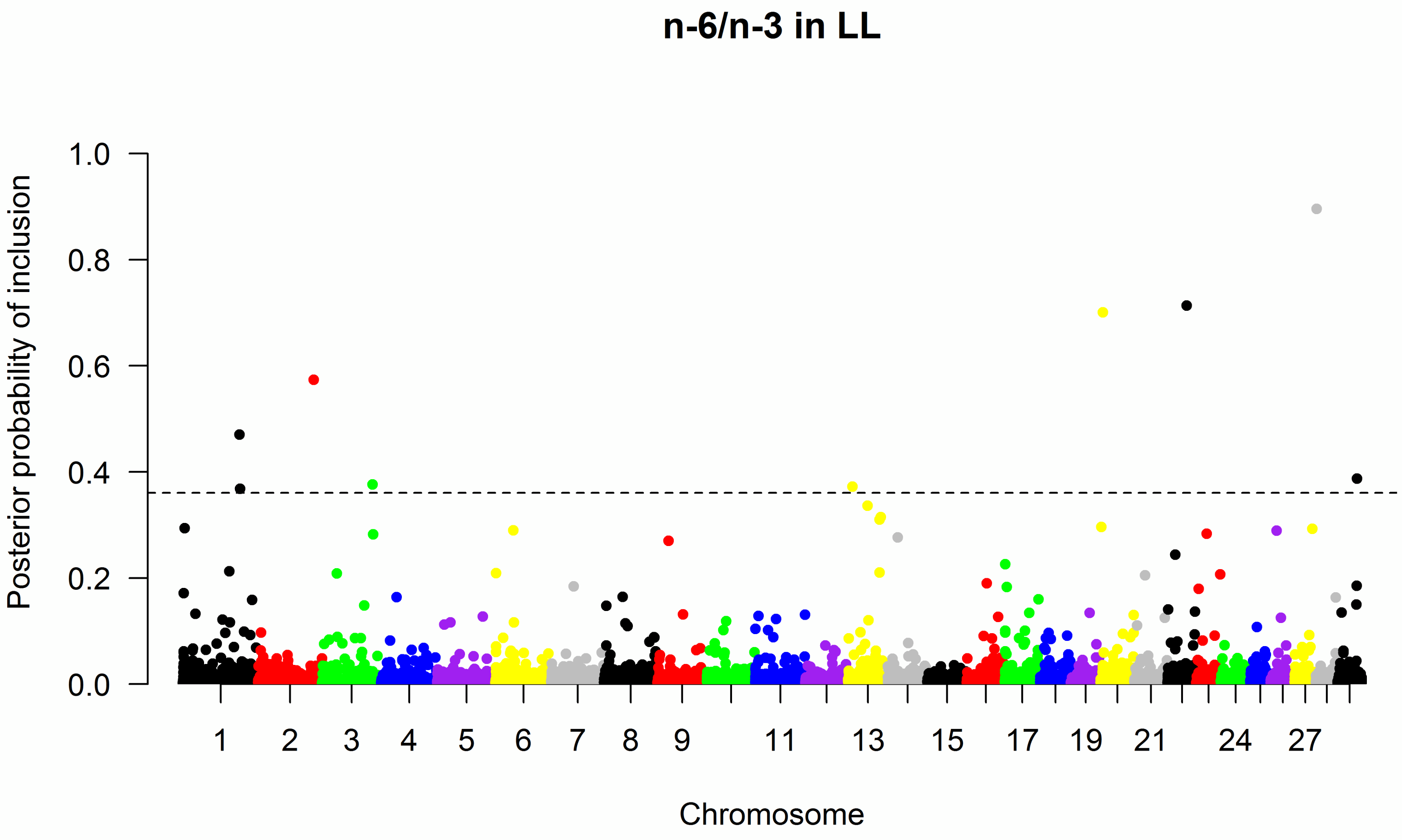
n-6/n-3 in LL
Posterior probability of inclusion
1.0
0.8
0.6
0.4
0.2
0.0
1
2
3
4
5
6
7
8
9
11
13
15
17
19
21
24
27
Chromosome

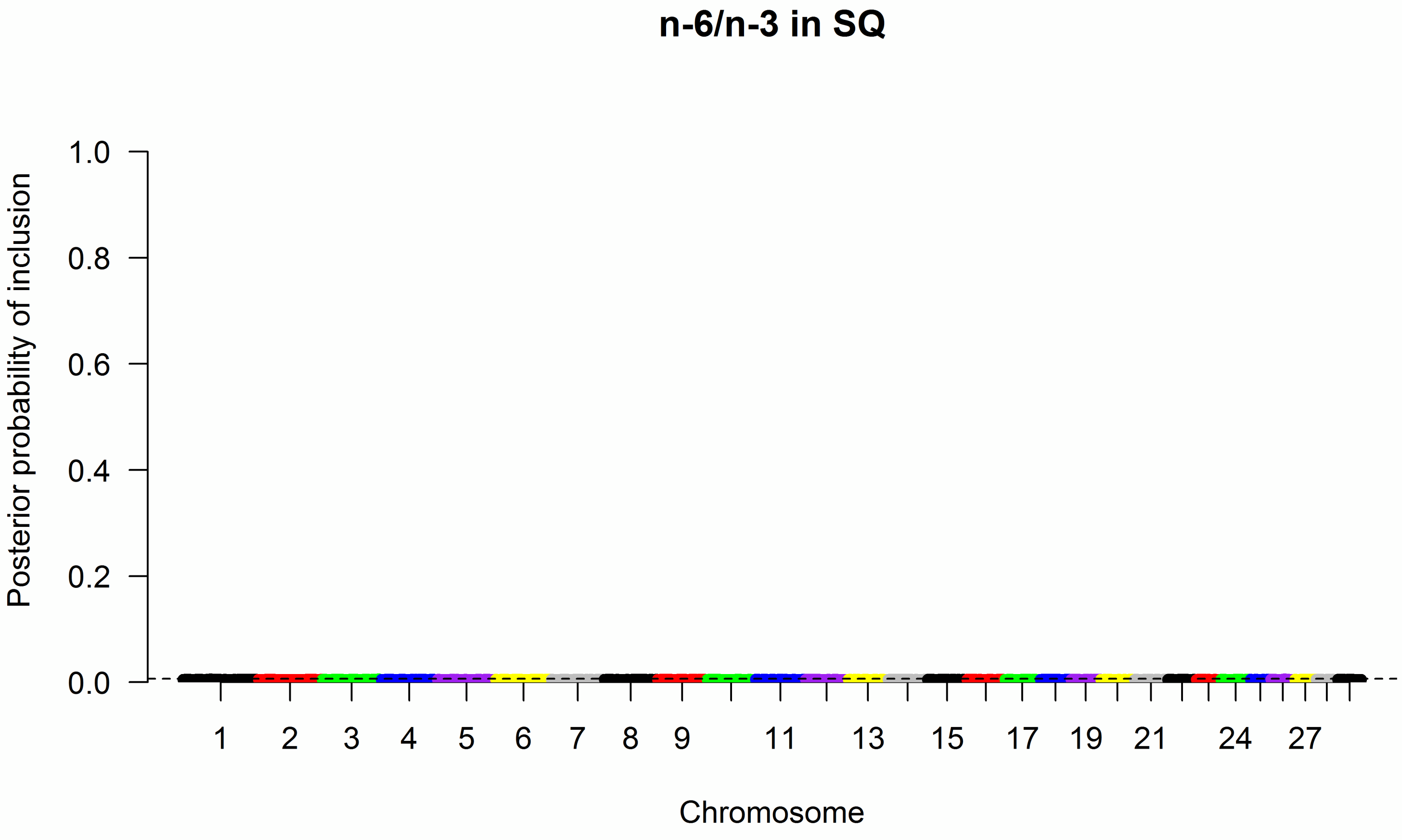
n-6/n-3 in SQ
Posterior probability of inclusion
1.0
0.8
0.6
0.4
0.2
0.0
1
2
3
4
5
6
7
8
9
11
13
15
17
19
21
24
27
Chromosome

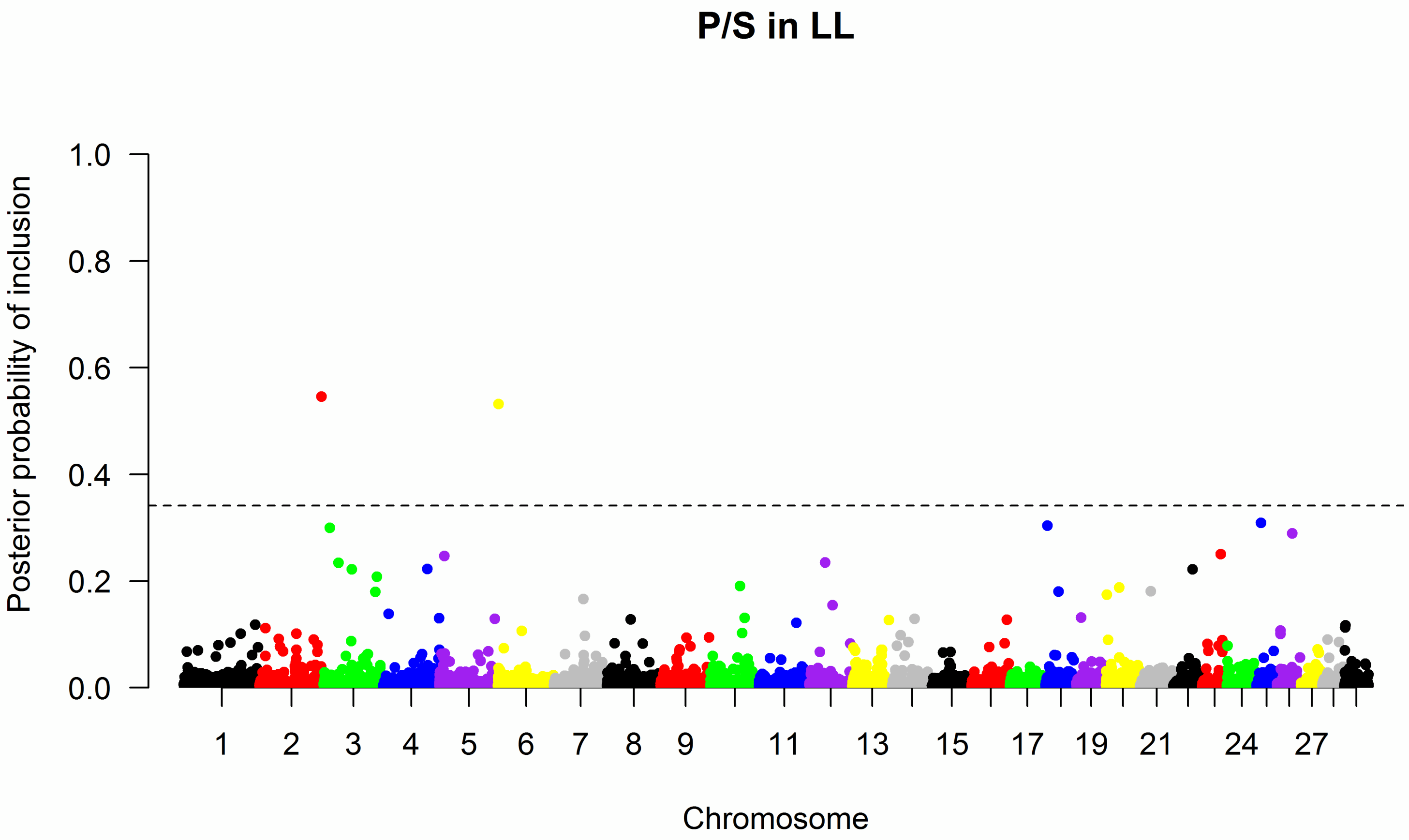
P/S in LL
Posterior probability of inclusion
1.0
0.8
0.6
0.4
0.2
0.0
1
2
3
4
5
6
7
8
9
11
13
15
17
19
21
24
27
Chromosome

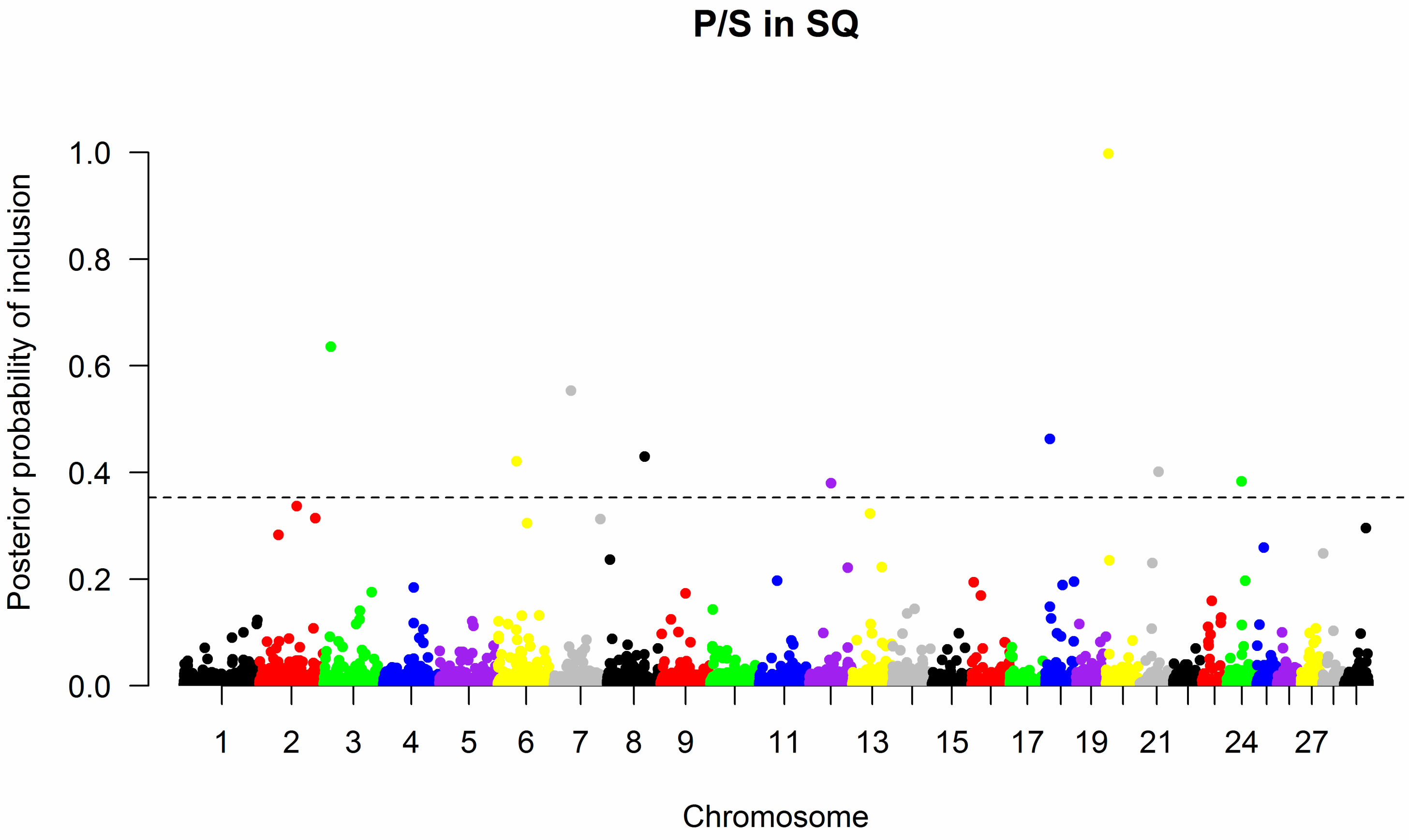
P/S in SQ
Posterior probability of inclusion
1.0
0.8
0.6
0.4
0.2
0.0
1
2
3
4
5
6
7
8
9
11
13
15
17
19
21
24
27
Chromosome

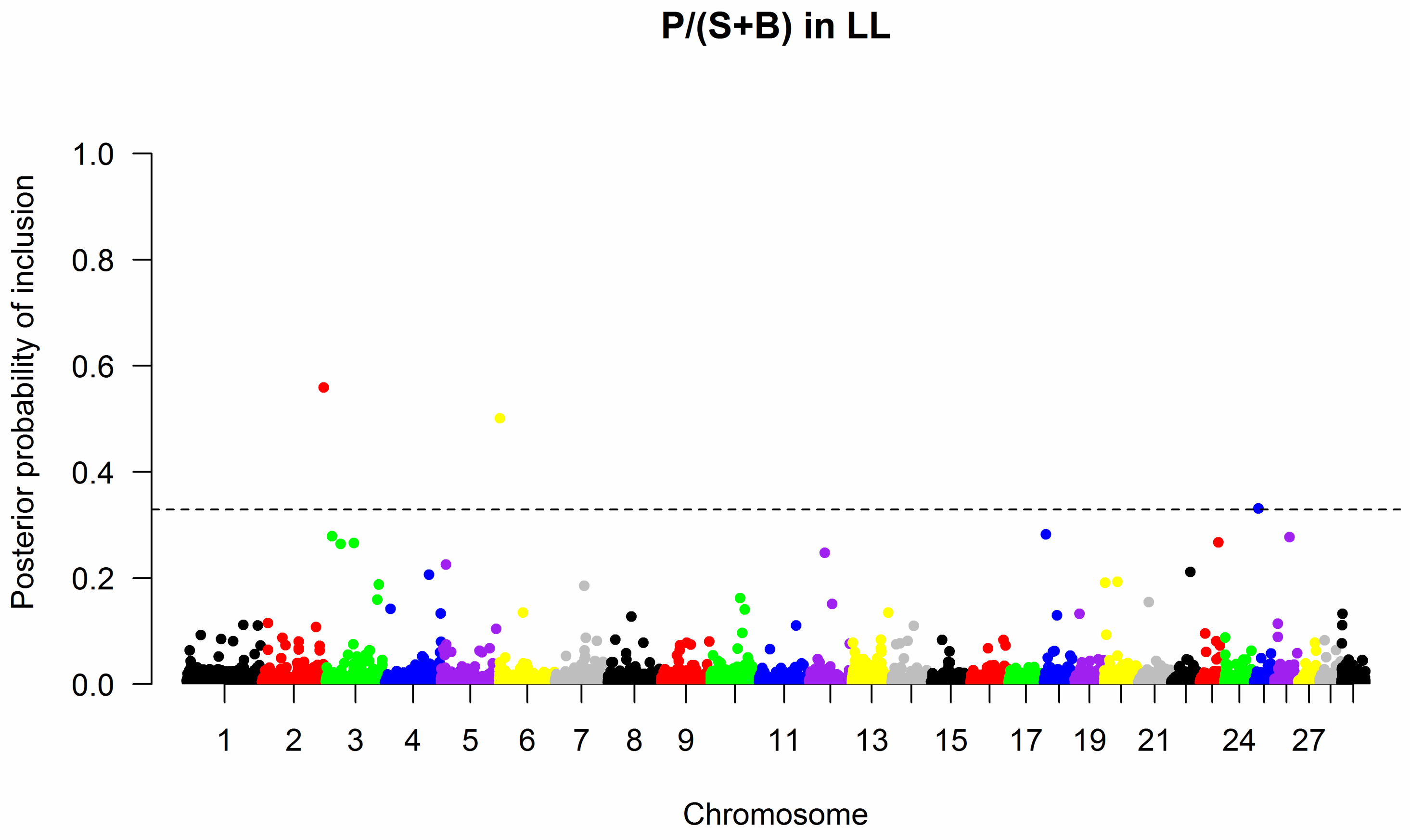
P/(S+B) in LL
Posterior probability of inclusion
1.0
0.8
0.6
0.4
0.2
0.0
1
2
3
4
5
6
7
8
9
11
13
15
17
19
21
24
27
Chromosome

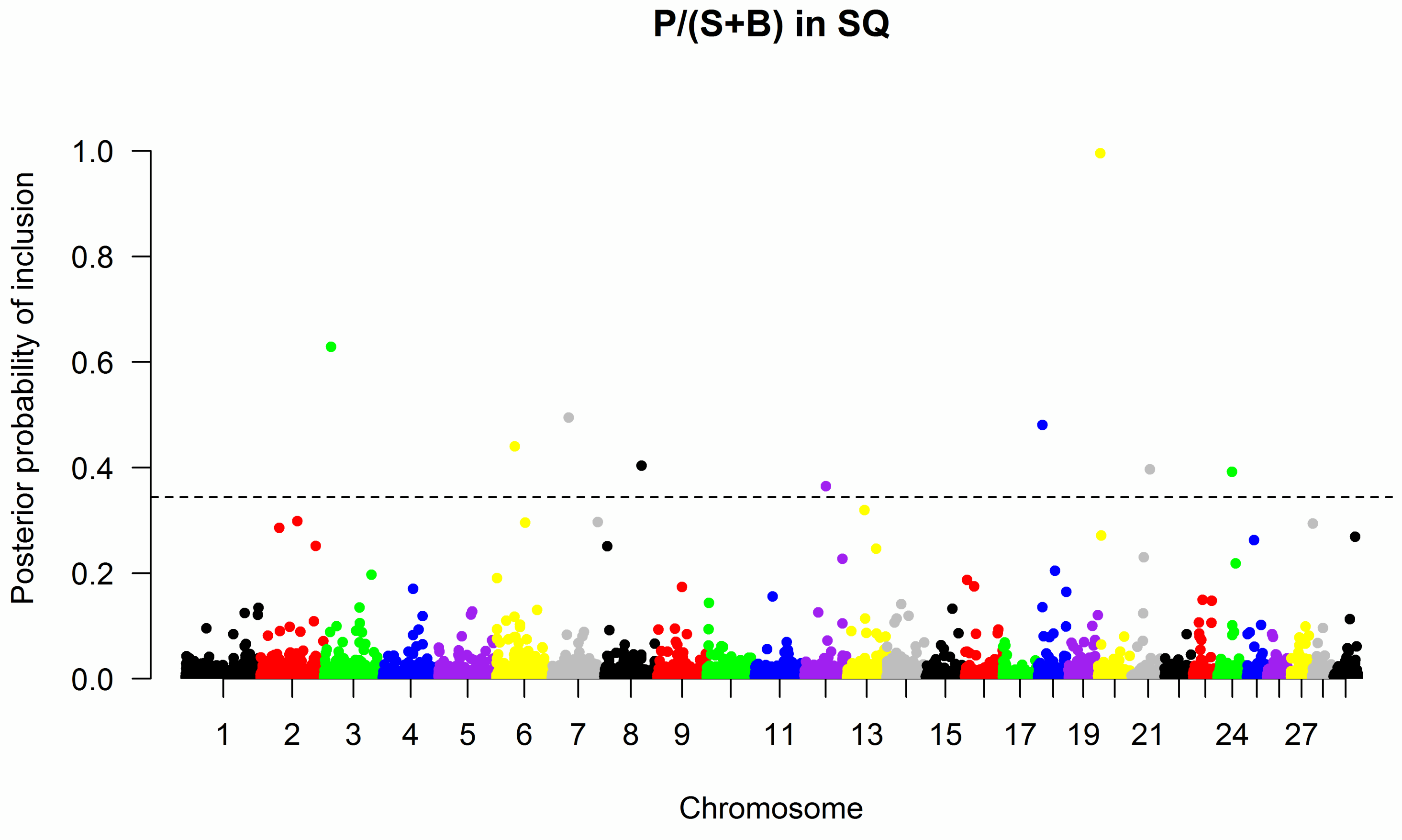
P/(S+B) in SQ
Posterior probability of inclusion
1.0
0.8
0.6
0.4
0.2
0.0
1
2
3
4
5
6
7
8
9
11
13
15
17
19
21
24
27
Chromosome

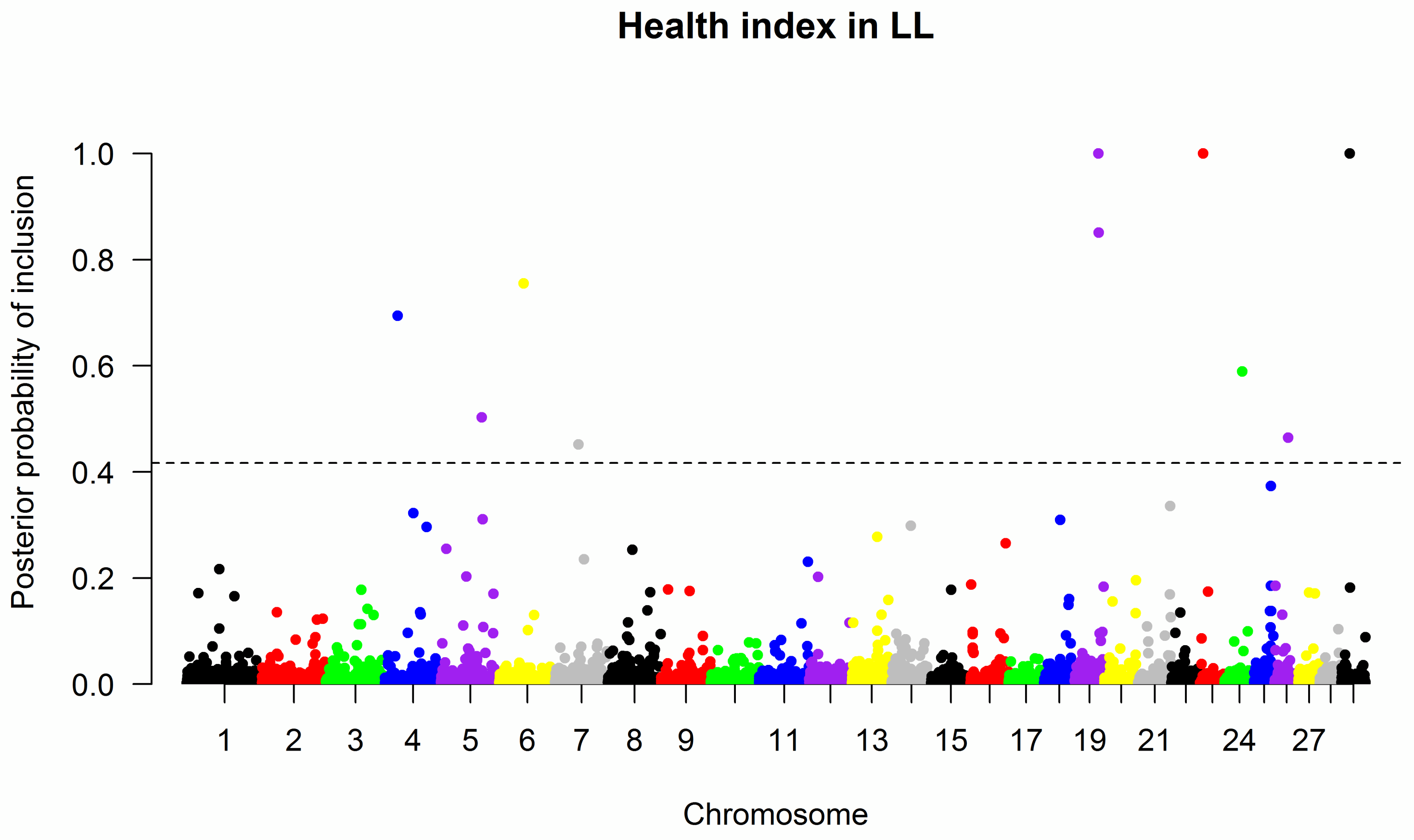
Health index in LL
Posterior probability of inclusion
1.0
0.8
0.6
0.4
0.2
0.0
1 2 3 4 5 6 7 8 9 11 13 15 17 19 21 24 27
Chromosome

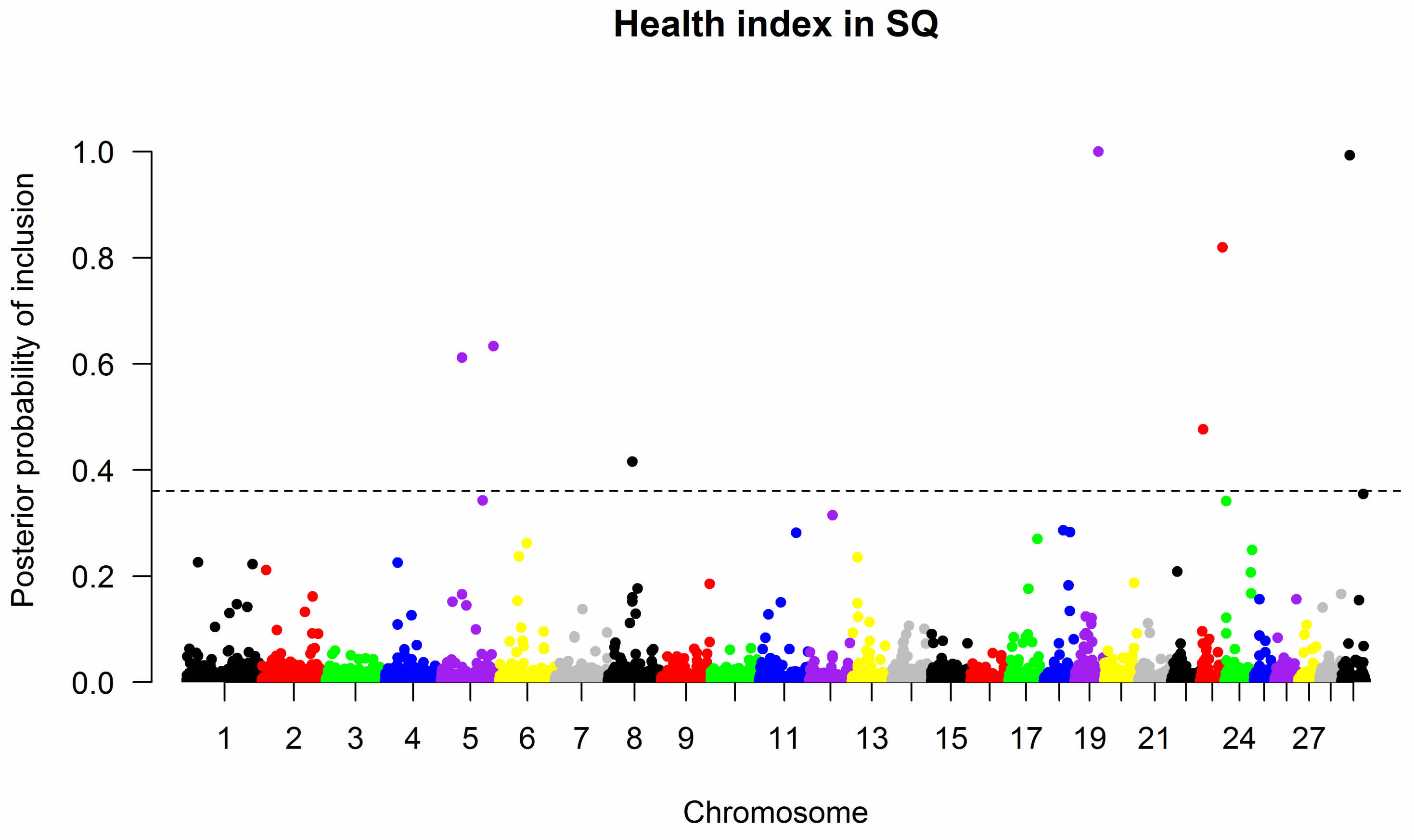
Health index in SQ
Posterior probability of inclusion
1.0
0.8
0.6
0.4
0.2
0.0
1 2 3 4 5 6 7 8 9 11 13 15 17 19 21 24 27
Chromosome
