# Supplementary figures and images for: Genome-wide association and genomic prediction of breeding values for fatty acid composition in subcutaneous adipose and longissimus lumborum muscle of beef cattle
Source: BMC Genet. 2015 Nov 21;16:135. doi: 10.1186/s12863-015-0290-0 (PMC4654876; doi:10.1186/s12863-015-0290-0)

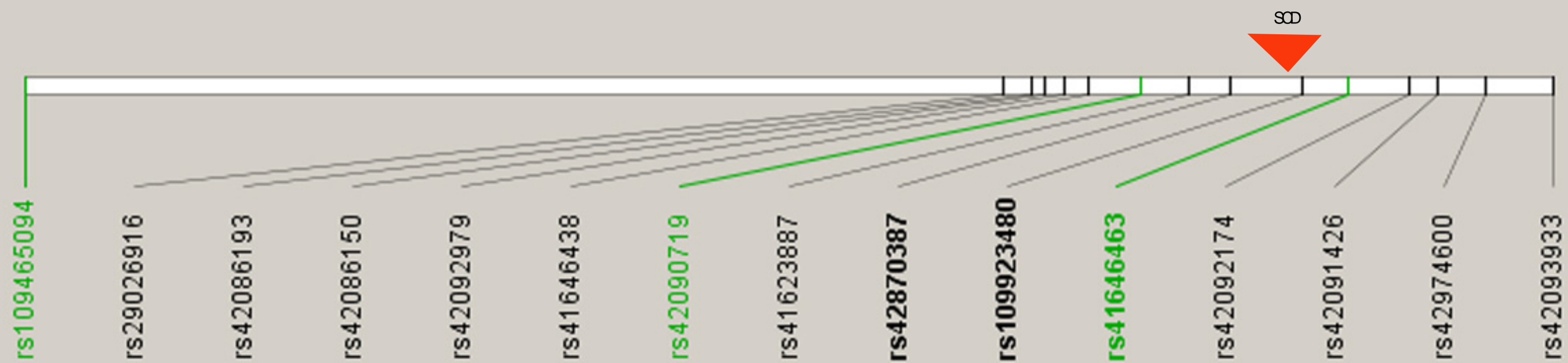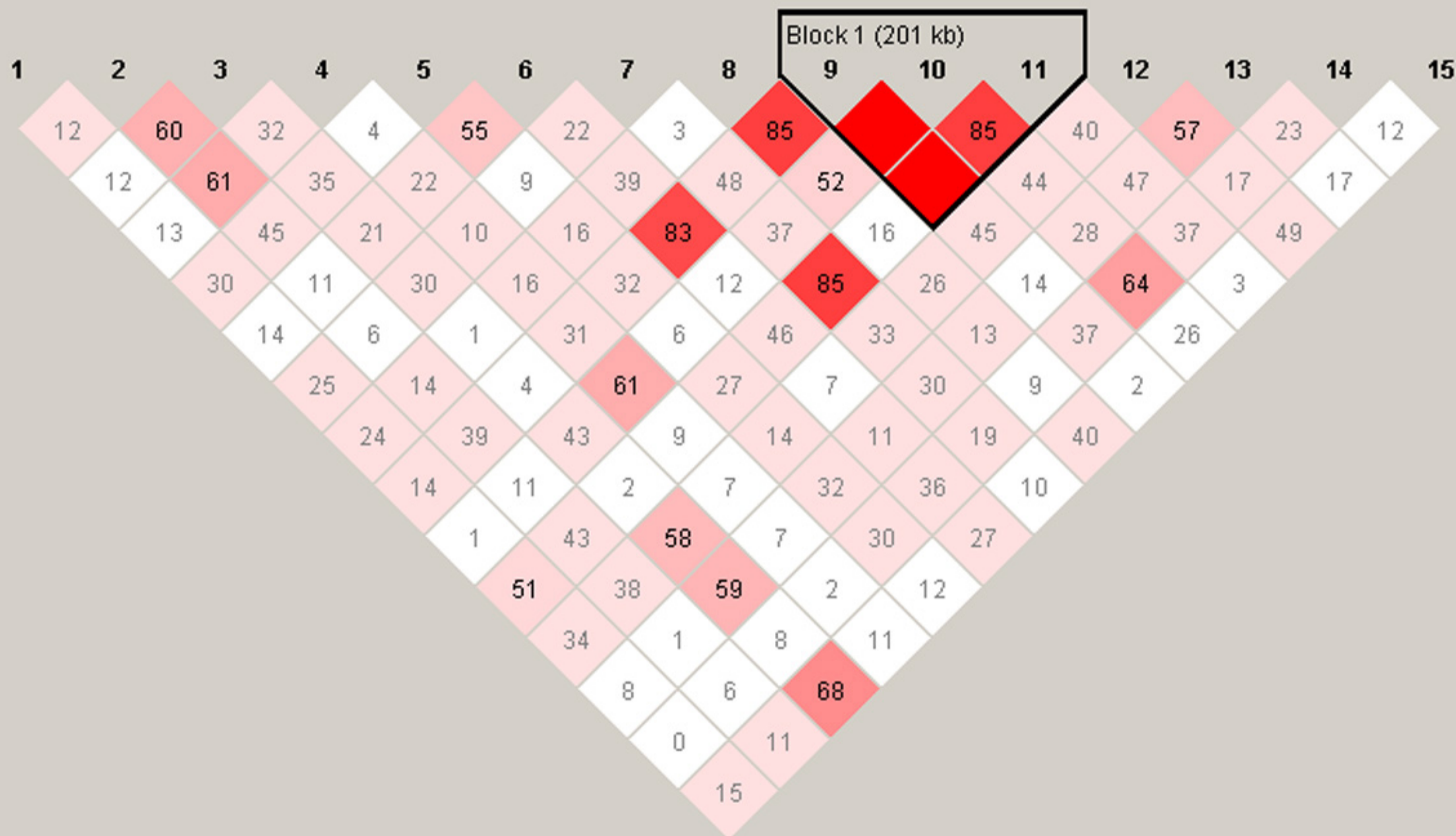

Supplement: Additional file 5: — Linkage disequilibrium (LD) beween single nucleotide polymorphisms (SNP) around the SCD gene. Linkage disequilibrium was measured as D prime. The red triangle indicates the location of the SCD gene. An LD block was shown surrounding the SCD gene. Names of the SNPs significantly associated with fatty acid composition were highlighted in green. (PDF 971 kb) [file 12863_2015_290_MOESM5_ESM.pdf]
