# Supplementary material for: Genome-wide association and genomic prediction of breeding values for fatty acid composition in subcutaneous adipose and longissimus lumborum muscle of beef cattle
Source: BMC Genet. 2015 Nov 21;16:135. doi: 10.1186/s12863-015-0290-0 (PMC4654876; doi:10.1186/s12863-015-0290-0)
Supplement: Additional file 6: — Pearson’s correlation coefficients between estimated breeding values and adjusted phenotypes and regression coefficients of adjusted phenotypes on estimated breeding values. (DOCX 35 kb) [file 12863_2015_290_MOESM6_ESM.docx]

Table S1. Pearson’s correlation coefficients between estimated breeding values and adjusted phenotypes [$r_{\left( GEBV,y \right)}$] and regression coefficients by regressing adjusted phenotypes on estimated breeding values [$b_{\left( GEBV,y \right)}$] for fatty acid traits in the subcutaneous adipose tissue.

|  | PBLUP | | GBLUP | | BayesCπ | |
| --- | --- | --- | --- | --- | --- | --- |
| Trait^2^ | $r_{\left( GEBV,y \right)}$ | $b_{\left( GEBV,y \right)}$ | $r_{\left( GEBV,y \right)}$ | $b_{\left( GEBV,y \right)}$ | $r_{\left( GEBV,y \right)}$ | $b_{\left( GEBV,y \right)}$ |
| 10:0 | 0.21 ± 0.03 | 1.10 ± 0.16 | 0.24 ± 0.04 | 0.80 ± 0.12 | 0.30 ± 0.03 | 1.02 ± 0.14 |
| 12:0 | 0.19 ± 0.03 | 1.05 ± 0.15 | 0.31 ± 0.03 | 0.86 ± 0.07 | 0.42 ± 0.02 | 1.07 ± 0.08 |
| 13:0 | 0.03 ± 0.03 | 0.30 ± 0.36 | 0.18 ± 0.02 | 0.90 ± 0.14 | 0.17 ± 0.02 | 0.97 ± 0.15 |
| 14:0 | 0.20 ± 0.03 | 1.00 ± 0.13 | 0.23 ± 0.03 | 0.56 ± 0.06 | 0.36 ± 0.02 | 0.93 ± 0.06 |
| 15:0 | 0.18 ± 0.03 | 1.05 ± 0.19 | 0.26 ± 0.03 | 0.75 ± 0.10 | 0.29 ± 0.03 | 0.92 ± 0.11 |
| 16:0 | 0.15 ± 0.02 | 1.27 ± 0.22 | 0.13 ± 0.02 | 0.67 ± 0.14 | 0.14 ± 0.03 | 1.11 ± 0.22 |
| 17:0 | 0.17 ± 0.03 | 1.02 ± 0.21 | 0.19 ± 0.03 | 0.55 ± 0.10 | 0.19 ± 0.03 | 0.63 ± 0.10 |
| 18:0 | 0.09 ± 0.03 | 0.65 ± 0.23 | 0.19 ± 0.04 | 0.69 ± 0.16 | 0.19 ± 0.04 | 0.77 ± 0.19 |
| 19:0 | 0.08 ± 0.03 | 1.16 ± 0.32 | 0.10 ± 0.02 | 0.91 ± 0.21 | 0.10 ± 0.03 | 1.25 ± 0.36 |
| 20:0 | 0.02 ± 0.03 | 0.15 ± 0.34 | 0.10 ± 0.04 | 0.60 ± 0.21 | 0.10 ± 0.04 | 0.72 ± 0.28 |
| 22:0 | 0.04 ± 0.02 | 2.65 ± 1.94 | -0.03 ± 0.03 | -1.18 ± 1.06 | -0.03 ± 0.03 | -1.70 ± 1.08 |
| 24:0 | 0.11 ± 0.03 | 0.99 ± 0.28 | 0.17 ± 0.02 | 0.92 ± 0.12 | 0.18 ± 0.02 | 1.07 ± 0.15 |
| SFA | 0.15 ± 0.03 | 1.08 ± 0.24 | 0.15 ± 0.04 | 0.56 ± 0.16 | 0.16 ± 0.04 | 0.77 ± 0.20 |
| iso14:0 | 0.06 ± 0.03 | 0.67 ± 0.63 | 0.09 ± 0.03 | 0.68 ± 0.33 | 0.08 ± 0.03 | 1.09 ± 0.48 |
| iso15:0 | 0.10 ± 0.02 | 1.59 ± 0.46 | 0.12 ± 0.03 | 0.98 ± 0.27 | 0.12 ± 0.03 | 1.34 ± 0.40 |
| ai15:0 | 0.15 ± 0.02 | 1.16 ± 0.13 | 0.21 ± 0.03 | 0.79 ± 0.07 | 0.22 ± 0.03 | 0.84 ± 0.08 |
| iso16:0 | 0.12 ± 0.04 | 1.38 ± 0.46 | 0.16 ± 0.03 | 0.86 ± 0.20 | 0.16 ± 0.03 | 0.91 ± 0.20 |
| iso17:0 | 0.10 ± 0.03 | 1.24 ± 0.44 | 0.15 ± 0.03 | 0.96 ± 0.18 | 0.15 ± 0.03 | 0.89 ± 0.15 |
| ai17:0 | 0.06 ± 0.03 | 0.78 ± 0.50 | 0.15 ± 0.04 | 1.25 ± 0.31 | 0.14 ± 0.04 | 1.30 ± 0.38 |
| iso18:0 | 0.06 ± 0.02 | 0.59 ± 0.27 | 0.11 ± 0.02 | 0.59 ± 0.15 | 0.11 ± 0.02 | 0.64 ± 0.16 |
| BFA | 0.11 ± 0.03 | 1.32 ± 0.40 | 0.17 ± 0.03 | 1.05 ± 0.16 | 0.17 ± 0.03 | 1.05 ± 0.21 |
| SFA+BFA | 0.16 ± 0.03 | 1.12 ± 0.24 | 0.15 ± 0.04 | 0.58 ± 0.16 | 0.16 ± 0.04 | 0.85 ± 0.22 |
| 9c-14:1 | 0.12 ± 0.04 | 0.82 ± 0.25 | 0.20 ± 0.03 | 0.79 ± 0.09 | 0.28 ± 0.03 | 1.00 ± 0.12 |
| 9c-15:1 | 0.08 ± 0.03 | 0.77 ± 0.29 | 0.11 ± 0.03 | 0.51 ± 0.20 | 0.11 ± 0.04 | 0.57 ± 0.25 |
| 7c-16:1 | 0.11 ± 0.03 | 1.27 ± 0.34 | 0.10 ± 0.04 | 0.62 ± 0.24 | 0.11 ± 0.04 | 0.68 ± 0.22 |
| 9c-16:1 | 0.09 ± 0.02 | 0.60 ± 0.15 | 0.16 ± 0.04 | 0.54 ± 0.13 | 0.17 ± 0.04 | 0.72 ± 0.19 |
| 11t-16:1 | 0.05 ± 0.03 | 0.82 ± 0.40 | 0.11 ± 0.02 | 1.01 ± 0.22 | 0.11 ± 0.02 | 1.09 ± 0.21 |
| 12c-16:1 | 0.13 ± 0.03 | 1.01 ± 0.21 | 0.21 ± 0.02 | 0.93 ± 0.08 | 0.26 ± 0.02 | 1.06 ± 0.09 |
| 7c-17:1 | 0.03 ± 0.02 | 0.32 ± 0.22 | 0.05 ± 0.02 | 0.24 ± 0.09 | 0.04 ± 0.02 | 0.62 ± 0.30 |
| 9c-17:1 | 0.15 ± 0.03 | 1.14 ± 0.29 | 0.21 ± 0.02 | 0.86 ± 0.13 | 0.21 ± 0.02 | 0.88 ± 0.13 |
| 9c-18:1 | 0.13 ± 0.03 | 1.60 ± 0.54 | 0.11 ± 0.04 | 0.72 ± 0.29 | 0.11 ± 0.04 | 1.27 ± 0.42 |
| 11c-18:1 | 0.13 ± 0.04 | 5.74 ± 3.10 | 0.13 ± 0.04 | 1.09 ± 0.41 | 0.13 ± 0.04 | 1.65 ± 0.51 |
| 12c-18:1 | 0.00 ± 0.03 | -0.47 ± 0.63 | 0.03 ± 0.03 | 0.39 ± 0.28 | 0.03 ± 0.03 | 0.43 ± 0.23 |
| 13c-18:1 | 0.13 ± 0.03 | 1.00 ± 0.31 | 0.24 ± 0.03 | 1.13 ± 0.10 | 0.27 ± 0.03 | 1.11 ± 0.10 |
| 14c-18:1 | 0.03 ± 0.03 | 0.79 ± 0.66 | 0.10 ± 0.04 | 1.04 ± 0.41 | 0.10 ± 0.04 | 1.11 ± 0.44 |
| 15c-18:1 | 0.12 ± 0.03 | 1.34 ± 0.24 | 0.19 ± 0.02 | 1.19 ± 0.15 | 0.19 ± 0.02 | 1.01 ± 0.16 |
| 6t+8t-18:1 | 0.17 ± 0.03 | 1.68 ± 0.21 | 0.21 ± 0.02 | 1.19 ± 0.15 | 0.20 ± 0.02 | 1.06 ± 0.14 |
| 9t-18:1 | 0.16 ± 0.04 | 1.54 ± 0.32 | 0.17 ± 0.03 | 1.13 ± 0.19 | 0.17 ± 0.03 | 1.01 ± 0.17 |
| 10t-18:1 | 0.17 ± 0.02 | 1.44 ± 0.15 | 0.26 ± 0.02 | 1.16 ± 0.13 | 0.26 ± 0.02 | 1.02 ± 0.14 |
| 11t-18:1 | 0.14 ± 0.03 | 1.25 ± 0.26 | 0.18 ± 0.04 | 1.10 ± 0.25 | 0.17 ± 0.04 | 1.12 ± 0.25 |
| 12t-18:1 | 0.00 ± 0.02 | 0.15 ± 0.91 | -0.03 ± 0.03 | -0.02 ± 0.04 | -0.03 ± 0.03 | -0.34 ± 0.23 |
| 15t-18:1 | 0.03 ± 0.07 | -0.45 ± 2.47 | 0.03 ± 0.05 | 0.44 ± 0.84 | 0.03 ± 0.04 | 2.07 ± 2.50 |
| 16t-18:1 | 0.14 ± 0.02 | 1.60 ± 0.29 | 0.22 ± 0.04 | 1.54 ± 0.32 | 0.21 ± 0.04 | 1.53 ± 0.38 |
| sumtrans18:1 | 0.14 ± 0.02 | 1.27 ± 0.15 | 0.22 ± 0.02 | 1.13 ± 0.16 | 0.22 ± 0.02 | 0.99 ± 0.16 |
| 9c-20:1 | 0.16 ± 0.02 | 1.79 ± 0.49 | 0.21 ± 0.04 | 1.35 ± 0.28 | 0.21 ± 0.03 | 1.26 ± 0.25 |
| 11c-20:1 | 0.12 ± 0.03 | 1.32 ± 0.44 | 0.16 ± 0.02 | 0.88 ± 0.15 | 0.18 ± 0.03 | 1.00 ± 0.17 |
| MUFA | 0.15 ± 0.04 | 1.22 ± 0.29 | 0.15 ± 0.04 | 0.63 ± 0.19 | 0.15 ± 0.04 | 0.86 ± 0.24 |
| 9c,13t+8t,12c-18:2 | 0.12 ± 0.03 | 1.24 ± 0.28 | 0.18 ± 0.04 | 0.87 ± 0.19 | 0.18 ± 0.04 | 0.98 ± 0.23 |
| 9c,15c-18:2 | 0.09 ± 0.03 | 1.28 ± 0.55 | 0.17 ± 0.02 | 1.07 ± 0.12 | 0.18 ± 0.03 | 1.13 ± 0.14 |
| 8t,13c-18:2 | 0.10 ± 0.02 | 1.25 ± 0.28 | 0.17 ± 0.03 | 1.07 ± 0.18 | 0.17 ± 0.03 | 0.97 ± 0.14 |
| 11t,15c-18:2 | 0.14 ± 0.03 | 1.39 ± 0.33 | 0.17 ± 0.03 | 0.97 ± 0.22 | 0.18 ± 0.03 | 1.04 ± 0.25 |
| 9c,11t+9t,11c- 18:2 | 0.03 ± 0.04 | 0.24 ± 0.60 | 0.12 ± 0.03 | 0.93 ± 0.28 | 0.12 ± 0.03 | 1.11 ± 0.42 |
| 6t,8t-18:2 | 0.07 ± 0.04 | 0.40 ± 0.37 | 0.07 ± 0.04 | 0.27 ± 0.17 | 0.06 ± 0.05 | 0.91 ± 0.65 |
| 7t,9c-18:2 | 0.29 ± 0.06 | 1.09 ± 0.22 | 0.32 ± 0.06 | 0.93 ± 0.15 | 0.32 ± 0.06 | 1.04 ± 0.19 |
| 12t,14t-18:2 | 0.01 ± 0.04 | -0.21 ± 0.43 | 0.07 ± 0.05 | 0.33 ± 0.25 | 0.06 ± 0.05 | 0.60 ± 0.44 |
| 11t,13t-18:2 | 0.15 ± 0.05 | 1.51 ± 0.70 | 0.15 ± 0.04 | 0.76 ± 0.15 | 0.15 ± 0.04 | 1.13 ± 0.31 |
| 10t,12t-18:2 | 0.07 ± 0.06 | -0.59 ± 3.87 | 0.03 ± 0.06 | 0.14 ± 3.18 | 0.03 ± 0.06 | 0.45 ± 1.60 |
| 9t,11t-18:2 | -0.06 ± 0.05 | -0.64 ± 0.37 | 0.00 ± 0.05 | -0.11 ± 0.16 | -0.03 ± 0.05 | -0.56 ± 0.74 |
| 8t,10t-18:2 | 0.10 ± 0.04 | 1.41 ± 0.91 | 0.08 ± 0.04 | 0.78 ± 0.39 | 0.08 ± 0.04 | 0.71 ± 0.36 |
| 7t,9t-18:2 | 0.06 ± 0.06 | 2.66 ± 2.83 | 0.01 ± 0.06 | 0.91 ± 1.50 | 0.00 ± 0.06 | 0.90 ± 1.14 |
| 12t,14c+12c,14t -18:2 | 0.00 ± 0.06 | -1.10 ± 1.52 | 0.09 ± 0.07 | 0.66 ± 1.06 | 0.09 ± 0.06 | 1.78 ± 1.35 |
| 11t,13c+11c,13t -18:2 | 0.21 ± 0.02 | 1.64 ± 0.32 | 0.21 ± 0.03 | 1.19 ± 0.18 | 0.21 ± 0.03 | 1.57 ± 0.24 |
| 10t,12c-18:2 | 0.22 ± 0.04 | 1.45 ± 0.34 | 0.25 ± 0.04 | 1.15 ± 0.16 | 0.26 ± 0.04 | 1.32 ± 0.22 |
| 8t,10c-18:2 | 0.11 ± 0.05 | 1.81 ± 1.04 | 0.11 ± 0.05 | 1.02 ± 0.43 | 0.10 ± 0.05 | 1.39 ± 0.63 |
| Total CLA | 0.13 ± 0.05 | 0.91 ± 0.45 | 0.20 ± 0.05 | 1.02 ± 0.25 | 0.20 ± 0.05 | 1.18 ± 0.38 |
| 18:2n-6 | 0.15 ± 0.02 | 1.30 ± 0.14 | 0.26 ± 0.03 | 1.09 ± 0.14 | 0.26 ± 0.03 | 0.92 ± 0.10 |
| 18:3n-3 | 0.17 ± 0.02 | 1.26 ± 0.17 | 0.25 ± 0.03 | 1.07 ± 0.17 | 0.25 ± 0.03 | 1.11 ± 0.18 |
| 18:3n-6 | 0.02 ± 0.02 | 0.62 ± 1.91 | 0.02 ± 0.02 | 1.08 ± 0.85 | 0.03 ± 0.02 | 1.16 ± 0.80 |
| 20:2n-6 | 0.09 ± 0.03 | 0.78 ± 0.27 | 0.03 ± 0.02 | 0.08 ± 0.13 | 0.03 ± 0.03 | 0.06 ± 0.24 |
| 20:3n-6 | 0.03 ± 0.03 | 0.51 ± 0.67 | 0.07 ± 0.03 | 0.78 ± 0.35 | 0.06 ± 0.03 | 1.62 ± 0.74 |
| 20:3n-9 | 0.05 ± 0.04 | 1.15 ± 0.83 | -0.01 ± 0.03 | 0.05 ± 0.30 | -0.01 ± 0.03 | 0.14 ± 0.79 |
| 20:4n-6 | -0.04 ± 0.01 | -0.58 ± 0.26 | 0.04 ± 0.03 | 0.42 ± 0.39 | 0.03 ± 0.03 | 0.63 ± 0.54 |
| 22:4n-6 | 0.04 ± 0.02 | 0.57 ± 0.27 | 0.07 ± 0.03 | 0.61 ± 0.31 | 0.07 ± 0.03 | 0.96 ± 0.44 |
| 22:5n-3 | 0.01 ± 0.03 | 0.19 ± 1.04 | 0.05 ± 0.03 | 0.40 ± 0.30 | 0.05 ± 0.03 | 0.83 ± 0.56 |
| PUFA | 0.15 ± 0.02 | 1.31 ± 0.15 | 0.25 ± 0.03 | 1.09 ± 0.14 | 0.26 ± 0.03 | 0.93 ± 0.10 |
| n-3 | 0.17 ± 0.02 | 1.28 ± 0.18 | 0.24 ± 0.03 | 1.10 ± 0.18 | 0.24 ± 0.03 | 1.15 ± 0.20 |
| n-6 | 0.15 ± 0.02 | 1.28 ± 0.14 | 0.25 ± 0.03 | 1.08 ± 0.14 | 0.26 ± 0.03 | 0.93 ± 0.11 |
| n-6/n-3 | 0.10 ± 0.02 | 0.90 ± 0.18 | 0.07 ± 0.06 | - | 0.04 ± 0.04 | 0.53 ± 0.20 |
| P/S | 0.14 ± 0.02 | 1.29 ± 0.12 | 0.23 ± 0.03 | 0.91 ± 0.14 | 0.24 ± 0.03 | 0.84 ± 0.10 |
| P/(S+B) | 0.14 ± 0.02 | 0.91 ± 0.12 | 0.24 ± 0.03 | 0.92 ± 0.15 | 0.24 ± 0.03 | 0.87 ± 0.11 |
| HI | 0.17 ± 0.03 | 1.30 ± 0.20 | 0.17 ± 0.03 | 0.63 ± 0.10 | 0.22 ± 0.03 | 0.84 ± 0.10 |

Table S2. Pearson’s correlation coefficients between estimated breeding values and adjusted phenotypes [$r_{\left( GEBV,y \right)}$] and regression coefficients by regressing adjusted phenotypes on estimated breeding values [$b_{\left( GEBV,y \right)}$] for fatty acid traits in the *longissimus lumborum* muscle.

|  | PBLUP | | GBLUP | | BayesCπ | |
| --- | --- | --- | --- | --- | --- | --- |
| Trait^2^ | $r_{\left( GEBV, y \right)}$ | $b_{\left( GEBV, y \right)}$ | $r_{\left( GEBV, y \right)}$ | $b_{\left( GEBV,y \right)}$ | $r_{\left( GEBV,y \right)}$ | $b_{\left( GEBV,y \right)}$ |
| 10:0 | 0.16 ± 0.03 | 1.00 ± 0.16 | 0.23 ± 0.04 | 0.74 ± 0.11 | 0.32 ± 0.03 | 0.98 ± 0.09 |
| 12:0 | 0.17 ± 0.02 | 1.04 ± 0.17 | 0.23 ± 0.04 | 0.64 ± 0.09 | 0.40 ± 0.03 | 0.97 ± 0.10 |
| 13:0 | -0.02 ± 0.02 | -0.73 ± 0.64 | 0.12 ± 0.03 | 0.92 ± 0.18 | 0.13 ± 0.03 | 0.97 ± 0.24 |
| 14:0 | 0.20 ± 0.03 | 1.03 ± 0.14 | 0.33 ± 0.04 | 0.87 ± 0.10 | 0.53 ± 0.03 | 1.10 ± 0.07 |
| 15:0 | 0.13 ± 0.03 | 1.02 ± 0.21 | 0.27 ± 0.04 | 0.99 ± 0.14 | 0.33 ± 0.04 | 1.05 ± 0.15 |
| 16:0 | 0.15 ± 0.02 | 0.95 ± 0.13 | 0.23 ± 0.03 | 0.74 ± 0.10 | 0.32 ± 0.03 | 0.99 ± 0.09 |
| 17:0 | 0.16 ± 0.03 | 1.08 ± 0.22 | 0.22 ± 0.02 | 0.79 ± 0.07 | 0.25 ± 0.02 | 0.83 ± 0.08 |
| 18:0 | 0.15 ± 0.03 | 1.22 ± 0.18 | 0.21 ± 0.03 | 1.00 ± 0.14 | 0.23 ± 0.03 | 0.88 ± 0.09 |
| 19:0 | 0.05 ± 0.03 | 0.42 ± 0.28 | 0.05 ± 0.04 | 0.20 ± 0.17 | 0.05 ± 0.04 | 0.33 ± 0.25 |
| 20:0 | 0.10 ± 0.02 | 1.21 ± 0.27 | 0.10 ± 0.02 | 0.60 ± 0.15 | 0.10 ± 0.02 | 0.68 ± 0.17 |
| 22:0 | 0.05 ± 0.02 | 1.13 ± 0.37 | 0.13 ± 0.02 | 1.12 ± 0.18 | 0.13 ± 0.02 | 0.92 ± 0.20 |
| 24:0 | 0.11 ± 0.03 | 1.29 ± 0.38 | 0.12 ± 0.03 | 0.69 ± 0.16 | 0.13 ± 0.03 | 0.73 ± 0.17 |
| SFA | 0.09 ± 0.02 | 0.81 ± 0.24 | 0.24 ± 0.04 | 0.83 ± 0.14 | 0.28 ± 0.04 | 0.92 ± 0.13 |
| iso14:0 | 0.09 ± 0.02 | 1.91 ± 0.36 | 0.06 ± 0.02 | 0.73 ± 0.28 | 0.06 ± 0.02 | 1.41 ± 0.59 |
| iso15:0 | 0.08 ± 0.01 | 0.88 ± 0.16 | 0.10 ± 0.03 | 0.53 ± 0.19 | 0.09 ± 0.03 | 0.65 ± 0.25 |
| ai15:0 | 0.10 ± 0.02 | 0.84 ± 0.21 | 0.20 ± 0.03 | 0.83 ± 0.11 | 0.21 ± 0.03 | 0.86 ± 0.11 |
| iso16:0 | 0.07 ± 0.02 | 0.86 ± 0.26 | 0.13 ± 0.03 | 0.79 ± 0.17 | 0.14 ± 0.02 | 0.77 ± 0.15 |
| iso17:0 | 0.08 ± 0.02 | 1.68 ± 0.53 | 0.10 ± 0.02 | 0.95 ± 0.19 | 0.09 ± 0.02 | 0.71 ± 0.15 |
| ai17:0 | 0.07 ± 0.02 | 0.61 ± 0.16 | 0.15 ± 0.02 | 0.74 ± 0.09 | 0.15 ± 0.02 | 0.77 ± 0.10 |
| iso18:0 | 0.10 ± 0.02 | 0.94 ± 0.28 | 0.15 ± 0.03 | 0.78 ± 0.15 | 0.17 ± 0.03 | 0.87 ± 0.15 |
| BFA | 0.07 ± 0.02 | 0.84 ± 0.27 | 0.13 ± 0.02 | 0.83 ± 0.10 | 0.13 ± 0.02 | 0.79 ± 0.10 |
| SFA+BFA | 0.09 ± 0.02 | 0.81 ± 0.24 | 0.23 ± 0.04 | 0.82 ± 0.15 | 0.28 ± 0.04 | 0.92 ± 0.14 |
| 9c-14:1 | 0.21 ± 0.04 | 1.08 ± 0.19 | 0.26 ± 0.03 | 0.97 ± 0.09 | 0.42 ± 0.02 | 1.07 ± 0.08 |
| 9c-15:1 | 0.07 ± 0.03 | 2.69 ± 1.61 | 0.10 ± 0.03 | 3.24 ± 1.15 | 0.11 ± 0.03 | 4.67 ± 1.49 |
| 7c-16:1 | 0.09 ± 0.04 | 0.92 ± 0.54 | 0.12 ± 0.03 | 0.69 ± 0.15 | 0.13 ± 0.03 | 0.64 ± 0.15 |
| 9c-16:1 | 0.23 ± 0.02 | 1.25 ± 0.10 | 0.30 ± 0.02 | 0.83 ± 0.04 | 0.39 ± 0.02 | 0.99 ± 0.07 |
| 11t-16:1 | 0.02 ± 0.02 | 1.93 ± 1.55 | 0.03 ± 0.02 | 0.91 ± 0.74 | 0.03 ± 0.02 | 0.99 ± 0.70 |
| 12c-16:1 | 0.18 ± 0.04 | 1.10 ± 0.19 | 0.23 ± 0.03 | 0.96 ± 0.09 | 0.40 ± 0.02 | 1.06 ± 0.05 |
| 7c-17:1 | 0.00 ± 0.02 | - | 0.05 ± 0.03 | - | 0.05 ± 0.03 | 0.57 ± 0.30 |
| 9c-17:1 | 0.14 ± 0.03 | 1.35 ± 0.36 | 0.16 ± 0.03 | 0.76 ± 0.17 | 0.16 ± 0.03 | 0.85 ± 0.18 |
| 9c-18:1 | 0.10 ± 0.02 | 0.95 ± 0.22 | 0.18 ± 0.04 | 0.76 ± 0.18 | 0.26 ± 0.03 | 0.88 ± 0.13 |
| 11c-18:1 | 0.16 ± 0.01 | 1.52 ± 0.15 | 0.24 ± 0.04 | 1.03 ± 0.19 | 0.25 ± 0.04 | 0.92 ± 0.15 |
| 12c-18:1 | 0.04 ± 0.04 | 0.63 ± 0.64 | 0.07 ± 0.03 | 0.62 ± 0.25 | 0.07 ± 0.04 | 0.61 ± 0.32 |
| 13c-18:1 | 0.16 ± 0.02 | 1.12 ± 0.11 | 0.27 ± 0.04 | 1.04 ± 0.15 | 0.38 ± 0.03 | 1.08 ± 0.09 |
| 14c-18:1 | 0.07 ± 0.04 | 0.90 ± 0.56 | 0.07 ± 0.03 | 0.78 ± 0.33 | 0.07 ± 0.03 | 0.86 ± 0.34 |
| 15c-18:1 | 0.10 ± 0.03 | 1.02 ± 0.28 | 0.14 ± 0.03 | 0.89 ± 0.20 | 0.13 ± 0.03 | 0.92 ± 0.21 |
| 6t+8t-18:1 | 0.09 ± 0.03 | 1.15 ± 0.51 | 0.13 ± 0.02 | 1.13 ± 0.17 | 0.14 ± 0.02 | 0.95 ± 0.15 |
| 9t-18:1 | 0.12 ± 0.04 | 1.42 ± 0.55 | 0.14 ± 0.02 | 1.22 ± 0.14 | 0.15 ± 0.02 | 1.06 ± 0.15 |
| 10t-18:1 | 0.16 ± 0.02 | 1.67 ± 0.17 | 0.25 ± 0.02 | 1.42 ± 0.13 | 0.25 ± 0.02 | 1.05 ± 0.10 |
| 11t-18:1 | 0.15 ± 0.02 | 1.10 ± 0.16 | 0.19 ± 0.02 | 0.96 ± 0.12 | 0.18 ± 0.02 | 0.93 ± 0.14 |
| 12t-18:1 | 0.02 ± 0.02 | 0.43 ± 0.68 | 0.10 ± 0.04 | 1.35 ± 0.46 | 0.10 ± 0.04 | 1.40 ± 0.51 |
| 15t-18:1 | -0.05 ± 0.03 | -4.39 ± 7.97 | -0.01 ± 0.03 | -1.20 ± 2.95 | 0.00 ± 0.03 | -0.35 ± 5.58 |
| 16t-18:1 | 0.14 ± 0.02 | 1.35 ± 0.23 | 0.20 ± 0.03 | 1.13 ± 0.19 | 0.21 ± 0.03 | 1.14 ± 0.21 |
| sumtrans18:1 | 0.15 ± 0.01 | 1.64 ± 0.11 | 0.22 ± 0.02 | 1.36 ± 0.15 | 0.22 ± 0.02 | 1.09 ± 0.12 |
| 9c-20:1 | -0.01 ± 0.03 | 0.01 ± 0.47 | 0.00 ± 0.03 | 0.05 ± 0.16 | 0.00 ± 0.03 | 0.03 ± 0.33 |
| 11c-20:1 | 0.22 ± 0.03 | 1.57 ± 0.33 | 0.23 ± 0.03 | 0.80 ± 0.09 | 0.29 ± 0.02 | 1.07 ± 0.09 |
| MUFA | 0.08 ± 0.02 | 0.88 ± 0.22 | 0.17 ± 0.05 | 0.68 ± 0.24 | 0.21 ± 0.04 | 0.80 ± 0.17 |
| 9c,13t+8t,12c-18:2 | 0.07 ± 0.03 | 0.79 ± 0.33 | 0.18 ± 0.04 | 0.96 ± 0.23 | 0.18 ± 0.04 | 0.92 ± 0.22 |
| 9c,15c-18:2 | 0.11 ± 0.02 | 1.08 ± 0.20 | 0.19 ± 0.03 | 0.97 ± 0.15 | 0.22 ± 0.03 | 1.05 ± 0.15 |
| 8t,13c-18:2 | 0.01 ± 0.03 | 0.06 ± 1.41 | 0.09 ± 0.01 | 2.14 ± 0.30 | 0.10 ± 0.01 | 2.27 ± 0.34 |
| 11t,15c-18:2 | 0.15 ± 0.02 | 1.27 ± 0.19 | 0.21 ± 0.03 | 1.03 ± 0.14 | 0.22 ± 0.02 | 0.96 ± 0.14 |
| 9c,11t+9t,11c-18:2 | 0.10 ± 0.02 | 1.14 ± 0.30 | 0.10 ± 0.03 | 0.64 ± 0.18 | 0.10 ± 0.02 | 0.72 ± 0.19 |
| 6t,8t-18:2 | -0.03 ± 0.03 | -7.40 ± 9.95 | -0.04 ± 0.03 | -7.18 ± 7.92 | -0.03 ± 0.03 | -2.51 ± 2.68 |
| 7t,9c-18:2 | 0.08 ± 0.04 | 2.97 ± 2.27 | 0.06 ± 0.05 | 2.89 ± 2.51 | 0.05 ± 0.05 | 1.37 ± 0.93 |
| 12t,14t-18:2 | 0.04 ± 0.03 | 0.87 ± 0.75 | 0.08 ± 0.04 | 0.74 ± 0.27 | 0.08 ± 0.03 | 0.96 ± 0.33 |
| 11t,13t-18:2 | 0.04 ± 0.04 | 0.29 ± 0.46 | 0.02 ± 0.04 | 0.19 ± 0.23 | 0.02 ± 0.04 | 0.31 ± 0.29 |
| 10t,12t-18:2 | 0.03 ± 0.03 | 0.54 ± 0.56 | 0.03 ± 0.04 | 0.46 ± 0.47 | 0.03 ± 0.04 | 0.79 ± 0.63 |
| 9t,11t-18:2 | 0.03 ± 0.03 | 0.52 ± 0.65 | 0.00 ± 0.02 | -0.01 ± 0.22 | 0.00 ± 0.02 | 0.13 ± 0.32 |
| 8t,10t-18:2 | 0.05 ± 0.02 | 5.93 ± 3.86 | 0.00 ± 0.02 | 0.08 ± 0.88 | 0.01 ± 0.02 | 0.10 ± 0.20 |
| 7t,9t-18:2 | 0.04 ± 0.03 | 1.14 ± 0.92 | -0.05 ± 0.03 | -1.02 ± 0.51 | -0.06 ± 0.03 | -2.26 ± 1.04 |
| 12t,14c+12c,14t -18:2 | 0.03 ± 0.04 | 0.48 ± 0.59 | 0.02 ± 0.03 | 0.16 ± 0.33 | 0.02 ± 0.03 | 0.35 ± 0.37 |
| 11t,13c+11c,13t -18:2 | 0.06 ± 0.04 | 1.68 ± 1.19 | 0.11 ± 0.02 | 1.34 ± 0.32 | 0.11 ± 0.02 | 1.31 ± 0.31 |
| 10t,12c-18:2 | 0.01 ± 0.03 | 0.36 ± 1.53 | 0.06 ± 0.04 | 1.58 ± 1.09 | 0.06 ± 0.04 | 1.75 ± 0.93 |
| 8t,10c-18:2 | 0.12 ± 0.04 | 0.86 ± 0.30 | 0.07 ± 0.03 | 0.31 ± 0.13 | 0.06 ± 0.03 | 0.47 ± 0.21 |
| Total CLA | 0.04 ± 0.02 | 0.72 ± 0.41 | 0.02 ± 0.02 | 0.19 ± 0.19 | 0.02 ± 0.02 | 0.27 ± 0.25 |
| 18:2n-6 | 0.09 ± 0.03 | 1.43 ± 0.52 | 0.18 ± 0.03 | 1.27 ± 0.21 | 0.20 ± 0.02 | 0.98 ± 0.16 |
| 18:3n-3 | 0.13 ± 0.03 | 1.09 ± 0.26 | 0.15 ± 0.02 | 0.86 ± 0.10 | 0.15 ± 0.02 | 1.01 ± 0.14 |
| 18:3n-6 | 0.08 ± 0.02 | 1.41 ± 0.33 | 0.14 ± 0.02 | 1.23 ± 0.19 | 0.17 ± 0.02 | 1.16 ± 0.16 |
| 20:2n-6 | 0.02 ± 0.04 | 0.81 ± 1.09 | 0.03 ± 0.03 | 0.55 ± 0.45 | 0.04 ± 0.03 | 0.48 ± 0.35 |
| 20:3n-6 | 0.08 ± 0.02 | 0.99 ± 0.29 | 0.18 ± 0.02 | 1.25 ± 0.20 | 0.20 ± 0.03 | 1.01 ± 0.17 |
| 20:3n-9 | 0.05 ± 0.02 | 1.05 ± 0.37 | 0.15 ± 0.03 | 1.38 ± 0.31 | 0.15 ± 0.03 | 1.11 ± 0.23 |
| 20:4n-6 | 0.04 ± 0.02 | 0.86 ± 0.35 | 0.15 ± 0.03 | 1.35 ± 0.29 | 0.16 ± 0.03 | 1.01 ± 0.20 |
| 20:5n-3 | 0.07 ± 0.02 | 1.08 ± 0.42 | 0.02 ± 0.03 | 1.23 ± 0.32 | 0.02 ± 0.03 | 1.38 ± 2.62 |
| 22:4n-6 | 0.07 ± 0.03 | 0.85 ± 0.32 | 0.17 ± 0.03 | 1.02 ± 0.19 | 0.18 ± 0.04 | 1.03 ± 0.25 |
| 22:5n-3 | 0.06 ± 0.03 | 1.32 ± 0.49 | 0.17 ± 0.03 | 1.30 ± 0.21 | 0.17 ± 0.03 | 0.90 ± 0.16 |
| 22:6n-3 | 0.00 ± 0.02 | 1.02 ± 0.29 | 0.04 ± 0.03 | 0.91 ± 0.13 | 0.04 ± 0.03 | 0.44 ± 0.30 |
| PUFA | 0.08 ± 0.02 | 1.23 ± 0.46 | 0.17 ± 0.02 | 1.25 ± 0.21 | 0.18 ± 0.02 | 1.02 ± 0.17 |
| n-3 | 0.09 ± 0.02 | 0.53 ± 0.21 | 0.16 ± 0.02 | 0.38 ± 0.09 | 0.16 ± 0.02 | 0.91 ± 0.14 |
| n-6 | 0.08 ± 0.02 | 1.13 ± 0.42 | 0.17 ± 0.02 | 1.44 ± 0.18 | 0.18 ± 0.02 | 0.98 ± 0.15 |
| n-6/n-3 | 0.07 ± 0.03 | 1.13 ± 0.42 | 0.10 ± 0.03 | 1.43 ± 0.18 | 0.09 ± 0.02 | 0.37 ± 0.09 |
| P/S | 0.08 ± 0.02 | 1.04 ± 0.14 | 0.21 ± 0.02 | 0.89 ± 0.12 | 0.21 ± 0.02 | 1.09 ± 0.15 |
| P/(S+B) | 0.08 ± 0.02 | 1.49 ± 0.40 | 0.21 ± 0.02 | 1.42 ± 0.22 | 0.21 ± 0.02 | 1.11 ± 0.16 |
| HI | 0.15 ± 0.02 | 1.40 ± 0.38 | 0.28 ± 0.04 | 1.27 ± 0.20 | 0.40 ± 0.04 | 1.06 ± 0.10 |
